# Supplementary material for: Phenotypic and genomic characterization of ST11-K1 CR-hvKP with highly homologous blaKPC-2-bearing plasmids in China
Source: mSystems. 2024 Nov 18;9(12):e01101-24. doi: 10.1128/msystems.01101-24 (PMC11651102; doi:10.1128/msystems.01101-24)
Supplement: Captions — Supplemental table captions. [file msystems.01101-24-s0001.docx]

**Supplementary legends**

**Title****:** Phenotypic and genomic characterization of ST11-K1 CR-hvKP with highly homologous *bla*_KPC-2_-bearing plasmids in China

**Authors:** Yu-Ling Han^1, 2^, Hua Wang^1, 3^, Hong-Zhe Zhu^1, 2^, Ying-Ying Lv^1^, Wen Zhao^1^, Yan-Yan Wang^1^, Jian-Xun Wen^4^, Zhi-De Hu^1^, Jun-Rui Wang^1^ and Wen-Qi Zheng^1, 2*^

**Affiliations:** ^1^ Department of Laboratory Medicine, the Affiliated Hospital of Inner Mongolia Medical University, Hohhot, China. ^2^ Department of Parasitology, the Basic Medical College of Inner Mongolia Medical University, Hohhot, China. ^3^ Medical Research Center, Beijing Institute of Respiratory Medicine and Beijing Chao-Yang Hospital, Capital Medical University, Beijing, China. ^4^ Department of Medical Experiment Center, the Basic Medical Sciences College of Inner Mongolia Medical University, Hohhot, China.

**Supplemental** **legends for figures and tables.**

**Table S1:** The assembly results of seven CR-hvKP strains by Unicycler.

**Table S2:** BLAST searching of the NCBI nucleotide database of the plasmids assembled in CR-hvKP221, CR-hvKP005, CR-hvKP006, CR-hvKP26, CR-hvKP128, CR-hvKP132, and CR-hvKP173 stains.

**Table S3:** The function of resistance genes and virulence genes detected in CR-hvKP221, CR-hvKP005, CR-hvKP006, CR-hvKP26, CR-hvKP128, CR-hvKP132, and CR-hvKP173 stains.

**Table S4**：All the SNP loci were detected in 402 genomes using the MUMmer alignment software. The alignment results of the genome coverage between CR-hvKP221 and 402 strains are shown in the last column.
