# Supplementary material for: Phenotypic and genomic characterization of ST11-K1 CR-hvKP with highly homologous blaKPC-2-bearing plasmids in China
Source: mSystems. 2024 Nov 18;9(12):e01101-24. doi: 10.1128/msystems.01101-24 (PMC11651102; doi:10.1128/msystems.01101-24)
Supplement: Table S1 — The assembly results of seven CR-hvKP strains by Unicycler. [file msystems.01101-24-s0002.docx]

**Table S1** The assembly results of seven CR-hvKP strains by Unicycler.

| Strains | CR-hvKP005 | CR-hvKP006 | CR-hvKP26 | CR-hvKP128 | CR-hvKP132 | CR-hvKP173 | CR-hvKP221 |
| --- | --- | --- | --- | --- | --- | --- | --- |
| Total Number | 4 | 4 | 5 | 3 | 5 | 6 | 7 |
| Number of scaffolds | 4 | 4 | 5 | 3 | 5 | 6 | 7 |
| Number of circularised replicons | 4 | 4 | 5 | 3 | 5 | 6 | 7 |
| Total Length/ Predicted genome size (bp) | 5583382 | 5686131 | 5706345 | 5618685 | 5706345 | 5893274 | 6010146 |
| Average Length (bp) | 1395845.5 | 1421532.75 | 1116814.8 | 1872895 | 1141269 | 982212.33 | 858592.28 |
| Mean GC Content (%) | 57.3 | 57.21 | 57.28 | 57.27 | 57.21 | 56.95 | 56.78 |
| N50 length (bp) | 5496361 | 5467613 | 5422087 | 5453906 | 5533017 | 5464083 | 5517123 |
| N90 length (bp) | 5496361 | 5467613 | 5422087 | 5453906 | 5533017 | 5464083 | 5517123 |
| Gene number | 5279 | 5366 | 5286 | 5314 | 5411 | 5633 | 5779 |
| Gene total length (bp) | 4857309 | 4916916 | 4863360 | 4885971 | 4958367 | 5102673 | 5191971 |
| Gene average length (bp) | 920 | 916 | 920 | 919 | 916 | 905 | 898 |
| Gene density genes per kb | 0.945 | 0.943 | 0.946 | 0.945 | 0.948 | 0.955 | 0.961 |
| GC content in gene region (%) | 58.6 | 58.5 | 58.6 | 58.5 | 58.5 | 58.2 | 58.1 |
| Gene/Geonme(%) | 87 | 86.5 | 87.1 | 87 | 86.9 | 86.6 | 86.4 |
| Plus Gene number | 2758 | 2829 | 2837 | 2780 | 2827 | 2914 | 3112 |
| Minus Gene number | 2521 | 2537 | 2449 | 2534 | 2584 | 2719 | 2667 |
| Median coverage of the scaffolds | 415X | 331.4X | 340.6X | 341.2X | 374.2X | 332.9X | 369X |
