# Supplementary material for: Phenotypic and genomic characterization of ST11-K1 CR-hvKP with highly homologous blaKPC-2-bearing plasmids in China
Source: mSystems. 2024 Nov 18;9(12):e01101-24. doi: 10.1128/msystems.01101-24 (PMC11651102; doi:10.1128/msystems.01101-24)
Supplement: Table S2 — BLAST searching of the NCBI nucleotide database of the plasmids assembled in CR-hvKP221, CR-hvKP005, CR-hvKP006, CR-hvKP26, CR-hvKP128, CR-hvKP132, and CR-hvKP173 strains. [file msystems.01101-24-s0003.docx]

**Table S2-1** Molecular characterization of plasmids of the CR-hvKP isolates

| Isolates | Plasmids | Replicon | Size (bp) | GC (%) | Accession number |
| --- | --- | --- | --- | --- | --- |
| CR-hvKP005 | pCR-hvKP005-KPC-P1 | IncFII, IncR | 69,455 | 54.3 | CP119014 |
|  | pCR-hvKP005-P2 | ColRNAI | 11,970 | 55.58 | CP119015 |
|  | pCR-hvKP005-P3 | ND | 5,596 | 51.14 | CP119016 |
| CR-hvKP006 | pCR-hvKP006-KPC-P1 | IncFII, IncR | 111,731 | 55.27 | CP119018 |
|  | pCR-hvKP006-P2 | ND | 96,727 | 51.67 | CP119019 |
|  | pCR-hvKP006-P3 | ColRNAI | 10,060 | 55.06 | CP119020 |
| CR-hvKP26 | pCR-hvKP26-KPC-P1 | IncFII | 89,906 | 53.87 | CP119022 |
|  | pCR-hvKP26-P2 | ND | 54,515 | 52.75 | CP119023 |
|  | pCR-hvKP26-P3 | ColRNAI | 11,970 | 55.58 | CP119024 |
|  | pCR-hvKP26-P4 | ND | 5,596 | 51.14 | CP119025 |
| CR-hvKP128 | pCR-hvKP128-KPC-P1 | IncFII, IncR | 154,719 | 53.08 | CP119027 |
|  | pCR-hvKP128-P2 | ColRNAI | 10,060 | 55.06 | CP119028 |
| CR-hvKP132 | pCR-hvKP132-KPC-P1 | IncFII, IncR | 154,719 | 53.08 | CP119030 |
|  | pCR-hvKP132-P2 | ColRNAI | 10,060 | 55.06 | CP119031 |
|  | pCR-hvKP132-P3 | ND | 5,596 | 51.14 | CP119032 |
|  | pCR-hvKP132-P4 | ND | 2,953 | 64.75 | CP119033 |
| CR-hvKP173 | pCR-hvKP173-Vir-P1 | IncHI1B, repB | 178,873 | 49.37 | CP119035 |
|  | pCR-hvKP173-KPC-P2 | IncFII, IncR | 144,880 | 53.49 | CP119036 |
|  | pCR-hvKP173-P3 | ND | 87,872 | 53.93 | CP119037 |
|  | pCR-hvKP173-P4 | ColRNAI | 11,970 | 55.58 | CP119038 |
|  | pCR-hvKP173-P5 | ND | 5,596 | 51.14 | CP119039 |
| CR-hvKP221 | pCR-hvKP221-Vir-P1 | IncHI1B, repB | 145,168 | 50.3 | CP119041 |
|  | pCR-hvKP221-Vir-P2 | IncFIB, IncFIC | 140,816 | 50.68 | CP119042 |
|  | pCR-hvKP221-KPC-P3 | IncFII, IncR | 136,943 | 53.02 | CP119043 |
|  | pCR-hvKP221-P4 | ND | 10,060 | 52.89 | CP119044 |
|  | pCR-hvKP221-P5 | ColRNAI | 10,060 | 55.08 | CP119045 |
|  | pCR-hvKP221-P6 | ND | 4,073 | 51.44 | CP119046 |

**Table S2-2** BLAST searching of the NCBI nucleotide database of the plasmids assembled in CR-hvKP221, CR-hvKP005, CR-hvKP006, CR-hvKP26, CR-hvKP128, CR-hvKP132, and CR-hvKP173 stains.

| Plasmids | Description | Scientific Name | Max Score | Total Score | Query Cover | E value | Per. ident | Query length | Alignment length | Accession |
| --- | --- | --- | --- | --- | --- | --- | --- | --- | --- | --- |
| pCR-hvKP005-KPC-P1 | *Klebsiella pneumoniae* strain KP18-238 plasmid pKP18-238-4, complete sequence | *Klebsiella pneumoniae* | 10334 | 20671 | 100% | 0 | 100 | 69455 | 11192 | CP082017.1 |
|  | *Klebsiella pneumoniae* strain KP20194c5 plasmid pKP20194c5-p5, complete sequence | *Klebsiella pneumoniae* | 10325 | 10325 | 99% | 0 | 99.98 | 69455 | 5596 | CP054743.1 |
|  | *Klebsiella pneumoniae* strain 150040X1B1 plasmid p2_150040X1B1, complete sequence | *Klebsiella pneumoniae* | 10296 | 10296 | 99% | 0 | 100 | 69455 | 5596 | CP101730.1 |
|  | *Klebsiella pneumoniae* *subsp.* pneumoniae strain DD02280 plasmid pDD02280-4, complete sequence | *Klebsiella pneumoniae subsp.* pneumoniae | 10257 | 10336 | 100% | 0 | 100 | 69455 | 5596 | CP087627.1 |
|  | *Klebsiella pneumoniae* strain RIVM_C014947 plasmid pRIVM_C014947_4 | *Klebsiella pneumoniae* | 10168 | 10325 | 100% | 0 | 99.96 | 69455 | 5596 | MT560067.1 |
|  | *Klebsiella pneumoniae* *subsp.* pneumoniae strain SCKP020079 plasmid p2_020079, complete sequence | *Klebsiella pneumoniae* *subsp.* pneumoniae | 10126 | 10336 | 100% | 0 | 100 | 69455 | 5596 | CP029380.1 |
|  | *Klebsiella pneumoniae* strain WCHKP040035 plasmid p2_040035, complete sequence | *Klebsiella pneumoniae* | 10124 | 10336 | 100% | 0 | 100 | 69455 | 5596 | CP028795.2 |
|  | *Klebsiella pneumoniae* isolate 392 genome assembly, plasmid: P4 | *Klebsiella pneumoniae* | 10109 | 10304 | 99% | 0 | 99.91 | 69455 | 5599 | OW848883.1 |
|  | *Klebsiella pneumoniae* isolate 392 genome assembly, plasmid: P3 | *Klebsiella pneumoniae* | 10109 | 10304 | 99% | 0 | 99.91 | 69455 | 5599 | OW849089.1 |
|  | *Klebsiella pneumoniae* strain KP20194a plasmid pKP20194a-p5, complete sequence | *Klebsiella pneumoniae* | 10098 | 10330 | 100% | 0 | 99.98 | 69455 | 5596 | CP054785.1 |
|  | *Klebsiella pneumoniae* *subsp.* pneumoniae strain WCHKP020120 plasmid p3_020120, complete sequence | *Klebsiella pneumoniae* *subsp.* pneumoniae | 10067 | 10336 | 100% | 0 | 100 | 69455 | 5596 | CP043361.1 |
|  | *Klebsiella pneumoniae* strain JX-CR-hvKP-10 plasmid pJX10-4, complete sequence | *Klebsiella pneumoniae* | 10046 | 10336 | 100% | 0 | 100 | 69455 | 5596 | CP064262.1 |
|  | *Klebsiella pneumoniae* isolate 392 genome assembly, plasmid: P4 | *Klebsiella pneumoniae* | 9963 | 10304 | 99% | 0 | 99.91 | 69455 | 5599 | OW849049.1 |
|  | *Klebsiella pneumoniae* strain ZRKP01 plasmid unnamed5, complete sequence | *Klebsiella pneumoniae* | 9910 | 10336 | 100% | 0 | 100 | 69455 | 5596 | CP050359.1 |
|  | *Klebsiella pneumoniae* strain KPWX136 plasmid pF, complete sequence | *Klebsiella pneumoniae* | 9878 | 10336 | 100% | 0 | 100 | 69455 | 5596 | CP069175.1 |
|  | *Klebsiella pneumoniae* strain KP20194a2 plasmid pKP20194a2-p5, complete sequence | *Klebsiella pneumoniae* | 9860 | 10330 | 100% | 0 | 99.98 | 69455 | 5596 | CP054779.1 |
|  | *Klebsiella pneumoniae* strain WCHKP8F4 plasmid p2_095084, complete sequence | *Klebsiella pneumoniae* | 9808 | 10336 | 100% | 0 | 100 | 69455 | 5596 | CP027066.3 |
|  | *Klebsiella pneumoniae* strain WCHKP090045 plasmid p2_090045, complete sequence | *Klebsiella pneumoniae* | 9801 | 10336 | 100% | 0 | 100 | 69455 | 5596 | CP043368.1 |
|  | *Klebsiella pneumoniae* strain WCHKP090374 plasmid p2_090374, complete sequence | *Klebsiella pneumoniae* | 9792 | 10330 | 99% | 0 | 100 | 69455 | 5595 | CP066538.1 |
|  | *Klebsiella pneumoniae* strain WCHKP020115 plasmid p2_020115, complete sequence | *Klebsiella pneumoniae* | 9788 | 10336 | 100% | 0 | 100 | 69455 | 5596 | CP043356.1 |
|  | *Klebsiella pneumoniae* isolate 91eed288-b809-11e8-aae5-3c4a9275d6c8 genome assembly, chromosome: 1 | *Klebsiella pneumoniae* | 9716 | 10761 | 100% | 0 | 99.98 | 69455 | 5510473 | LR596808.1 |
|  | *Klebsiella pneumoniae* strain SH12 plasmid pSH12_5, complete sequence | *Klebsiella pneumoniae* | 9716 | 10336 | 100% | 0 | 100 | 69455 | 5596 | CP040838.1 |
|  | *Klebsiella pneumoniae* strain CP19 plasmid unnamed2, complete sequence | *Klebsiella pneumoniae* | 9679 | 10330 | 100% | 0 | 99.98 | 69455 | 5597 | CP073353.1 |
|  | *Klebsiella pneumoniae* strain FK 6768 plasmid unnamed5, complete sequence | *Klebsiella pneumoniae* | 9481 | 10336 | 100% | 0 | 100 | 69455 | 5596 | CP065559.1 |
|  | *Klebsiella pneumoniae* strain hvKP841 plasmid unnamed5, complete sequence | *Klebsiella pneumoniae* | 9465 | 10336 | 100% | 0 | 100 | 69455 | 5596 | CP101789.1 |
|  | *Klebsiella pneumoniae* strain KP20194c3 plasmid pKP20194c3-p5, complete sequence | *Klebsiella pneumoniae* | 9313 | 10330 | 100% | 0 | 99.98 | 69455 | 5596 | CP054755.1 |
|  | *Klebsiella pneumoniae* strain WCHKP020037 plasmid p3_020037, complete sequence | *Klebsiella pneumoniae* | 9274 | 10336 | 100% | 0 | 100 | 69455 | 5596 | CP036375.1 |
|  | *Klebsiella pneumoniae* strain WCHKP3 plasmid p3_020003, complete sequence | *Klebsiella pneumoniae* | 9254 | 10336 | 100% | 0 | 100 | 69455 | 5596 | CP031719.1 |
|  | *Klebsiella pneumoniae* strain WCHKP090357 plasmid p3_090357, complete sequence | *Klebsiella pneumoniae* | 9208 | 10336 | 100% | 0 | 100 | 69455 | 5596 | CP066527.1 |
|  | *Klebsiella pneumoniae* strain 21080937 plasmid p21080937_9, complete sequence | *Klebsiella pneumoniae* | 9143 | 10336 | 100% | 0 | 100 | 69455 | 5596 | CP095262.1 |
|  | *Klebsiella pneumoniae* strain ZRKP03 plasmid unnamed5, complete sequence | *Klebsiella pneumoniae* | 9082 | 10336 | 100% | 0 | 100 | 69455 | 5596 | CP050347.1 |
|  | *Klebsiella pneumoniae* strain XH1508 plasmid pXH1508-5, complete sequence | *Klebsiella pneumoniae* | 9070 | 10336 | 100% | 0 | 100 | 69455 | 5596 | CP092791.1 |
|  | *Klebsiella pneumoniae* strain KP1878 plasmid p1878-6k, complete sequence | *Klebsiella pneumoniae* | 9047 | 10343 | 100% | 0 | 99.98 | 69455 | 6796 | CP073000.1 |
|  | *Klebsiella pneumoniae* strain 16HN-263 plasmid p16HN-263_3, complete sequence | *Klebsiella pneumoniae* | 9010 | 10336 | 100% | 0 | 100 | 69455 | 5596 | CP045266.1 |
|  | *Klebsiella pneumoniae* strain KP-CT77 plasmid unnamed2, complete sequence | *Klebsiella pneumoniae* | 9005 | 10336 | 100% | 0 | 100 | 69455 | 5596 | CP080308.1 |
|  | *Klebsiella pneumoniae* strain S234 plasmid pS234-5, complete sequence | *Klebsiella pneumoniae* | 8981 | 10336 | 100% | 0 | 100 | 69455 | 5596 | CP102191.1 |
|  | *Klebsiella pneumoniae* strain KP51248 plasmid p51248_2, complete sequence | *Klebsiella pneumoniae* | 8981 | 10188 | 99% | 0 | 99.55 | 69455 | 5598 | CP070566.1 |
|  | *Klebsiella pneumoniae* strain hvKP319 plasmid unnamed5, complete sequence | *Klebsiella pneumoniae* | 8966 | 10336 | 100% | 0 | 100 | 69455 | 5596 | CP101769.1 |
|  | *Klebsiella pneumoniae* strain KP20194b2 plasmid pKP20194b2-p5, complete sequence | *Klebsiella pneumoniae* | 8938 | 10330 | 100% | 0 | 99.98 | 69455 | 5596 | CP054767.1 |
|  | *Klebsiella pneumoniae* strain 140253 plasmid p4_140253, complete sequence | *Klebsiella pneumoniae* | 8887 | 10330 | 99% | 0 | 100 | 69455 | 5595 | CP097631.1 |
|  | *Klebsiella pneumoniae* isolate 13 genome assembly, plasmid: P5 | *Klebsiella pneumoniae* | 8857 | 10204 | 99% | 0 | 99.61 | 69455 | 5602 | OW968430.1 |
|  | *Klebsiella pneumoniae* strain CR-HvKP3 plasmid p5-CR-HvKP3, complete sequence | *Klebsiella pneumoniae* | 8761 | 10336 | 100% | 0 | 100 | 69455 | 5596 | MW598235.1 |
|  | *Klebsiella pneumoniae* strain KP20194c plasmid pKP20194c-p5, complete sequence | *Klebsiella pneumoniae* | 8741 | 10330 | 100% | 0 | 100 | 69455 | 5596 | CP054761.1 |
|  | *Klebsiella pneumoniae* strain CRKP66R plasmid pCRKP66R-6, complete sequence | *Klebsiella pneumoniae* | 8741 | 10336 | 100% | 0 | 100 | 69455 | 5596 | CP063838.1 |
|  | *Klebsiella pneumoniae* *subsp.* pneumoniae strain DD01304 plasmid pDD01304-4, complete sequence | *Klebsiella pneumoniae* *subsp.* pneumoniae | 8715 | 10336 | 100% | 0 | 100 | 69455 | 5596 | CP087610.1 |
|  | *Klebsiella pneumoniae* strain 36 plasmid pKP36_6, complete sequence | *Klebsiella pneumoniae* | 8704 | 10336 | 100% | 0 | 100 | 69455 | 5596 | CP082764.1 |
|  | *Klebsiella pneumoniae* strain 21091025 plasmid p21091025_4, complete sequence | *Klebsiella pneumoniae* | 8702 | 10336 | 100% | 0 | 100 | 69455 | 5596 | CP095268.1 |
|  | *Klebsiella pneumoniae* strain FO15 plasmid unnamed2, complete sequence | *Klebsiella pneumoniae* | 8639 | 17396 | 100% | 0 | 100 | 69455 | 9417 | CP073004.1 |
|  | *Klebsiella pneumoniae* strain KP58 plasmid pKP58-5, complete sequence | *Klebsiella pneumoniae* | 8480 | 10336 | 100% | 0 | 100 | 69455 | 5596 | CP041378.1 |
|  | *Klebsiella pneumoniae* strain KP18-2079 plasmid pKP18-2079_5kb, complete sequence | *Klebsiella pneumoniae* | 8436 | 10336 | 100% | 0 | 100 | 69455 | 5596 | MT090963.1 |
|  | *Klebsiella pneumoniae* strain CR-HvKP5 plasmid pCR-HvKP5-p5, complete sequence | *Klebsiella pneumoniae* | 8407 | 10336 | 100% | 0 | 100 | 69455 | 5596 | CP040550.1 |
|  | *Klebsiella pneumoniae* strain F726925 plasmid pF726925-4, complete sequence | *Klebsiella pneumoniae* | 8392 | 10336 | 100% | 0 | 100 | 69455 | 5596 | CP081824.1 |
|  | *Klebsiella pneumoniae* strain CDI694 plasmid pCDI694-5.6, complete sequence | *Klebsiella pneumoniae* | 8381 | 10336 | 100% | 0 | 100 | 69455 | 5596 | CP077774.1 |
|  | *Klebsiella pneumoniae* *subsp.* pneumoniae strain DD02341 plasmid pDD02341-4, complete sequence | *Klebsiella pneumoniae* *subsp.* pneumoniae | 8351 | 10336 | 100% | 0 | 100 | 69455 | 5596 | CP087633.1 |
|  | *Klebsiella pneumoniae* strain XH1507 plasmid pXH1507-6, complete sequence | *Klebsiella pneumoniae* | 8351 | 10336 | 100% | 0 | 100 | 69455 | 5596 | CP092799.1 |
|  | *Klebsiella pneumoniae* strain 21072329 plasmid p21072329_5, complete sequence | *Klebsiella pneumoniae* | 8222 | 10336 | 100% | 0 | 100 | 69455 | 5596 | CP095239.1 |
|  | *Klebsiella pneumoniae* strain KP69 plasmid p69-4, complete sequence | *Klebsiella pneumoniae* | 8216 | 10336 | 100% | 0 | 100 | 69455 | 5596 | CP025460.1 |
|  | *Klebsiella pneumoniae* strain JX-CR-hvKP-9 plasmid pJX9-5, complete sequence | *Klebsiella pneumoniae* | 8202 | 10336 | 100% | 0 | 100 | 69455 | 5596 | CP064216.1 |
|  | *Klebsiella pneumoniae* *subsp.* pneumoniae strain KP65 plasmid p65_3, complete sequence | *Klebsiella pneumoniae* *subsp.* pneumoniae | 8196 | 10330 | 100% | 0 | 99.98 | 69455 | 5596 | CP101566.1 |
|  | *Klebsiella pneumoniae* strain XHKP502 plasmid pXHKP502-5, complete sequence | *Klebsiella pneumoniae* | 8196 | 10330 | 100% | 0 | 99.98 | 69455 | 5596 | CP066909.1 |
|  | *Klebsiella pneumoniae* strain WCHKP2080 plasmid p2_095080, complete sequence | *Klebsiella pneumoniae* | 8187 | 10336 | 100% | 0 | 100 | 69455 | 5596 | CP036364.1 |
|  | *Klebsiella pneumoniae* strain JX-CR-hvKP-7 plasmid pJX7-5, complete sequence | *Klebsiella pneumoniae* | 8179 | 10336 | 100% | 0 | 100 | 69455 | 5596 | CP064228.1 |
|  | *Klebsiella pneumoniae* strain KP18-1 plasmid pKP18-1-3, complete sequence | *Klebsiella pneumoniae* | 8170 | 10336 | 100% | 0 | 100 | 69455 | 5596 | CP082000.1 |
|  | *Klebsiella pneumoniae* strain KP18-41 plasmid pKP18-41-3, complete sequence | *Klebsiella pneumoniae* | 8168 | 10336 | 100% | 0 | 100 | 69455 | 5596 | CP082009.1 |
|  | Citrobacter koseri ATCC BAA-895 plasmid pCKO2, complete sequence | Citrobacter koseri ATCC BAA-895 | 8139 | 10203 | 99% | 0 | 99.73 | 69455 | 5601 | CP000824.1 |
|  | *Klebsiella pneumoniae* strain KPA9853 plasmid pA9853_1, complete sequence | *Klebsiella pneumoniae* | 8106 | 10188 | 99% | 0 | 99.53 | 69455 | 5598 | CP070601.1 |
|  | *Klebsiella pneumoniae* strain KP55 plasmid pKP55_6, complete sequence | *Klebsiella pneumoniae* | 8104 | 10336 | 100% | 0 | 100 | 69455 | 5596 | CP055300.1 |
|  | *Klebsiella pneumoniae* *subsp.* pneumoniae strain DD02391 plasmid pDD02391-5, complete sequence | *Klebsiella pneumoniae* *subsp.* pneumoniae | 8094 | 10336 | 100% | 0 | 100 | 69455 | 5596 | CP087644.1 |
|  | *Klebsiella pneumoniae* *subsp.* pneumoniae strain WCHKP015093 plasmid p3_015093, complete sequence | *Klebsiella pneumoniae* *subsp.* pneumoniae | 8082 | 10336 | 100% | 0 | 100 | 69455 | 5596 | CP036304.1 |
|  | *Klebsiella pneumoniae* strain KP20194c4 plasmid pKP20194c4-p5, complete sequence | *Klebsiella pneumoniae* | 8065 | 10330 | 100% | 0 | 99.98 | 69455 | 5596 | CP054749.1 |
|  | *Klebsiella pneumoniae* *subsp.* pneumoniae strain SCKP020143 plasmid p4_020143, complete sequence | *Klebsiella pneumoniae* *subsp.* pneumoniae | 8052 | 10336 | 100% | 0 | 100 | 69455 | 5596 | CP028546.2 |
|  | *Klebsiella pneumoniae* isolate 392 genome assembly, plasmid: P4 | *Klebsiella pneumoniae* | 8043 | 10304 | 99% | 0 | 99.91 | 69455 | 5598 | OW848890.1 |
|  | *Klebsiella pneumoniae* strain WCHKP2 plasmid p2_020002, complete sequence | *Klebsiella pneumoniae* | 8013 | 10336 | 100% | 0 | 100 | 69455 | 5596 | CP028540.3 |
|  | *Klebsiella pneumoniae* strain BJCFK909 plasmid p4s2, complete sequence | *Klebsiella pneumoniae* | 7956 | 10336 | 100% | 0 | 100 | 69455 | 5596 | CP034127.1 |
|  | *Klebsiella pneumoniae* strain ARLG-4861 plasmid pC592_5, complete sequence | *Klebsiella pneumoniae* | 7801 | 9037 | 99% | 0 | 99.76 | 69455 | 5584 | CP067621.1 |
|  | *Klebsiella pneumoniae* strain L39_2 plasmid p6_L39, complete sequence | *Klebsiella pneumoniae* | 7781 | 10336 | 100% | 0 | 100 | 69455 | 5596 | CP033959.1 |
|  | *Klebsiella pneumoniae* strain F44 plasmid p44-4, complete sequence | *Klebsiella pneumoniae* | 7758 | 10336 | 100% | 0 | 100 | 69455 | 5596 | CP025465.1 |
|  | *Klebsiella pneumoniae* strain 135077 plasmid p3_135077, complete sequence | *Klebsiella pneumoniae* | 7705 | 10336 | 100% | 0 | 100 | 69455 | 5596 | CP073295.1 |
|  | *Klebsiella pneumoniae* strain K191663 plasmid unnamed1, complete sequence | *Klebsiella pneumoniae* | 7677 | 10336 | 100% | 0 | 100 | 69455 | 5596 | CP080354.1 |
|  | *Klebsiella pneumoniae* strain KP55 plasmid pKPC-5505, complete sequence | *Klebsiella pneumoniae* | 7670 | 10336 | 100% | 0 | 100 | 69455 | 5596 | OL891655.1 |
|  | *Klebsiella pneumoniae* isolate 97706988-b809-11e8-aae5-3c4a9275d6c8 genome assembly, chromosome: 1 | *Klebsiella pneumoniae* | 7625 | 11066 | 100% | 0 | 100 | 69455 | 5652578 | LR596813.1 |
|  | *Klebsiella pneumoniae* strain KP-C76 plasmid unnamed1, complete sequence | *Klebsiella pneumoniae* | 7579 | 10336 | 100% | 0 | 100 | 69455 | 5596 | CP080302.1 |
|  | *Klebsiella pneumoniae* strain KP-426 plasmid unnamed2, complete sequence | *Klebsiella pneumoniae* | 7509 | 10336 | 100% | 0 | 100 | 69455 | 5596 | CP080315.1 |
|  | *Klebsiella pneumoniae* strain CR-HvKP4 plasmid pCR-HvKP4-p5, complete sequence | *Klebsiella pneumoniae* | 7492 | 10336 | 100% | 0 | 100 | 69455 | 5596 | CP040544.1 |
|  | *Klebsiella pneumoniae* isolate 392 genome assembly, plasmid: P4 | *Klebsiella pneumoniae* | 7487 | 10304 | 99% | 0 | 99.95 | 69455 | 5599 | OW849056.1 |
|  | *Klebsiella pneumoniae* strain 160111 plasmid p5.6K_L111, complete sequence | *Klebsiella pneumoniae* | 7426 | 10336 | 100% | 0 | 100 | 69455 | 5596 | CP030131.1 |
|  | *Klebsiella pneumoniae* strain KP19-2196 plasmid pKP19-2196-4, complete sequence | *Klebsiella pneumoniae* | 7341 | 10336 | 100% | 0 | 100 | 69455 | 5596 | CP082040.1 |
|  | *Klebsiella pneumoniae* strain KP18-2172 plasmid pKP18-2172-3, complete sequence | *Klebsiella pneumoniae* | 7317 | 10336 | 100% | 0 | 100 | 69455 | 5596 | CP082036.1 |
|  | *Klebsiella pneumoniae* strain ZRKP04 plasmid unnamed5, complete sequence | *Klebsiella pneumoniae* | 7304 | 10336 | 100% | 0 | 100 | 69455 | 5596 | CP050341.1 |
|  | *Klebsiella pneumoniae* strain 49088 plasmid p49088-6.796, complete sequence | *Klebsiella pneumoniae* | 7302 | 10344 | 100% | 0 | 99.97 | 69455 | 6796 | CP088998.1 |
|  | *Klebsiella pneumoniae* strain 37 plasmid pKP37_6, complete sequence | *Klebsiella pneumoniae* | 7286 | 10336 | 100% | 0 | 100 | 69455 | 5596 | CP082758.1 |
|  | *Klebsiella pneumoniae* *subsp.* pneumoniae strain KP21 plasmid p21_3, complete sequence | *Klebsiella pneumoniae* *subsp.* pneumoniae | 7278 | 10336 | 100% | 0 | 100 | 69455 | 5596 | CP101545.1 |
|  | *Klebsiella pneumoniae* *subsp.* pneumoniae strain DD01635 plasmid pDD01635-5, complete sequence | *Klebsiella pneumoniae* *subsp.* pneumoniae | 7254 | 10336 | 100% | 0 | 100 | 69455 | 5596 | CP087662.1 |
|  | *Klebsiella pneumoniae* strain WCHKP090050 plasmid p2_090050, complete sequence | *Klebsiella pneumoniae* | 7177 | 10336 | 100% | 0 | 100 | 69455 | 5596 | CP043372.1 |
|  | *Klebsiella pneumoniae* strain KPN142 plasmid pn142_3, complete sequence | *Klebsiella pneumoniae* | 7167 | 10336 | 100% | 0 | 100 | 69455 | 5596 | CP053878.1 |
|  | *Klebsiella pneumoniae* strain KPC-2 plasmid pKP169-P6, complete sequence | *Klebsiella pneumoniae* | 7118 | 10330 | 100% | 0 | 100 | 69455 | 5596 | CP078128.1 |
|  | *Klebsiella pneumoniae* strain FZKP4523 plasmid p2_FZKP4523, complete sequence | *Klebsiella pneumoniae* | 7107 | 10330 | 99% | 0 | 100 | 69455 | 5595 | CP101536.1 |
|  | *Klebsiella pneumoniae* strain WSCRKP plasmid pWSCRKP-5, complete sequence | *Klebsiella pneumoniae* | 7103 | 10336 | 100% | 0 | 100 | 69455 | 5596 | CP091073.1 |
|  | *Klebsiella pneumoniae* strain WCHKP115011 plasmid p3_115011, complete sequence | *Klebsiella pneumoniae* | 7064 | 10336 | 100% | 0 | 100 | 69455 | 5596 | CP089958.1 |
|  | *Klebsiella pneumoniae* isolate 99060032-b809-11e8-aae5-3c4a9275d6c8 genome assembly, chromosome: 1 | *Klebsiella pneumoniae* | 7059 | 10334 | 99% | 0 | 100 | 69455 | 5479501 | LR596812.1 |
| pCR-hvKP005-P2 | *Klebsiella pneumoniae* strain KP0079 plasmid pKP0079-4, complete sequence |  | 22099 | 44201 | 100% | 0 | 99.99 | 11970 | 23940 | CP102840.1 |
|  | *Klebsiella pneumoniae* strain 36 plasmid pKP36_5, complete sequence | *Klebsiella pneumoniae* | 22083 | 22083 | 99% | 0 | 100 | 11970 | 11970 | CP082763.1 |
|  | *Klebsiella pneumoniae* strain CRKP66R plasmid pCRKP66R-5, complete sequence | *Klebsiella pneumoniae* | 22077 | 22077 | 99% | 0 | 100 | 11970 | 11970 | CP063837.1 |
|  | *Klebsiella pneumoniae* strain KP20194c plasmid pKP20194c-p4, complete sequence | *Klebsiella pneumoniae* | 22075 | 22075 | 99% | 0 | 99.99 | 11970 | 11970 | CP054760.1 |
|  | *Klebsiella pneumoniae* strain KP20194a plasmid pKP20194a-p4, complete sequence | *Klebsiella pneumoniae* | 22074 | 22074 | 99% | 0 | 99.99 | 11970 | 11970 | CP054784.1 |
|  | *Klebsiella pneumoniae* strain C2414 plasmid pC2414-4, complete sequence | *Klebsiella pneumoniae* | 22072 | 22072 | 99% | 0 | 100 | 11970 | 11970 | CP039822.1 |
|  | *Klebsiella pneumoniae* *subsp.* pneumoniae strain DD01635 plasmid pDD01635-4, complete sequence | *Klebsiella pneumoniae* *subsp.* pneumoniae | 22064 | 22064 | 99% | 0 | 100 | 11970 | 11970 | CP087661.1 |
|  | *Klebsiella pneumoniae* strain C2660 plasmid pC2660-5, complete sequence | *Klebsiella pneumoniae* | 22061 | 22061 | 99% | 0 | 99.99 | 11970 | 11970 | CP039812.1 |
|  | *Klebsiella pneumoniae* strain 21080937 plasmid p21080937_6, complete sequence | *Klebsiella pneumoniae* | 22061 | 22061 | 99% | 0 | 100 | 11970 | 11970 | CP095259.1 |
|  | *Klebsiella pneumoniae* *subsp.* pneumoniae strain DD01845 plasmid pDD01845-4, complete sequence | *Klebsiella pneumoniae* *subsp.* pneumoniae | 22053 | 22053 | 99% | 0 | 99.99 | 11970 | 11970 | CP087667.1 |
|  | *Klebsiella pneumoniae* strain 8695 plasmid p4, complete sequence | *Klebsiella pneumoniae* | 22044 | 44121 | 100% | 0 | 99.89 | 11970 | 23957 | CP085893.1 |
|  | *Klebsiella pneumoniae* strain CDI694 plasmid pCDI694-12.0, complete sequence | *Klebsiella pneumoniae* | 21861 | 21861 | 99% | 0 | 99.67 | 11970 | 11972 | CP077775.1 |
|  | *Klebsiella pneumoniae* strain 21091025 plasmid p21091025_3, complete sequence | *Klebsiella pneumoniae* | 21608 | 43319 | 100% | 0 | 99.27 | 11970 | 23759 | CP095267.1 |
|  | *Escherichia coli* strain CR-HvKP5TC-5 plasmid pCR-HvKP5TC-5_p4, complete sequence | *Escherichia coli* | 20753 | 22106 | 100% | 0 | 100 | 11970 | 11970 | OM001479.1 |
|  | *Klebsiella pneumoniae* strain KPWX136 plasmid pE, complete sequence | *Klebsiella pneumoniae* | 20751 | 22101 | 100% | 0 | 99.99 | 11970 | 11970 | CP069174.1 |
|  | *Klebsiella pneumoniae* strain Kp36 plasmid unnamed4, complete sequence | *Klebsiella pneumoniae* | 19908 | 22074 | 100% | 0 | 99.86 | 11970 | 12001 | CP047196.1 |
|  | *Klebsiella pneumoniae* strain KP20194f plasmid pKP20194f-p4, complete sequence | *Klebsiella pneumoniae* | 19645 | 22101 | 100% | 0 | 99.99 | 11970 | 11970 | CP054724.1 |
|  | *Klebsiella pneumoniae* *subsp.* pneumoniae strain DD01653 plasmid pDD01653-4, complete sequence | *Klebsiella pneumoniae* *subsp.* pneumoniae | 19642 | 22095 | 100% | 0 | 99.98 | 11970 | 11970 | CP087655.1 |
|  | *Klebsiella pneumoniae* strain KP58 plasmid pKP58-4, complete sequence | *Klebsiella pneumoniae* | 19396 | 22101 | 100% | 0 | 99.99 | 11970 | 11970 | CP041377.1 |
|  | *Klebsiella pneumoniae* *subsp.* pneumoniae strain DD02391 plasmid pDD02391-4, complete sequence | *Klebsiella pneumoniae* *subsp.* pneumoniae | 19014 | 21724 | 100% | 0 | 99.36 | 11970 | 11959 | CP087643.1 |
|  | *Klebsiella pneumoniae* strain XHKP502 plasmid pXHKP502-4, complete sequence | *Klebsiella pneumoniae* | 18766 | 22095 | 100% | 0 | 99.98 | 11970 | 11971 | CP066908.1 |
|  | *Klebsiella pneumoniae* strain WCHKP090361 plasmid p2_090361, complete sequence | *Klebsiella pneumoniae* | 18349 | 22106 | 100% | 0 | 100 | 11970 | 11970 | CP066532.1 |
|  | *Klebsiella pneumoniae* strain KP18-2079 plasmid pKP18-2079_11kb, complete sequence | *Klebsiella pneumoniae* | 18329 | 22101 | 100% | 0 | 99.99 | 11970 | 11970 | MT090962.1 |
|  | *Klebsiella pneumoniae* strain 9949 plasmid unnamed4, complete sequence | *Klebsiella pneumoniae* | 18144 | 29934 | 100% | 0 | 99.97 | 11970 | 16223 | CP050284.1 |
|  | *Klebsiella pneumoniae* strain 37 plasmid pKP37_5, complete sequence | *Klebsiella pneumoniae* | 18066 | 22093 | 100% | 0 | 99.98 | 11970 | 11969 | CP082757.1 |
|  | *Klebsiella pneumoniae* strain FK 6768 plasmid unnamed4, complete sequence | *Klebsiella pneumoniae* | 17516 | 21957 | 100% | 0 | 99.68 | 11970 | 12001 | CP065558.1 |
|  | *Klebsiella pneumoniae* strain KP1064WHY plasmid p4, complete sequence | *Klebsiella pneumoniae* | 17256 | 22008 | 100% | 0 | 99.82 | 11970 | 11972 | CP084709.1 |
|  | *Escherichia coli* strain CR-HvKP3TC plasmid pCR-HvKP3TC_p4, complete sequence | *Escherichia coli* | 16982 | 22106 | 100% | 0 | 100 | 11970 | 11970 | MW598243.1 |
|  | *Klebsiella pneumoniae* strain CR-HvKP3 plasmid p4-CR-HvKP3, complete sequence | *Klebsiella pneumoniae* | 16982 | 22106 | 100% | 0 | 100 | 11970 | 11970 | MW598236.1 |
|  | *Klebsiella pneumoniae* strain KP697 plasmid unnamed4, complete sequence | *Klebsiella pneumoniae* | 16973 | 22101 | 100% | 0 | 99.99 | 11970 | 11970 | CP066155.1 |
|  | *Klebsiella pneumoniae* strain KP18-2113 plasmid pKP18-2113-3, complete sequence | *Klebsiella pneumoniae* | 16957 | 22088 | 100% | 0 | 99.97 | 11970 | 11969 | CP082028.1 |
|  | *Klebsiella pneumoniae* strain KP20194c4 plasmid pKP20194c4-p4, complete sequence | *Klebsiella pneumoniae* | 16842 | 21979 | 100% | 0 | 99.75 | 11970 | 11971 | CP054748.1 |
|  | *Klebsiella pneumoniae* strain KP20194b2 plasmid pKP20194b2-p4, complete sequence | *Klebsiella pneumoniae* | 16809 | 22101 | 100% | 0 | 99.99 | 11970 | 11970 | CP054766.1 |
|  | *Klebsiella pneumoniae* strain KP200731214 plasmid pKP-5 | *Klebsiella pneumoniae* | 16720 | 22106 | 100% | 0 | 100 | 11970 | 11970 | CP084748.1 |
|  | *Klebsiella pneumoniae* strain KP18-238 plasmid pKP18-238-3, complete sequence | *Klebsiella pneumoniae* | 16687 | 21724 | 100% | 0 | 99.28 | 11970 | 11959 | CP082013.1 |
|  | *Klebsiella pneumoniae* strain 12 plasmid pKP12_5, complete sequence | *Klebsiella pneumoniae* | 15653 | 22084 | 100% | 0 | 100 | 11970 | 11970 | CP082769.1 |
|  | *Escherichia coli* strain CR-HvKP4TC plasmid pCR-HvKP4TC_Vir-p4, complete sequence | *Escherichia coli* | 15034 | 25688 | 100% | 0 | 100 | 11970 | 190130 | MW598245.1 |
|  | *Escherichia coli* strain CR-HvKP4TC-2 plasmid pCR-HvKP4TC-2_p4, complete sequence | *Escherichia coli* | 14994 | 22106 | 100% | 0 | 100 | 11970 | 11970 | OM001477.1 |
|  | *Klebsiella pneumoniae* strain KP1517 plasmid unnamed3, complete sequence | *Klebsiella pneumoniae* | 14922 | 22106 | 100% | 0 | 100 | 11970 | 11970 | CP072466.1 |
|  | *Klebsiella pneumoniae* strain LZKP00003 plasmid pZR1, complete sequence | *Klebsiella pneumoniae* | 14698 | 22106 | 100% | 0 | 100 | 11970 | 11970 | CP089992.1 |
|  | *Klebsiella pneumoniae* strain FDAARGOS_443 plasmid unnamed4, complete sequence | *Klebsiella pneumoniae* | 14683 | 22097 | 100% | 0 | 99.97 | 11970 | 11972 | CP023932.1 |
|  | *Klebsiella pneumoniae* strain JX-CR-hvKP-3 plasmid pJX3-4, complete sequence | *Klebsiella pneumoniae* | 14534 | 22106 | 100% | 0 | 100 | 11970 | 11970 | CP064245.1 |
|  | *Klebsiella pneumoniae* strain KP20194a2 plasmid pKP20194a2-p4, complete sequence | *Klebsiella pneumoniae* | 14314 | 22046 | 100% | 0 | 99.83 | 11970 | 15931 | CP054778.1 |
|  | *Klebsiella pneumoniae* strain KP55 plasmid pKP55_5, complete sequence | *Klebsiella pneumoniae* | 14299 | 22106 | 100% | 0 | 100 | 11970 | 11970 | CP055299.1 |
|  | *Klebsiella pneumoniae* strain L482 plasmid p5_L382, complete sequence | *Klebsiella pneumoniae* | 14296 | 22106 | 100% | 0 | 100 | 11970 | 11970 | CP033964.1 |
|  | *Klebsiella pneumoniae* strain SCKP020009 plasmid p1_020009, complete sequence | *Klebsiella pneumoniae* | 14283 | 22106 | 100% | 0 | 100 | 11970 | 11970 | CP038005.1 |
|  | *Klebsiella pneumoniae* strain L39_2 plasmid p5_L39, complete sequence | *Klebsiella pneumoniae* | 14277 | 22101 | 100% | 0 | 99.99 | 11970 | 11970 | CP033958.1 |
|  | *Klebsiella pneumoniae* strain KP16 plasmid unnamed4, complete sequence | *Klebsiella pneumoniae* | 14277 | 22101 | 100% | 0 | 99.99 | 11970 | 11970 | CP087150.1 |
|  | *Klebsiella pneumoniae* strain KP14 plasmid unnamed4, complete sequence | *Klebsiella pneumoniae* | 14277 | 22101 | 100% | 0 | 99.99 | 11970 | 11970 | CP087155.1 |
|  | *Klebsiella pneumoniae* strain WSCRKP plasmid pWSCRKP-4, complete sequence | *Klebsiella pneumoniae* | 14235 | 22115 | 100% | 0 | 99.99 | 11970 | 15931 | CP091072.1 |
|  | *Klebsiella pneumoniae* strain KP15 plasmid unnamed3, complete sequence | *Klebsiella pneumoniae* | 14044 | 25657 | 100% | 0 | 99.97 | 11970 | 99065 | CP087145.1 |
|  | *Klebsiella pneumoniae* strain hvKP323 plasmid unnamed3, complete sequence | *Klebsiella pneumoniae* | 14039 | 22106 | 100% | 0 | 100 | 11970 | 11970 | CP101773.1 |
|  | *Klebsiella pneumoniae* strain XH1507 plasmid pXH1507-5, complete sequence | *Klebsiella pneumoniae* | 14039 | 22106 | 100% | 0 | 100 | 11970 | 11970 | CP092798.1 |
|  | *Klebsiella pneumoniae* strain CRKP52R plasmid pCRKP52R-4-tetA, complete sequence | *Klebsiella pneumoniae* | 14028 | 25641 | 100% | 0 | 99.93 | 11970 | 99066 | CP066252.1 |
|  | *Escherichia coli* strain CR-HvKP5TC plasmid pCR-HvKP5TC_Vir-p4, complete sequence | *Escherichia coli* | 14026 | 25646 | 100% | 0 | 99.93 | 11970 | 190129 | MW598247.1 |
|  | *Escherichia coli* strain CR-HvKP1TC plasmid pCR-HvKP1TC_Vir-p4, complete sequence | *Escherichia coli* | 14026 | 25646 | 100% | 0 | 99.93 | 11970 | 190129 | MW598240.1 |
|  | *Klebsiella pneumoniae* strain BSI058 plasmid pBSI058-KPC2 | *Klebsiella pneumoniae* | 14024 | 25644 | 100% | 0 | 99.91 | 11970 | 114712 | MT269836.1 |
|  | *Klebsiella pneumoniae* strain 1864 plasmid p1864-4, complete sequence | *Klebsiella pneumoniae* | 14020 | 22106 | 100% | 0 | 100 | 11970 | 11970 | CP084496.1 |
|  | *Klebsiella pneumoniae* strain KP-CT77 plasmid pCT77-tetA, complete sequence | *Klebsiella pneumoniae* | 14007 | 25620 | 100% | 0 | 99.88 | 11970 | 92645 | CP080306.1 |
|  | *Klebsiella pneumoniae* strain WCHKP115068 plasmid p2_115068, complete sequence | *Klebsiella pneumoniae* | 13825 | 22121 | 100% | 0 | 100 | 11970 | 12746 | CP036369.1 |
|  | *Klebsiella pneumoniae* strain WCHKP020098 plasmid p2_020098, complete sequence | *Klebsiella pneumoniae* | 13444 | 22106 | 100% | 0 | 100 | 11970 | 11970 | CP036308.1 |
|  | *Klebsiella pneumoniae* strain JX-CR-hvKP-1 plasmid pJX1-4, complete sequence | *Klebsiella pneumoniae* | 13345 | 22106 | 100% | 0 | 100 | 11970 | 11970 | CP064256.1 |
|  | *Klebsiella pneumoniae* JM45 plasmid p2, complete sequence | *Klebsiella pneumoniae* JM45 | 13274 | 16372 | 80% | 0 | 97.22 | 11970 | 12207 | CP006658.1 |
|  | *Klebsiella pneumoniae* isolate 307 genome assembly, plasmid: P3 | *Klebsiella pneumoniae* | 12912 | 19526 | 80% | 0 | 97.31 | 11970 | 34959 | OW967853.1 |
|  | *Klebsiella pneumoniae* isolate 307 genome assembly, plasmid: P3 | *Klebsiella pneumoniae* | 12912 | 19526 | 80% | 0 | 97.31 | 11970 | 34959 | OW967206.1 |
|  | *Klebsiella pneumoniae* isolate 307 genome assembly, plasmid: P3 | *Klebsiella pneumoniae* | 12912 | 19526 | 80% | 0 | 97.31 | 11970 | 34959 | OW849062.1 |
|  | *Klebsiella pneumoniae* strain RIVM_C019006 plasmid pRIVM_C019006_3, complete sequence | *Klebsiella pneumoniae* | 12912 | 19522 | 80% | 0 | 97.3 | 11970 | 34959 | CP068923.1 |
|  | *Klebsiella pneumoniae* strain WCHKP090329 plasmid p2_090329, complete sequence | *Klebsiella pneumoniae* | 12912 | 22106 | 100% | 0 | 100 | 11970 | 11970 | CP066521.1 |
|  | *Klebsiella pneumoniae* strain DD521 plasmid pDD521.4, complete sequence | *Klebsiella pneumoniae* | 12896 | 22088 | 100% | 0 | 99.97 | 11970 | 11969 | CP075320.1 |
|  | *Klebsiella pneumoniae* strain IR12243_1 plasmid unnamed1, complete sequence | *Klebsiella pneumoniae* | 12700 | 22124 | 100% | 0 | 99.99 | 11970 | 12798 | CP097661.1 |
|  | *Klebsiella pneumoniae* strain K64 plasmid pColRNAI-5, complete sequence | *Klebsiella pneumoniae* | 12626 | 20693 | 99% | 0 | 96.8 | 11970 | 11934 | CP102395.1 |
|  | *Klebsiella pneumoniae* strain S234 plasmid pS234-3, complete sequence | *Klebsiella pneumoniae* | 12183 | 22101 | 100% | 0 | 99.98 | 11970 | 11970 | CP102189.1 |
|  | *Klebsiella pneumoniae* strain L491 plasmid p4-L491 | *Klebsiella pneumoniae* | 11875 | 22106 | 100% | 0 | 100 | 11970 | 11970 | CP029229.1 |
|  | *Klebsiella pneumoniae* strain L388 plasmid p5-L388 | *Klebsiella pneumoniae* | 11869 | 22101 | 100% | 0 | 99.98 | 11970 | 11970 | CP029224.1 |
|  | *Klebsiella pneumoniae* *subsp.* pneumoniae strain KPN857 plasmid pD, complete sequence | *Klebsiella pneumoniae* *subsp.* pneumoniae | 11854 | 22101 | 100% | 0 | 99.98 | 11970 | 11970 | CP090436.1 |
|  | *Klebsiella pneumoniae* strain JX-CR-hvKP-4 plasmid pJX4-4, complete sequence | *Klebsiella pneumoniae* | 11701 | 22106 | 100% | 0 | 100 | 11970 | 11970 | CP064239.1 |
|  | *Escherichia coli* strain CR-HvKP1TC-3 plasmid pCR-HvKP1TC-3_p4, complete sequence | *Escherichia coli* | 11620 | 22106 | 100% | 0 | 100 | 11970 | 11970 | OM001473.1 |
|  | *Klebsiella pneumoniae* strain WCHKP36 plasmid p2_020036, complete sequence | *Klebsiella pneumoniae* | 11616 | 22122 | 100% | 0 | 100 | 11970 | 12746 | CP028580.2 |
|  | *Klebsiella pneumoniae* strain hvKP340 plasmid unnamed5, complete sequence | *Klebsiella pneumoniae* | 11616 | 22106 | 100% | 0 | 100 | 11970 | 11970 | CP101781.1 |
|  | *Klebsiella pneumoniae* strain hvKP841 plasmid unnamed3, complete sequence | *Klebsiella pneumoniae* | 11616 | 22106 | 100% | 0 | 100 | 11970 | 11970 | CP101787.1 |
|  | *Klebsiella pneumoniae* strain 19PDR22 plasmid p7, complete sequence | *Klebsiella pneumoniae* | 11616 | 22106 | 100% | 0 | 100 | 11970 | 11970 | CP076551.1 |
|  | *Klebsiella pneumoniae* strain S270v plasmid pS270V-3, complete sequence | *Klebsiella pneumoniae* | 11611 | 22101 | 100% | 0 | 99.98 | 11970 | 11970 | CP102195.1 |
|  | *Klebsiella pneumoniae* strain hvKP319 plasmid unnamed4, complete sequence | *Klebsiella pneumoniae* | 11611 | 22101 | 100% | 0 | 99.98 | 11970 | 11970 | CP101768.1 |
|  | *Klebsiella pneumoniae* strain FRPDR plasmid pFRPDR_5, complete sequence | *Klebsiella pneumoniae* | 11611 | 22101 | 100% | 0 | 99.98 | 11970 | 11970 | CP063764.1 |
|  | *Klebsiella pneumoniae* strain CR-HvKP4 plasmid pCR-HvKP4-p4, complete sequence | *Klebsiella pneumoniae* | 11548 | 22106 | 100% | 0 | 100 | 11970 | 11970 | CP040543.1 |
|  | *Klebsiella pneumoniae* strain CR-HvKP5 plasmid pCR-HvKP5-p4, complete sequence | *Klebsiella pneumoniae* | 11527 | 22106 | 100% | 0 | 100 | 11970 | 11970 | CP040549.1 |
|  | *Klebsiella pneumoniae* strain CR-HvKP1 plasmid pCR-HvKP1-p4, complete sequence | *Klebsiella pneumoniae* | 11514 | 22106 | 100% | 0 | 100 | 11970 | 11970 | CP040537.1 |
|  | *Klebsiella pneumoniae* strain 50700 plasmid p50700-12.0, complete sequence | *Klebsiella pneumoniae* | 11420 | 21920 | 100% | 0 | 99.46 | 11970 | 11972 | CP088993.1 |
|  | *Klebsiella pneumoniae* isolate 991178e0-b809-11e8-aae5-3c4a9275d6c8 genome assembly, chromosome: 1 | *Klebsiella pneumoniae* | 10707 | 24599 | 98% | 0 | 100 | 11970 | 5570095 | LR596809.1 |
|  | *Klebsiella pneumoniae* isolate 98fb0f42-b809-11e8-aae5-3c4a9275d6c8 genome assembly, chromosome: 1 | *Klebsiella pneumoniae* | 10707 | 25085 | 93% | 0 | 100 | 11970 | 5716474 | LR596807.1 |
|  | *Klebsiella pneumoniae* *subsp.* pneumoniae strain RJBSI76 plasmid pRJBSI76-4, complete sequence | *Klebsiella pneumoniae* *subsp.* pneumoniae | 10623 | 18843 | 85% | 0 | 99.98 | 11970 | 10206 | CP068693.1 |
|  | *Klebsiella pneumoniae* isolate 15 genome assembly, plasmid: P2 | *Klebsiella pneumoniae* | 10495 | 15990 | 80% | 0 | 96.82 | 11970 | 11955 | OW969695.1 |
|  | *Klebsiella pneumoniae* strain 150040X1B1 plasmid p1_150040X1B1, complete sequence | *Klebsiella pneumoniae* | 10490 | 18580 | 84% | 0 | 100 | 11970 | 10060 | CP101729.1 |
|  | *Klebsiella pneumoniae* *subsp.* pneumoniae strain KP29 plasmid p29_3, complete sequence | *Klebsiella pneumoniae* *subsp.* pneumoniae | 10490 | 18580 | 84% | 0 | 100 | 11970 | 10060 | CP101570.1 |
|  | *Klebsiella pneumoniae* strain FZKP4523 plasmid p1_FZKP4523, complete sequence | *Klebsiella pneumoniae* | 10490 | 18580 | 84% | 0 | 100 | 11970 | 10060 | CP101535.1 |
|  | *Klebsiella pneumoniae* strain XHKP75 plasmid pXHKP75-3, complete sequence | *Klebsiella pneumoniae* | 10490 | 18580 | 84% | 0 | 100 | 11970 | 10060 | CP066898.1 |
|  | *Klebsiella pneumoniae* strain XHKPN391 plasmid pXHKPN391-2, complete sequence | *Klebsiella pneumoniae* | 10490 | 18579 | 84% | 0 | 100 | 11970 | 10060 | CP066917.1 |
|  | *Klebsiella pneumoniae* strain HvKp-su1 plasmid unnamed2, complete sequence | *Klebsiella pneumoniae* | 10490 | 18582 | 84% | 0 | 100 | 11970 | 10061 | CP092719.1 |
|  | *Klebsiella pneumoniae* *subsp.* pneumoniae strain DD02280 plasmid pDD02280-3 | *Klebsiella pneumoniae* *subsp.* pneumoniae | 10490 | 18568 | 83% | 0 | 100 | 11970 | 91826 | CP087626.1 |
|  | *Klebsiella pneumoniae* *subsp.* pneumoniae strain DD02172 plasmid pDD02172-5, complete sequence | *Klebsiella pneumoniae* *subsp.* pneumoniae | 10490 | 18580 | 84% | 0 | 100 | 11970 | 10060 | CP087616.1 |
| pCR-hvKP005-P3 | *Klebsiella pneumoniae* strain KP18-238 plasmid pKP18-238-4, complete sequence | *Klebsiella pneumoniae* | 10334 | 20671 | 100% | 0 | 100 | 5596 | 11192 | CP082017.1 |
|  | *Klebsiella pneumoniae* strain KP20194c5 plasmid pKP20194c5-p5, complete sequence | *Klebsiella pneumoniae* | 10325 | 10325 | 99% | 0 | 99.98 | 5596 | 5596 | CP054743.1 |
|  | *Klebsiella pneumoniae* strain 150040X1B1 plasmid p2_150040X1B1, complete sequence | *Klebsiella pneumoniae* | 10296 | 10296 | 99% | 0 | 100 | 5596 | 5596 | CP101730.1 |
|  | *Klebsiella pneumoniae* *subsp.* pneumoniae strain DD02280 plasmid pDD02280-4, complete sequence | *Klebsiella pneumoniae* *subsp.* pneumoniae | 10257 | 10336 | 100% | 0 | 100 | 5596 | 5596 | CP087627.1 |
|  | *Klebsiella pneumoniae* strain RIVM_C014947 plasmid pRIVM_C014947_4 | *Klebsiella pneumoniae* | 10168 | 10325 | 100% | 0 | 99.96 | 5596 | 5596 | MT560067.1 |
|  | *Klebsiella pneumoniae* *subsp.* pneumoniae strain SCKP020079 plasmid p2_020079, complete sequence | *Klebsiella pneumoniae* *subsp.* pneumoniae | 10126 | 10336 | 100% | 0 | 100 | 5596 | 5596 | CP029380.1 |
|  | *Klebsiella pneumoniae* strain WCHKP040035 plasmid p2_040035, complete sequence | *Klebsiella pneumoniae* | 10124 | 10336 | 100% | 0 | 100 | 5596 | 5596 | CP028795.2 |
|  | *Klebsiella pneumoniae* isolate 392 genome assembly, plasmid: P4 | *Klebsiella pneumoniae* | 10109 | 10304 | 99% | 0 | 99.91 | 5596 | 5599 | OW848883.1 |
|  | *Klebsiella pneumoniae* isolate 392 genome assembly, plasmid: P3 | *Klebsiella pneumoniae* | 10109 | 10304 | 99% | 0 | 99.91 | 5596 | 5599 | OW849089.1 |
|  | *Klebsiella pneumoniae* strain KP20194a plasmid pKP20194a-p5, complete sequence | *Klebsiella pneumoniae* | 10098 | 10330 | 100% | 0 | 99.98 | 5596 | 5596 | CP054785.1 |
|  | *Klebsiella pneumoniae* *subsp.* pneumoniae strain WCHKP020120 plasmid p3_020120, complete sequence | *Klebsiella pneumoniae* *subsp.* pneumoniae | 10067 | 10336 | 100% | 0 | 100 | 5596 | 5596 | CP043361.1 |
|  | *Klebsiella pneumoniae* strain JX-CR-hvKP-10 plasmid pJX10-4, complete sequence | *Klebsiella pneumoniae* | 10046 | 10336 | 100% | 0 | 100 | 5596 | 5596 | CP064262.1 |
|  | *Klebsiella pneumoniae* isolate 392 genome assembly, plasmid: P4 | *Klebsiella pneumoniae* | 9963 | 10304 | 99% | 0 | 99.91 | 5596 | 5599 | OW849049.1 |
|  | *Klebsiella pneumoniae* strain ZRKP01 plasmid unnamed5, complete sequence | *Klebsiella pneumoniae* | 9910 | 10336 | 100% | 0 | 100 | 5596 | 5596 | CP050359.1 |
|  | *Klebsiella pneumoniae* strain KPWX136 plasmid pF, complete sequence | *Klebsiella pneumoniae* | 9878 | 10336 | 100% | 0 | 100 | 5596 | 5596 | CP069175.1 |
|  | *Klebsiella pneumoniae* strain KP20194a2 plasmid pKP20194a2-p5, complete sequence | *Klebsiella pneumoniae* | 9860 | 10330 | 100% | 0 | 99.98 | 5596 | 5596 | CP054779.1 |
|  | *Klebsiella pneumoniae* strain WCHKP8F4 plasmid p2_095084, complete sequence | *Klebsiella pneumoniae* | 9808 | 10336 | 100% | 0 | 100 | 5596 | 5596 | CP027066.3 |
|  | *Klebsiella pneumoniae* strain WCHKP090045 plasmid p2_090045, complete sequence | *Klebsiella pneumoniae* | 9801 | 10336 | 100% | 0 | 100 | 5596 | 5596 | CP043368.1 |
|  | *Klebsiella pneumoniae* strain WCHKP090374 plasmid p2_090374, complete sequence | *Klebsiella pneumoniae* | 9792 | 10330 | 99% | 0 | 100 | 5596 | 5595 | CP066538.1 |
|  | *Klebsiella pneumoniae* strain WCHKP020115 plasmid p2_020115, complete sequence | *Klebsiella pneumoniae* | 9788 | 10336 | 100% | 0 | 100 | 5596 | 5596 | CP043356.1 |
|  | *Klebsiella pneumoniae* isolate 91eed288-b809-11e8-aae5-3c4a9275d6c8 genome assembly, chromosome: 1 | *Klebsiella pneumoniae* | 9716 | 10761 | 100% | 0 | 99.98 | 5596 | 5510473 | LR596808.1 |
|  | *Klebsiella pneumoniae* strain SH12 plasmid pSH12_5, complete sequence | *Klebsiella pneumoniae* | 9716 | 10336 | 100% | 0 | 100 | 5596 | 5596 | CP040838.1 |
|  | *Klebsiella pneumoniae* strain CP19 plasmid unnamed2, complete sequence | *Klebsiella pneumoniae* | 9679 | 10330 | 100% | 0 | 99.98 | 5596 | 5597 | CP073353.1 |
|  | *Klebsiella pneumoniae* strain FK 6768 plasmid unnamed5, complete sequence | *Klebsiella pneumoniae* | 9481 | 10336 | 100% | 0 | 100 | 5596 | 5596 | CP065559.1 |
|  | *Klebsiella pneumoniae* strain hvKP841 plasmid unnamed5, complete sequence | *Klebsiella pneumoniae* | 9465 | 10336 | 100% | 0 | 100 | 5596 | 5596 | CP101789.1 |
|  | *Klebsiella pneumoniae* strain KP20194c3 plasmid pKP20194c3-p5, complete sequence | *Klebsiella pneumoniae* | 9313 | 10330 | 100% | 0 | 99.98 | 5596 | 5596 | CP054755.1 |
|  | *Klebsiella pneumoniae* strain WCHKP020037 plasmid p3_020037, complete sequence | *Klebsiella pneumoniae* | 9274 | 10336 | 100% | 0 | 100 | 5596 | 5596 | CP036375.1 |
|  | *Klebsiella pneumoniae* strain WCHKP3 plasmid p3_020003, complete sequence | *Klebsiella pneumoniae* | 9254 | 10336 | 100% | 0 | 100 | 5596 | 5596 | CP031719.1 |
|  | *Klebsiella pneumoniae* strain WCHKP090357 plasmid p3_090357, complete sequence | *Klebsiella pneumoniae* | 9208 | 10336 | 100% | 0 | 100 | 5596 | 5596 | CP066527.1 |
|  | *Klebsiella pneumoniae* strain 21080937 plasmid p21080937_9, complete sequence | *Klebsiella pneumoniae* | 9143 | 10336 | 100% | 0 | 100 | 5596 | 5596 | CP095262.1 |
|  | *Klebsiella pneumoniae* strain ZRKP03 plasmid unnamed5, complete sequence | *Klebsiella pneumoniae* | 9082 | 10336 | 100% | 0 | 100 | 5596 | 5596 | CP050347.1 |
|  | *Klebsiella pneumoniae* strain XH1508 plasmid pXH1508-5, complete sequence | *Klebsiella pneumoniae* | 9070 | 10336 | 100% | 0 | 100 | 5596 | 5596 | CP092791.1 |
|  | *Klebsiella pneumoniae* strain KP1878 plasmid p1878-6k, complete sequence | *Klebsiella pneumoniae* | 9047 | 10343 | 100% | 0 | 99.98 | 5596 | 6796 | CP073000.1 |
|  | *Klebsiella pneumoniae* strain 16HN-263 plasmid p16HN-263_3, complete sequence | *Klebsiella pneumoniae* | 9010 | 10336 | 100% | 0 | 100 | 5596 | 5596 | CP045266.1 |
|  | *Klebsiella pneumoniae* strain KP-CT77 plasmid unnamed2, complete sequence | *Klebsiella pneumoniae* | 9005 | 10336 | 100% | 0 | 100 | 5596 | 5596 | CP080308.1 |
|  | *Klebsiella pneumoniae* strain S234 plasmid pS234-5, complete sequence | *Klebsiella pneumoniae* | 8981 | 10336 | 100% | 0 | 100 | 5596 | 5596 | CP102191.1 |
|  | *Klebsiella pneumoniae* strain KP51248 plasmid p51248_2, complete sequence | *Klebsiella pneumoniae* | 8981 | 10188 | 99% | 0 | 99.55 | 5596 | 5598 | CP070566.1 |
|  | *Klebsiella pneumoniae* strain hvKP319 plasmid unnamed5, complete sequence | *Klebsiella pneumoniae* | 8966 | 10336 | 100% | 0 | 100 | 5596 | 5596 | CP101769.1 |
|  | *Klebsiella pneumoniae* strain KP20194b2 plasmid pKP20194b2-p5, complete sequence | *Klebsiella pneumoniae* | 8938 | 10330 | 100% | 0 | 99.98 | 5596 | 5596 | CP054767.1 |
|  | *Klebsiella pneumoniae* strain 140253 plasmid p4_140253, complete sequence | *Klebsiella pneumoniae* | 8887 | 10330 | 99% | 0 | 100 | 5596 | 5595 | CP097631.1 |
|  | *Klebsiella pneumoniae* isolate 13 genome assembly, plasmid: P5 | *Klebsiella pneumoniae* | 8857 | 10204 | 99% | 0 | 99.61 | 5596 | 5602 | OW968430.1 |
|  | *Klebsiella pneumoniae* strain CR-HvKP3 plasmid p5-CR-HvKP3, complete sequence | *Klebsiella pneumoniae* | 8761 | 10336 | 100% | 0 | 100 | 5596 | 5596 | MW598235.1 |
|  | *Klebsiella pneumoniae* strain KP20194c plasmid pKP20194c-p5, complete sequence | *Klebsiella pneumoniae* | 8741 | 10330 | 100% | 0 | 100 | 5596 | 5596 | CP054761.1 |
|  | *Klebsiella pneumoniae* strain CRKP66R plasmid pCRKP66R-6, complete sequence | *Klebsiella pneumoniae* | 8741 | 10336 | 100% | 0 | 100 | 5596 | 5596 | CP063838.1 |
|  | *Klebsiella pneumoniae* *subsp.* pneumoniae strain DD01304 plasmid pDD01304-4, complete sequence | *Klebsiella pneumoniae* *subsp.* pneumoniae | 8715 | 10336 | 100% | 0 | 100 | 5596 | 5596 | CP087610.1 |
|  | *Klebsiella pneumoniae* strain 36 plasmid pKP36_6, complete sequence | *Klebsiella pneumoniae* | 8704 | 10336 | 100% | 0 | 100 | 5596 | 5596 | CP082764.1 |
|  | *Klebsiella pneumoniae* strain 21091025 plasmid p21091025_4, complete sequence | *Klebsiella pneumoniae* | 8702 | 10336 | 100% | 0 | 100 | 5596 | 5596 | CP095268.1 |
|  | *Klebsiella pneumoniae* strain FO15 plasmid unnamed2, complete sequence | *Klebsiella pneumoniae* | 8639 | 17396 | 100% | 0 | 100 | 5596 | 9417 | CP073004.1 |
|  | *Klebsiella pneumoniae* strain KP58 plasmid pKP58-5, complete sequence | *Klebsiella pneumoniae* | 8480 | 10336 | 100% | 0 | 100 | 5596 | 5596 | CP041378.1 |
|  | *Klebsiella pneumoniae* strain KP18-2079 plasmid pKP18-2079_5kb, complete sequence | *Klebsiella pneumoniae* | 8436 | 10336 | 100% | 0 | 100 | 5596 | 5596 | MT090963.1 |
|  | *Klebsiella pneumoniae* strain CR-HvKP5 plasmid pCR-HvKP5-p5, complete sequence | *Klebsiella pneumoniae* | 8407 | 10336 | 100% | 0 | 100 | 5596 | 5596 | CP040550.1 |
|  | *Klebsiella pneumoniae* strain F726925 plasmid pF726925-4, complete sequence | *Klebsiella pneumoniae* | 8392 | 10336 | 100% | 0 | 100 | 5596 | 5596 | CP081824.1 |
|  | *Klebsiella pneumoniae* strain CDI694 plasmid pCDI694-5.6, complete sequence | *Klebsiella pneumoniae* | 8381 | 10336 | 100% | 0 | 100 | 5596 | 5596 | CP077774.1 |
|  | *Klebsiella pneumoniae* *subsp.* pneumoniae strain DD02341 plasmid pDD02341-4, complete sequence | *Klebsiella pneumoniae* *subsp.* pneumoniae | 8351 | 10336 | 100% | 0 | 100 | 5596 | 5596 | CP087633.1 |
|  | *Klebsiella pneumoniae* strain XH1507 plasmid pXH1507-6, complete sequence | *Klebsiella pneumoniae* | 8351 | 10336 | 100% | 0 | 100 | 5596 | 5596 | CP092799.1 |
|  | *Klebsiella pneumoniae* strain 21072329 plasmid p21072329_5, complete sequence | *Klebsiella pneumoniae* | 8222 | 10336 | 100% | 0 | 100 | 5596 | 5596 | CP095239.1 |
|  | *Klebsiella pneumoniae* strain KP69 plasmid p69-4, complete sequence | *Klebsiella pneumoniae* | 8216 | 10336 | 100% | 0 | 100 | 5596 | 5596 | CP025460.1 |
|  | *Klebsiella pneumoniae* strain JX-CR-hvKP-9 plasmid pJX9-5, complete sequence | *Klebsiella pneumoniae* | 8202 | 10336 | 100% | 0 | 100 | 5596 | 5596 | CP064216.1 |
|  | *Klebsiella pneumoniae* *subsp.* pneumoniae strain KP65 plasmid p65_3, complete sequence | *Klebsiella pneumoniae* *subsp.* pneumoniae | 8196 | 10330 | 100% | 0 | 99.98 | 5596 | 5596 | CP101566.1 |
|  | *Klebsiella pneumoniae* strain XHKP502 plasmid pXHKP502-5, complete sequence | *Klebsiella pneumoniae* | 8196 | 10330 | 100% | 0 | 99.98 | 5596 | 5596 | CP066909.1 |
|  | *Klebsiella pneumoniae* strain WCHKP2080 plasmid p2_095080, complete sequence | *Klebsiella pneumoniae* | 8187 | 10336 | 100% | 0 | 100 | 5596 | 5596 | CP036364.1 |
|  | *Klebsiella pneumoniae* strain JX-CR-hvKP-7 plasmid pJX7-5, complete sequence | *Klebsiella pneumoniae* | 8179 | 10336 | 100% | 0 | 100 | 5596 | 5596 | CP064228.1 |
|  | *Klebsiella pneumoniae* strain KP18-1 plasmid pKP18-1-3, complete sequence | *Klebsiella pneumoniae* | 8170 | 10336 | 100% | 0 | 100 | 5596 | 5596 | CP082000.1 |
|  | *Klebsiella pneumoniae* strain KP18-41 plasmid pKP18-41-3, complete sequence | *Klebsiella pneumoniae* | 8168 | 10336 | 100% | 0 | 100 | 5596 | 5596 | CP082009.1 |
|  | Citrobacter koseri ATCC BAA-895 plasmid pCKO2, complete sequence | Citrobacter koseri ATCC BAA-895 | 8139 | 10203 | 99% | 0 | 99.73 | 5596 | 5601 | CP000824.1 |
|  | *Klebsiella pneumoniae* strain KPA9853 plasmid pA9853_1, complete sequence | *Klebsiella pneumoniae* | 8106 | 10188 | 99% | 0 | 99.53 | 5596 | 5598 | CP070601.1 |
|  | *Klebsiella pneumoniae* strain KP55 plasmid pKP55_6, complete sequence | *Klebsiella pneumoniae* | 8104 | 10336 | 100% | 0 | 100 | 5596 | 5596 | CP055300.1 |
|  | *Klebsiella pneumoniae* *subsp.* pneumoniae strain DD02391 plasmid pDD02391-5, complete sequence | *Klebsiella pneumoniae* *subsp.* pneumoniae | 8094 | 10336 | 100% | 0 | 100 | 5596 | 5596 | CP087644.1 |
|  | *Klebsiella pneumoniae* *subsp.* pneumoniae strain WCHKP015093 plasmid p3_015093, complete sequence | *Klebsiella pneumoniae* *subsp.* pneumoniae | 8082 | 10336 | 100% | 0 | 100 | 5596 | 5596 | CP036304.1 |
|  | *Klebsiella pneumoniae* strain KP20194c4 plasmid pKP20194c4-p5, complete sequence | *Klebsiella pneumoniae* | 8065 | 10330 | 100% | 0 | 99.98 | 5596 | 5596 | CP054749.1 |
|  | *Klebsiella pneumoniae* *subsp.* pneumoniae strain SCKP020143 plasmid p4_020143, complete sequence | *Klebsiella pneumoniae* *subsp.* pneumoniae | 8052 | 10336 | 100% | 0 | 100 | 5596 | 5596 | CP028546.2 |
|  | *Klebsiella pneumoniae* isolate 392 genome assembly, plasmid: P4 | *Klebsiella pneumoniae* | 8043 | 10304 | 99% | 0 | 99.91 | 5596 | 5598 | OW848890.1 |
|  | *Klebsiella pneumoniae* strain WCHKP2 plasmid p2_020002, complete sequence | *Klebsiella pneumoniae* | 8013 | 10336 | 100% | 0 | 100 | 5596 | 5596 | CP028540.3 |
|  | *Klebsiella pneumoniae* strain BJCFK909 plasmid p4s2, complete sequence | *Klebsiella pneumoniae* | 7956 | 10336 | 100% | 0 | 100 | 5596 | 5596 | CP034127.1 |
|  | *Klebsiella pneumoniae* strain ARLG-4861 plasmid pC592_5, complete sequence | *Klebsiella pneumoniae* | 7801 | 9037 | 99% | 0 | 99.76 | 5596 | 5584 | CP067621.1 |
|  | *Klebsiella pneumoniae* strain L39_2 plasmid p6_L39, complete sequence | *Klebsiella pneumoniae* | 7781 | 10336 | 100% | 0 | 100 | 5596 | 5596 | CP033959.1 |
|  | *Klebsiella pneumoniae* strain F44 plasmid p44-4, complete sequence | *Klebsiella pneumoniae* | 7758 | 10336 | 100% | 0 | 100 | 5596 | 5596 | CP025465.1 |
|  | *Klebsiella pneumoniae* strain 135077 plasmid p3_135077, complete sequence | *Klebsiella pneumoniae* | 7705 | 10336 | 100% | 0 | 100 | 5596 | 5596 | CP073295.1 |
|  | *Klebsiella pneumoniae* strain K191663 plasmid unnamed1, complete sequence | *Klebsiella pneumoniae* | 7677 | 10336 | 100% | 0 | 100 | 5596 | 5596 | CP080354.1 |
|  | *Klebsiella pneumoniae* strain KP55 plasmid pKPC-5505, complete sequence | *Klebsiella pneumoniae* | 7670 | 10336 | 100% | 0 | 100 | 5596 | 5596 | OL891655.1 |
|  | *Klebsiella pneumoniae* isolate 97706988-b809-11e8-aae5-3c4a9275d6c8 genome assembly, chromosome: 1 | *Klebsiella pneumoniae* | 7625 | 11066 | 100% | 0 | 100 | 5596 | 5652578 | LR596813.1 |
|  | *Klebsiella pneumoniae* strain KP-C76 plasmid unnamed1, complete sequence | *Klebsiella pneumoniae* | 7579 | 10336 | 100% | 0 | 100 | 5596 | 5596 | CP080302.1 |
|  | *Klebsiella pneumoniae* strain KP-426 plasmid unnamed2, complete sequence | *Klebsiella pneumoniae* | 7509 | 10336 | 100% | 0 | 100 | 5596 | 5596 | CP080315.1 |
|  | *Klebsiella pneumoniae* strain CR-HvKP4 plasmid pCR-HvKP4-p5, complete sequence | *Klebsiella pneumoniae* | 7492 | 10336 | 100% | 0 | 100 | 5596 | 5596 | CP040544.1 |
|  | *Klebsiella pneumoniae* isolate 392 genome assembly, plasmid: P4 | *Klebsiella pneumoniae* | 7487 | 10304 | 99% | 0 | 99.95 | 5596 | 5599 | OW849056.1 |
|  | *Klebsiella pneumoniae* strain 160111 plasmid p5.6K_L111, complete sequence | *Klebsiella pneumoniae* | 7426 | 10336 | 100% | 0 | 100 | 5596 | 5596 | CP030131.1 |
|  | *Klebsiella pneumoniae* strain KP19-2196 plasmid pKP19-2196-4, complete sequence | *Klebsiella pneumoniae* | 7341 | 10336 | 100% | 0 | 100 | 5596 | 5596 | CP082040.1 |
|  | *Klebsiella pneumoniae* strain KP18-2172 plasmid pKP18-2172-3, complete sequence | *Klebsiella pneumoniae* | 7317 | 10336 | 100% | 0 | 100 | 5596 | 5596 | CP082036.1 |
|  | *Klebsiella pneumoniae* strain ZRKP04 plasmid unnamed5, complete sequence | *Klebsiella pneumoniae* | 7304 | 10336 | 100% | 0 | 100 | 5596 | 5596 | CP050341.1 |
|  | *Klebsiella pneumoniae* strain 49088 plasmid p49088-6.796, complete sequence | *Klebsiella pneumoniae* | 7302 | 10344 | 100% | 0 | 99.97 | 5596 | 6796 | CP088998.1 |
|  | *Klebsiella pneumoniae* strain 37 plasmid pKP37_6, complete sequence | *Klebsiella pneumoniae* | 7286 | 10336 | 100% | 0 | 100 | 5596 | 5596 | CP082758.1 |
|  | *Klebsiella pneumoniae* *subsp.* pneumoniae strain KP21 plasmid p21_3, complete sequence | *Klebsiella pneumoniae* *subsp.* pneumoniae | 7278 | 10336 | 100% | 0 | 100 | 5596 | 5596 | CP101545.1 |
|  | *Klebsiella pneumoniae* *subsp.* pneumoniae strain DD01635 plasmid pDD01635-5, complete sequence | *Klebsiella pneumoniae* *subsp.* pneumoniae | 7254 | 10336 | 100% | 0 | 100 | 5596 | 5596 | CP087662.1 |
|  | *Klebsiella pneumoniae* strain WCHKP090050 plasmid p2_090050, complete sequence | *Klebsiella pneumoniae* | 7177 | 10336 | 100% | 0 | 100 | 5596 | 5596 | CP043372.1 |
|  | *Klebsiella pneumoniae* strain KPN142 plasmid pn142_3, complete sequence | *Klebsiella pneumoniae* | 7167 | 10336 | 100% | 0 | 100 | 5596 | 5596 | CP053878.1 |
|  | *Klebsiella pneumoniae* strain KPC-2 plasmid pKP169-P6, complete sequence | *Klebsiella pneumoniae* | 7118 | 10330 | 100% | 0 | 100 | 5596 | 5596 | CP078128.1 |
|  | *Klebsiella pneumoniae* strain FZKP4523 plasmid p2_FZKP4523, complete sequence | *Klebsiella pneumoniae* | 7107 | 10330 | 99% | 0 | 100 | 5596 | 5595 | CP101536.1 |
|  | *Klebsiella pneumoniae* strain WSCRKP plasmid pWSCRKP-5, complete sequence | *Klebsiella pneumoniae* | 7103 | 10336 | 100% | 0 | 100 | 5596 | 5596 | CP091073.1 |
|  | *Klebsiella pneumoniae* strain WCHKP115011 plasmid p3_115011, complete sequence | *Klebsiella pneumoniae* | 7064 | 10336 | 100% | 0 | 100 | 5596 | 5596 | CP089958.1 |
|  | *Klebsiella pneumoniae* isolate 99060032-b809-11e8-aae5-3c4a9275d6c8 genome assembly, chromosome: 1 | *Klebsiella pneumoniae* | 7059 | 10334 | 99% | 0 | 100 | 5596 | 5479501 | LR596812.1 |
| pCR-hvKP006-KPC-P1 | *Klebsiella pneumoniae* strain WCHKP090050 plasmid p1_090050, complete sequence | *Klebsiella pneumoniae* | 18399 | 18579 | 100% | 0 | 100 | 111731 | 10060 | CP043371.1 |
|  | *Klebsiella pneumoniae* strain LSH-KPN148 plasmid pLSH-KPN148-3, complete sequence | *Klebsiella pneumoniae* | 18205 | 18579 | 100% | 0 | 100 | 111731 | 10060 | CP040125.1 |
|  | *Klebsiella pneumoniae* strain KP18-2050 plasmid pKP18-2050-3, complete sequence | *Klebsiella pneumoniae* | 18000 | 18197 | 100% | 0 | 99.33 | 111731 | 10049 | CP082022.1 |
|  | *Klebsiella pneumoniae* *subsp.* pneumoniae strain DD02162 plasmid pDD02162-4, complete sequence | *Klebsiella pneumoniae* *subsp.* pneumoniae | 17932 | 18579 | 100% | 0 | 100 | 111731 | 10060 | CP087622.1 |
|  | *Klebsiella pneumoniae* strain FDAARGOS_444 plasmid unnamed3, complete sequence | *Klebsiella pneumoniae* | 17921 | 18573 | 100% | 0 | 99.99 | 111731 | 10061 | CP023944.1 |
|  | *Klebsiella pneumoniae* strain WCHKP115011 plasmid p2_115011, complete sequence | *Klebsiella pneumoniae* | 17821 | 18579 | 100% | 0 | 100 | 111731 | 10060 | CP089957.1 |
|  | *Klebsiella pneumoniae* strain SW1780 plasmid pB, complete sequence | *Klebsiella pneumoniae* | 17821 | 18579 | 100% | 0 | 100 | 111731 | 10060 | CP073304.1 |
|  | *Klebsiella pneumoniae* strain WCHKP7E2 plasmid p1_085072, complete sequence | *Klebsiella pneumoniae* | 17501 | 18579 | 100% | 0 | 100 | 111731 | 10060 | CP028801.2 |
|  | *Klebsiella pneumoniae* strain KP167 plasmid pKP167-10, complete sequence | *Klebsiella pneumoniae* | 17472 | 18579 | 100% | 0 | 100 | 111731 | 10060 | CP098761.1 |
|  | *Klebsiella pneumoniae* strain 135077 plasmid p2_135077, complete sequence | *Klebsiella pneumoniae* | 17472 | 18579 | 100% | 0 | 100 | 111731 | 10060 | CP073294.1 |
|  | *Klebsiella pneumoniae* strain KPN142 plasmid pn142_2, complete sequence | *Klebsiella pneumoniae* | 17398 | 18579 | 100% | 0 | 100 | 111731 | 10060 | CP053877.1 |
|  | *Klebsiella pneumoniae* strain XHKP6 plasmid pXHKP6-2, complete sequence | *Klebsiella pneumoniae* | 17248 | 18614 | 100% | 0 | 99.99 | 111731 | 10098 | CP066889.1 |
|  | *Klebsiella pneumoniae* strain KP19-2029 plasmid pKP19-2029-Col, complete sequence | *Klebsiella pneumoniae* | 17224 | 18579 | 100% | 0 | 100 | 111731 | 10060 | CP047162.1 |
|  | *Klebsiella pneumoniae* strain ZRKP03 plasmid unnamed4, complete sequence | *Klebsiella pneumoniae* | 17163 | 18546 | 100% | 0 | 99.94 | 111731 | 10060 | CP050346.1 |
|  | *Klebsiella pneumoniae* strain WCHKP090045 plasmid p1_090045, complete sequence | *Klebsiella pneumoniae* | 17047 | 18579 | 100% | 0 | 100 | 111731 | 10060 | CP043367.1 |
|  | *Klebsiella pneumoniae* strain KP4863 plasmid pKP4863-2, complete sequence |  | 17016 | 34033 | 100% | 0 | 97.21 | 111731 | 20110 | CP102843.1 |
|  | *Klebsiella pneumoniae* GSU10-3 plasmid pGSU10-3-4 DNA, complete genome | *Klebsiella pneumoniae* | 16750 | 18579 | 100% | 0 | 100 | 111731 | 10060 | AP018675.1 |
|  | *Klebsiella pneumoniae* *subsp.* pneumoniae strain kpn-hnqyy plasmid unnamed3, complete sequence | *Klebsiella pneumoniae* *subsp.* pneumoniae | 16560 | 18579 | 100% | 0 | 100 | 111731 | 10060 | CP074119.1 |
|  | *Klebsiella pneumoniae* strain 21080237 plasmid p21080237_6, complete sequence | *Klebsiella pneumoniae* | 16541 | 18573 | 100% | 0 | 99.99 | 111731 | 10060 | CP095246.1 |
|  | *Klebsiella pneumoniae* strain BSI073 plasmid pBSI073-KPC2 | *Klebsiella pneumoniae* | 16366 | 18594 | 100% | 0 | 100 | 111731 | 118923 | MT269846.1 |
|  | *Klebsiella pneumoniae* strain KP18-1 plasmid pKP18-1-2, complete sequence | *Klebsiella pneumoniae* | 16070 | 18245 | 99% | 0 | 99.82 | 111731 | 10049 | CP082002.1 |
|  | *Klebsiella pneumoniae* strain XHKP53 plasmid pXHKP53-2, complete sequence | *Klebsiella pneumoniae* | 16026 | 18064 | 100% | 0 | 99.35 | 111731 | 10013 | CP066893.1 |
|  | *Klebsiella pneumoniae* isolate 91eed288-b809-11e8-aae5-3c4a9275d6c8 genome assembly, chromosome: 1 | *Klebsiella pneumoniae* | 15993 | 19203 | 100% | 0 | 100 | 111731 | 5510473 | LR596808.1 |
|  | *Klebsiella pneumoniae* strain 21072329 plasmid p21072329_4, complete sequence | *Klebsiella pneumoniae* | 15911 | 18579 | 100% | 0 | 100 | 111731 | 10060 | CP095238.1 |
|  | *Klebsiella pneumoniae* strain WCHKP8F4 plasmid p1_095084, complete sequence | *Klebsiella pneumoniae* | 15645 | 18579 | 100% | 0 | 100 | 111731 | 10060 | CP027065.3 |
|  | *Klebsiella pneumoniae* strain 140253 plasmid p3_140253, complete sequence | *Klebsiella pneumoniae* | 15640 | 18579 | 100% | 0 | 100 | 111731 | 10060 | CP097630.1 |
|  | *Klebsiella pneumoniae* *subsp.* pneumoniae strain SCKP020143 plasmid p3_020143, complete sequence | *Klebsiella pneumoniae* *subsp.* pneumoniae | 15603 | 18579 | 100% | 0 | 100 | 111731 | 10060 | CP028545.2 |
|  | *Klebsiella pneumoniae* strain 1632 plasmid p1632-3, complete sequence | *Klebsiella pneumoniae* | 15570 | 18579 | 100% | 0 | 100 | 111731 | 10060 | CP084500.1 |
|  | *Klebsiella pneumoniae* plasmid unnamed, complete sequence | *Klebsiella pneumoniae* | 15477 | 18579 | 100% | 0 | 100 | 111731 | 10060 | MK181634.1 |
|  | *Klebsiella pneumoniae* *subsp.* pneumoniae strain WCHKP020120 plasmid p2_020120, complete sequence | *Klebsiella pneumoniae* *subsp.* pneumoniae | 15418 | 18579 | 100% | 0 | 100 | 111731 | 10060 | CP043360.1 |
|  | Serratia marcescens Sm4 plasmid pSm4Col DNA, complete sequence | Serratia marcescens | 15407 | 18579 | 100% | 0 | 100 | 111731 | 10060 | LC543864.1 |
|  | *Klebsiella pneumoniae* strain WCHKP649 plasmid p1_095649, complete sequence | *Klebsiella pneumoniae* | 15271 | 18579 | 100% | 0 | 100 | 111731 | 10060 | CP026582.3 |
|  | *Klebsiella pneumoniae* strain 160111 plasmid p10K_L111, complete sequence | *Klebsiella pneumoniae* | 15139 | 18579 | 100% | 0 | 100 | 111731 | 10060 | CP030130.1 |
|  | *Klebsiella pneumoniae* *subsp.* pneumoniae strain DD01754 plasmid pDD01754-4, complete sequence | *Klebsiella pneumoniae* *subsp.* pneumoniae | 15080 | 18540 | 100% | 0 | 100 | 111731 | 10061 | CP087649.1 |
|  | *Klebsiella pneumoniae* strain LSH-KPN25 plasmid pLSH-KPN25-2, complete sequence | *Klebsiella pneumoniae* | 15066 | 18579 | 100% | 0 | 100 | 111731 | 10060 | CP040181.1 |
|  | *Klebsiella pneumoniae* strain XHKPN391 plasmid pXHKPN391-2, complete sequence | *Klebsiella pneumoniae* | 14951 | 18579 | 100% | 0 | 100 | 111731 | 10060 | CP066917.1 |
|  | *Klebsiella pneumoniae* strain JX-CR-hvKP-2 plasmid pJX2-4, complete sequence | *Klebsiella pneumoniae* | 14931 | 18579 | 100% | 0 | 100 | 111731 | 10060 | CP064250.1 |
|  | *Klebsiella pneumoniae* *subsp.* pneumoniae strain KP21 plasmid p21_2, complete sequence | *Klebsiella pneumoniae* *subsp.* pneumoniae | 14925 | 18573 | 100% | 0 | 99.99 | 111731 | 10061 | CP101544.1 |
|  | *Klebsiella pneumoniae* strain KPCZA02 plasmid pKPCZA02_1, complete sequence | *Klebsiella pneumoniae* | 14272 | 18577 | 99% | 0 | 100 | 111731 | 10059 | CP058227.1 |
|  | *Klebsiella pneumoniae* strain KP18-2073 plasmid pKP18-2073-3, complete sequence | *Klebsiella pneumoniae* | 14209 | 18197 | 100% | 0 | 100 | 111731 | 10049 | CP082027.1 |
|  | *Klebsiella pneumoniae* *subsp.* pneumoniae strain WCHKP015093 plasmid p2_015093, complete sequence | *Klebsiella pneumoniae* *subsp.* pneumoniae | 14113 | 18579 | 100% | 0 | 100 | 111731 | 10060 | CP036303.1 |
|  | *Klebsiella pneumoniae* strain HvKp-su1 plasmid unnamed2, complete sequence | *Klebsiella pneumoniae* | 14087 | 18581 | 100% | 0 | 100 | 111731 | 10061 | CP092719.1 |
|  | *Klebsiella pneumoniae* strain GD21SC417 plasmid pHNGS471-4, complete sequence | *Klebsiella pneumoniae* | 14079 | 18579 | 100% | 0 | 100 | 111731 | 10060 | CP089513.1 |
|  | *Klebsiella pneumoniae* strain KPN23 plasmid pKPN23_2, complete sequence | *Klebsiella pneumoniae* | 14078 | 18579 | 100% | 0 | 100 | 111731 | 10060 | CP089864.1 |
|  | *Klebsiella pneumoniae* strain ZRKP04 plasmid unnamed4, complete sequence | *Klebsiella pneumoniae* | 13884 | 18191 | 100% | 0 | 99.12 | 111731 | 10049 | CP050340.1 |
|  | *Klebsiella pneumoniae* strain KP55 plasmid pKPC-5504, complete sequence | *Klebsiella pneumoniae* | 13780 | 18572 | 100% | 0 | 99.99 | 111731 | 10059 | OL891654.1 |
|  | *Klebsiella pneumoniae* *subsp.* pneumoniae strain WCHKP020039 plasmid p1_020039, complete sequence | *Klebsiella pneumoniae* *subsp.* pneumoniae | 13605 | 18579 | 100% | 0 | 100 | 111731 | 10060 | CP043346.1 |
|  | *Klebsiella pneumoniae* strain KP69 plasmid p69-3, complete sequence | *Klebsiella pneumoniae* | 13598 | 18579 | 100% | 0 | 100 | 111731 | 10060 | CP025459.1 |
|  | *Klebsiella pneumoniae* strain ZRKP02 plasmid unnamed4, complete sequence | *Klebsiella pneumoniae* | 13411 | 18579 | 100% | 0 | 100 | 111731 | 10060 | CP050352.1 |
|  | *Klebsiella pneumoniae* strain WCHKP020030 plasmid p1_020030, complete sequence | *Klebsiella pneumoniae* | 13092 | 18579 | 100% | 0 | 100 | 111731 | 10060 | CP028788.2 |
|  | *Klebsiella pneumoniae* strain KP2509 plasmid pKP2509-5, complete sequence | *Klebsiella pneumoniae* | 13027 | 18579 | 100% | 0 | 100 | 111731 | 10060 | CP065951.1 |
|  | *Klebsiella pneumoniae* *subsp.* pneumoniae strain DD02172 plasmid pDD02172-5, complete sequence | *Klebsiella pneumoniae* *subsp.* pneumoniae | 12802 | 18579 | 100% | 0 | 100 | 111731 | 10060 | CP087616.1 |
|  | *Klebsiella pneumoniae* strain C789 plasmid unnamed1, complete sequence | *Klebsiella pneumoniae* | 12589 | 18573 | 100% | 0 | 100 | 111731 | 10061 | CP034418.1 |
|  | *Klebsiella pneumoniae* strain WCHKP3 plasmid p2_020003, complete sequence | *Klebsiella pneumoniae* | 12484 | 18579 | 100% | 0 | 100 | 111731 | 10060 | CP031718.1 |
|  | *Klebsiella pneumoniae* strain WCHKP115069 plasmid p2_115069, complete sequence | *Klebsiella pneumoniae* | 12438 | 18579 | 100% | 0 | 100 | 111731 | 10060 | CP033403.1 |
|  | *Klebsiella pneumoniae* strain KP46 plasmid pKP46_5, complete sequence | *Klebsiella pneumoniae* | 12412 | 18579 | 100% | 0 | 100 | 111731 | 10060 | CP090131.1 |
|  | *Klebsiella pneumoniae* strain KP18-8 plasmid pKP18-8-4, complete sequence | *Klebsiella pneumoniae* | 12227 | 18573 | 100% | 0 | 99.98 | 111731 | 10060 | CP082008.1 |
|  | *Klebsiella pneumoniae* strain WCHKP2 plasmid p1_020002, complete sequence | *Klebsiella pneumoniae* | 12028 | 18579 | 100% | 0 | 100 | 111731 | 10060 | CP028539.3 |
|  | *Klebsiella pneumoniae* strain SH12 plasmid pSH12_3, complete sequence | *Klebsiella pneumoniae* | 11810 | 18579 | 100% | 0 | 100 | 111731 | 10060 | CP040836.1 |
|  | *Klebsiella pneumoniae* strain JX-CR-hvKP-7 plasmid pJX7-4, complete sequence | *Klebsiella pneumoniae* | 11801 | 18579 | 100% | 0 | 100 | 111731 | 10060 | CP064227.1 |
|  | *Klebsiella pneumoniae* strain FO15 plasmid unnamed4, complete sequence | *Klebsiella pneumoniae* | 11795 | 27510 | 100% | 0 | 99.98 | 111731 | 14945 | CP073006.1 |
|  | *Klebsiella pneumoniae* strain 16HN-263 plasmid p16HN-263_2, complete sequence | *Klebsiella pneumoniae* | 11771 | 18579 | 100% | 0 | 100 | 111731 | 10060 | CP045265.1 |
|  | *Klebsiella pneumoniae* strain KP20194c4 plasmid pKP20194c4-p4, complete sequence | *Klebsiella pneumoniae* | 11716 | 18458 | 100% | 0 | 100 | 111731 | 11971 | CP054748.1 |
|  | *Klebsiella pneumoniae* strain KP18-2079 plasmid pKP18-2079_11kb, complete sequence | *Klebsiella pneumoniae* | 11716 | 18580 | 100% | 0 | 99.98 | 111731 | 11970 | MT090962.1 |
|  | *Klebsiella pneumoniae* strain WCHKP36 plasmid p2_020036, complete sequence | *Klebsiella pneumoniae* | 11716 | 18595 | 100% | 0 | 100 | 111731 | 12746 | CP028580.2 |
|  | *Klebsiella pneumoniae* strain L482 plasmid p5_L382, complete sequence | *Klebsiella pneumoniae* | 11716 | 18580 | 100% | 0 | 100 | 111731 | 11970 | CP033964.1 |
|  | *Klebsiella pneumoniae* isolate 97706988-b809-11e8-aae5-3c4a9275d6c8 genome assembly, chromosome: 1 | *Klebsiella pneumoniae* | 11716 | 19114 | 100% | 0 | 100 | 111731 | 5652578 | LR596813.1 |
|  | *Klebsiella pneumoniae* strain KP0079 plasmid pKP0079-4, complete sequence |  | 11716 | 37160 | 100% | 0 | 99.98 | 111731 | 23940 | CP102840.1 |
|  | *Klebsiella pneumoniae* strain S270v plasmid pS270V-3, complete sequence | *Klebsiella pneumoniae* | 11716 | 18579 | 100% | 0 | 100 | 111731 | 11970 | CP102195.1 |
|  | *Klebsiella pneumoniae* strain S234 plasmid pS234-3, complete sequence | *Klebsiella pneumoniae* | 11716 | 18580 | 100% | 0 | 99.98 | 111731 | 11970 | CP102189.1 |
|  | *Klebsiella pneumoniae* strain hvKP340 plasmid unnamed5, complete sequence | *Klebsiella pneumoniae* | 11716 | 18579 | 100% | 0 | 100 | 111731 | 11970 | CP101781.1 |
|  | *Klebsiella pneumoniae* strain hvKP841 plasmid unnamed3, complete sequence | *Klebsiella pneumoniae* | 11716 | 18579 | 100% | 0 | 100 | 111731 | 11970 | CP101787.1 |
|  | *Klebsiella pneumoniae* strain hvKP319 plasmid unnamed4, complete sequence | *Klebsiella pneumoniae* | 11716 | 18579 | 100% | 0 | 100 | 111731 | 11970 | CP101768.1 |
|  | *Klebsiella pneumoniae* strain KP15 plasmid unnamed3, complete sequence | *Klebsiella pneumoniae* | 11716 | 18579 | 100% | 0 | 100 | 111731 | 99065 | CP087145.1 |
|  | *Escherichia coli* strain CR-HvKP1TC-3 plasmid pCR-HvKP1TC-3_p4, complete sequence | *Escherichia coli* | 11716 | 18575 | 99% | 0 | 100 | 111731 | 11970 | OM001473.1 |
|  | *Klebsiella pneumoniae* strain WSCRKP plasmid pWSCRKP-4, complete sequence | *Klebsiella pneumoniae* | 11716 | 18594 | 100% | 0 | 100 | 111731 | 15931 | CP091072.1 |
|  | *Klebsiella pneumoniae* strain KP697 plasmid unnamed4, complete sequence | *Klebsiella pneumoniae* | 11716 | 18580 | 100% | 0 | 100 | 111731 | 11970 | CP066155.1 |
|  | *Klebsiella pneumoniae* *subsp.* pneumoniae strain KPN857 plasmid pD, complete sequence | *Klebsiella pneumoniae* *subsp.* pneumoniae | 11716 | 18580 | 100% | 0 | 99.98 | 111731 | 11970 | CP090436.1 |
|  | *Klebsiella pneumoniae* strain LZKP00003 plasmid pZR1, complete sequence | *Klebsiella pneumoniae* | 11716 | 18580 | 100% | 0 | 100 | 111731 | 11970 | CP089992.1 |
|  | *Klebsiella pneumoniae* strain 50700 plasmid p50700-12.0, complete sequence | *Klebsiella pneumoniae* | 11716 | 18393 | 100% | 0 | 100 | 111731 | 11972 | CP088993.1 |
|  | *Klebsiella pneumoniae* strain 8695 plasmid p4, complete sequence | *Klebsiella pneumoniae* | 11716 | 37104 | 100% | 0 | 100 | 111731 | 23957 | CP085893.1 |
|  | *Klebsiella pneumoniae* strain 12 plasmid pKP12_5, complete sequence | *Klebsiella pneumoniae* | 11716 | 18558 | 100% | 0 | 99.98 | 111731 | 11970 | CP082769.1 |
|  | *Klebsiella pneumoniae* strain KP18-2113 plasmid pKP18-2113-3, complete sequence | *Klebsiella pneumoniae* | 11716 | 18580 | 100% | 0 | 100 | 111731 | 11969 | CP082028.1 |
|  | *Klebsiella pneumoniae* strain KP-CT77 plasmid pCT77-tetA, complete sequence | *Klebsiella pneumoniae* | 11716 | 18537 | 100% | 0 | 99.98 | 111731 | 92645 | CP080306.1 |
|  | *Klebsiella pneumoniae* strain 19PDR22 plasmid p7, complete sequence | *Klebsiella pneumoniae* | 11716 | 18579 | 100% | 0 | 100 | 111731 | 11970 | CP076551.1 |
|  | *Klebsiella pneumoniae* strain DD521 plasmid pDD521.4, complete sequence | *Klebsiella pneumoniae* | 11716 | 18579 | 100% | 0 | 99.98 | 111731 | 11969 | CP075320.1 |
|  | *Escherichia coli* strain CR-HvKP5TC plasmid pCR-HvKP5TC_Vir-p4, complete sequence | *Escherichia coli* | 11716 | 18579 | 100% | 0 | 100 | 111731 | 190129 | MW598247.1 |
|  | *Escherichia coli* strain CR-HvKP3TC plasmid pCR-HvKP3TC_p4, complete sequence | *Escherichia coli* | 11716 | 18580 | 100% | 0 | 100 | 111731 | 11970 | MW598243.1 |
|  | *Escherichia coli* strain CR-HvKP1TC plasmid pCR-HvKP1TC_Vir-p4, complete sequence | *Escherichia coli* | 11716 | 18579 | 100% | 0 | 100 | 111731 | 190129 | MW598240.1 |
|  | *Klebsiella pneumoniae* strain CR-HvKP3 plasmid p4-CR-HvKP3, complete sequence | *Klebsiella pneumoniae* | 11716 | 18580 | 100% | 0 | 100 | 111731 | 11970 | MW598236.1 |
|  | *Klebsiella pneumoniae* strain KP55 plasmid pKP55_5, complete sequence | *Klebsiella pneumoniae* | 11716 | 18580 | 100% | 0 | 100 | 111731 | 11970 | CP055299.1 |
|  | *Klebsiella pneumoniae* strain BSI058 plasmid pBSI058-KPC2 | *Klebsiella pneumoniae* | 11716 | 18579 | 100% | 0 | 100 | 111731 | 114712 | MT269836.1 |
|  | *Klebsiella pneumoniae* strain 015625 plasmid p2_015625, complete sequence | *Klebsiella pneumoniae* | 11716 | 17042 | 91% | 0 | 99.98 | 111731 | 11904 | CP033392.2 |
|  | *Klebsiella pneumoniae* *subsp.* pneumoniae strain RJBSI76 plasmid pRJBSI76-4, complete sequence | *Klebsiella pneumoniae* *subsp.* pneumoniae | 11716 | 18573 | 100% | 0 | 99.98 | 111731 | 10206 | CP068693.1 |
|  | *Klebsiella pneumoniae* strain WCHKP090361 plasmid p2_090361, complete sequence | *Klebsiella pneumoniae* | 11716 | 18580 | 100% | 0 | 99.98 | 111731 | 11970 | CP066532.1 |
|  | *Klebsiella pneumoniae* strain WCHKP090329 plasmid p2_090329, complete sequence | *Klebsiella pneumoniae* | 11716 | 18579 | 100% | 0 | 99.98 | 111731 | 11970 | CP066521.1 |
|  | *Klebsiella pneumoniae* strain CRKP52R plasmid pCRKP52R-4-tetA, complete sequence | *Klebsiella pneumoniae* | 11716 | 18557 | 100% | 0 | 99.98 | 111731 | 99066 | CP066252.1 |
|  | *Klebsiella pneumoniae* strain JX-CR-hvKP-1 plasmid pJX1-4, complete sequence | *Klebsiella pneumoniae* | 11716 | 18579 | 100% | 0 | 100 | 111731 | 11970 | CP064256.1 |
|  | *Klebsiella pneumoniae* strain JX-CR-hvKP-4 plasmid pJX4-4, complete sequence | *Klebsiella pneumoniae* | 11716 | 18580 | 100% | 0 | 100 | 111731 | 11970 | CP064239.1 |
|  | *Klebsiella pneumoniae* strain FRPDR plasmid pFRPDR_5, complete sequence | *Klebsiella pneumoniae* | 11716 | 18579 | 100% | 0 | 100 | 111731 | 11970 | CP063764.1 |
| pCR-hvKP006-P2 | *Klebsiella pneumoniae* strain IR12197_1 plasmid unnamed3 | *Klebsiella pneumoniae* | 70690 | 1.94E+05 | 71% | 0 | 99.66 | 96727 | 96993 | CP097706.1 |
|  | *Klebsiella pneumoniae* strain IR12024_1 plasmid unnamed1 | *Klebsiella pneumoniae* | 70690 | 2.16E+05 | 72% | 0 | 99.66 | 96727 | 103399 | CP097676.1 |
|  | *Klebsiella pneumoniae* strain IR5755 plasmid unnamed1, complete sequence | *Klebsiella pneumoniae* | 70655 | 2.12E+05 | 72% | 0 | 99.65 | 96727 | 103224 | CP061971.1 |
|  | *Klebsiella pneumoniae* strain IR5726 plasmid unnamed2, complete sequence | *Klebsiella pneumoniae* | 70641 | 2.28E+05 | 72% | 0 | 99.64 | 96727 | 110359 | CP061959.1 |
|  | *Klebsiella pneumoniae* strain JX-CR-hvKP-1 plasmid pJX1-3, complete sequence | *Klebsiella pneumoniae* | 69734 | 1.15E+05 | 72% | 0 | 100 | 96727 | 55186 | CP064255.1 |
|  | *Klebsiella pneumoniae* strain JX-CR-hvKP-2 plasmid pJX2-3, complete sequence | *Klebsiella pneumoniae* | 66827 | 1.13E+05 | 71% | 0 | 99.38 | 96727 | 56365 | CP064249.1 |
|  | *Klebsiella pneumoniae* strain KP18-2079 plasmid pKP18-2079_54kb, complete sequence | *Klebsiella pneumoniae* | 62404 | 1.08E+05 | 69% | 0 | 98.75 | 96727 | 54881 | MT090961.1 |
|  | *Klebsiella pneumoniae* strain 19PDR22 plasmid p6, complete sequence | *Klebsiella pneumoniae* | 49517 | 63169 | 42% | 0 | 99.99 | 96727 | 30607 | CP076550.1 |
|  | *Klebsiella pneumoniae* strain hvKP340 plasmid unnamed3, complete sequence | *Klebsiella pneumoniae* | 47157 | 1.15E+05 | 71% | 0 | 99.99 | 96727 | 55186 | CP101779.1 |
|  | *Klebsiella pneumoniae* *subsp.* pneumoniae strain DD01754 plasmid pDD01754-3, complete sequence | *Klebsiella pneumoniae* *subsp.* pneumoniae | 44937 | 1.13E+05 | 71% | 0 | 99.1 | 96727 | 56365 | CP087648.1 |
|  | *Klebsiella pneumoniae* strain WCHKP040035 plasmid p1_040035, complete sequence | *Klebsiella pneumoniae* | 44040 | 1.06E+05 | 68% | 0 | 98.59 | 96727 | 54847 | CP028794.1 |
|  | Klebsiella phage GTai-2021a, complete sequence | Klebsiella phage GTai-2021a | 39539 | 1.04E+05 | 64% | 0 | 98.41 | 96727 | 58864 | CP072507.1 |
|  | Klebsiella phage 48ST307, complete genome | Klebsiella phage 48ST307 | 39325 | 88296 | 59% | 0 | 98.23 | 96727 | 52338 | KY271402.1 |
|  | *Klebsiella pneumoniae* isolate CNR146C9 genome assembly, plasmid: p2 | *Klebsiella pneumoniae* | 35820 | 88393 | 59% | 0 | 98.12 | 96727 | 52608 | OV408204.1 |
|  | *Klebsiella pneumoniae* strain KPH1 plasmid p3, complete sequence | *Klebsiella pneumoniae* | 35569 | 87716 | 58% | 0 | 98.22 | 96727 | 52856 | CP102549.1 |
|  | *Klebsiella pneumoniae* strain IR12182_1 plasmid unnamed2, complete sequence | *Klebsiella pneumoniae* | 33096 | 72387 | 24% | 0 | 99.29 | 96727 | 214956 | CP097696.1 |
|  | *Klebsiella pneumoniae* *subsp.* pneumoniae strain kpn-hnqyy plasmid unnamed1, complete sequence | *Klebsiella pneumoniae* *subsp.* pneumoniae | 29903 | 1.58E+05 | 66% | 0 | 99.22 | 96727 | 123557 | CP074117.1 |
|  | *Klebsiella pneumoniae* *subsp.* pneumoniae strain ST2017:950142398 plasmid p18-43_03 | *Klebsiella pneumoniae* *subsp.* pneumoniae | 27100 | 98396 | 57% | 0 | 98.15 | 96727 | 65209 | CP023556.1 |
|  | *Klebsiella pneumoniae* isolate 147 genome assembly, plasmid: P3 | *Klebsiella pneumoniae* | 25579 | 87886 | 60% | 0 | 96.97 | 96727 | 55200 | OW970433.1 |
|  | *Klebsiella pneumoniae* isolate INF302-sc-2280090 genome assembly, plasmid: 3 | *Klebsiella pneumoniae* | 25412 | 98813 | 64% | 0 | 99.12 | 96727 | 53776 | LR890558.1 |
|  | *Klebsiella pneumoniae* isolate KSB2_5A-sc-2280323 genome assembly, plasmid: 2 | *Klebsiella pneumoniae* | 25412 | 98818 | 64% | 0 | 99.12 | 96727 | 53776 | LR890532.1 |
|  | *Klebsiella pneumoniae* isolate KSB2_10B-sc-2280350 genome assembly, plasmid: 3 | *Klebsiella pneumoniae* | 25412 | 98884 | 64% | 0 | 99.09 | 96727 | 53776 | LR890486.1 |
|  | *Klebsiella pneumoniae* strain RHB30-C05 plasmid pRHB30-C05_5, complete sequence | *Klebsiella pneumoniae* | 25412 | 98651 | 64% | 0 | 99.12 | 96727 | 53776 | CP057317.1 |
|  | *Klebsiella pneumoniae* strain INF116-sc-2279924 plasmid unnamed2, complete sequence | *Klebsiella pneumoniae* | 25261 | 98764 | 65% | 0 | 98.9 | 96727 | 53774 | CP031794.1 |
|  | *Klebsiella pneumoniae* isolate KP9201 genome assembly, plasmid: 4 | *Klebsiella pneumoniae* | 25178 | 1.00E+05 | 64% | 0 | 98.96 | 96727 | 55398 | LR025094.1 |
|  | *Klebsiella pneumoniae* isolate KP980 genome assembly, plasmid: 4 | *Klebsiella pneumoniae* | 25178 | 98401 | 63% | 0 | 98.96 | 96727 | 53251 | LR025090.1 |
|  | *Klebsiella pneumoniae* strain Kp7224 chromosome | *Klebsiella pneumoniae* | 25030 | 56499 | 36% | 0 | 99.54 | 96727 | 5514367 | CP074199.1 |
|  | *Klebsiella pneumoniae* isolate 15 genome assembly, plasmid: P3 | *Klebsiella pneumoniae* | 24866 | 87474 | 58% | 0 | 98.56 | 96727 | 53196 | OW968321.1 |
|  | Klebsiella variicola strain DX120E plasmid pKV2, complete sequence | Klebsiella variicola | 24794 | 98772 | 65% | 0 | 97.35 | 96727 | 54715 | CP009276.1 |
|  | *Klebsiella pneumoniae* isolate 392 genome assembly, plasmid: P2 | *Klebsiella pneumoniae* | 24772 | 88543 | 59% | 0 | 98.44 | 96727 | 53918 | OW959916.1 |
|  | *Klebsiella pneumoniae* isolate 15 genome assembly, plasmid: P6 | *Klebsiella pneumoniae* | 24772 | 88566 | 59% | 0 | 98.44 | 96727 | 53928 | OW968257.1 |
|  | *Klebsiella pneumoniae* isolate 15 genome assembly, plasmid: P5 | *Klebsiella pneumoniae* | 24772 | 88633 | 59% | 0 | 98.44 | 96727 | 53928 | OW848837.1 |
|  | *Klebsiella pneumoniae* strain 1_GR_13 plasmid unnamed, complete sequence | *Klebsiella pneumoniae* | 24484 | 71167 | 48% | 0 | 98.07 | 96727 | 55018 | CP027046.1 |
|  | *Klebsiella pneumoniae* strain AR_0046 plasmid unnamed2, complete sequence | *Klebsiella pneumoniae* | 24484 | 85052 | 49% | 0 | 98.07 | 96727 | 73056 | CP032224.1 |
|  | *Klebsiella pneumoniae* strain FDAARGOS_442 plasmid unnamed3, complete sequence | *Klebsiella pneumoniae* | 24484 | 1.03E+05 | 49% | 0 | 98.07 | 96727 | 88025 | CP023927.1 |
|  | *Klebsiella pneumoniae* strain Nord5-1_R48 plasmid pR48_5, complete sequence | *Klebsiella pneumoniae* | 24484 | 71149 | 48% | 0 | 98.07 | 96727 | 55026 | CP091590.1 |
|  | *Klebsiella pneumoniae* strain ARLG-4866 plasmid pC597_3, complete sequence | *Klebsiella pneumoniae* | 24371 | 88558 | 59% | 0 | 96.83 | 96727 | 54134 | CP067601.1 |
|  | *Klebsiella pneumoniae* strain ARLG-8053 plasmid pC717_2, complete sequence | *Klebsiella pneumoniae* | 24371 | 88558 | 59% | 0 | 96.84 | 96727 | 54134 | CP067497.1 |
|  | *Klebsiella pneumoniae* strain KP5 plasmid pSg1-3, complete sequence | *Klebsiella pneumoniae* | 24336 | 82652 | 54% | 0 | 99.17 | 96727 | 46883 | CP012429.1 |
|  | *Klebsiella pneumoniae* strain HKP0067 plasmid pHKP0067.4, complete sequence | *Klebsiella pneumoniae* | 24308 | 90000 | 59% | 0 | 99.14 | 96727 | 57450 | CP061057.1 |
|  | *Klebsiella pneumoniae* isolate INF305-sc-2280094 genome assembly, plasmid: 7 | *Klebsiella pneumoniae* | 24275 | 87886 | 59% | 0 | 99.09 | 96727 | 53758 | LR890708.1 |
|  | *Klebsiella pneumoniae* isolate INF281-sc-2280206 genome assembly, plasmid: 7 | *Klebsiella pneumoniae* | 24275 | 87964 | 60% | 0 | 99.09 | 96727 | 53943 | LR890521.1 |
|  | *Klebsiella pneumoniae* isolate KSB1_5H-sc-2280283 genome assembly, plasmid: 7 | *Klebsiella pneumoniae* | 24275 | 87965 | 60% | 0 | 99.09 | 96727 | 53943 | LR890506.1 |
|  | *Klebsiella pneumoniae* isolate INF298 genome assembly, plasmid: 7 | *Klebsiella pneumoniae* | 24275 | 87902 | 59% | 0 | 99.09 | 96727 | 53938 | LR890432.1 |
|  | *Klebsiella pneumoniae* *subsp.* pneumoniae strain WGR351 plasmid p3WGR351, complete sequence | *Klebsiella pneumoniae* *subsp.* pneumoniae | 24260 | 70904 | 49% | 0 | 98.18 | 96727 | 54615 | CP076011.1 |
|  | *Klebsiella pneumoniae* strain ARLG-3204 plasmid pC258_4, complete sequence | *Klebsiella pneumoniae* | 24103 | 91298 | 62% | 0 | 98.63 | 96727 | 55121 | CP067889.1 |
|  | *Klebsiella pneumoniae* strain 1050 plasmid unnamed, complete sequence | *Klebsiella pneumoniae* | 23998 | 95603 | 62% | 0 | 98.49 | 96727 | 53096 | CP023421.1 |
|  | *Klebsiella pneumoniae* strain 47733 plasmid p47733L, complete sequence | *Klebsiella pneumoniae* | 23859 | 93667 | 63% | 0 | 98.31 | 96727 | 55119 | CP050366.1 |
|  | *Klebsiella pneumoniae* strain CRKP-2297 plasmid pCRKP-2297_3, complete sequence | *Klebsiella pneumoniae* | 23859 | 1.20E+05 | 64% | 0 | 98.31 | 96727 | 69628 | CP024837.1 |
|  | *Klebsiella pneumoniae* strain AR_0138 plasmid tig00000003, complete sequence | *Klebsiella pneumoniae* | 23859 | 95638 | 63% | 0 | 98.31 | 96727 | 54404 | CP021760.1 |
|  | *Klebsiella pneumoniae* isolate 147 genome assembly, plasmid: P4 | *Klebsiella pneumoniae* | 23859 | 95696 | 63% | 0 | 98.31 | 96727 | 54404 | OW970564.1 |
|  | *Klebsiella pneumoniae* isolate 147 genome assembly, plasmid: P4 | *Klebsiella pneumoniae* | 23859 | 95725 | 63% | 0 | 98.31 | 96727 | 54404 | OW970557.1 |
|  | *Klebsiella pneumoniae* isolate 147 genome assembly, plasmid: P4 | *Klebsiella pneumoniae* | 23859 | 95701 | 63% | 0 | 98.31 | 96727 | 54404 | OW970544.1 |
|  | *Klebsiella pneumoniae* isolate 147 genome assembly, plasmid: P3 | *Klebsiella pneumoniae* | 23859 | 95706 | 63% | 0 | 98.31 | 96727 | 54404 | OW970533.1 |
|  | *Klebsiella pneumoniae* isolate 147 genome assembly, plasmid: P3 | *Klebsiella pneumoniae* | 23859 | 95725 | 63% | 0 | 98.31 | 96727 | 54404 | OW970524.1 |
|  | *Klebsiella pneumoniae* isolate 147 genome assembly, plasmid: P2 | *Klebsiella pneumoniae* | 23859 | 95707 | 63% | 0 | 98.31 | 96727 | 54404 | OW970516.1 |
|  | *Klebsiella pneumoniae* isolate 147 genome assembly, plasmid: P4 | *Klebsiella pneumoniae* | 23859 | 95630 | 63% | 0 | 98.31 | 96727 | 55181 | OW970496.1 |
|  | *Klebsiella pneumoniae* isolate 147 genome assembly, plasmid: P3 | *Klebsiella pneumoniae* | 23859 | 95699 | 63% | 0 | 98.31 | 96727 | 54404 | OW970489.1 |
|  | *Klebsiella pneumoniae* isolate 147 genome assembly, plasmid: P2 | *Klebsiella pneumoniae* | 23859 | 95707 | 63% | 0 | 98.31 | 96727 | 54404 | OW970480.1 |
|  | *Klebsiella pneumoniae* isolate 147 genome assembly, plasmid: P4 | *Klebsiella pneumoniae* | 23859 | 95707 | 63% | 0 | 98.31 | 96727 | 54404 | OW970456.1 |
|  | *Klebsiella pneumoniae* isolate 147 genome assembly, plasmid: P3 | *Klebsiella pneumoniae* | 23859 | 95646 | 63% | 0 | 98.31 | 96727 | 55958 | OW970425.1 |
|  | *Klebsiella pneumoniae* isolate 147 genome assembly, plasmid: P4 | *Klebsiella pneumoniae* | 23859 | 95754 | 63% | 0 | 98.31 | 96727 | 54404 | OW970383.1 |
|  | *Klebsiella pneumoniae* isolate 147 genome assembly, plasmid: P4 | *Klebsiella pneumoniae* | 23859 | 90169 | 62% | 0 | 98.31 | 96727 | 53996 | OW970369.1 |
|  | *Klebsiella pneumoniae* isolate 147 genome assembly, plasmid: P4 | *Klebsiella pneumoniae* | 23859 | 95703 | 63% | 0 | 98.31 | 96727 | 54404 | OW970361.1 |
|  | *Klebsiella pneumoniae* isolate 147 genome assembly, plasmid: P4 | *Klebsiella pneumoniae* | 23859 | 95708 | 63% | 0 | 98.31 | 96727 | 54404 | OW969776.1 |
|  | *Klebsiella pneumoniae* isolate 147 genome assembly, plasmid: P3 | *Klebsiella pneumoniae* | 23859 | 95707 | 63% | 0 | 98.31 | 96727 | 54404 | OW967818.1 |
|  | *Klebsiella pneumoniae* strain DJ genome assembly, plasmid: DJ_-phage_N15 | *Klebsiella pneumoniae* | 23859 | 93630 | 63% | 0 | 98.31 | 96727 | 57678 | OV049816.1 |
|  | *Klebsiella pneumoniae* strain CRKP-1215 plasmid pCRKP-1215_3, complete sequence | *Klebsiella pneumoniae* | 23846 | 1.25E+05 | 64% | 0 | 98.3 | 96727 | 72689 | CP024841.1 |
|  | *Klebsiella pneumoniae* strain 1613258639 plasmid p1-1613258639 | *Klebsiella pneumoniae* | 23721 | 94257 | 62% | 0 | 98.2 | 96727 | 204868 | CP083056.1 |
|  | *Klebsiella pneumoniae* strain KP2 plasmid pKP2_7, complete sequence | *Klebsiella pneumoniae* | 23562 | 93039 | 63% | 0 | 97.91 | 96727 | 55133 | CP041953.1 |
|  | *Klebsiella pneumoniae* strain BA39649 plasmid p_unnamed_1_VBA39649, complete sequence | *Klebsiella pneumoniae* | 23464 | 88916 | 59% | 0 | 98.39 | 96727 | 57223 | CP058944.1 |
|  | *Klebsiella pneumoniae* strain ARLG-3237 plasmid pC291_2, complete sequence | *Klebsiella pneumoniae* | 22255 | 51132 | 34% | 0 | 98.62 | 96727 | 73047 | CP067810.1 |
|  | *Klebsiella pneumoniae* strain TH164 chromosome, complete genome | *Klebsiella pneumoniae* | 21970 | 52076 | 34% | 0 | 98.6 | 96727 | 5381065 | CP035210.1 |
|  | *Klebsiella pneumoniae* strain SKGH01 plasmid unnamed 4, complete sequence | *Klebsiella pneumoniae* | 21621 | 83357 | 57% | 0 | 97.99 | 96727 | 56230 | CP015504.1 |
|  | *Klebsiella pneumoniae* strain MS6671 genome assembly, plasmid: _Phage_Kpneumoniae_MS6671 | *Klebsiella pneumoniae* | 21621 | 82392 | 57% | 0 | 97.99 | 96727 | 55416 | LN824139.1 |
|  | *Klebsiella pneumoniae* strain MH9CRKP chromosome, complete genome | *Klebsiella pneumoniae* | 21457 | 54587 | 34% | 0 | 99.24 | 96727 | 5541076 | CP048412.1 |
|  | *Klebsiella pneumoniae* isolate INF333 genome assembly, chromosome: 1 | *Klebsiella pneumoniae* | 20842 | 52268 | 34% | 0 | 98.31 | 96727 | 5227693 | LR890384.1 |
|  | *Klebsiella pneumoniae* isolate KSB2_2B-sc-2280339 genome assembly, chromosome: 1 | *Klebsiella pneumoniae* | 20831 | 52429 | 34% | 0 | 98.29 | 96727 | 5245448 | LR890343.1 |
|  | *Klebsiella pneumoniae* isolate KSB2_2C-sc-2280354 genome assembly, chromosome: 1 | *Klebsiella pneumoniae* | 20814 | 52497 | 34% | 0 | 98.27 | 96727 | 5228933 | LR890391.1 |
|  | *Klebsiella pneumoniae* isolate KSB1_6J genome assembly, chromosome: 1 | *Klebsiella pneumoniae* | 20803 | 52609 | 34% | 0 | 98.25 | 96727 | 5226186 | LR890230.1 |
|  | *Klebsiella pneumoniae* isolate KSB2_9A-sc-2280332 genome assembly, chromosome: 1 | *Klebsiella pneumoniae* | 20792 | 52497 | 34% | 0 | 98.23 | 96727 | 5245542 | LR890564.1 |
|  | *Klebsiella pneumoniae* isolate KSB2_8B-sc-2280349 genome assembly, chromosome: 1 | *Klebsiella pneumoniae* | 20770 | 51955 | 34% | 0 | 98.2 | 96727 | 5233233 | LR890451.1 |
|  | *Klebsiella pneumoniae* isolate KSB2_10B-sc-2280350 genome assembly, chromosome: 1 | *Klebsiella pneumoniae* | 20764 | 51833 | 34% | 0 | 98.19 | 96727 | 5228900 | LR890484.1 |
|  | *Klebsiella pneumoniae* strain KSB1_10J chromosome, complete genome | *Klebsiella pneumoniae* | 20753 | 51811 | 34% | 0 | 98.17 | 96727 | 5228900 | CP024515.1 |
|  | *Klebsiella pneumoniae* strain KSB2_1B chromosome, complete genome | *Klebsiella pneumoniae* | 20753 | 51811 | 34% | 0 | 98.17 | 96727 | 5228889 | CP024504.1 |
|  | *Klebsiella pneumoniae* strain KPN110 chromosome, complete genome | *Klebsiella pneumoniae* | 20753 | 51811 | 34% | 0 | 98.17 | 96727 | 5275802 | CP065172.1 |
|  | *Klebsiella pneumoniae* isolate KSB1_1H genome assembly, chromosome: 1 | *Klebsiella pneumoniae* | 20753 | 51811 | 34% | 0 | 98.17 | 96727 | 5230117 | LR890722.1 |
|  | *Klebsiella pneumoniae* isolate INF327-sc-2280132 genome assembly, chromosome: 1 | *Klebsiella pneumoniae* | 20753 | 51811 | 34% | 0 | 98.17 | 96727 | 5228889 | LR890571.1 |
|  | *Klebsiella pneumoniae* isolate INF302-sc-2280090 genome assembly, chromosome: 1 | *Klebsiella pneumoniae* | 20753 | 51811 | 34% | 0 | 98.17 | 96727 | 5228896 | LR890556.1 |
|  | *Klebsiella pneumoniae* isolate INF018-sc-2279888 genome assembly, chromosome: 1 | *Klebsiella pneumoniae* | 20753 | 51811 | 34% | 0 | 98.17 | 96727 | 5231789 | LR890493.1 |
|  | *Klebsiella pneumoniae* isolate INF148-sc-2279987 genome assembly, chromosome: 1 | *Klebsiella pneumoniae* | 20753 | 51811 | 34% | 0 | 98.17 | 96727 | 5460439 | LR890381.1 |
|  | *Klebsiella pneumoniae* isolate INF329-sc-2280137 genome assembly, chromosome: 1 | *Klebsiella pneumoniae* | 20753 | 51811 | 34% | 0 | 98.17 | 96727 | 5230561 | LR890240.1 |
|  | *Klebsiella pneumoniae* isolate INF277 genome assembly, chromosome: 1 | *Klebsiella pneumoniae* | 20753 | 51811 | 34% | 0 | 98.17 | 96727 | 5230141 | LR890204.1 |
|  | *Klebsiella pneumoniae* isolate INF133-sc-2279960 genome assembly, chromosome: 1 | *Klebsiella pneumoniae* | 20753 | 51811 | 34% | 0 | 98.17 | 96727 | 5230102 | LR890189.1 |
|  | *Klebsiella pneumoniae* strain KSB1_4E chromosome, complete genome | *Klebsiella pneumoniae* | 20751 | 51809 | 34% | 0 | 98.21 | 96727 | 5234963 | CP024499.1 |
|  | *Klebsiella pneumoniae* strain KSB1_7E chromosome, complete genome | *Klebsiella pneumoniae* | 20751 | 51809 | 34% | 0 | 98.21 | 96727 | 5232513 | CP024496.1 |
|  | *Klebsiella pneumoniae* isolate KSB2_5A-sc-2280323 genome assembly, chromosome: 1 | *Klebsiella pneumoniae* | 20742 | 51954 | 34% | 0 | 98.16 | 96727 | 5473592 | LR890531.1 |
|  | *Klebsiella pneumoniae* *subsp.* pneumoniae Kp13 chromosome, complete genome | *Klebsiella pneumoniae* *subsp.* pneumoniae Kp13 | 20737 | 51146 | 33% | 0 | 98.15 | 96727 | 5307003 | CP003999.1 |
|  | Klebsiella sp. LY chromosome, complete genome | Klebsiella sp. LY | 20692 | 38724 | 26% | 0 | 98.11 | 96727 | 5158003 | CP022444.1 |
|  | *Klebsiella pneumoniae* strain FDAARGOS_1306 chromosome, complete genome | *Klebsiella pneumoniae* | 20624 | 43704 | 28% | 0 | 98.21 | 96727 | 5345150 | CP070088.1 |
| pCR-hvKP006-P3 | *Klebsiella pneumoniae* strain WCHKP090050 plasmid p1_090050, complete sequence | *Klebsiella pneumoniae* | 18399 | 18579 | 100% | 0 | 100 | 10060 | 10060 | CP043371.1 |
|  | *Klebsiella pneumoniae* strain LSH-KPN148 plasmid pLSH-KPN148-3, complete sequence | *Klebsiella pneumoniae* | 18205 | 18579 | 100% | 0 | 100 | 10060 | 10060 | CP040125.1 |
|  | *Klebsiella pneumoniae* strain KP18-2050 plasmid pKP18-2050-3, complete sequence | *Klebsiella pneumoniae* | 18000 | 18197 | 100% | 0 | 99.33 | 10060 | 10049 | CP082022.1 |
|  | *Klebsiella pneumoniae* *subsp.* pneumoniae strain DD02162 plasmid pDD02162-4, complete sequence | *Klebsiella pneumoniae* *subsp.* pneumoniae | 17932 | 18579 | 100% | 0 | 100 | 10060 | 10060 | CP087622.1 |
|  | *Klebsiella pneumoniae* strain FDAARGOS_444 plasmid unnamed3, complete sequence | *Klebsiella pneumoniae* | 17921 | 18573 | 100% | 0 | 99.99 | 10060 | 10061 | CP023944.1 |
|  | *Klebsiella pneumoniae* strain WCHKP115011 plasmid p2_115011, complete sequence | *Klebsiella pneumoniae* | 17821 | 18579 | 100% | 0 | 100 | 10060 | 10060 | CP089957.1 |
|  | *Klebsiella pneumoniae* strain SW1780 plasmid pB, complete sequence | *Klebsiella pneumoniae* | 17821 | 18579 | 100% | 0 | 100 | 10060 | 10060 | CP073304.1 |
|  | *Klebsiella pneumoniae* strain WCHKP7E2 plasmid p1_085072, complete sequence | *Klebsiella pneumoniae* | 17501 | 18579 | 100% | 0 | 100 | 10060 | 10060 | CP028801.2 |
|  | *Klebsiella pneumoniae* strain KP167 plasmid pKP167-10, complete sequence | *Klebsiella pneumoniae* | 17472 | 18579 | 100% | 0 | 100 | 10060 | 10060 | CP098761.1 |
|  | *Klebsiella pneumoniae* strain 135077 plasmid p2_135077, complete sequence | *Klebsiella pneumoniae* | 17472 | 18579 | 100% | 0 | 100 | 10060 | 10060 | CP073294.1 |
|  | *Klebsiella pneumoniae* strain KPN142 plasmid pn142_2, complete sequence | *Klebsiella pneumoniae* | 17398 | 18579 | 100% | 0 | 100 | 10060 | 10060 | CP053877.1 |
|  | *Klebsiella pneumoniae* strain XHKP6 plasmid pXHKP6-2, complete sequence | *Klebsiella pneumoniae* | 17248 | 18614 | 100% | 0 | 99.99 | 10060 | 10098 | CP066889.1 |
|  | *Klebsiella pneumoniae* strain KP19-2029 plasmid pKP19-2029-Col, complete sequence | *Klebsiella pneumoniae* | 17224 | 18579 | 100% | 0 | 100 | 10060 | 10060 | CP047162.1 |
|  | *Klebsiella pneumoniae* strain ZRKP03 plasmid unnamed4, complete sequence | *Klebsiella pneumoniae* | 17163 | 18546 | 100% | 0 | 99.94 | 10060 | 10060 | CP050346.1 |
|  | *Klebsiella pneumoniae* strain WCHKP090045 plasmid p1_090045, complete sequence | *Klebsiella pneumoniae* | 17047 | 18579 | 100% | 0 | 100 | 10060 | 10060 | CP043367.1 |
|  | *Klebsiella pneumoniae* strain KP4863 plasmid pKP4863-2, complete sequence |  | 17016 | 34033 | 100% | 0 | 97.21 | 10060 | 20110 | CP102843.1 |
|  | *Klebsiella pneumoniae* GSU10-3 plasmid pGSU10-3-4 DNA, complete genome | *Klebsiella pneumoniae* | 16750 | 18579 | 100% | 0 | 100 | 10060 | 10060 | AP018675.1 |
|  | *Klebsiella pneumoniae* *subsp.* pneumoniae strain kpn-hnqyy plasmid unnamed3, complete sequence | *Klebsiella pneumoniae* *subsp.* pneumoniae | 16560 | 18579 | 100% | 0 | 100 | 10060 | 10060 | CP074119.1 |
|  | *Klebsiella pneumoniae* strain 21080237 plasmid p21080237_6, complete sequence | *Klebsiella pneumoniae* | 16541 | 18573 | 100% | 0 | 99.99 | 10060 | 10060 | CP095246.1 |
|  | *Klebsiella pneumoniae* strain BSI073 plasmid pBSI073-KPC2 | *Klebsiella pneumoniae* | 16366 | 18594 | 100% | 0 | 100 | 10060 | 118923 | MT269846.1 |
|  | *Klebsiella pneumoniae* strain KP18-1 plasmid pKP18-1-2, complete sequence | *Klebsiella pneumoniae* | 16070 | 18245 | 99% | 0 | 99.82 | 10060 | 10049 | CP082002.1 |
|  | *Klebsiella pneumoniae* strain XHKP53 plasmid pXHKP53-2, complete sequence | *Klebsiella pneumoniae* | 16026 | 18064 | 100% | 0 | 99.35 | 10060 | 10013 | CP066893.1 |
|  | *Klebsiella pneumoniae* isolate 91eed288-b809-11e8-aae5-3c4a9275d6c8 genome assembly, chromosome: 1 | *Klebsiella pneumoniae* | 15993 | 19203 | 100% | 0 | 100 | 10060 | 5510473 | LR596808.1 |
|  | *Klebsiella pneumoniae* strain 21072329 plasmid p21072329_4, complete sequence | *Klebsiella pneumoniae* | 15911 | 18579 | 100% | 0 | 100 | 10060 | 10060 | CP095238.1 |
|  | *Klebsiella pneumoniae* strain WCHKP8F4 plasmid p1_095084, complete sequence | *Klebsiella pneumoniae* | 15645 | 18579 | 100% | 0 | 100 | 10060 | 10060 | CP027065.3 |
|  | *Klebsiella pneumoniae* strain 140253 plasmid p3_140253, complete sequence | *Klebsiella pneumoniae* | 15640 | 18579 | 100% | 0 | 100 | 10060 | 10060 | CP097630.1 |
|  | *Klebsiella pneumoniae* *subsp.* pneumoniae strain SCKP020143 plasmid p3_020143, complete sequence | *Klebsiella pneumoniae* *subsp.* pneumoniae | 15603 | 18579 | 100% | 0 | 100 | 10060 | 10060 | CP028545.2 |
|  | *Klebsiella pneumoniae* strain 1632 plasmid p1632-3, complete sequence | *Klebsiella pneumoniae* | 15570 | 18579 | 100% | 0 | 100 | 10060 | 10060 | CP084500.1 |
|  | *Klebsiella pneumoniae* plasmid unnamed, complete sequence | *Klebsiella pneumoniae* | 15477 | 18579 | 100% | 0 | 100 | 10060 | 10060 | MK181634.1 |
|  | *Klebsiella pneumoniae* *subsp.* pneumoniae strain WCHKP020120 plasmid p2_020120, complete sequence | *Klebsiella pneumoniae* *subsp.* pneumoniae | 15418 | 18579 | 100% | 0 | 100 | 10060 | 10060 | CP043360.1 |
|  | Serratia marcescens Sm4 plasmid pSm4Col DNA, complete sequence | Serratia marcescens | 15407 | 18579 | 100% | 0 | 100 | 10060 | 10060 | LC543864.1 |
|  | *Klebsiella pneumoniae* strain WCHKP649 plasmid p1_095649, complete sequence | *Klebsiella pneumoniae* | 15271 | 18579 | 100% | 0 | 100 | 10060 | 10060 | CP026582.3 |
|  | *Klebsiella pneumoniae* strain 160111 plasmid p10K_L111, complete sequence | *Klebsiella pneumoniae* | 15139 | 18579 | 100% | 0 | 100 | 10060 | 10060 | CP030130.1 |
|  | *Klebsiella pneumoniae* *subsp.* pneumoniae strain DD01754 plasmid pDD01754-4, complete sequence | *Klebsiella pneumoniae* *subsp.* pneumoniae | 15080 | 18540 | 100% | 0 | 100 | 10060 | 10061 | CP087649.1 |
|  | *Klebsiella pneumoniae* strain LSH-KPN25 plasmid pLSH-KPN25-2, complete sequence | *Klebsiella pneumoniae* | 15066 | 18579 | 100% | 0 | 100 | 10060 | 10060 | CP040181.1 |
|  | *Klebsiella pneumoniae* strain XHKPN391 plasmid pXHKPN391-2, complete sequence | *Klebsiella pneumoniae* | 14951 | 18579 | 100% | 0 | 100 | 10060 | 10060 | CP066917.1 |
|  | *Klebsiella pneumoniae* strain JX-CR-hvKP-2 plasmid pJX2-4, complete sequence | *Klebsiella pneumoniae* | 14931 | 18579 | 100% | 0 | 100 | 10060 | 10060 | CP064250.1 |
|  | *Klebsiella pneumoniae* *subsp.* pneumoniae strain KP21 plasmid p21_2, complete sequence | *Klebsiella pneumoniae* *subsp.* pneumoniae | 14925 | 18573 | 100% | 0 | 99.99 | 10060 | 10061 | CP101544.1 |
|  | *Klebsiella pneumoniae* strain KPCZA02 plasmid pKPCZA02_1, complete sequence | *Klebsiella pneumoniae* | 14272 | 18577 | 99% | 0 | 100 | 10060 | 10059 | CP058227.1 |
|  | *Klebsiella pneumoniae* strain KP18-2073 plasmid pKP18-2073-3, complete sequence | *Klebsiella pneumoniae* | 14209 | 18197 | 100% | 0 | 100 | 10060 | 10049 | CP082027.1 |
|  | *Klebsiella pneumoniae* *subsp.* pneumoniae strain WCHKP015093 plasmid p2_015093, complete sequence | *Klebsiella pneumoniae* *subsp.* pneumoniae | 14113 | 18579 | 100% | 0 | 100 | 10060 | 10060 | CP036303.1 |
|  | *Klebsiella pneumoniae* strain HvKp-su1 plasmid unnamed2, complete sequence | *Klebsiella pneumoniae* | 14087 | 18581 | 100% | 0 | 100 | 10060 | 10061 | CP092719.1 |
|  | *Klebsiella pneumoniae* strain GD21SC417 plasmid pHNGS471-4, complete sequence | *Klebsiella pneumoniae* | 14079 | 18579 | 100% | 0 | 100 | 10060 | 10060 | CP089513.1 |
|  | *Klebsiella pneumoniae* strain KPN23 plasmid pKPN23_2, complete sequence | *Klebsiella pneumoniae* | 14078 | 18579 | 100% | 0 | 100 | 10060 | 10060 | CP089864.1 |
|  | *Klebsiella pneumoniae* strain ZRKP04 plasmid unnamed4, complete sequence | *Klebsiella pneumoniae* | 13884 | 18191 | 100% | 0 | 99.12 | 10060 | 10049 | CP050340.1 |
|  | *Klebsiella pneumoniae* strain KP55 plasmid pKPC-5504, complete sequence | *Klebsiella pneumoniae* | 13780 | 18572 | 100% | 0 | 99.99 | 10060 | 10059 | OL891654.1 |
|  | *Klebsiella pneumoniae* *subsp.* pneumoniae strain WCHKP020039 plasmid p1_020039, complete sequence | *Klebsiella pneumoniae* *subsp.* pneumoniae | 13605 | 18579 | 100% | 0 | 100 | 10060 | 10060 | CP043346.1 |
|  | *Klebsiella pneumoniae* strain KP69 plasmid p69-3, complete sequence | *Klebsiella pneumoniae* | 13598 | 18579 | 100% | 0 | 100 | 10060 | 10060 | CP025459.1 |
|  | *Klebsiella pneumoniae* strain ZRKP02 plasmid unnamed4, complete sequence | *Klebsiella pneumoniae* | 13411 | 18579 | 100% | 0 | 100 | 10060 | 10060 | CP050352.1 |
|  | *Klebsiella pneumoniae* strain WCHKP020030 plasmid p1_020030, complete sequence | *Klebsiella pneumoniae* | 13092 | 18579 | 100% | 0 | 100 | 10060 | 10060 | CP028788.2 |
|  | *Klebsiella pneumoniae* strain KP2509 plasmid pKP2509-5, complete sequence | *Klebsiella pneumoniae* | 13027 | 18579 | 100% | 0 | 100 | 10060 | 10060 | CP065951.1 |
|  | *Klebsiella pneumoniae* *subsp.* pneumoniae strain DD02172 plasmid pDD02172-5, complete sequence | *Klebsiella pneumoniae* *subsp.* pneumoniae | 12802 | 18579 | 100% | 0 | 100 | 10060 | 10060 | CP087616.1 |
|  | *Klebsiella pneumoniae* strain C789 plasmid unnamed1, complete sequence | *Klebsiella pneumoniae* | 12589 | 18573 | 100% | 0 | 100 | 10060 | 10061 | CP034418.1 |
|  | *Klebsiella pneumoniae* strain WCHKP3 plasmid p2_020003, complete sequence | *Klebsiella pneumoniae* | 12484 | 18579 | 100% | 0 | 100 | 10060 | 10060 | CP031718.1 |
|  | *Klebsiella pneumoniae* strain WCHKP115069 plasmid p2_115069, complete sequence | *Klebsiella pneumoniae* | 12438 | 18579 | 100% | 0 | 100 | 10060 | 10060 | CP033403.1 |
|  | *Klebsiella pneumoniae* strain KP46 plasmid pKP46_5, complete sequence | *Klebsiella pneumoniae* | 12412 | 18579 | 100% | 0 | 100 | 10060 | 10060 | CP090131.1 |
|  | *Klebsiella pneumoniae* strain KP18-8 plasmid pKP18-8-4, complete sequence | *Klebsiella pneumoniae* | 12227 | 18573 | 100% | 0 | 99.98 | 10060 | 10060 | CP082008.1 |
|  | *Klebsiella pneumoniae* strain WCHKP2 plasmid p1_020002, complete sequence | *Klebsiella pneumoniae* | 12028 | 18579 | 100% | 0 | 100 | 10060 | 10060 | CP028539.3 |
|  | *Klebsiella pneumoniae* strain SH12 plasmid pSH12_3, complete sequence | *Klebsiella pneumoniae* | 11810 | 18579 | 100% | 0 | 100 | 10060 | 10060 | CP040836.1 |
|  | *Klebsiella pneumoniae* strain JX-CR-hvKP-7 plasmid pJX7-4, complete sequence | *Klebsiella pneumoniae* | 11801 | 18579 | 100% | 0 | 100 | 10060 | 10060 | CP064227.1 |
|  | *Klebsiella pneumoniae* strain FO15 plasmid unnamed4, complete sequence | *Klebsiella pneumoniae* | 11795 | 27510 | 100% | 0 | 99.98 | 10060 | 14945 | CP073006.1 |
|  | *Klebsiella pneumoniae* strain 16HN-263 plasmid p16HN-263_2, complete sequence | *Klebsiella pneumoniae* | 11771 | 18579 | 100% | 0 | 100 | 10060 | 10060 | CP045265.1 |
|  | *Klebsiella pneumoniae* strain KP20194c4 plasmid pKP20194c4-p4, complete sequence | *Klebsiella pneumoniae* | 11716 | 18458 | 100% | 0 | 100 | 10060 | 11971 | CP054748.1 |
|  | *Klebsiella pneumoniae* strain KP18-2079 plasmid pKP18-2079_11kb, complete sequence | *Klebsiella pneumoniae* | 11716 | 18580 | 100% | 0 | 99.98 | 10060 | 11970 | MT090962.1 |
|  | *Klebsiella pneumoniae* strain WCHKP36 plasmid p2_020036, complete sequence | *Klebsiella pneumoniae* | 11716 | 18595 | 100% | 0 | 100 | 10060 | 12746 | CP028580.2 |
|  | *Klebsiella pneumoniae* strain L482 plasmid p5_L382, complete sequence | *Klebsiella pneumoniae* | 11716 | 18580 | 100% | 0 | 100 | 10060 | 11970 | CP033964.1 |
|  | *Klebsiella pneumoniae* isolate 97706988-b809-11e8-aae5-3c4a9275d6c8 genome assembly, chromosome: 1 | *Klebsiella pneumoniae* | 11716 | 19114 | 100% | 0 | 100 | 10060 | 5652578 | LR596813.1 |
|  | *Klebsiella pneumoniae* strain KP0079 plasmid pKP0079-4, complete sequence |  | 11716 | 37160 | 100% | 0 | 99.98 | 10060 | 23940 | CP102840.1 |
|  | *Klebsiella pneumoniae* strain S270v plasmid pS270V-3, complete sequence | *Klebsiella pneumoniae* | 11716 | 18579 | 100% | 0 | 100 | 10060 | 11970 | CP102195.1 |
|  | *Klebsiella pneumoniae* strain S234 plasmid pS234-3, complete sequence | *Klebsiella pneumoniae* | 11716 | 18580 | 100% | 0 | 99.98 | 10060 | 11970 | CP102189.1 |
|  | *Klebsiella pneumoniae* strain hvKP340 plasmid unnamed5, complete sequence | *Klebsiella pneumoniae* | 11716 | 18579 | 100% | 0 | 100 | 10060 | 11970 | CP101781.1 |
|  | *Klebsiella pneumoniae* strain hvKP841 plasmid unnamed3, complete sequence | *Klebsiella pneumoniae* | 11716 | 18579 | 100% | 0 | 100 | 10060 | 11970 | CP101787.1 |
|  | *Klebsiella pneumoniae* strain hvKP319 plasmid unnamed4, complete sequence | *Klebsiella pneumoniae* | 11716 | 18579 | 100% | 0 | 100 | 10060 | 11970 | CP101768.1 |
|  | *Klebsiella pneumoniae* strain KP15 plasmid unnamed3, complete sequence | *Klebsiella pneumoniae* | 11716 | 18579 | 100% | 0 | 100 | 10060 | 99065 | CP087145.1 |
|  | *Escherichia coli* strain CR-HvKP1TC-3 plasmid pCR-HvKP1TC-3_p4, complete sequence | *Escherichia coli* | 11716 | 18575 | 99% | 0 | 100 | 10060 | 11970 | OM001473.1 |
|  | *Klebsiella pneumoniae* strain WSCRKP plasmid pWSCRKP-4, complete sequence | *Klebsiella pneumoniae* | 11716 | 18594 | 100% | 0 | 100 | 10060 | 15931 | CP091072.1 |
|  | *Klebsiella pneumoniae* strain KP697 plasmid unnamed4, complete sequence | *Klebsiella pneumoniae* | 11716 | 18580 | 100% | 0 | 100 | 10060 | 11970 | CP066155.1 |
|  | *Klebsiella pneumoniae* *subsp.* pneumoniae strain KPN857 plasmid pD, complete sequence | *Klebsiella pneumoniae* *subsp.* pneumoniae | 11716 | 18580 | 100% | 0 | 99.98 | 10060 | 11970 | CP090436.1 |
|  | *Klebsiella pneumoniae* strain LZKP00003 plasmid pZR1, complete sequence | *Klebsiella pneumoniae* | 11716 | 18580 | 100% | 0 | 100 | 10060 | 11970 | CP089992.1 |
|  | *Klebsiella pneumoniae* strain 50700 plasmid p50700-12.0, complete sequence | *Klebsiella pneumoniae* | 11716 | 18393 | 100% | 0 | 100 | 10060 | 11972 | CP088993.1 |
|  | *Klebsiella pneumoniae* strain 8695 plasmid p4, complete sequence | *Klebsiella pneumoniae* | 11716 | 37104 | 100% | 0 | 100 | 10060 | 23957 | CP085893.1 |
|  | *Klebsiella pneumoniae* strain 12 plasmid pKP12_5, complete sequence | *Klebsiella pneumoniae* | 11716 | 18558 | 100% | 0 | 99.98 | 10060 | 11970 | CP082769.1 |
|  | *Klebsiella pneumoniae* strain KP18-2113 plasmid pKP18-2113-3, complete sequence | *Klebsiella pneumoniae* | 11716 | 18580 | 100% | 0 | 100 | 10060 | 11969 | CP082028.1 |
|  | *Klebsiella pneumoniae* strain KP-CT77 plasmid pCT77-tetA, complete sequence | *Klebsiella pneumoniae* | 11716 | 18537 | 100% | 0 | 99.98 | 10060 | 92645 | CP080306.1 |
|  | *Klebsiella pneumoniae* strain 19PDR22 plasmid p7, complete sequence | *Klebsiella pneumoniae* | 11716 | 18579 | 100% | 0 | 100 | 10060 | 11970 | CP076551.1 |
|  | *Klebsiella pneumoniae* strain DD521 plasmid pDD521.4, complete sequence | *Klebsiella pneumoniae* | 11716 | 18579 | 100% | 0 | 99.98 | 10060 | 11969 | CP075320.1 |
|  | *Escherichia coli* strain CR-HvKP5TC plasmid pCR-HvKP5TC_Vir-p4, complete sequence | *Escherichia coli* | 11716 | 18579 | 100% | 0 | 100 | 10060 | 190129 | MW598247.1 |
|  | *Escherichia coli* strain CR-HvKP3TC plasmid pCR-HvKP3TC_p4, complete sequence | *Escherichia coli* | 11716 | 18580 | 100% | 0 | 100 | 10060 | 11970 | MW598243.1 |
|  | *Escherichia coli* strain CR-HvKP1TC plasmid pCR-HvKP1TC_Vir-p4, complete sequence | *Escherichia coli* | 11716 | 18579 | 100% | 0 | 100 | 10060 | 190129 | MW598240.1 |
|  | *Klebsiella pneumoniae* strain CR-HvKP3 plasmid p4-CR-HvKP3, complete sequence | *Klebsiella pneumoniae* | 11716 | 18580 | 100% | 0 | 100 | 10060 | 11970 | MW598236.1 |
|  | *Klebsiella pneumoniae* strain KP55 plasmid pKP55_5, complete sequence | *Klebsiella pneumoniae* | 11716 | 18580 | 100% | 0 | 100 | 10060 | 11970 | CP055299.1 |
|  | *Klebsiella pneumoniae* strain BSI058 plasmid pBSI058-KPC2 | *Klebsiella pneumoniae* | 11716 | 18579 | 100% | 0 | 100 | 10060 | 114712 | MT269836.1 |
|  | *Klebsiella pneumoniae* strain 015625 plasmid p2_015625, complete sequence | *Klebsiella pneumoniae* | 11716 | 17042 | 91% | 0 | 99.98 | 10060 | 11904 | CP033392.2 |
|  | *Klebsiella pneumoniae* *subsp.* pneumoniae strain RJBSI76 plasmid pRJBSI76-4, complete sequence | *Klebsiella pneumoniae* *subsp.* pneumoniae | 11716 | 18573 | 100% | 0 | 99.98 | 10060 | 10206 | CP068693.1 |
|  | *Klebsiella pneumoniae* strain WCHKP090361 plasmid p2_090361, complete sequence | *Klebsiella pneumoniae* | 11716 | 18580 | 100% | 0 | 99.98 | 10060 | 11970 | CP066532.1 |
|  | *Klebsiella pneumoniae* strain WCHKP090329 plasmid p2_090329, complete sequence | *Klebsiella pneumoniae* | 11716 | 18579 | 100% | 0 | 99.98 | 10060 | 11970 | CP066521.1 |
|  | *Klebsiella pneumoniae* strain CRKP52R plasmid pCRKP52R-4-tetA, complete sequence | *Klebsiella pneumoniae* | 11716 | 18557 | 100% | 0 | 99.98 | 10060 | 99066 | CP066252.1 |
|  | *Klebsiella pneumoniae* strain JX-CR-hvKP-1 plasmid pJX1-4, complete sequence | *Klebsiella pneumoniae* | 11716 | 18579 | 100% | 0 | 100 | 10060 | 11970 | CP064256.1 |
|  | *Klebsiella pneumoniae* strain JX-CR-hvKP-4 plasmid pJX4-4, complete sequence | *Klebsiella pneumoniae* | 11716 | 18580 | 100% | 0 | 100 | 10060 | 11970 | CP064239.1 |
|  | *Klebsiella pneumoniae* strain FRPDR plasmid pFRPDR_5, complete sequence | *Klebsiella pneumoniae* | 11716 | 18579 | 100% | 0 | 100 | 10060 | 11970 | CP063764.1 |
| pCR-hvKP26-KPC-P1 | *Klebsiella pneumoniae* strain 16HN-263 plasmid p16HN-263_KPC, complete sequence | *Klebsiella pneumoniae* | 60718 | 3.54E+05 | 100% | 0 | 99.99 | 89906 | 129252 | CP045264.1 |
|  | *Klebsiella pneumoniae* strain EBSI036 plasmid pEBSI036-2-KPC, complete sequence | *Klebsiella pneumoniae* | 60718 | 3.69E+05 | 100% | 0 | 99.99 | 89906 | 129869 | MT648513.1 |
|  | *Klebsiella pneumoniae* strain IR12094_1 plasmid unnamed1, complete sequence | *Klebsiella pneumoniae* | 58578 | 3.38E+05 | 100% | 0 | 99.99 | 89906 | 130677 | CP097693.1 |
|  | *Klebsiella pneumoniae* strain SW1780 plasmid pSW1780-KPC, complete sequence | *Klebsiella pneumoniae* | 58578 | 3.83E+05 | 100% | 0 | 99.99 | 89906 | 125619 | CP073303.1 |
|  | *Klebsiella pneumoniae* strain KP69 plasmid p69-2, complete sequence | *Klebsiella pneumoniae* | 60707 | 3.57E+05 | 99% | 0 | 99.98 | 89906 | 128563 | CP025458.1 |
|  | *Klebsiella pneumoniae* strain KP18-2050 plasmid pKP18-2050-KPC2, complete sequence | *Klebsiella pneumoniae* | 60730 | 3.74E+05 | 98% | 0 | 100 | 89906 | 135837 | CP082020.1 |
|  | *Klebsiella pneumoniae* strain KP18-2073 plasmid pKP18-2073-KPC2, complete sequence | *Klebsiella pneumoniae* | 60730 | 3.73E+05 | 98% | 0 | 100 | 89906 | 130597 | CP082025.1 |
|  | *Klebsiella pneumoniae* strain JNKPN52 plasmid pJNKPN52_kpc_fosA, complete sequence | *Klebsiella pneumoniae* | 60702 | 3.35E+05 | 98% | 0 | 99.98 | 89906 | 127668 | MZ709016.1 |
|  | *Klebsiella pneumoniae* strain KP19-2029 plasmid pKP19-2029-KPC2, complete sequence | *Klebsiella pneumoniae* | 60683 | 3.45E+05 | 98% | 0 | 99.98 | 89906 | 127240 | CP047161.1 |
|  | *Klebsiella pneumoniae* strain 1864 plasmid p1864-2, complete sequence | *Klebsiella pneumoniae* | 57389 | 3.52E+05 | 98% | 0 | 99.99 | 89906 | 152725 | CP084494.1 |
|  | *Klebsiella pneumoniae* strain A1708 plasmid pA1708-KPC, complete sequence | *Klebsiella pneumoniae* | 57367 | 3.68E+05 | 98% | 0 | 99.98 | 89906 | 173280 | MT810354.1 |
|  | *Klebsiella pneumoniae* strain 911021 plasmid p911021-KPC, complete sequence | *Klebsiella pneumoniae* | 57361 | 3.38E+05 | 97% | 0 | 99.98 | 89906 | 169824 | MK036888.1 |
|  | *Klebsiella pneumoniae* strain KPN361 plasmid pKPN361-1, complete sequence | *Klebsiella pneumoniae* | 57361 | 3.35E+05 | 97% | 0 | 99.98 | 89906 | 169824 | CP053017.1 |
|  | *Klebsiella pneumoniae* strain 12139 plasmid p12139-KPC, complete sequence | *Klebsiella pneumoniae* | 57356 | 3.38E+05 | 97% | 0 | 99.97 | 89906 | 169424 | MF168403.1 |
|  | *Klebsiella pneumoniae* strain SH12 plasmid pSH12_KPC, complete sequence | *Klebsiella pneumoniae* | 57350 | 3.55E+05 | 97% | 0 | 99.97 | 89906 | 167468 | CP040835.1 |
|  | *Klebsiella pneumoniae* strain WCHKP020098 plasmid pKPC2_020098, complete sequence | *Klebsiella pneumoniae* | 57361 | 3.56E+05 | 95% | 0 | 99.98 | 89906 | 149519 | CP036306.1 |
|  | *Klebsiella pneumoniae* strain WCHKP090329 plasmid pKPC2_090329, complete sequence | *Klebsiella pneumoniae* | 57356 | 3.60E+05 | 95% | 0 | 99.97 | 89906 | 149181 | CP066518.1 |
|  | *Klebsiella pneumoniae* strain KPCZA02 plasmid pKPCZA02_2, complete sequence | *Klebsiella pneumoniae* | 57350 | 3.73E+05 | 95% | 0 | 99.97 | 89906 | 149200 | CP058228.1 |
|  | *Klebsiella pneumoniae* strain BSI055 plasmid pBSI055-KPC2 | *Klebsiella pneumoniae* | 57346 | 3.45E+05 | 95% | 0 | 99.97 | 89906 | 142890 | MT269834.1 |
|  | *Klebsiella pneumoniae* strain BSI032 plasmid pBSI032-KPC2 | *Klebsiella pneumoniae* | 57346 | 3.62E+05 | 95% | 0 | 99.97 | 89906 | 132173 | MT269827.1 |
|  | *Klebsiella pneumoniae* strain BSI010 plasmid pBSI010-KPC2 | *Klebsiella pneumoniae* | 57346 | 3.46E+05 | 95% | 0 | 99.97 | 89906 | 146268 | MT269820.1 |
|  | *Klebsiella pneumoniae* strain KP1880 plasmid pKPC1880, complete sequence | *Klebsiella pneumoniae* | 57345 | 3.30E+05 | 95% | 0 | 99.97 | 89906 | 168960 | CP061347.1 |
|  | *Klebsiella pneumoniae* strain 283747 plasmid p283747-1, complete sequence | *Klebsiella pneumoniae* | 57367 | 3.48E+05 | 94% | 0 | 99.98 | 89906 | 148021 | CP030301.1 |
|  | *Klebsiella pneumoniae* strain 283747 plasmid p283747-KPC, complete sequence | *Klebsiella pneumoniae* | 57367 | 3.51E+05 | 94% | 0 | 99.98 | 89906 | 148021 | MF168406.1 |
|  | *Klebsiella pneumoniae* *subsp.* pneumoniae strain DD01304 plasmid pDD01304-2, complete sequence | *Klebsiella pneumoniae* *subsp.* pneumoniae | 57367 | 2.98E+05 | 94% | 0 | 99.98 | 89906 | 169136 | CP087608.1 |
|  | *Klebsiella pneumoniae* strain F726925 plasmid pF726925-1, complete sequence | *Klebsiella pneumoniae* | 57367 | 3.57E+05 | 94% | 0 | 99.98 | 89906 | 172862 | CP081821.1 |
|  | *Klebsiella pneumoniae* strain JX-CR-hvKP-2 plasmid pJX2-2, complete sequence | *Klebsiella pneumoniae* | 57367 | 3.33E+05 | 94% | 0 | 99.98 | 89906 | 168025 | CP064248.1 |
|  | *Klebsiella pneumoniae* strain 156070 plasmid p156070-KPC, complete sequence | *Klebsiella pneumoniae* | 57363 | 3.29E+05 | 94% | 0 | 99.98 | 89906 | 138959 | MT109193.1 |
|  | *Klebsiella pneumoniae* strain FDAARGOS_444 plasmid unnamed2 | *Klebsiella pneumoniae* | 57363 | 3.71E+05 | 94% | 0 | 99.98 | 89906 | 187926 | CP023942.1 |
|  | *Klebsiella pneumoniae* strain WCHKP020037 plasmid pKPC2_020037, complete sequence | *Klebsiella pneumoniae* | 57361 | 3.62E+05 | 94% | 0 | 99.98 | 89906 | 172770 | CP036372.1 |
|  | *Klebsiella pneumoniae* *subsp.* pneumoniae strain DD01754 plasmid pDD01754-2, complete sequence | *Klebsiella pneumoniae* *subsp.* pneumoniae | 57361 | 3.81E+05 | 94% | 0 | 99.98 | 89906 | 182556 | CP087647.1 |
|  | *Klebsiella pneumoniae* strain BSI054 plasmid pBSI054-KPC2 | *Klebsiella pneumoniae* | 57350 | 3.40E+05 | 94% | 0 | 99.97 | 89906 | 168038 | MT269833.1 |
|  | *Klebsiella pneumoniae* strain BSI069 plasmid pBSI069-KPC2 | *Klebsiella pneumoniae* | 57346 | 3.40E+05 | 94% | 0 | 99.97 | 89906 | 138950 | MT269845.1 |
|  | *Klebsiella pneumoniae* strain BSI046 plasmid pBSI046-KPC2 | *Klebsiella pneumoniae* | 57345 | 3.40E+05 | 94% | 0 | 99.97 | 89906 | 166373 | MT269840.1 |
|  | *Klebsiella pneumoniae* strain ST11 plasmid p158590-KPC, complete sequence | *Klebsiella pneumoniae* | 57363 | 3.26E+05 | 93% | 0 | 99.98 | 89906 | 138959 | MT066418.1 |
|  | *Klebsiella pneumoniae* strain BSI052 plasmid pBSI052-KPC2 | *Klebsiella pneumoniae* | 57350 | 3.21E+05 | 93% | 0 | 99.97 | 89906 | 165819 | MT269832.1 |
|  | *Klebsiella pneumoniae* plasmid p1512-KPC, complete sequence | *Klebsiella pneumoniae* | 57346 | 3.11E+05 | 93% | 0 | 99.97 | 89906 | 117697 | MF918372.1 |
|  | *Klebsiella pneumoniae* isolate KSH203 plasmid pKSH203-KPC, complete sequence | *Klebsiella pneumoniae* | 57352 | 3.26E+05 | 92% | 0 | 99.97 | 89906 | 159467 | CP034324.1 |
|  | *Klebsiella pneumoniae* strain 675920 plasmid p675920-1, complete sequence | *Klebsiella pneumoniae* | 57350 | 2.95E+05 | 92% | 0 | 99.97 | 89906 | 163995 | MF133495.1 |
|  | *Klebsiella pneumoniae* strain BSI009 plasmid pBSI009-KPC2 | *Klebsiella pneumoniae* | 57346 | 3.23E+05 | 92% | 0 | 99.97 | 89906 | 164150 | MT269819.1 |
|  | *Klebsiella pneumoniae* strain 49088 plasmid p49088-279.2, complete sequence | *Klebsiella pneumoniae* | 57345 | 3.45E+05 | 92% | 0 | 99.97 | 89906 | 279210 | CP089000.1 |
|  | *Klebsiella pneumoniae* strain CR-HvKP2 plasmid pKPC-CR-HvKP2, complete sequence | *Klebsiella pneumoniae* | 57367 | 3.77E+05 | 91% | 0 | 99.98 | 89906 | 182593 | MW598248.1 |
|  | *Klebsiella pneumoniae* strain F127 plasmid pF127_1, complete sequence | *Klebsiella pneumoniae* | 57358 | 3.04E+05 | 91% | 0 | 99.98 | 89906 | 164501 | CP026141.1 |
|  | *Klebsiella pneumoniae* strain F1 plasmid pF1_1, complete sequence | *Klebsiella pneumoniae* | 57358 | 3.04E+05 | 91% | 0 | 99.98 | 89906 | 164510 | CP026131.1 |
|  | *Klebsiella pneumoniae* *subsp.* pneumoniae strain KP1034 plasmid pKP1034, complete sequence | *Klebsiella pneumoniae* *subsp.* pneumoniae | 57406 | 3.39E+05 | 90% | 0 | 100 | 89906 | 136848 | KP893385.1 |
|  | *Klebsiella pneumoniae* strain 40 plasmid pTZ40-KPC, complete sequence | *Klebsiella pneumoniae* | 57367 | 2.85E+05 | 90% | 0 | 99.98 | 89906 | 124760 | MT810372.1 |
|  | *Klebsiella pneumoniae* strain Kp36 plasmid unnamed2, complete sequence | *Klebsiella pneumoniae* | 57361 | 2.97E+05 | 90% | 0 | 99.98 | 89906 | 142228 | CP047194.1 |
|  | *Klebsiella pneumoniae* strain 33367 plasmid p33367_KPC2, complete sequence | *Klebsiella pneumoniae* | 57361 | 3.02E+05 | 90% | 0 | 99.98 | 89906 | 150096 | CP099415.1 |
|  | *Klebsiella pneumoniae* strain 7849 plasmid pKP7849_KPC, complete sequence | *Klebsiella pneumoniae* | 57361 | 2.68E+05 | 90% | 0 | 99.98 | 89906 | 148977 | MW478298.1 |
|  | *Klebsiella pneumoniae* strain CRKP78R plasmid p3, complete sequence | *Klebsiella pneumoniae* | 57361 | 3.11E+05 | 90% | 0 | 99.98 | 89906 | 149407 | CP066256.1 |
|  | *Klebsiella pneumoniae* *subsp.* pneumoniae strain SCKP020079 plasmid pKPC2_020079, complete sequence | *Klebsiella pneumoniae* *subsp.* pneumoniae | 57358 | 2.80E+05 | 90% | 0 | 99.98 | 89906 | 146790 | CP029381.1 |
|  | *Klebsiella pneumoniae* strain C789 plasmid pKPC-CR-hvKP-C789, complete sequence | *Klebsiella pneumoniae* | 57356 | 2.77E+05 | 90% | 0 | 99.97 | 89906 | 128299 | CP034417.1 |
|  | *Klebsiella pneumoniae* strain SWU01 plasmid unnamed, complete sequence | *Klebsiella pneumoniae* | 57356 | 3.09E+05 | 90% | 0 | 99.98 | 89906 | 162552 | CP018455.1 |
|  | *Klebsiella pneumoniae* strain JNKPN26 plasmid pJNKPN26_KPC, complete sequence | *Klebsiella pneumoniae* | 57352 | 2.60E+05 | 90% | 0 | 99.97 | 89906 | 126203 | MZ546615.1 |
|  | *Klebsiella pneumoniae* strain JNKPN26 plasmid pJNKPN26_KPC, complete sequence | *Klebsiella pneumoniae* | 57352 | 2.54E+05 | 90% | 0 | 99.97 | 89906 | 126203 | CP090204.1 |
|  | *Klebsiella pneumoniae* strain C2582 plasmid pRes_C2582 | *Klebsiella pneumoniae* | 57350 | 2.71E+05 | 90% | 0 | 99.97 | 89906 | 128399 | CP079210.1 |
|  | *Klebsiella pneumoniae* *subsp.* pneumoniae strain DD02341 plasmid pDD02341-1, complete sequence | *Klebsiella pneumoniae* *subsp.* pneumoniae | 57350 | 3.23E+05 | 89% | 0 | 99.97 | 89906 | 178812 | CP087630.1 |
|  | *Klebsiella pneumoniae* *subsp.* pneumoniae strain RJBSI76-pV plasmid pRJBSI76-pV-3, complete sequence | *Klebsiella pneumoniae* *subsp.* pneumoniae | 57374 | 2.71E+05 | 88% | 0 | 99.98 | 89906 | 184751 | CP068687.1 |
|  | *Klebsiella pneumoniae* *subsp.* pneumoniae strain RJBSI76 plasmid pRJBSI76-3, complete sequence | *Klebsiella pneumoniae* *subsp.* pneumoniae | 57374 | 2.71E+05 | 88% | 0 | 99.98 | 89906 | 184748 | CP068692.1 |
|  | *Klebsiella pneumoniae* strain KP137060 plasmid unnamed2, complete sequence | *Klebsiella pneumoniae* | 57358 | 2.34E+05 | 88% | 0 | 99.98 | 89906 | 146878 | MW218143.1 |
|  | *Klebsiella pneumoniae* strain WCHKP040035 plasmid pKPC2_040035, complete sequence | *Klebsiella pneumoniae* | 77772 | 2.62E+05 | 87% | 0 | 99.99 | 89906 | 112467 | CP028796.1 |
|  | *Klebsiella pneumoniae* strain WCHKP649 plasmid pKPC2_095649, complete sequence | *Klebsiella pneumoniae* | 57402 | 2.79E+05 | 86% | 0 | 100 | 89906 | 156099 | CP026584.1 |
|  | *Klebsiella pneumoniae* strain KPC-2 plasmid pKP169-P2, complete sequence | *Klebsiella pneumoniae* | 57400 | 2.68E+05 | 86% | 0 | 100 | 89906 | 157394 | CP078123.1 |
|  | *Klebsiella pneumoniae* strain C2660 plasmid pC2660-3-KPC, complete sequence | *Klebsiella pneumoniae* | 57402 | 2.59E+05 | 84% | 0 | 100 | 89906 | 153556 | CP039810.1 |
|  | *Klebsiella pneumoniae* *subsp.* pneumoniae strain WCHKP015093 plasmid pKPC2_015093, complete sequence | *Klebsiella pneumoniae* *subsp.* pneumoniae | 57402 | 2.82E+05 | 84% | 0 | 100 | 89906 | 154724 | CP036301.1 |
|  | *Klebsiella pneumoniae* strain FZKP4523 plasmid pKPC-2_FZKP4523, complete sequence | *Klebsiella pneumoniae* | 57402 | 2.86E+05 | 84% | 0 | 100 | 89906 | 141697 | CP101534.1 |
|  | *Klebsiella pneumoniae* strain 140253 plasmid pKPC2_140253, complete sequence | *Klebsiella pneumoniae* | 57402 | 2.47E+05 | 84% | 0 | 100 | 89906 | 155793 | CP097627.1 |
|  | *Klebsiella pneumoniae* strain WCHKP090050 plasmid pKPC2_090050, complete sequence | *Klebsiella pneumoniae* | 57402 | 2.71E+05 | 84% | 0 | 100 | 89906 | 154724 | CP043370.1 |
|  | *Klebsiella pneumoniae* strain WCHKP090045 plasmid pKPC2_090045, complete sequence | *Klebsiella pneumoniae* | 57402 | 2.71E+05 | 84% | 0 | 100 | 89906 | 154724 | CP043366.1 |
|  | *Klebsiella pneumoniae* strain WCHKP090374 plasmid pKPC2_090374, complete sequence | *Klebsiella pneumoniae* | 57402 | 2.85E+05 | 84% | 0 | 100 | 89906 | 154728 | CP066536.1 |
|  | *Klebsiella pneumoniae* *subsp.* pneumoniae strain WCHKP020120 plasmid pKPC2_020120, complete sequence | *Klebsiella pneumoniae* *subsp.* pneumoniae | 57402 | 2.79E+05 | 84% | 0 | 100 | 89906 | 154719 | CP043358.1 |
|  | *Klebsiella pneumoniae* strain WCHKP3 plasmid pKPC2_020003, complete sequence | *Klebsiella pneumoniae* | 57385 | 2.59E+05 | 84% | 0 | 99.99 | 89906 | 154957 | CP031720.1 |
|  | *Klebsiella pneumoniae* strain WCHKP115069 plasmid pKPC2_115069, complete sequence | *Klebsiella pneumoniae* | 57363 | 2.62E+05 | 84% | 0 | 99.98 | 89906 | 154986 | CP033404.1 |
|  | *Klebsiella pneumoniae* strain CY814036 plasmid pCY814036-KPC2, complete sequence | *Klebsiella pneumoniae* | 57363 | 2.73E+05 | 84% | 0 | 99.98 | 89906 | 140105 | CP093153.1 |
|  | *Klebsiella pneumoniae* strain KP19-2196 plasmid pKP19-2196-KPC2, complete sequence | *Klebsiella pneumoniae* | 58545 | 3.60E+05 | 83% | 0 | 99.97 | 89906 | 160563 | CP082042.1 |
|  | *Klebsiella pneumoniae* strain KP18-41 plasmid pKP18-41-KPC2, complete sequence | *Klebsiella pneumoniae* | 58545 | 3.42E+05 | 83% | 0 | 99.97 | 89906 | 153214 | CP082012.1 |
|  | *Klebsiella pneumoniae* strain KP18-2172 plasmid pKP18-2172-KPC2, complete sequence | *Klebsiella pneumoniae* | 58534 | 3.60E+05 | 83% | 0 | 99.97 | 89906 | 160747 | CP082039.1 |
|  | *Klebsiella pneumoniae* strain KP18-1 plasmid pKP18-1-KPC2, complete sequence | *Klebsiella pneumoniae* | 58534 | 3.42E+05 | 83% | 0 | 99.97 | 89906 | 153399 | CP082003.1 |
|  | *Klebsiella pneumoniae* strain WCHKP115038 plasmid pKPC2_115038, complete sequence | *Klebsiella pneumoniae* | 57402 | 2.69E+05 | 83% | 0 | 100 | 89906 | 153486 | CP043603.1 |
|  | *Klebsiella pneumoniae* strain 21091025 plasmid p21091025_1, complete sequence | *Klebsiella pneumoniae* | 72502 | 2.54E+05 | 82% | 0 | 99.98 | 89906 | 133771 | CP095265.1 |
|  | *Klebsiella pneumoniae* strain WCHKP115011 plasmid pKPC2_115011, complete sequence | *Klebsiella pneumoniae* | 57402 | 3.22E+05 | 80% | 0 | 100 | 89906 | 157388 | CP089954.1 |
|  | *Klebsiella pneumoniae* *subsp.* pneumoniae strain DD01653 plasmid pDD01653-2, complete sequence | *Klebsiella pneumoniae* *subsp.* pneumoniae | 57361 | 2.24E+05 | 80% | 0 | 99.98 | 89906 | 112393 | CP087653.1 |
|  | *Klebsiella pneumoniae* strain 2014042281 plasmid p42281-KPC, complete sequence | *Klebsiella pneumoniae* | 57361 | 2.75E+05 | 80% | 0 | 99.98 | 89906 | 115305 | MT810369.1 |
|  | *Klebsiella pneumoniae* *subsp.* pneumoniae strain DD01635 plasmid pDD01635-3, complete sequence | *Klebsiella pneumoniae* *subsp.* pneumoniae | 57356 | 2.39E+05 | 80% | 0 | 99.97 | 89906 | 112390 | CP087660.1 |
|  | *Klebsiella pneumoniae* strain HvKp-su1 plasmid unnamed1, complete sequence | *Klebsiella pneumoniae* | 57402 | 2.41E+05 | 79% | 0 | 100 | 89906 | 133346 | CP092718.1 |
|  | *Klebsiella pneumoniae* strain 9489 plasmid pBJ9489-KPC | *Klebsiella pneumoniae* | 57402 | 2.30E+05 | 79% | 0 | 100 | 89906 | 132246 | MN821371.1 |
|  | *Klebsiella pneumoniae* strain WCHKP020030 plasmid pKPC2_020030, complete sequence | *Klebsiella pneumoniae* | 57361 | 2.84E+05 | 79% | 0 | 99.98 | 89906 | 104810 | CP028790.2 |
|  | *Klebsiella pneumoniae* *subsp.* pneumoniae strain DD01845 plasmid pDD01845-3, complete sequence | *Klebsiella pneumoniae* *subsp.* pneumoniae | 57361 | 2.24E+05 | 79% | 0 | 99.98 | 89906 | 112224 | CP087666.1 |
|  | *Klebsiella pneumoniae* strain 381810-51 plasmid p181051-KPC, complete sequence | *Klebsiella pneumoniae* | 57358 | 2.13E+05 | 79% | 0 | 99.98 | 89906 | 303071 | MT920903.1 |
|  | *Klebsiella pneumoniae* strain BJ20 plasmid pBJ20-KPC, complete sequence | *Klebsiella pneumoniae* | 57354 | 3.06E+05 | 79% | 0 | 99.97 | 89906 | 97460 | MT108208.1 |
|  | *Klebsiella pneumoniae* strain 20150420184 plasmid p420184-KPC, complete sequence | *Klebsiella pneumoniae* | 57402 | 2.42E+05 | 78% | 0 | 100 | 89906 | 108236 | MT810359.1 |
|  | *Klebsiella pneumoniae* strain XHKPN083 plasmid pXHKPN083-2, complete sequence | *Klebsiella pneumoniae* | 57385 | 3.20E+05 | 78% | 0 | 99.99 | 89906 | 122553 | CP066912.1 |
|  | *Klebsiella pneumoniae* strain WSCRKP plasmid pWSCRKP-2, complete sequence | *Klebsiella pneumoniae* | 77739 | 2.47E+05 | 77% | 0 | 99.98 | 89906 | 103130 | CP091070.1 |
|  | *Klebsiella pneumoniae* strain JNKPN52 plasmid pJNKPN52_KPC_FOS | *Klebsiella pneumoniae* | 60702 | 2.57E+05 | 72% | 0 | 99.98 | 89906 | 279300 | MZ512197.1 |
|  | *Klebsiella pneumoniae* strain 12478 plasmid p12478-rmtB, complete sequence | *Klebsiella pneumoniae* | 57361 | 2.17E+05 | 67% | 0 | 99.98 | 89906 | 62594 | MN419308.1 |
|  | *Klebsiella pneumoniae* strain IR5077_1 plasmid unnamed3, complete sequence | *Klebsiella pneumoniae* | 57367 | 2.27E+05 | 64% | 0 | 99.98 | 89906 | 292919 | CP097673.1 |
|  | *Klebsiella pneumoniae* strain KP55 plasmid pKPC-5502, complete sequence | *Klebsiella pneumoniae* | 57358 | 2.17E+05 | 64% | 0 | 99.97 | 89906 | 100658 | OL891652.1 |
|  | *Klebsiella pneumoniae* strain XHKPN391 chromosome, complete genome | *Klebsiella pneumoniae* | 57387 | 3.74E+05 | 55% | 0 | 99.99 | 89906 | 5633245 | CP066915.1 |
|  | Citrobacter werkmanii strain LYYSPS2 plasmid pLYYSPS2-3, complete sequence | Citrobacter werkmanii | 57400 | 1.43E+05 | 54% | 0 | 100 | 89906 | 87232 | MZ342958.1 |
|  | *Escherichia coli* strain HNEC55 plasmid pHNEC55, complete sequence | *Escherichia coli* | 57361 | 1.44E+05 | 54% | 0 | 99.98 | 89906 | 81498 | KT879914.1 |
| pCR-hvKP26-P2 | *Klebsiella pneumoniae* strain IR5726 plasmid unnamed2, complete sequence | *Klebsiella pneumoniae* | 39451 | 1.89E+05 | 98% | 0 | 98.25 | 54515 | 110359 | CP061959.1 |
|  | *Klebsiella pneumoniae* strain IR12024_1 plasmid unnamed1 | *Klebsiella pneumoniae* | 39451 | 1.79E+05 | 98% | 0 | 98.25 | 54515 | 103399 | CP097676.1 |
|  | *Klebsiella pneumoniae* strain IR5755 plasmid unnamed1, complete sequence | *Klebsiella pneumoniae* | 39440 | 1.77E+05 | 98% | 0 | 98.24 | 54515 | 103224 | CP061971.1 |
|  | *Klebsiella pneumoniae* strain KP18-238 plasmid pKP18-238-2, complete sequence | *Klebsiella pneumoniae* | 29713 | 1.77E+05 | 95% | 0 | 98.85 | 54515 | 107713 | CP082016.1 |
|  | *Klebsiella pneumoniae* strain IR12197_1 plasmid unnamed3 | *Klebsiella pneumoniae* | 39451 | 1.69E+05 | 98% | 0 | 98.25 | 54515 | 96993 | CP097706.1 |
|  | *Klebsiella pneumoniae* *subsp.* pneumoniae strain kpn-hnqyy plasmid unnamed1, complete sequence | *Klebsiella pneumoniae* *subsp.* pneumoniae | 31194 | 1.38E+05 | 89% | 0 | 99.86 | 54515 | 123557 | CP074117.1 |
|  | *Klebsiella pneumoniae* strain KP47693 plasmid p47693_2, complete sequence | *Klebsiella pneumoniae* | 25176 | 1.13E+05 | 85% | 0 | 97.78 | 54515 | 81467 | CP070529.1 |
|  | *Klebsiella pneumoniae* strain KPA9853 plasmid pA9853_2, complete sequence | *Klebsiella pneumoniae* | 25176 | 1.03E+05 | 85% | 0 | 97.78 | 54515 | 75571 | CP070600.1 |
|  | *Klebsiella pneumoniae* strain WCHKP040035 plasmid p1_040035, complete sequence | *Klebsiella pneumoniae* | 42529 | 97811 | 99% | 0 | 99.17 | 54515 | 54847 | CP028794.1 |
|  | *Klebsiella pneumoniae* strain KP18-2079 plasmid pKP18-2079_54kb, complete sequence | *Klebsiella pneumoniae* | 39776 | 95312 | 98% | 0 | 98.49 | 54515 | 54881 | MT090961.1 |
|  | *Klebsiella pneumoniae* strain WCHKP090361 plasmid p1_090361, complete sequence | *Klebsiella pneumoniae* | 26515 | 95187 | 98% | 0 | 98.88 | 54515 | 55161 | CP066531.1 |
|  | *Klebsiella pneumoniae* strain WCHKP090329 plasmid p1_090329, complete sequence | *Klebsiella pneumoniae* | 26515 | 95187 | 98% | 0 | 98.87 | 54515 | 55161 | CP066520.1 |
| pCR-hvKP26-P3 | *Klebsiella pneumoniae* strain KP0079 plasmid pKP0079-4, complete sequence |  | 22099 | 44201 | 100% | 0 | 99.99 | 11970 | 23940 | CP102840.1 |
|  | *Klebsiella pneumoniae* strain 36 plasmid pKP36_5, complete sequence | *Klebsiella pneumoniae* | 22083 | 22083 | 99% | 0 | 100 | 11970 | 11970 | CP082763.1 |
|  | *Klebsiella pneumoniae* strain CRKP66R plasmid pCRKP66R-5, complete sequence | *Klebsiella pneumoniae* | 22077 | 22077 | 99% | 0 | 100 | 11970 | 11970 | CP063837.1 |
|  | *Klebsiella pneumoniae* strain KP20194c plasmid pKP20194c-p4, complete sequence | *Klebsiella pneumoniae* | 22075 | 22075 | 99% | 0 | 99.99 | 11970 | 11970 | CP054760.1 |
|  | *Klebsiella pneumoniae* strain KP20194a plasmid pKP20194a-p4, complete sequence | *Klebsiella pneumoniae* | 22074 | 22074 | 99% | 0 | 99.99 | 11970 | 11970 | CP054784.1 |
|  | *Klebsiella pneumoniae* strain C2414 plasmid pC2414-4, complete sequence | *Klebsiella pneumoniae* | 22072 | 22072 | 99% | 0 | 100 | 11970 | 11970 | CP039822.1 |
|  | *Klebsiella pneumoniae* *subsp.* pneumoniae strain DD01635 plasmid pDD01635-4, complete sequence | *Klebsiella pneumoniae* *subsp.* pneumoniae | 22064 | 22064 | 99% | 0 | 100 | 11970 | 11970 | CP087661.1 |
|  | *Klebsiella pneumoniae* strain C2660 plasmid pC2660-5, complete sequence | *Klebsiella pneumoniae* | 22061 | 22061 | 99% | 0 | 99.99 | 11970 | 11970 | CP039812.1 |
|  | *Klebsiella pneumoniae* strain 21080937 plasmid p21080937_6, complete sequence | *Klebsiella pneumoniae* | 22061 | 22061 | 99% | 0 | 100 | 11970 | 11970 | CP095259.1 |
|  | *Klebsiella pneumoniae* *subsp.* pneumoniae strain DD01845 plasmid pDD01845-4, complete sequence | *Klebsiella pneumoniae* *subsp.* pneumoniae | 22053 | 22053 | 99% | 0 | 99.99 | 11970 | 11970 | CP087667.1 |
|  | *Klebsiella pneumoniae* strain 8695 plasmid p4, complete sequence | *Klebsiella pneumoniae* | 22044 | 44121 | 100% | 0 | 99.89 | 11970 | 23957 | CP085893.1 |
|  | *Klebsiella pneumoniae* strain CDI694 plasmid pCDI694-12.0, complete sequence | *Klebsiella pneumoniae* | 21861 | 21861 | 99% | 0 | 99.67 | 11970 | 11972 | CP077775.1 |
|  | *Klebsiella pneumoniae* strain 21091025 plasmid p21091025_3, complete sequence | *Klebsiella pneumoniae* | 21608 | 43319 | 100% | 0 | 99.27 | 11970 | 23759 | CP095267.1 |
|  | *Escherichia coli* strain CR-HvKP5TC-5 plasmid pCR-HvKP5TC-5_p4, complete sequence | *Escherichia coli* | 20753 | 22106 | 100% | 0 | 100 | 11970 | 11970 | OM001479.1 |
|  | *Klebsiella pneumoniae* strain KPWX136 plasmid pE, complete sequence | *Klebsiella pneumoniae* | 20751 | 22101 | 100% | 0 | 99.99 | 11970 | 11970 | CP069174.1 |
|  | *Klebsiella pneumoniae* strain Kp36 plasmid unnamed4, complete sequence | *Klebsiella pneumoniae* | 19908 | 22074 | 100% | 0 | 99.86 | 11970 | 12001 | CP047196.1 |
|  | *Klebsiella pneumoniae* strain KP20194f plasmid pKP20194f-p4, complete sequence | *Klebsiella pneumoniae* | 19645 | 22101 | 100% | 0 | 99.99 | 11970 | 11970 | CP054724.1 |
|  | *Klebsiella pneumoniae* *subsp.* pneumoniae strain DD01653 plasmid pDD01653-4, complete sequence | *Klebsiella pneumoniae* *subsp.* pneumoniae | 19642 | 22095 | 100% | 0 | 99.98 | 11970 | 11970 | CP087655.1 |
|  | *Klebsiella pneumoniae* strain KP58 plasmid pKP58-4, complete sequence | *Klebsiella pneumoniae* | 19396 | 22101 | 100% | 0 | 99.99 | 11970 | 11970 | CP041377.1 |
|  | *Klebsiella pneumoniae* *subsp.* pneumoniae strain DD02391 plasmid pDD02391-4, complete sequence | *Klebsiella pneumoniae* *subsp.* pneumoniae | 19014 | 21724 | 100% | 0 | 99.36 | 11970 | 11959 | CP087643.1 |
|  | *Klebsiella pneumoniae* strain XHKP502 plasmid pXHKP502-4, complete sequence | *Klebsiella pneumoniae* | 18766 | 22095 | 100% | 0 | 99.98 | 11970 | 11971 | CP066908.1 |
|  | *Klebsiella pneumoniae* strain WCHKP090361 plasmid p2_090361, complete sequence | *Klebsiella pneumoniae* | 18349 | 22106 | 100% | 0 | 100 | 11970 | 11970 | CP066532.1 |
|  | *Klebsiella pneumoniae* strain KP18-2079 plasmid pKP18-2079_11kb, complete sequence | *Klebsiella pneumoniae* | 18329 | 22101 | 100% | 0 | 99.99 | 11970 | 11970 | MT090962.1 |
|  | *Klebsiella pneumoniae* strain 9949 plasmid unnamed4, complete sequence | *Klebsiella pneumoniae* | 18144 | 29934 | 100% | 0 | 99.97 | 11970 | 16223 | CP050284.1 |
|  | *Klebsiella pneumoniae* strain 37 plasmid pKP37_5, complete sequence | *Klebsiella pneumoniae* | 18066 | 22093 | 100% | 0 | 99.98 | 11970 | 11969 | CP082757.1 |
|  | *Klebsiella pneumoniae* strain FK 6768 plasmid unnamed4, complete sequence | *Klebsiella pneumoniae* | 17516 | 21957 | 100% | 0 | 99.68 | 11970 | 12001 | CP065558.1 |
|  | *Klebsiella pneumoniae* strain KP1064WHY plasmid p4, complete sequence | *Klebsiella pneumoniae* | 17256 | 22008 | 100% | 0 | 99.82 | 11970 | 11972 | CP084709.1 |
|  | *Escherichia coli* strain CR-HvKP3TC plasmid pCR-HvKP3TC_p4, complete sequence | *Escherichia coli* | 16982 | 22106 | 100% | 0 | 100 | 11970 | 11970 | MW598243.1 |
|  | *Klebsiella pneumoniae* strain CR-HvKP3 plasmid p4-CR-HvKP3, complete sequence | *Klebsiella pneumoniae* | 16982 | 22106 | 100% | 0 | 100 | 11970 | 11970 | MW598236.1 |
|  | *Klebsiella pneumoniae* strain KP697 plasmid unnamed4, complete sequence | *Klebsiella pneumoniae* | 16973 | 22101 | 100% | 0 | 99.99 | 11970 | 11970 | CP066155.1 |
|  | *Klebsiella pneumoniae* strain KP18-2113 plasmid pKP18-2113-3, complete sequence | *Klebsiella pneumoniae* | 16957 | 22088 | 100% | 0 | 99.97 | 11970 | 11969 | CP082028.1 |
|  | *Klebsiella pneumoniae* strain KP20194c4 plasmid pKP20194c4-p4, complete sequence | *Klebsiella pneumoniae* | 16842 | 21979 | 100% | 0 | 99.75 | 11970 | 11971 | CP054748.1 |
|  | *Klebsiella pneumoniae* strain KP20194b2 plasmid pKP20194b2-p4, complete sequence | *Klebsiella pneumoniae* | 16809 | 22101 | 100% | 0 | 99.99 | 11970 | 11970 | CP054766.1 |
|  | *Klebsiella pneumoniae* strain KP200731214 plasmid pKP-5 | *Klebsiella pneumoniae* | 16720 | 22106 | 100% | 0 | 100 | 11970 | 11970 | CP084748.1 |
|  | *Klebsiella pneumoniae* strain KP18-238 plasmid pKP18-238-3, complete sequence | *Klebsiella pneumoniae* | 16687 | 21724 | 100% | 0 | 99.28 | 11970 | 11959 | CP082013.1 |
|  | *Klebsiella pneumoniae* strain 12 plasmid pKP12_5, complete sequence | *Klebsiella pneumoniae* | 15653 | 22084 | 100% | 0 | 100 | 11970 | 11970 | CP082769.1 |
|  | *Escherichia coli* strain CR-HvKP4TC plasmid pCR-HvKP4TC_Vir-p4, complete sequence | *Escherichia coli* | 15034 | 25688 | 100% | 0 | 100 | 11970 | 190130 | MW598245.1 |
|  | *Escherichia coli* strain CR-HvKP4TC-2 plasmid pCR-HvKP4TC-2_p4, complete sequence | *Escherichia coli* | 14994 | 22106 | 100% | 0 | 100 | 11970 | 11970 | OM001477.1 |
|  | *Klebsiella pneumoniae* strain KP1517 plasmid unnamed3, complete sequence | *Klebsiella pneumoniae* | 14922 | 22106 | 100% | 0 | 100 | 11970 | 11970 | CP072466.1 |
|  | *Klebsiella pneumoniae* strain LZKP00003 plasmid pZR1, complete sequence | *Klebsiella pneumoniae* | 14698 | 22106 | 100% | 0 | 100 | 11970 | 11970 | CP089992.1 |
|  | *Klebsiella pneumoniae* strain FDAARGOS_443 plasmid unnamed4, complete sequence | *Klebsiella pneumoniae* | 14683 | 22097 | 100% | 0 | 99.97 | 11970 | 11972 | CP023932.1 |
|  | *Klebsiella pneumoniae* strain JX-CR-hvKP-3 plasmid pJX3-4, complete sequence | *Klebsiella pneumoniae* | 14534 | 22106 | 100% | 0 | 100 | 11970 | 11970 | CP064245.1 |
|  | *Klebsiella pneumoniae* strain KP20194a2 plasmid pKP20194a2-p4, complete sequence | *Klebsiella pneumoniae* | 14314 | 22046 | 100% | 0 | 99.83 | 11970 | 15931 | CP054778.1 |
|  | *Klebsiella pneumoniae* strain KP55 plasmid pKP55_5, complete sequence | *Klebsiella pneumoniae* | 14299 | 22106 | 100% | 0 | 100 | 11970 | 11970 | CP055299.1 |
|  | *Klebsiella pneumoniae* strain L482 plasmid p5_L382, complete sequence | *Klebsiella pneumoniae* | 14296 | 22106 | 100% | 0 | 100 | 11970 | 11970 | CP033964.1 |
|  | *Klebsiella pneumoniae* strain SCKP020009 plasmid p1_020009, complete sequence | *Klebsiella pneumoniae* | 14283 | 22106 | 100% | 0 | 100 | 11970 | 11970 | CP038005.1 |
|  | *Klebsiella pneumoniae* strain L39_2 plasmid p5_L39, complete sequence | *Klebsiella pneumoniae* | 14277 | 22101 | 100% | 0 | 99.99 | 11970 | 11970 | CP033958.1 |
|  | *Klebsiella pneumoniae* strain KP16 plasmid unnamed4, complete sequence | *Klebsiella pneumoniae* | 14277 | 22101 | 100% | 0 | 99.99 | 11970 | 11970 | CP087150.1 |
|  | *Klebsiella pneumoniae* strain KP14 plasmid unnamed4, complete sequence | *Klebsiella pneumoniae* | 14277 | 22101 | 100% | 0 | 99.99 | 11970 | 11970 | CP087155.1 |
|  | *Klebsiella pneumoniae* strain WSCRKP plasmid pWSCRKP-4, complete sequence | *Klebsiella pneumoniae* | 14235 | 22115 | 100% | 0 | 99.99 | 11970 | 15931 | CP091072.1 |
|  | *Klebsiella pneumoniae* strain KP15 plasmid unnamed3, complete sequence | *Klebsiella pneumoniae* | 14044 | 25657 | 100% | 0 | 99.97 | 11970 | 99065 | CP087145.1 |
|  | *Klebsiella pneumoniae* strain hvKP323 plasmid unnamed3, complete sequence | *Klebsiella pneumoniae* | 14039 | 22106 | 100% | 0 | 100 | 11970 | 11970 | CP101773.1 |
|  | *Klebsiella pneumoniae* strain XH1507 plasmid pXH1507-5, complete sequence | *Klebsiella pneumoniae* | 14039 | 22106 | 100% | 0 | 100 | 11970 | 11970 | CP092798.1 |
|  | *Klebsiella pneumoniae* strain CRKP52R plasmid pCRKP52R-4-tetA, complete sequence | *Klebsiella pneumoniae* | 14028 | 25641 | 100% | 0 | 99.93 | 11970 | 99066 | CP066252.1 |
|  | *Escherichia coli* strain CR-HvKP5TC plasmid pCR-HvKP5TC_Vir-p4, complete sequence | *Escherichia coli* | 14026 | 25646 | 100% | 0 | 99.93 | 11970 | 190129 | MW598247.1 |
|  | *Escherichia coli* strain CR-HvKP1TC plasmid pCR-HvKP1TC_Vir-p4, complete sequence | *Escherichia coli* | 14026 | 25646 | 100% | 0 | 99.93 | 11970 | 190129 | MW598240.1 |
|  | *Klebsiella pneumoniae* strain BSI058 plasmid pBSI058-KPC2 | *Klebsiella pneumoniae* | 14024 | 25644 | 100% | 0 | 99.91 | 11970 | 114712 | MT269836.1 |
|  | *Klebsiella pneumoniae* strain 1864 plasmid p1864-4, complete sequence | *Klebsiella pneumoniae* | 14020 | 22106 | 100% | 0 | 100 | 11970 | 11970 | CP084496.1 |
|  | *Klebsiella pneumoniae* strain KP-CT77 plasmid pCT77-tetA, complete sequence | *Klebsiella pneumoniae* | 14007 | 25620 | 100% | 0 | 99.88 | 11970 | 92645 | CP080306.1 |
|  | *Klebsiella pneumoniae* strain WCHKP115068 plasmid p2_115068, complete sequence | *Klebsiella pneumoniae* | 13825 | 22121 | 100% | 0 | 100 | 11970 | 12746 | CP036369.1 |
|  | *Klebsiella pneumoniae* strain WCHKP020098 plasmid p2_020098, complete sequence | *Klebsiella pneumoniae* | 13444 | 22106 | 100% | 0 | 100 | 11970 | 11970 | CP036308.1 |
|  | *Klebsiella pneumoniae* strain JX-CR-hvKP-1 plasmid pJX1-4, complete sequence | *Klebsiella pneumoniae* | 13345 | 22106 | 100% | 0 | 100 | 11970 | 11970 | CP064256.1 |
|  | *Klebsiella pneumoniae* JM45 plasmid p2, complete sequence | *Klebsiella pneumoniae* JM45 | 13274 | 16372 | 80% | 0 | 97.22 | 11970 | 12207 | CP006658.1 |
|  | *Klebsiella pneumoniae* isolate 307 genome assembly, plasmid: P3 | *Klebsiella pneumoniae* | 12912 | 19526 | 80% | 0 | 97.31 | 11970 | 34959 | OW967853.1 |
|  | *Klebsiella pneumoniae* isolate 307 genome assembly, plasmid: P3 | *Klebsiella pneumoniae* | 12912 | 19526 | 80% | 0 | 97.31 | 11970 | 34959 | OW967206.1 |
|  | *Klebsiella pneumoniae* isolate 307 genome assembly, plasmid: P3 | *Klebsiella pneumoniae* | 12912 | 19526 | 80% | 0 | 97.31 | 11970 | 34959 | OW849062.1 |
|  | *Klebsiella pneumoniae* strain RIVM_C019006 plasmid pRIVM_C019006_3, complete sequence | *Klebsiella pneumoniae* | 12912 | 19522 | 80% | 0 | 97.3 | 11970 | 34959 | CP068923.1 |
|  | *Klebsiella pneumoniae* strain WCHKP090329 plasmid p2_090329, complete sequence | *Klebsiella pneumoniae* | 12912 | 22106 | 100% | 0 | 100 | 11970 | 11970 | CP066521.1 |
|  | *Klebsiella pneumoniae* strain DD521 plasmid pDD521.4, complete sequence | *Klebsiella pneumoniae* | 12896 | 22088 | 100% | 0 | 99.97 | 11970 | 11969 | CP075320.1 |
|  | *Klebsiella pneumoniae* strain IR12243_1 plasmid unnamed1, complete sequence | *Klebsiella pneumoniae* | 12700 | 22124 | 100% | 0 | 99.99 | 11970 | 12798 | CP097661.1 |
|  | *Klebsiella pneumoniae* strain K64 plasmid pColRNAI-5, complete sequence | *Klebsiella pneumoniae* | 12626 | 20693 | 99% | 0 | 96.8 | 11970 | 11934 | CP102395.1 |
|  | *Klebsiella pneumoniae* strain S234 plasmid pS234-3, complete sequence | *Klebsiella pneumoniae* | 12183 | 22101 | 100% | 0 | 99.98 | 11970 | 11970 | CP102189.1 |
|  | *Klebsiella pneumoniae* strain L491 plasmid p4-L491 | *Klebsiella pneumoniae* | 11875 | 22106 | 100% | 0 | 100 | 11970 | 11970 | CP029229.1 |
|  | *Klebsiella pneumoniae* strain L388 plasmid p5-L388 | *Klebsiella pneumoniae* | 11869 | 22101 | 100% | 0 | 99.98 | 11970 | 11970 | CP029224.1 |
|  | *Klebsiella pneumoniae* *subsp.* pneumoniae strain KPN857 plasmid pD, complete sequence | *Klebsiella pneumoniae* *subsp.* pneumoniae | 11854 | 22101 | 100% | 0 | 99.98 | 11970 | 11970 | CP090436.1 |
|  | *Klebsiella pneumoniae* strain JX-CR-hvKP-4 plasmid pJX4-4, complete sequence | *Klebsiella pneumoniae* | 11701 | 22106 | 100% | 0 | 100 | 11970 | 11970 | CP064239.1 |
|  | *Escherichia coli* strain CR-HvKP1TC-3 plasmid pCR-HvKP1TC-3_p4, complete sequence | *Escherichia coli* | 11620 | 22106 | 100% | 0 | 100 | 11970 | 11970 | OM001473.1 |
|  | *Klebsiella pneumoniae* strain WCHKP36 plasmid p2_020036, complete sequence | *Klebsiella pneumoniae* | 11616 | 22122 | 100% | 0 | 100 | 11970 | 12746 | CP028580.2 |
|  | *Klebsiella pneumoniae* strain hvKP340 plasmid unnamed5, complete sequence | *Klebsiella pneumoniae* | 11616 | 22106 | 100% | 0 | 100 | 11970 | 11970 | CP101781.1 |
|  | *Klebsiella pneumoniae* strain hvKP841 plasmid unnamed3, complete sequence | *Klebsiella pneumoniae* | 11616 | 22106 | 100% | 0 | 100 | 11970 | 11970 | CP101787.1 |
|  | *Klebsiella pneumoniae* strain 19PDR22 plasmid p7, complete sequence | *Klebsiella pneumoniae* | 11616 | 22106 | 100% | 0 | 100 | 11970 | 11970 | CP076551.1 |
|  | *Klebsiella pneumoniae* strain S270v plasmid pS270V-3, complete sequence | *Klebsiella pneumoniae* | 11611 | 22101 | 100% | 0 | 99.98 | 11970 | 11970 | CP102195.1 |
|  | *Klebsiella pneumoniae* strain hvKP319 plasmid unnamed4, complete sequence | *Klebsiella pneumoniae* | 11611 | 22101 | 100% | 0 | 99.98 | 11970 | 11970 | CP101768.1 |
|  | *Klebsiella pneumoniae* strain FRPDR plasmid pFRPDR_5, complete sequence | *Klebsiella pneumoniae* | 11611 | 22101 | 100% | 0 | 99.98 | 11970 | 11970 | CP063764.1 |
|  | *Klebsiella pneumoniae* strain CR-HvKP4 plasmid pCR-HvKP4-p4, complete sequence | *Klebsiella pneumoniae* | 11548 | 22106 | 100% | 0 | 100 | 11970 | 11970 | CP040543.1 |
|  | *Klebsiella pneumoniae* strain CR-HvKP5 plasmid pCR-HvKP5-p4, complete sequence | *Klebsiella pneumoniae* | 11527 | 22106 | 100% | 0 | 100 | 11970 | 11970 | CP040549.1 |
|  | *Klebsiella pneumoniae* strain CR-HvKP1 plasmid pCR-HvKP1-p4, complete sequence | *Klebsiella pneumoniae* | 11514 | 22106 | 100% | 0 | 100 | 11970 | 11970 | CP040537.1 |
|  | *Klebsiella pneumoniae* strain 50700 plasmid p50700-12.0, complete sequence | *Klebsiella pneumoniae* | 11420 | 21920 | 100% | 0 | 99.46 | 11970 | 11972 | CP088993.1 |
|  | *Klebsiella pneumoniae* isolate 991178e0-b809-11e8-aae5-3c4a9275d6c8 genome assembly, chromosome: 1 | *Klebsiella pneumoniae* | 10707 | 24599 | 98% | 0 | 100 | 11970 | 5570095 | LR596809.1 |
|  | *Klebsiella pneumoniae* isolate 98fb0f42-b809-11e8-aae5-3c4a9275d6c8 genome assembly, chromosome: 1 | *Klebsiella pneumoniae* | 10707 | 25085 | 93% | 0 | 100 | 11970 | 5716474 | LR596807.1 |
|  | *Klebsiella pneumoniae* *subsp.* pneumoniae strain RJBSI76 plasmid pRJBSI76-4, complete sequence | *Klebsiella pneumoniae* *subsp.* pneumoniae | 10623 | 18843 | 85% | 0 | 99.98 | 11970 | 10206 | CP068693.1 |
|  | *Klebsiella pneumoniae* isolate 15 genome assembly, plasmid: P2 | *Klebsiella pneumoniae* | 10495 | 15990 | 80% | 0 | 96.82 | 11970 | 11955 | OW969695.1 |
|  | *Klebsiella pneumoniae* strain 150040X1B1 plasmid p1_150040X1B1, complete sequence | *Klebsiella pneumoniae* | 10490 | 18580 | 84% | 0 | 100 | 11970 | 10060 | CP101729.1 |
|  | *Klebsiella pneumoniae* *subsp.* pneumoniae strain KP29 plasmid p29_3, complete sequence | *Klebsiella pneumoniae* *subsp.* pneumoniae | 10490 | 18580 | 84% | 0 | 100 | 11970 | 10060 | CP101570.1 |
|  | *Klebsiella pneumoniae* strain FZKP4523 plasmid p1_FZKP4523, complete sequence | *Klebsiella pneumoniae* | 10490 | 18580 | 84% | 0 | 100 | 11970 | 10060 | CP101535.1 |
|  | *Klebsiella pneumoniae* strain XHKP75 plasmid pXHKP75-3, complete sequence | *Klebsiella pneumoniae* | 10490 | 18580 | 84% | 0 | 100 | 11970 | 10060 | CP066898.1 |
|  | *Klebsiella pneumoniae* strain XHKPN391 plasmid pXHKPN391-2, complete sequence | *Klebsiella pneumoniae* | 10490 | 18579 | 84% | 0 | 100 | 11970 | 10060 | CP066917.1 |
|  | *Klebsiella pneumoniae* strain HvKp-su1 plasmid unnamed2, complete sequence | *Klebsiella pneumoniae* | 10490 | 18582 | 84% | 0 | 100 | 11970 | 10061 | CP092719.1 |
|  | *Klebsiella pneumoniae* *subsp.* pneumoniae strain DD02280 plasmid pDD02280-3 | *Klebsiella pneumoniae* *subsp.* pneumoniae | 10490 | 18568 | 83% | 0 | 100 | 11970 | 91826 | CP087626.1 |
|  | *Klebsiella pneumoniae* *subsp.* pneumoniae strain DD02172 plasmid pDD02172-5, complete sequence | *Klebsiella pneumoniae* *subsp.* pneumoniae | 10490 | 18580 | 84% | 0 | 100 | 11970 | 10060 | CP087616.1 |
| pCR-hvKP26-P4 | *Klebsiella pneumoniae* strain KP18-238 plasmid pKP18-238-4, complete sequence | *Klebsiella pneumoniae* | 10334 | 20671 | 100% | 0 | 100 | 5596 | 11192 | CP082017.1 |
|  | *Klebsiella pneumoniae* strain KP20194c5 plasmid pKP20194c5-p5, complete sequence | *Klebsiella pneumoniae* | 10325 | 10325 | 99% | 0 | 99.98 | 5596 | 5596 | CP054743.1 |
|  | *Klebsiella pneumoniae* strain 150040X1B1 plasmid p2_150040X1B1, complete sequence | *Klebsiella pneumoniae* | 10296 | 10296 | 99% | 0 | 100 | 5596 | 5596 | CP101730.1 |
|  | *Klebsiella pneumoniae* *subsp.* pneumoniae strain DD02280 plasmid pDD02280-4, complete sequence | *Klebsiella pneumoniae* *subsp.* pneumoniae | 10257 | 10336 | 100% | 0 | 100 | 5596 | 5596 | CP087627.1 |
|  | *Klebsiella pneumoniae* strain RIVM_C014947 plasmid pRIVM_C014947_4 | *Klebsiella pneumoniae* | 10168 | 10325 | 100% | 0 | 99.96 | 5596 | 5596 | MT560067.1 |
|  | *Klebsiella pneumoniae* *subsp.* pneumoniae strain SCKP020079 plasmid p2_020079, complete sequence | *Klebsiella pneumoniae* *subsp.* pneumoniae | 10126 | 10336 | 100% | 0 | 100 | 5596 | 5596 | CP029380.1 |
|  | *Klebsiella pneumoniae* strain WCHKP040035 plasmid p2_040035, complete sequence | *Klebsiella pneumoniae* | 10124 | 10336 | 100% | 0 | 100 | 5596 | 5596 | CP028795.2 |
|  | *Klebsiella pneumoniae* isolate 392 genome assembly, plasmid: P4 | *Klebsiella pneumoniae* | 10109 | 10304 | 99% | 0 | 99.91 | 5596 | 5599 | OW848883.1 |
|  | *Klebsiella pneumoniae* isolate 392 genome assembly, plasmid: P3 | *Klebsiella pneumoniae* | 10109 | 10304 | 99% | 0 | 99.91 | 5596 | 5599 | OW849089.1 |
|  | *Klebsiella pneumoniae* strain KP20194a plasmid pKP20194a-p5, complete sequence | *Klebsiella pneumoniae* | 10098 | 10330 | 100% | 0 | 99.98 | 5596 | 5596 | CP054785.1 |
|  | *Klebsiella pneumoniae* *subsp.* pneumoniae strain WCHKP020120 plasmid p3_020120, complete sequence | *Klebsiella pneumoniae* *subsp.* pneumoniae | 10067 | 10336 | 100% | 0 | 100 | 5596 | 5596 | CP043361.1 |
|  | *Klebsiella pneumoniae* strain JX-CR-hvKP-10 plasmid pJX10-4, complete sequence | *Klebsiella pneumoniae* | 10046 | 10336 | 100% | 0 | 100 | 5596 | 5596 | CP064262.1 |
|  | *Klebsiella pneumoniae* isolate 392 genome assembly, plasmid: P4 | *Klebsiella pneumoniae* | 9963 | 10304 | 99% | 0 | 99.91 | 5596 | 5599 | OW849049.1 |
|  | *Klebsiella pneumoniae* strain ZRKP01 plasmid unnamed5, complete sequence | *Klebsiella pneumoniae* | 9910 | 10336 | 100% | 0 | 100 | 5596 | 5596 | CP050359.1 |
|  | *Klebsiella pneumoniae* strain KPWX136 plasmid pF, complete sequence | *Klebsiella pneumoniae* | 9878 | 10336 | 100% | 0 | 100 | 5596 | 5596 | CP069175.1 |
|  | *Klebsiella pneumoniae* strain KP20194a2 plasmid pKP20194a2-p5, complete sequence | *Klebsiella pneumoniae* | 9860 | 10330 | 100% | 0 | 99.98 | 5596 | 5596 | CP054779.1 |
|  | *Klebsiella pneumoniae* strain WCHKP8F4 plasmid p2_095084, complete sequence | *Klebsiella pneumoniae* | 9808 | 10336 | 100% | 0 | 100 | 5596 | 5596 | CP027066.3 |
|  | *Klebsiella pneumoniae* strain WCHKP090045 plasmid p2_090045, complete sequence | *Klebsiella pneumoniae* | 9801 | 10336 | 100% | 0 | 100 | 5596 | 5596 | CP043368.1 |
|  | *Klebsiella pneumoniae* strain WCHKP090374 plasmid p2_090374, complete sequence | *Klebsiella pneumoniae* | 9792 | 10330 | 99% | 0 | 100 | 5596 | 5595 | CP066538.1 |
|  | *Klebsiella pneumoniae* strain WCHKP020115 plasmid p2_020115, complete sequence | *Klebsiella pneumoniae* | 9788 | 10336 | 100% | 0 | 100 | 5596 | 5596 | CP043356.1 |
|  | *Klebsiella pneumoniae* isolate 91eed288-b809-11e8-aae5-3c4a9275d6c8 genome assembly, chromosome: 1 | *Klebsiella pneumoniae* | 9716 | 10761 | 100% | 0 | 99.98 | 5596 | 5510473 | LR596808.1 |
|  | *Klebsiella pneumoniae* strain SH12 plasmid pSH12_5, complete sequence | *Klebsiella pneumoniae* | 9716 | 10336 | 100% | 0 | 100 | 5596 | 5596 | CP040838.1 |
|  | *Klebsiella pneumoniae* strain CP19 plasmid unnamed2, complete sequence | *Klebsiella pneumoniae* | 9679 | 10330 | 100% | 0 | 99.98 | 5596 | 5597 | CP073353.1 |
|  | *Klebsiella pneumoniae* strain FK 6768 plasmid unnamed5, complete sequence | *Klebsiella pneumoniae* | 9481 | 10336 | 100% | 0 | 100 | 5596 | 5596 | CP065559.1 |
|  | *Klebsiella pneumoniae* strain hvKP841 plasmid unnamed5, complete sequence | *Klebsiella pneumoniae* | 9465 | 10336 | 100% | 0 | 100 | 5596 | 5596 | CP101789.1 |
|  | *Klebsiella pneumoniae* strain KP20194c3 plasmid pKP20194c3-p5, complete sequence | *Klebsiella pneumoniae* | 9313 | 10330 | 100% | 0 | 99.98 | 5596 | 5596 | CP054755.1 |
|  | *Klebsiella pneumoniae* strain WCHKP020037 plasmid p3_020037, complete sequence | *Klebsiella pneumoniae* | 9274 | 10336 | 100% | 0 | 100 | 5596 | 5596 | CP036375.1 |
|  | *Klebsiella pneumoniae* strain WCHKP3 plasmid p3_020003, complete sequence | *Klebsiella pneumoniae* | 9254 | 10336 | 100% | 0 | 100 | 5596 | 5596 | CP031719.1 |
|  | *Klebsiella pneumoniae* strain WCHKP090357 plasmid p3_090357, complete sequence | *Klebsiella pneumoniae* | 9208 | 10336 | 100% | 0 | 100 | 5596 | 5596 | CP066527.1 |
|  | *Klebsiella pneumoniae* strain 21080937 plasmid p21080937_9, complete sequence | *Klebsiella pneumoniae* | 9143 | 10336 | 100% | 0 | 100 | 5596 | 5596 | CP095262.1 |
|  | *Klebsiella pneumoniae* strain ZRKP03 plasmid unnamed5, complete sequence | *Klebsiella pneumoniae* | 9082 | 10336 | 100% | 0 | 100 | 5596 | 5596 | CP050347.1 |
|  | *Klebsiella pneumoniae* strain XH1508 plasmid pXH1508-5, complete sequence | *Klebsiella pneumoniae* | 9070 | 10336 | 100% | 0 | 100 | 5596 | 5596 | CP092791.1 |
|  | *Klebsiella pneumoniae* strain KP1878 plasmid p1878-6k, complete sequence | *Klebsiella pneumoniae* | 9047 | 10343 | 100% | 0 | 99.98 | 5596 | 6796 | CP073000.1 |
|  | *Klebsiella pneumoniae* strain 16HN-263 plasmid p16HN-263_3, complete sequence | *Klebsiella pneumoniae* | 9010 | 10336 | 100% | 0 | 100 | 5596 | 5596 | CP045266.1 |
|  | *Klebsiella pneumoniae* strain KP-CT77 plasmid unnamed2, complete sequence | *Klebsiella pneumoniae* | 9005 | 10336 | 100% | 0 | 100 | 5596 | 5596 | CP080308.1 |
|  | *Klebsiella pneumoniae* strain S234 plasmid pS234-5, complete sequence | *Klebsiella pneumoniae* | 8981 | 10336 | 100% | 0 | 100 | 5596 | 5596 | CP102191.1 |
|  | *Klebsiella pneumoniae* strain KP51248 plasmid p51248_2, complete sequence | *Klebsiella pneumoniae* | 8981 | 10188 | 99% | 0 | 99.55 | 5596 | 5598 | CP070566.1 |
|  | *Klebsiella pneumoniae* strain hvKP319 plasmid unnamed5, complete sequence | *Klebsiella pneumoniae* | 8966 | 10336 | 100% | 0 | 100 | 5596 | 5596 | CP101769.1 |
|  | *Klebsiella pneumoniae* strain KP20194b2 plasmid pKP20194b2-p5, complete sequence | *Klebsiella pneumoniae* | 8938 | 10330 | 100% | 0 | 99.98 | 5596 | 5596 | CP054767.1 |
|  | *Klebsiella pneumoniae* strain 140253 plasmid p4_140253, complete sequence | *Klebsiella pneumoniae* | 8887 | 10330 | 99% | 0 | 100 | 5596 | 5595 | CP097631.1 |
|  | *Klebsiella pneumoniae* isolate 13 genome assembly, plasmid: P5 | *Klebsiella pneumoniae* | 8857 | 10204 | 99% | 0 | 99.61 | 5596 | 5602 | OW968430.1 |
|  | *Klebsiella pneumoniae* strain CR-HvKP3 plasmid p5-CR-HvKP3, complete sequence | *Klebsiella pneumoniae* | 8761 | 10336 | 100% | 0 | 100 | 5596 | 5596 | MW598235.1 |
|  | *Klebsiella pneumoniae* strain KP20194c plasmid pKP20194c-p5, complete sequence | *Klebsiella pneumoniae* | 8741 | 10330 | 100% | 0 | 100 | 5596 | 5596 | CP054761.1 |
|  | *Klebsiella pneumoniae* strain CRKP66R plasmid pCRKP66R-6, complete sequence | *Klebsiella pneumoniae* | 8741 | 10336 | 100% | 0 | 100 | 5596 | 5596 | CP063838.1 |
|  | *Klebsiella pneumoniae* *subsp.* pneumoniae strain DD01304 plasmid pDD01304-4, complete sequence | *Klebsiella pneumoniae* *subsp.* pneumoniae | 8715 | 10336 | 100% | 0 | 100 | 5596 | 5596 | CP087610.1 |
|  | *Klebsiella pneumoniae* strain 36 plasmid pKP36_6, complete sequence | *Klebsiella pneumoniae* | 8704 | 10336 | 100% | 0 | 100 | 5596 | 5596 | CP082764.1 |
|  | *Klebsiella pneumoniae* strain 21091025 plasmid p21091025_4, complete sequence | *Klebsiella pneumoniae* | 8702 | 10336 | 100% | 0 | 100 | 5596 | 5596 | CP095268.1 |
|  | *Klebsiella pneumoniae* strain FO15 plasmid unnamed2, complete sequence | *Klebsiella pneumoniae* | 8639 | 17396 | 100% | 0 | 100 | 5596 | 9417 | CP073004.1 |
|  | *Klebsiella pneumoniae* strain KP58 plasmid pKP58-5, complete sequence | *Klebsiella pneumoniae* | 8480 | 10336 | 100% | 0 | 100 | 5596 | 5596 | CP041378.1 |
|  | *Klebsiella pneumoniae* strain KP18-2079 plasmid pKP18-2079_5kb, complete sequence | *Klebsiella pneumoniae* | 8436 | 10336 | 100% | 0 | 100 | 5596 | 5596 | MT090963.1 |
|  | *Klebsiella pneumoniae* strain CR-HvKP5 plasmid pCR-HvKP5-p5, complete sequence | *Klebsiella pneumoniae* | 8407 | 10336 | 100% | 0 | 100 | 5596 | 5596 | CP040550.1 |
|  | *Klebsiella pneumoniae* strain F726925 plasmid pF726925-4, complete sequence | *Klebsiella pneumoniae* | 8392 | 10336 | 100% | 0 | 100 | 5596 | 5596 | CP081824.1 |
|  | *Klebsiella pneumoniae* strain CDI694 plasmid pCDI694-5.6, complete sequence | *Klebsiella pneumoniae* | 8381 | 10336 | 100% | 0 | 100 | 5596 | 5596 | CP077774.1 |
|  | *Klebsiella pneumoniae* *subsp.* pneumoniae strain DD02341 plasmid pDD02341-4, complete sequence | *Klebsiella pneumoniae* *subsp.* pneumoniae | 8351 | 10336 | 100% | 0 | 100 | 5596 | 5596 | CP087633.1 |
|  | *Klebsiella pneumoniae* strain XH1507 plasmid pXH1507-6, complete sequence | *Klebsiella pneumoniae* | 8351 | 10336 | 100% | 0 | 100 | 5596 | 5596 | CP092799.1 |
|  | *Klebsiella pneumoniae* strain 21072329 plasmid p21072329_5, complete sequence | *Klebsiella pneumoniae* | 8222 | 10336 | 100% | 0 | 100 | 5596 | 5596 | CP095239.1 |
|  | *Klebsiella pneumoniae* strain KP69 plasmid p69-4, complete sequence | *Klebsiella pneumoniae* | 8216 | 10336 | 100% | 0 | 100 | 5596 | 5596 | CP025460.1 |
|  | *Klebsiella pneumoniae* strain JX-CR-hvKP-9 plasmid pJX9-5, complete sequence | *Klebsiella pneumoniae* | 8202 | 10336 | 100% | 0 | 100 | 5596 | 5596 | CP064216.1 |
|  | *Klebsiella pneumoniae* *subsp.* pneumoniae strain KP65 plasmid p65_3, complete sequence | *Klebsiella pneumoniae* *subsp.* pneumoniae | 8196 | 10330 | 100% | 0 | 99.98 | 5596 | 5596 | CP101566.1 |
|  | *Klebsiella pneumoniae* strain XHKP502 plasmid pXHKP502-5, complete sequence | *Klebsiella pneumoniae* | 8196 | 10330 | 100% | 0 | 99.98 | 5596 | 5596 | CP066909.1 |
|  | *Klebsiella pneumoniae* strain WCHKP2080 plasmid p2_095080, complete sequence | *Klebsiella pneumoniae* | 8187 | 10336 | 100% | 0 | 100 | 5596 | 5596 | CP036364.1 |
|  | *Klebsiella pneumoniae* strain JX-CR-hvKP-7 plasmid pJX7-5, complete sequence | *Klebsiella pneumoniae* | 8179 | 10336 | 100% | 0 | 100 | 5596 | 5596 | CP064228.1 |
|  | *Klebsiella pneumoniae* strain KP18-1 plasmid pKP18-1-3, complete sequence | *Klebsiella pneumoniae* | 8170 | 10336 | 100% | 0 | 100 | 5596 | 5596 | CP082000.1 |
|  | *Klebsiella pneumoniae* strain KP18-41 plasmid pKP18-41-3, complete sequence | *Klebsiella pneumoniae* | 8168 | 10336 | 100% | 0 | 100 | 5596 | 5596 | CP082009.1 |
|  | Citrobacter koseri ATCC BAA-895 plasmid pCKO2, complete sequence | Citrobacter koseri ATCC BAA-895 | 8139 | 10203 | 99% | 0 | 99.73 | 5596 | 5601 | CP000824.1 |
|  | *Klebsiella pneumoniae* strain KPA9853 plasmid pA9853_1, complete sequence | *Klebsiella pneumoniae* | 8106 | 10188 | 99% | 0 | 99.53 | 5596 | 5598 | CP070601.1 |
|  | *Klebsiella pneumoniae* strain KP55 plasmid pKP55_6, complete sequence | *Klebsiella pneumoniae* | 8104 | 10336 | 100% | 0 | 100 | 5596 | 5596 | CP055300.1 |
|  | *Klebsiella pneumoniae* *subsp.* pneumoniae strain DD02391 plasmid pDD02391-5, complete sequence | *Klebsiella pneumoniae* *subsp.* pneumoniae | 8094 | 10336 | 100% | 0 | 100 | 5596 | 5596 | CP087644.1 |
|  | *Klebsiella pneumoniae* *subsp.* pneumoniae strain WCHKP015093 plasmid p3_015093, complete sequence | *Klebsiella pneumoniae* *subsp.* pneumoniae | 8082 | 10336 | 100% | 0 | 100 | 5596 | 5596 | CP036304.1 |
|  | *Klebsiella pneumoniae* strain KP20194c4 plasmid pKP20194c4-p5, complete sequence | *Klebsiella pneumoniae* | 8065 | 10330 | 100% | 0 | 99.98 | 5596 | 5596 | CP054749.1 |
|  | *Klebsiella pneumoniae* *subsp.* pneumoniae strain SCKP020143 plasmid p4_020143, complete sequence | *Klebsiella pneumoniae* *subsp.* pneumoniae | 8052 | 10336 | 100% | 0 | 100 | 5596 | 5596 | CP028546.2 |
|  | *Klebsiella pneumoniae* isolate 392 genome assembly, plasmid: P4 | *Klebsiella pneumoniae* | 8043 | 10304 | 99% | 0 | 99.91 | 5596 | 5598 | OW848890.1 |
|  | *Klebsiella pneumoniae* strain WCHKP2 plasmid p2_020002, complete sequence | *Klebsiella pneumoniae* | 8013 | 10336 | 100% | 0 | 100 | 5596 | 5596 | CP028540.3 |
|  | *Klebsiella pneumoniae* strain BJCFK909 plasmid p4s2, complete sequence | *Klebsiella pneumoniae* | 7956 | 10336 | 100% | 0 | 100 | 5596 | 5596 | CP034127.1 |
|  | *Klebsiella pneumoniae* strain ARLG-4861 plasmid pC592_5, complete sequence | *Klebsiella pneumoniae* | 7801 | 9037 | 99% | 0 | 99.76 | 5596 | 5584 | CP067621.1 |
|  | *Klebsiella pneumoniae* strain L39_2 plasmid p6_L39, complete sequence | *Klebsiella pneumoniae* | 7781 | 10336 | 100% | 0 | 100 | 5596 | 5596 | CP033959.1 |
|  | *Klebsiella pneumoniae* strain F44 plasmid p44-4, complete sequence | *Klebsiella pneumoniae* | 7758 | 10336 | 100% | 0 | 100 | 5596 | 5596 | CP025465.1 |
|  | *Klebsiella pneumoniae* strain 135077 plasmid p3_135077, complete sequence | *Klebsiella pneumoniae* | 7705 | 10336 | 100% | 0 | 100 | 5596 | 5596 | CP073295.1 |
|  | *Klebsiella pneumoniae* strain K191663 plasmid unnamed1, complete sequence | *Klebsiella pneumoniae* | 7677 | 10336 | 100% | 0 | 100 | 5596 | 5596 | CP080354.1 |
|  | *Klebsiella pneumoniae* strain KP55 plasmid pKPC-5505, complete sequence | *Klebsiella pneumoniae* | 7670 | 10336 | 100% | 0 | 100 | 5596 | 5596 | OL891655.1 |
|  | *Klebsiella pneumoniae* isolate 97706988-b809-11e8-aae5-3c4a9275d6c8 genome assembly, chromosome: 1 | *Klebsiella pneumoniae* | 7625 | 11066 | 100% | 0 | 100 | 5596 | 5652578 | LR596813.1 |
|  | *Klebsiella pneumoniae* strain KP-C76 plasmid unnamed1, complete sequence | *Klebsiella pneumoniae* | 7579 | 10336 | 100% | 0 | 100 | 5596 | 5596 | CP080302.1 |
|  | *Klebsiella pneumoniae* strain KP-426 plasmid unnamed2, complete sequence | *Klebsiella pneumoniae* | 7509 | 10336 | 100% | 0 | 100 | 5596 | 5596 | CP080315.1 |
|  | *Klebsiella pneumoniae* strain CR-HvKP4 plasmid pCR-HvKP4-p5, complete sequence | *Klebsiella pneumoniae* | 7492 | 10336 | 100% | 0 | 100 | 5596 | 5596 | CP040544.1 |
|  | *Klebsiella pneumoniae* isolate 392 genome assembly, plasmid: P4 | *Klebsiella pneumoniae* | 7487 | 10304 | 99% | 0 | 99.95 | 5596 | 5599 | OW849056.1 |
|  | *Klebsiella pneumoniae* strain 160111 plasmid p5.6K_L111, complete sequence | *Klebsiella pneumoniae* | 7426 | 10336 | 100% | 0 | 100 | 5596 | 5596 | CP030131.1 |
|  | *Klebsiella pneumoniae* strain KP19-2196 plasmid pKP19-2196-4, complete sequence | *Klebsiella pneumoniae* | 7341 | 10336 | 100% | 0 | 100 | 5596 | 5596 | CP082040.1 |
|  | *Klebsiella pneumoniae* strain KP18-2172 plasmid pKP18-2172-3, complete sequence | *Klebsiella pneumoniae* | 7317 | 10336 | 100% | 0 | 100 | 5596 | 5596 | CP082036.1 |
|  | *Klebsiella pneumoniae* strain ZRKP04 plasmid unnamed5, complete sequence | *Klebsiella pneumoniae* | 7304 | 10336 | 100% | 0 | 100 | 5596 | 5596 | CP050341.1 |
|  | *Klebsiella pneumoniae* strain 49088 plasmid p49088-6.796, complete sequence | *Klebsiella pneumoniae* | 7302 | 10344 | 100% | 0 | 99.97 | 5596 | 6796 | CP088998.1 |
|  | *Klebsiella pneumoniae* strain 37 plasmid pKP37_6, complete sequence | *Klebsiella pneumoniae* | 7286 | 10336 | 100% | 0 | 100 | 5596 | 5596 | CP082758.1 |
|  | *Klebsiella pneumoniae* *subsp.* pneumoniae strain KP21 plasmid p21_3, complete sequence | *Klebsiella pneumoniae* *subsp.* pneumoniae | 7278 | 10336 | 100% | 0 | 100 | 5596 | 5596 | CP101545.1 |
|  | *Klebsiella pneumoniae* *subsp.* pneumoniae strain DD01635 plasmid pDD01635-5, complete sequence | *Klebsiella pneumoniae* *subsp.* pneumoniae | 7254 | 10336 | 100% | 0 | 100 | 5596 | 5596 | CP087662.1 |
|  | *Klebsiella pneumoniae* strain WCHKP090050 plasmid p2_090050, complete sequence | *Klebsiella pneumoniae* | 7177 | 10336 | 100% | 0 | 100 | 5596 | 5596 | CP043372.1 |
|  | *Klebsiella pneumoniae* strain KPN142 plasmid pn142_3, complete sequence | *Klebsiella pneumoniae* | 7167 | 10336 | 100% | 0 | 100 | 5596 | 5596 | CP053878.1 |
|  | *Klebsiella pneumoniae* strain KPC-2 plasmid pKP169-P6, complete sequence | *Klebsiella pneumoniae* | 7118 | 10330 | 100% | 0 | 100 | 5596 | 5596 | CP078128.1 |
|  | *Klebsiella pneumoniae* strain FZKP4523 plasmid p2_FZKP4523, complete sequence | *Klebsiella pneumoniae* | 7107 | 10330 | 99% | 0 | 100 | 5596 | 5595 | CP101536.1 |
|  | *Klebsiella pneumoniae* strain WSCRKP plasmid pWSCRKP-5, complete sequence | *Klebsiella pneumoniae* | 7103 | 10336 | 100% | 0 | 100 | 5596 | 5596 | CP091073.1 |
|  | *Klebsiella pneumoniae* strain WCHKP115011 plasmid p3_115011, complete sequence | *Klebsiella pneumoniae* | 7064 | 10336 | 100% | 0 | 100 | 5596 | 5596 | CP089958.1 |
|  | *Klebsiella pneumoniae* isolate 99060032-b809-11e8-aae5-3c4a9275d6c8 genome assembly, chromosome: 1 | *Klebsiella pneumoniae* | 7059 | 10334 | 99% | 0 | 100 | 5596 | 5479501 | LR596812.1 |
| pCR-hvKP128-KPC-P1 | *Klebsiella pneumoniae* strain 140253 plasmid pKPC2_140253, complete sequence | *Klebsiella pneumoniae* | 1.69E+05 | 3.86E+05 | 100% | 0 | 100 | 154719 | 155793 | CP097627.1 |
|  | *Klebsiella pneumoniae* strain WCHKP115038 plasmid pKPC2_115038, complete sequence | *Klebsiella pneumoniae* | 1.43E+05 | 3.99E+05 | 99% | 0 | 100 | 154719 | 153486 | CP043603.1 |
|  | *Klebsiella pneumoniae* strain WCHKP090050 plasmid pKPC2_090050, complete sequence | *Klebsiella pneumoniae* | 1.43E+05 | 4.02E+05 | 100% | 0 | 100 | 154719 | 154724 | CP043370.1 |
|  | *Klebsiella pneumoniae* *subsp.* pneumoniae strain WCHKP020120 plasmid pKPC2_020120, complete sequence | *Klebsiella pneumoniae* *subsp.* pneumoniae | 1.43E+05 | 4.10E+05 | 100% | 0 | 100 | 154719 | 154719 | CP043358.1 |
|  | *Klebsiella pneumoniae* strain FZKP4523 plasmid pKPC-2_FZKP4523, complete sequence | *Klebsiella pneumoniae* | 1.43E+05 | 4.10E+05 | 91% | 0 | 99.98 | 154719 | 141697 | CP101534.1 |
|  | *Klebsiella pneumoniae* strain WCHKP090374 plasmid pKPC2_090374, complete sequence | *Klebsiella pneumoniae* | 1.43E+05 | 4.31E+05 | 100% | 0 | 99.98 | 154719 | 154728 | CP066536.1 |
|  | *Klebsiella pneumoniae* strain C2660 plasmid pC2660-3-KPC, complete sequence | *Klebsiella pneumoniae* | 1.43E+05 | 3.91E+05 | 99% | 0 | 99.97 | 154719 | 153556 | CP039810.1 |
|  | *Klebsiella pneumoniae* *subsp.* pneumoniae strain WCHKP015093 plasmid pKPC2_015093, complete sequence | *Klebsiella pneumoniae* *subsp.* pneumoniae | 1.43E+05 | 4.21E+05 | 100% | 0 | 99.94 | 154719 | 154724 | CP036301.1 |
|  | *Klebsiella pneumoniae* strain WCHKP090045 plasmid pKPC2_090045, complete sequence | *Klebsiella pneumoniae* | 1.43E+05 | 4.02E+05 | 100% | 0 | 99.94 | 154719 | 154724 | CP043366.1 |
|  | *Klebsiella pneumoniae* strain WCHKP115011 plasmid pKPC2_115011, complete sequence | *Klebsiella pneumoniae* | 1.43E+05 | 4.65E+05 | 97% | 0 | 99.93 | 154719 | 157388 | CP089954.1 |
|  | *Klebsiella pneumoniae* strain WCHKP649 plasmid pKPC2_095649, complete sequence | *Klebsiella pneumoniae* | 1.34E+05 | 4.26E+05 | 100% | 0 | 100 | 154719 | 156099 | CP026584.1 |
|  | *Klebsiella pneumoniae* strain WCHKP115069 plasmid pKPC2_115069, complete sequence | *Klebsiella pneumoniae* | 1.34E+05 | 4.11E+05 | 100% | 0 | 99.99 | 154719 | 154986 | CP033404.1 |
|  | *Klebsiella pneumoniae* strain A1750 plasmid pA1750-KPC, complete sequence | *Klebsiella pneumoniae* | 1.27E+05 | 2.80E+05 | 61% | 0 | 99.96 | 154719 | 111536 | MT108207.1 |
|  | *Klebsiella pneumoniae* strain XHKP6 plasmid pXHKP6-1, complete sequence | *Klebsiella pneumoniae* | 1.27E+05 | 4.82E+05 | 100% | 0 | 99.96 | 154719 | 180300 | CP066888.1 |
|  | *Klebsiella pneumoniae* *subsp.* pneumoniae strain DD01754 plasmid pDD01754-2, complete sequence | *Klebsiella pneumoniae* *subsp.* pneumoniae | 1.27E+05 | 4.76E+05 | 100% | 0 | 99.96 | 154719 | 182556 | CP087647.1 |
|  | *Klebsiella pneumoniae* strain JX-CR-hvKP-2 plasmid pJX2-2, complete sequence | *Klebsiella pneumoniae* | 1.27E+05 | 4.40E+05 | 100% | 0 | 99.96 | 154719 | 168025 | CP064248.1 |
|  | *Klebsiella pneumoniae* strain WCHKP020037 plasmid pKPC2_020037, complete sequence | *Klebsiella pneumoniae* | 1.27E+05 | 4.72E+05 | 100% | 0 | 99.96 | 154719 | 172770 | CP036372.1 |
|  | *Klebsiella pneumoniae* strain WCHKP2 plasmid pKPC2_020002, complete sequence | *Klebsiella pneumoniae* | 1.27E+05 | 4.76E+05 | 100% | 0 | 99.96 | 154719 | 177516 | CP028541.2 |
|  | *Klebsiella pneumoniae* strain XHKP75 plasmid pXHKP75-2, complete sequence | *Klebsiella pneumoniae* | 1.27E+05 | 4.36E+05 | 100% | 0 | 99.96 | 154719 | 163729 | CP066897.1 |
|  | *Klebsiella pneumoniae* strain XHKP309 plasmid pXHKP309-1, complete sequence | *Klebsiella pneumoniae* | 1.27E+05 | 5.03E+05 | 96% | 0 | 99.96 | 154719 | 178237 | CP066901.1 |
|  | *Klebsiella pneumoniae* strain FDAARGOS_444 plasmid unnamed2 | *Klebsiella pneumoniae* | 1.27E+05 | 4.96E+05 | 100% | 0 | 99.96 | 154719 | 187926 | CP023942.1 |
|  | *Klebsiella pneumoniae* strain KP1878 plasmid p1878-163k, complete sequence | *Klebsiella pneumoniae* | 1.27E+05 | 4.42E+05 | 97% | 0 | 99.93 | 154719 | 163348 | CP072998.1 |
|  | *Klebsiella pneumoniae* strain KP1880 plasmid pKPC1880, complete sequence | *Klebsiella pneumoniae* | 1.27E+05 | 4.35E+05 | 100% | 0 | 99.92 | 154719 | 168960 | CP061347.1 |
|  | *Klebsiella pneumoniae* strain 675920 plasmid p675920-1, complete sequence | *Klebsiella pneumoniae* | 1.27E+05 | 4.24E+05 | 99% | 0 | 99.91 | 154719 | 163995 | MF133495.1 |
|  | *Klebsiella pneumoniae* strain XHKPN083 plasmid pXHKPN083-2, complete sequence | *Klebsiella pneumoniae* | 1.25E+05 | 3.70E+05 | 76% | 0 | 99.98 | 154719 | 122553 | CP066912.1 |
|  | *Klebsiella pneumoniae* strain BSI014 plasmid pBSI014-KPC2 | *Klebsiella pneumoniae* | 1.24E+05 | 4.72E+05 | 100% | 0 | 99.97 | 154719 | 170701 | MT269822.1 |
|  | *Klebsiella pneumoniae* strain L388 plasmid pKPC-L388 | *Klebsiella pneumoniae* | 1.24E+05 | 3.45E+05 | 86% | 0 | 99.97 | 154719 | 145851 | CP029225.1 |
|  | *Klebsiella pneumoniae* strain HvKp-su1 plasmid unnamed1, complete sequence | *Klebsiella pneumoniae* | 1.18E+05 | 3.45E+05 | 83% | 0 | 99.99 | 154719 | 133346 | CP092718.1 |
|  | *Klebsiella pneumoniae* strain 9489 plasmid pBJ9489-KPC | *Klebsiella pneumoniae* | 1.18E+05 | 3.20E+05 | 82% | 0 | 99.99 | 154719 | 132246 | MN821371.1 |
|  | *Klebsiella pneumoniae* strain F1 plasmid pF1_1, complete sequence | *Klebsiella pneumoniae* | 1.17E+05 | 4.21E+05 | 96% | 0 | 99.97 | 154719 | 164510 | CP026131.1 |
|  | *Klebsiella pneumoniae* strain KP137060 plasmid unnamed2, complete sequence | *Klebsiella pneumoniae* | 1.16E+05 | 3.30E+05 | 85% | 0 | 99.9 | 154719 | 146878 | MW218143.1 |
|  | *Klebsiella pneumoniae* strain 49088 plasmid p49088-279.2, complete sequence | *Klebsiella pneumoniae* | 1.16E+05 | 4.67E+05 | 98% | 0 | 99.92 | 154719 | 279210 | CP089000.1 |
|  | *Klebsiella pneumoniae* *subsp.* pneumoniae strain SCKP020079 plasmid pKPC2_020079, complete sequence | *Klebsiella pneumoniae* *subsp.* pneumoniae | 1.16E+05 | 3.57E+05 | 84% | 0 | 99.97 | 154719 | 146790 | CP029381.1 |
|  | *Klebsiella pneumoniae* strain 381810-51 plasmid p181051-KPC, complete sequence | *Klebsiella pneumoniae* | 1.16E+05 | 2.52E+05 | 60% | 0 | 99.97 | 154719 | 303071 | MT920903.1 |
|  | *Klebsiella pneumoniae* strain F726925 plasmid pF726925-1, complete sequence | *Klebsiella pneumoniae* | 1.16E+05 | 4.72E+05 | 100% | 0 | 99.97 | 154719 | 172862 | CP081821.1 |
|  | *Klebsiella pneumoniae* strain 20049 plasmid p20049-KPC, complete sequence | *Klebsiella pneumoniae* | 1.13E+05 | 3.77E+05 | 87% | 0 | 99.97 | 154719 | 151653 | MF168404.1 |
|  | *Klebsiella pneumoniae* *subsp.* pneumoniae strain DD01304 plasmid pDD01304-2, complete sequence | *Klebsiella pneumoniae* *subsp.* pneumoniae | 1.12E+05 | 4.14E+05 | 100% | 0 | 99.99 | 154719 | 169136 | CP087608.1 |
|  | *Klebsiella pneumoniae* strain SH12 plasmid pSH12_KPC, complete sequence | *Klebsiella pneumoniae* | 1.12E+05 | 4.45E+05 | 97% | 0 | 99.99 | 154719 | 167468 | CP040835.1 |
|  | *Klebsiella pneumoniae* strain BSI054 plasmid pBSI054-KPC2 | *Klebsiella pneumoniae* | 1.12E+05 | 4.64E+05 | 100% | 0 | 99.99 | 154719 | 168038 | MT269833.1 |
|  | *Klebsiella pneumoniae* strain BSI052 plasmid pBSI052-KPC2 | *Klebsiella pneumoniae* | 1.12E+05 | 4.50E+05 | 100% | 0 | 99.99 | 154719 | 165819 | MT269832.1 |
|  | *Klebsiella pneumoniae* strain JX-CR-hvKP-1 plasmid pJX1-1, complete sequence | *Klebsiella pneumoniae* | 1.12E+05 | 4.39E+05 | 99% | 0 | 99.98 | 154719 | 167834 | CP064253.1 |
|  | *Klebsiella pneumoniae* strain 12139 plasmid p12139-KPC, complete sequence | *Klebsiella pneumoniae* | 1.12E+05 | 4.44E+05 | 99% | 0 | 99.98 | 154719 | 169424 | MF168403.1 |
|  | *Klebsiella pneumoniae* strain IR5077_1 plasmid unnamed3, complete sequence | *Klebsiella pneumoniae* | 1.12E+05 | 2.61E+05 | 54% | 0 | 99.98 | 154719 | 292919 | CP097673.1 |
|  | *Klebsiella pneumoniae* strain A1708 plasmid pA1708-KPC, complete sequence | *Klebsiella pneumoniae* | 1.12E+05 | 4.69E+05 | 97% | 0 | 99.98 | 154719 | 173280 | MT810354.1 |
|  | *Klebsiella pneumoniae* strain 911021 plasmid p911021-KPC, complete sequence | *Klebsiella pneumoniae* | 1.12E+05 | 4.56E+05 | 99% | 0 | 99.97 | 154719 | 169824 | MK036888.1 |
|  | *Klebsiella pneumoniae* strain 33367 plasmid p33367_KPC2, complete sequence | *Klebsiella pneumoniae* | 1.12E+05 | 3.96E+05 | 87% | 0 | 99.97 | 154719 | 150096 | CP099415.1 |
|  | *Klebsiella pneumoniae* strain 2014042281 plasmid p42281-KPC, complete sequence | *Klebsiella pneumoniae* | 1.12E+05 | 3.27E+05 | 76% | 0 | 99.97 | 154719 | 115305 | MT810369.1 |
|  | *Klebsiella pneumoniae* strain CRKP78R plasmid p3, complete sequence | *Klebsiella pneumoniae* | 1.12E+05 | 4.03E+05 | 87% | 0 | 99.97 | 154719 | 149407 | CP066256.1 |
|  | *Klebsiella pneumoniae* strain KPN361 plasmid pKPN361-1, complete sequence | *Klebsiella pneumoniae* | 1.12E+05 | 4.43E+05 | 99% | 0 | 99.97 | 154719 | 169824 | CP053017.1 |
|  | *Klebsiella pneumoniae* strain 64917 plasmid p64917-KPC, complete sequence | *Klebsiella pneumoniae* | 1.12E+05 | 4.44E+05 | 99% | 0 | 99.96 | 154719 | 169419 | MF168405.1 |
|  | *Klebsiella pneumoniae* strain 08291 plasmid pW08291-KPC, complete sequence | *Klebsiella pneumoniae* | 1.12E+05 | 4.39E+05 | 99% | 0 | 99.96 | 154719 | 169804 | MN842295.1 |
|  | *Klebsiella pneumoniae* strain 246421 plasmid p246421-KPC, complete sequence | *Klebsiella pneumoniae* | 1.11E+05 | 3.86E+05 | 84% | 0 | 99.98 | 154719 | 145209 | MT810356.1 |
|  | Citrobacter werkmanii strain LYYSPS2 plasmid pLYYSPS2-3, complete sequence | Citrobacter werkmanii | 1.11E+05 | 1.85E+05 | 49% | 0 | 99.98 | 154719 | 87232 | MZ342958.1 |
|  | *Escherichia coli* strain HNEC55 plasmid pHNEC55, complete sequence | *Escherichia coli* | 1.11E+05 | 1.89E+05 | 53% | 0 | 99.97 | 154719 | 81498 | KT879914.1 |
|  | *Klebsiella pneumoniae* *subsp.* pneumoniae strain SH9 plasmid pSH9-CTX-TEM, complete sequence | *Klebsiella pneumoniae* *subsp.* pneumoniae | 1.11E+05 | 2.23E+05 | 53% | 0 | 99.96 | 154719 | 98684 | MH255829.1 |
|  | *Escherichia coli* strain XD35 plasmid pXD35004, complete sequence | *Escherichia coli* | 1.10E+05 | 1.91E+05 | 55% | 0 | 99.86 | 154719 | 85891 | CP089137.1 |
|  | *Klebsiella pneumoniae* strain WCHKP3 plasmid pKPC2_020003, complete sequence | *Klebsiella pneumoniae* | 1.10E+05 | 3.94E+05 | 100% | 0 | 99.98 | 154719 | 154957 | CP031720.1 |
|  | *Escherichia coli* strain 7A8 plasmid pHN7A8, complete sequence | *Escherichia coli* | 1.09E+05 | 1.98E+05 | 56% | 0 | 99.99 | 154719 | 76878 | JN232517.1 |
|  | Enterobacter cloacae strain CBG15936 plasmid pTEM-CBG, complete sequence | Enterobacter cloacae | 1.09E+05 | 1.84E+05 | 56% | 0 | 99.98 | 154719 | 75044 | CP046117.1 |
|  | *Escherichia coli* strain HNC02 plasmid pHNHNC02, complete sequence | *Escherichia coli* | 1.09E+05 | 1.95E+05 | 53% | 0 | 99.98 | 154719 | 76869 | MG197497.1 |
|  | *Escherichia coli* strain HZMPC32 plasmid pHNMPC32, complete sequence | *Escherichia coli* | 1.09E+05 | 1.81E+05 | 53% | 0 | 99.96 | 154719 | 74768 | MG197499.1 |
|  | *Klebsiella pneumoniae* p477Kp plasmid, complete sequence | *Klebsiella pneumoniae* | 1.09E+05 | 1.85E+05 | 56% | 0 | 99.96 | 154719 | 74768 | LN897475.2 |
|  | *Klebsiella pneumoniae* p397Kp plasmid, complete sequence | *Klebsiella pneumoniae* | 1.09E+05 | 1.98E+05 | 56% | 0 | 99.96 | 154719 | 76863 | LN897474.2 |
|  | *Klebsiella pneumoniae* strain BSI047 plasmid pBSI047-KPC2 | *Klebsiella pneumoniae* | 1.09E+05 | 3.99E+05 | 88% | 0 | 99.94 | 154719 | 139571 | MT269830.1 |
|  | *Klebsiella pneumoniae* strain SWU01 plasmid unnamed, complete sequence | *Klebsiella pneumoniae* | 1.09E+05 | 4.24E+05 | 95% | 0 | 99.97 | 154719 | 162552 | CP018455.1 |
|  | *Klebsiella pneumoniae* *subsp.* pneumoniae strain SH2 plasmid pSH2-85K-MDR, complete sequence | *Klebsiella pneumoniae* *subsp.* pneumoniae | 1.08E+05 | 3.60E+05 | 85% | 0 | 99.96 | 154719 | 149033 | MH643792.1 |
|  | *Klebsiella pneumoniae* strain IR12197_1 plasmid unnamed4, complete sequence | *Klebsiella pneumoniae* | 1.06E+05 | 2.93E+05 | 78% | 0 | 99.97 | 154719 | 159393 | CP097707.1 |
|  | *Klebsiella pneumoniae* *subsp.* pneumoniae strain RJBSI76-pV plasmid pRJBSI76-pV-3, complete sequence | *Klebsiella pneumoniae* *subsp.* pneumoniae | 1.06E+05 | 3.88E+05 | 94% | 0 | 99.99 | 154719 | 184751 | CP068687.1 |
|  | *Klebsiella pneumoniae* *subsp.* pneumoniae strain RJBSI76 plasmid pRJBSI76-3, complete sequence | *Klebsiella pneumoniae* *subsp.* pneumoniae | 1.06E+05 | 3.88E+05 | 94% | 0 | 99.99 | 154719 | 184748 | CP068692.1 |
|  | *Klebsiella pneumoniae* *subsp.* pneumoniae strain SH9 plasmid pSH9-KPC, complete sequence | *Klebsiella pneumoniae* *subsp.* pneumoniae | 1.05E+05 | 3.13E+05 | 53% | 0 | 99.91 | 154719 | 113941 | MH255827.1 |
|  | *Klebsiella pneumoniae* strain 150040X1B1 plasmid pCTXM65_150040X1B1, complete sequence | *Klebsiella pneumoniae* | 1.04E+05 | 4.10E+05 | 94% | 0 | 99.99 | 154719 | 149214 | CP101727.1 |
|  | *Klebsiella pneumoniae* strain WCHKP090357 plasmid pKPC2_090357, complete sequence | *Klebsiella pneumoniae* | 1.04E+05 | 4.07E+05 | 97% | 0 | 99.99 | 154719 | 149116 | CP066524.1 |
|  | *Klebsiella pneumoniae* strain CDI694 plasmid pCDI694-140.8, complete sequence | *Klebsiella pneumoniae* | 1.04E+05 | 3.51E+05 | 85% | 0 | 99.97 | 154719 | 140828 | CP077777.1 |
|  | *Klebsiella pneumoniae* strain KP18-2079 plasmid pKP18-2079_KPC, complete sequence | *Klebsiella pneumoniae* | 1.04E+05 | 1.86E+05 | 51% | 0 | 99.95 | 154719 | 186564 | MT090959.1 |
|  | *Klebsiella pneumoniae* strain 20150420184 plasmid p420184-KPC, complete sequence | *Klebsiella pneumoniae* | 1.03E+05 | 3.21E+05 | 73% | 0 | 99.98 | 154719 | 108236 | MT810359.1 |
|  | *Escherichia coli* strain OW1E2 plasmid pOW1E2a, complete sequence | *Escherichia coli* | 1.02E+05 | 2.24E+05 | 53% | 0 | 99.98 | 154719 | 108766 | CP067246.1 |
|  | *Escherichia coli* strain NT1N25 plasmid pNT1N25-76kb, complete sequence | *Escherichia coli* | 1.02E+05 | 1.93E+05 | 49% | 0 | 99.97 | 154719 | 76891 | CP075483.1 |
|  | *Klebsiella pneumoniae* strain 135077 plasmid p1_135077, complete sequence | *Klebsiella pneumoniae* | 1.02E+05 | 3.67E+05 | 91% | 0 | 100 | 154719 | 139526 | CP073293.1 |
|  | *Escherichia coli* strain fEC.1 plasmid pfEC.1-3, complete sequence | *Escherichia coli* | 1.02E+05 | 1.80E+05 | 50% | 0 | 99.95 | 154719 | 78319 | OK605583.1 |
|  | Escherichia fergusonii strain EFCF056 plasmid pEF02, complete sequence | Escherichia fergusonii | 1.02E+05 | 2.16E+05 | 53% | 0 | 99.9 | 154719 | 90871 | CP040807.1 |
|  | *Klebsiella pneumoniae* strain 8695 plasmid pFK8695-KPC-33, complete sequence | *Klebsiella pneumoniae* | 1.02E+05 | 3.83E+05 | 85% | 0 | 99.97 | 154719 | 143980 | CP085890.1 |
|  | *Klebsiella pneumoniae* strain 116753 plasmid p116753-KPC, complete sequence | *Klebsiella pneumoniae* | 1.01E+05 | 2.22E+05 | 50% | 0 | 99.64 | 154719 | 137873 | MN891682.1 |
|  | *Klebsiella pneumoniae* strain KP19-3138 plasmid pKP19-3138-4, complete sequence | *Klebsiella pneumoniae* | 1.01E+05 | 2.25E+05 | 50% | 0 | 99.64 | 154719 | 95171 | CP090620.1 |
|  | *Klebsiella pneumoniae* strain 7849 plasmid pKP7849_KPC, complete sequence | *Klebsiella pneumoniae* | 1.01E+05 | 3.57E+05 | 86% | 0 | 99.99 | 154719 | 148977 | MW478298.1 |
|  | *Escherichia coli* strain HNEC46 plasmid PHNEC46, complete sequence | *Escherichia coli* | 1.01E+05 | 1.41E+05 | 50% | 0 | 99.96 | 154719 | 74046 | KX503323.1 |
|  | *Klebsiella pneumoniae* *subsp.* pneumoniae strain HA2 plasmid pHA2-23-KPC, complete sequence | *Klebsiella pneumoniae* *subsp.* pneumoniae | 1.01E+05 | 3.59E+05 | 86% | 0 | 99.98 | 154719 | 148749 | MH643789.1 |
|  | *Klebsiella pneumoniae* strain Kp36 plasmid unnamed2, complete sequence | *Klebsiella pneumoniae* | 98769 | 3.81E+05 | 83% | 0 | 99.99 | 154719 | 142228 | CP047194.1 |
|  | *Escherichia coli* MH13-051M plasmid pMH13-051M_1 DNA, complete genome | *Escherichia coli* | 96529 | 2.23E+05 | 51% | 0 | 98.96 | 154719 | 111544 | AP018572.2 |
|  | *Klebsiella pneumoniae* strain CY814036 plasmid pCY814036-KPC2, complete sequence | *Klebsiella pneumoniae* | 95188 | 3.73E+05 | 90% | 0 | 99.99 | 154719 | 140105 | CP093153.1 |
|  | *Klebsiella pneumoniae* strain F127 plasmid pF127_1, complete sequence | *Klebsiella pneumoniae* | 92616 | 4.10E+05 | 96% | 0 | 99.96 | 154719 | 164501 | CP026141.1 |
|  | *Klebsiella pneumoniae* strain HZMPC43 plasmid pHNMPC43, complete sequence | *Klebsiella pneumoniae* | 92300 | 1.65E+05 | 47% | 0 | 99.98 | 154719 | 69666 | MG197501.1 |
|  | *Klebsiella pneumoniae* strain HZMPC51-2 plasmid pHNMPC51, complete sequence | *Klebsiella pneumoniae* | 92294 | 1.65E+05 | 47% | 0 | 99.98 | 154719 | 69654 | MG197500.1 |
|  | *Klebsiella pneumoniae* strain BSI074 plasmid pBSI074-KPC2 | *Klebsiella pneumoniae* | 92233 | 3.36E+05 | 82% | 0 | 99.98 | 154719 | 135737 | MT269848.1 |
|  | *Klebsiella pneumoniae* strain CRKP66R plasmid pCRKP66R-3, complete sequence | *Klebsiella pneumoniae* | 91175 | 3.30E+05 | 78% | 0 | 99.97 | 154719 | 128675 | CP063835.1 |
|  | *Klebsiella pneumoniae* plasmid pvirhs3_HS-142_NODE2, complete sequence | *Klebsiella pneumoniae* | 90826 | 2.01E+05 | 49% | 0 | 99.95 | 154719 | 130295 | OM975893.1 |
|  | *Klebsiella pneumoniae* strain 21072329 plasmid p21072329_1, complete sequence | *Klebsiella pneumoniae* | 89498 | 4.45E+05 | 93% | 0 | 99.96 | 154719 | 173108 | CP095235.1 |
|  | *Klebsiella pneumoniae* strain KPN35 plasmid pKPN35-1KPC, complete sequence | *Klebsiella pneumoniae* | 88322 | 3.67E+05 | 91% | 0 | 99.97 | 154719 | 153841 | MT920905.1 |
|  | *Klebsiella pneumoniae* strain JNKPN26 plasmid pJNKPN26_KPC, complete sequence | *Klebsiella pneumoniae* | 87619 | 3.26E+05 | 75% | 0 | 99.98 | 154719 | 126203 | MZ546615.1 |
|  | *Klebsiella pneumoniae* strain JNKPN26 plasmid pJNKPN26_KPC, complete sequence | *Klebsiella pneumoniae* | 87619 | 3.18E+05 | 75% | 0 | 99.98 | 154719 | 126203 | CP090204.1 |
|  | *Klebsiella pneumoniae* strain QL24 plasmid pKPC-QL24, complete sequence | *Klebsiella pneumoniae* | 87447 | 2.97E+05 | 74% | 0 | 99.92 | 154719 | 126126 | MH263653.1 |
| pCR-hvKP128-P2 | *Klebsiella pneumoniae* strain KP69 plasmid p69-3, complete sequence | *Klebsiella pneumoniae* | 18569 | 18569 | 99% | 0 | 100 | 10060 | 10060 | CP025459.1 |
|  | *Klebsiella pneumoniae* *subsp.* pneumoniae strain WCHKP020039 plasmid p1_020039, complete sequence | *Klebsiella pneumoniae* *subsp.* pneumoniae | 18561 | 18561 | 99% | 0 | 100 | 10060 | 10060 | CP043346.1 |
|  | *Klebsiella pneumoniae* strain KP55 plasmid pKPC-5504, complete sequence | *Klebsiella pneumoniae* | 18371 | 18572 | 100% | 0 | 99.99 | 10060 | 10059 | OL891654.1 |
|  | *Klebsiella pneumoniae* strain WCHKP020030 plasmid p1_020030, complete sequence | *Klebsiella pneumoniae* | 18081 | 18579 | 100% | 0 | 100 | 10060 | 10060 | CP028788.2 |
|  | *Klebsiella pneumoniae* strain HvKp-su1 plasmid unnamed2, complete sequence | *Klebsiella pneumoniae* | 18081 | 18581 | 100% | 0 | 100 | 10060 | 10061 | CP092719.1 |
|  | *Klebsiella pneumoniae* *subsp.* pneumoniae strain DD02172 plasmid pDD02172-5, complete sequence | *Klebsiella pneumoniae* *subsp.* pneumoniae | 17791 | 18579 | 100% | 0 | 100 | 10060 | 10060 | CP087616.1 |
|  | *Klebsiella pneumoniae* strain WCHKP3 plasmid p2_020003, complete sequence | *Klebsiella pneumoniae* | 17474 | 18579 | 100% | 0 | 100 | 10060 | 10060 | CP031718.1 |
|  | *Klebsiella pneumoniae* strain KP46 plasmid pKP46_5, complete sequence | *Klebsiella pneumoniae* | 17402 | 18579 | 100% | 0 | 100 | 10060 | 10060 | CP090131.1 |
|  | *Klebsiella pneumoniae* strain XHKPN391 plasmid pXHKPN391-2, complete sequence | *Klebsiella pneumoniae* | 17215 | 18579 | 100% | 0 | 100 | 10060 | 10060 | CP066917.1 |
|  | *Klebsiella pneumoniae* strain LSH-KPN25 plasmid pLSH-KPN25-2, complete sequence | *Klebsiella pneumoniae* | 17101 | 18579 | 100% | 0 | 100 | 10060 | 10060 | CP040181.1 |
|  | *Klebsiella pneumoniae* *subsp.* pneumoniae strain WCHKP020120 plasmid p2_020120, complete sequence | *Klebsiella pneumoniae* *subsp.* pneumoniae | 16748 | 18579 | 100% | 0 | 100 | 10060 | 10060 | CP043360.1 |
|  | *Klebsiella pneumoniae* plasmid unnamed, complete sequence | *Klebsiella pneumoniae* | 16689 | 18579 | 100% | 0 | 100 | 10060 | 10060 | MK181634.1 |
|  | *Klebsiella pneumoniae* strain 1632 plasmid p1632-3, complete sequence | *Klebsiella pneumoniae* | 16596 | 18579 | 100% | 0 | 100 | 10060 | 10060 | CP084500.1 |
|  | *Klebsiella pneumoniae* *subsp.* pneumoniae strain SCKP020143 plasmid p3_020143, complete sequence | *Klebsiella pneumoniae* *subsp.* pneumoniae | 16563 | 18579 | 100% | 0 | 100 | 10060 | 10060 | CP028545.2 |
|  | *Klebsiella pneumoniae* strain 140253 plasmid p3_140253, complete sequence | *Klebsiella pneumoniae* | 16526 | 18579 | 100% | 0 | 100 | 10060 | 10060 | CP097630.1 |
|  | *Klebsiella pneumoniae* *subsp.* pneumoniae strain KP18069 plasmid pKP18069-3, complete sequence | *Klebsiella pneumoniae* *subsp.* pneumoniae | 16379 | 18579 | 100% | 0 | 100 | 10060 | 10060 | CP059892.1 |
|  | *Klebsiella pneumoniae* strain 21072329 plasmid p21072329_4, complete sequence | *Klebsiella pneumoniae* | 16255 | 18579 | 100% | 0 | 100 | 10060 | 10060 | CP095238.1 |
|  | *Klebsiella pneumoniae* strain WCHKP115038 plasmid p1_115038, complete sequence | *Klebsiella pneumoniae* | 16122 | 18579 | 100% | 0 | 100 | 10060 | 10060 | CP043606.1 |
|  | *Klebsiella pneumoniae* strain BSI073 plasmid pBSI073-KPC2 | *Klebsiella pneumoniae* | 15815 | 18594 | 100% | 0 | 100 | 10060 | 118923 | MT269846.1 |
|  | *Klebsiella pneumoniae* strain 21080237 plasmid p21080237_6, complete sequence | *Klebsiella pneumoniae* | 15614 | 18573 | 100% | 0 | 99.99 | 10060 | 10060 | CP095246.1 |
|  | *Klebsiella pneumoniae* strain K2606 plasmid unnamed5, complete sequence | *Klebsiella pneumoniae* | 15536 | 16910 | 100% | 0 | 97.56 | 10060 | 10077 | CP047638.1 |
|  | *Klebsiella pneumoniae* *subsp.* pneumoniae strain KP65 plasmid p65_2, complete sequence | *Klebsiella pneumoniae* *subsp.* pneumoniae | 15485 | 18568 | 100% | 0 | 99.98 | 10060 | 10060 | CP101565.1 |
|  | *Klebsiella pneumoniae* strain XHKP53 plasmid pXHKP53-2, complete sequence | *Klebsiella pneumoniae* | 15426 | 18064 | 100% | 0 | 99.32 | 10060 | 10013 | CP066893.1 |
|  | *Klebsiella pneumoniae* strain KP18-2138 plasmid pKP18-2138-3, complete sequence | *Klebsiella pneumoniae* | 15095 | 18579 | 100% | 0 | 100 | 10060 | 10060 | CP082035.1 |
|  | *Klebsiella pneumoniae* *subsp.* pneumoniae strain KP29 plasmid p29_3, complete sequence | *Klebsiella pneumoniae* *subsp.* pneumoniae | 15005 | 18579 | 100% | 0 | 100 | 10060 | 10060 | CP101570.1 |
|  | *Klebsiella pneumoniae* strain KP65 plasmid p3, complete sequence | *Klebsiella pneumoniae* | 14907 | 18579 | 100% | 0 | 100 | 10060 | 10060 | CP044260.1 |
|  | *Klebsiella pneumoniae* strain WCHKP090374 plasmid p1_090374, complete sequence | *Klebsiella pneumoniae* | 14737 | 18579 | 100% | 0 | 100 | 10060 | 10060 | CP066537.1 |
|  | *Klebsiella pneumoniae* strain WCHKP090357 plasmid p2_090357, complete sequence | *Klebsiella pneumoniae* | 14735 | 18579 | 100% | 0 | 100 | 10060 | 10060 | CP066526.1 |
|  | *Klebsiella pneumoniae* strain 135077 plasmid p2_135077, complete sequence | *Klebsiella pneumoniae* | 14694 | 18579 | 100% | 0 | 100 | 10060 | 10060 | CP073294.1 |
|  | *Klebsiella pneumoniae* strain WCHKP7E2 plasmid p1_085072, complete sequence | *Klebsiella pneumoniae* | 14665 | 18579 | 100% | 0 | 100 | 10060 | 10060 | CP028801.2 |
|  | *Klebsiella pneumoniae* strain KPN361 plasmid pKPN361-3, complete sequence | *Klebsiella pneumoniae* | 14641 | 18579 | 100% | 0 | 100 | 10060 | 10060 | CP053019.1 |
|  | *Klebsiella pneumoniae* strain WCHKP115011 plasmid p2_115011, complete sequence | *Klebsiella pneumoniae* | 14345 | 18579 | 100% | 0 | 100 | 10060 | 10060 | CP089957.1 |
|  | *Klebsiella pneumoniae* strain SW1780 plasmid pB, complete sequence | *Klebsiella pneumoniae* | 14345 | 18579 | 100% | 0 | 100 | 10060 | 10060 | CP073304.1 |
|  | *Klebsiella pneumoniae* strain 53374 plasmid p53374-15.0, complete sequence | *Klebsiella pneumoniae* | 14087 | 16957 | 99% | 0 | 97.5 | 10060 | 15032 | CP089015.1 |
|  | *Klebsiella pneumoniae* strain LSH-KPN148 plasmid pLSH-KPN148-3, complete sequence | *Klebsiella pneumoniae* | 13961 | 18579 | 100% | 0 | 100 | 10060 | 10060 | CP040125.1 |
|  | *Klebsiella pneumoniae* strain 150040X1B1 plasmid p1_150040X1B1, complete sequence | *Klebsiella pneumoniae* | 13880 | 18579 | 100% | 0 | 100 | 10060 | 10060 | CP101729.1 |
|  | *Klebsiella pneumoniae* strain ZRKP01 plasmid unnamed4, complete sequence | *Klebsiella pneumoniae* | 13806 | 18579 | 100% | 0 | 100 | 10060 | 10060 | CP050358.1 |
|  | *Klebsiella pneumoniae* strain WCHKP020037 plasmid p2_020037, complete sequence | *Klebsiella pneumoniae* | 13771 | 18579 | 100% | 0 | 100 | 10060 | 10060 | CP036374.1 |
|  | *Klebsiella pneumoniae* *subsp.* pneumoniae strain DD01304 plasmid pDD01304-3, complete sequence | *Klebsiella pneumoniae* *subsp.* pneumoniae | 13577 | 18537 | 100% | 0 | 99.89 | 10060 | 10062 | CP087609.1 |
|  | *Klebsiella pneumoniae* *subsp.* pneumoniae strain DD02297 plasmid pDD02297-3, complete sequence | *Klebsiella pneumoniae* *subsp.* pneumoniae | 13551 | 18537 | 100% | 0 | 99.89 | 10060 | 10062 | CP087637.1 |
|  | *Klebsiella pneumoniae* isolate 91a83dc8-b809-11e8-aae5-3c4a9275d6c8 genome assembly, chromosome: 1 | *Klebsiella pneumoniae* | 13428 | 18577 | 100% | 0 | 100 | 10060 | 5600586 | LR596806.1 |
|  | *Klebsiella pneumoniae* strain WCHKP090050 plasmid p1_090050, complete sequence | *Klebsiella pneumoniae* | 13409 | 18579 | 100% | 0 | 100 | 10060 | 10060 | CP043371.1 |
|  | *Klebsiella pneumoniae* strain CP19 plasmid unnamed3, complete sequence | *Klebsiella pneumoniae* | 13309 | 18561 | 100% | 0 | 99.94 | 10060 | 10064 | CP073354.1 |
|  | *Klebsiella pneumoniae* strain BJ107 plasmid pBJ107-KPC, complete sequence | *Klebsiella pneumoniae* | 13243 | 18599 | 100% | 0 | 100 | 10060 | 115489 | MN891684.1 |
|  | *Klebsiella pneumoniae* strain IR5077_1 plasmid unnamed1, complete sequence | *Klebsiella pneumoniae* | 12979 | 18381 | 100% | 0 | 99.66 | 10060 | 10033 | CP097671.1 |
|  | *Klebsiella pneumoniae* *subsp.* pneumoniae strain DD02162 plasmid pDD02162-4, complete sequence | *Klebsiella pneumoniae* *subsp.* pneumoniae | 12942 | 18579 | 100% | 0 | 100 | 10060 | 10060 | CP087622.1 |
|  | *Klebsiella pneumoniae* strain FDAARGOS_444 plasmid unnamed3, complete sequence | *Klebsiella pneumoniae* | 12931 | 18573 | 100% | 0 | 99.99 | 10060 | 10061 | CP023944.1 |
|  | *Klebsiella pneumoniae* plasmid unnamed, complete sequence | *Klebsiella pneumoniae* | 12702 | 18579 | 100% | 0 | 100 | 10060 | 10060 | MK181632.1 |
|  | *Klebsiella pneumoniae* strain KP167 plasmid pKP167-10, complete sequence | *Klebsiella pneumoniae* | 12482 | 18579 | 100% | 0 | 100 | 10060 | 10060 | CP098761.1 |
|  | *Klebsiella pneumoniae* strain KPN142 plasmid pn142_2, complete sequence | *Klebsiella pneumoniae* | 12408 | 18579 | 100% | 0 | 100 | 10060 | 10060 | CP053877.1 |
|  | *Klebsiella pneumoniae* strain XHKP75 plasmid pXHKP75-3, complete sequence | *Klebsiella pneumoniae* | 12369 | 18579 | 100% | 0 | 100 | 10060 | 10060 | CP066898.1 |
|  | *Klebsiella pneumoniae* strain XHKP6 plasmid pXHKP6-2, complete sequence | *Klebsiella pneumoniae* | 12259 | 18615 | 100% | 0 | 99.98 | 10060 | 10098 | CP066889.1 |
|  | *Klebsiella pneumoniae* strain KP18-3-8 plasmid pKP18-3-8_COL, complete sequence | *Klebsiella pneumoniae* | 12237 | 18573 | 100% | 0 | 100 | 10060 | 10060 | MT035877.1 |
|  | *Klebsiella pneumoniae* strain KP19-2029 plasmid pKP19-2029-Col, complete sequence | *Klebsiella pneumoniae* | 12235 | 18579 | 100% | 0 | 100 | 10060 | 10060 | CP047162.1 |
|  | *Klebsiella pneumoniae* strain ZRKP03 plasmid unnamed4, complete sequence | *Klebsiella pneumoniae* | 12174 | 18546 | 100% | 0 | 99.91 | 10060 | 10060 | CP050346.1 |
|  | *Klebsiella pneumoniae* strain F726925 plasmid pF726925-3, complete sequence | *Klebsiella pneumoniae* | 12067 | 18579 | 100% | 0 | 100 | 10060 | 10060 | CP081823.1 |
|  | *Klebsiella pneumoniae* strain WCHKP090045 plasmid p1_090045, complete sequence | *Klebsiella pneumoniae* | 12057 | 18579 | 100% | 0 | 100 | 10060 | 10060 | CP043367.1 |
|  | *Klebsiella pneumoniae* strain FZKP4523 plasmid p1_FZKP4523, complete sequence | *Klebsiella pneumoniae* | 11960 | 18579 | 100% | 0 | 100 | 10060 | 10060 | CP101535.1 |
|  | *Klebsiella pneumoniae* strain SCKP020018 plasmid p1_020018, complete sequence | *Klebsiella pneumoniae* | 11923 | 18579 | 100% | 0 | 100 | 10060 | 10060 | CP043584.1 |
|  | *Klebsiella pneumoniae* isolate 97706988-b809-11e8-aae5-3c4a9275d6c8 genome assembly, chromosome: 1 | *Klebsiella pneumoniae* | 11854 | 19114 | 100% | 0 | 100 | 10060 | 5652578 | LR596813.1 |
|  | *Klebsiella pneumoniae* strain KP20194b2 plasmid pKP20194b2-p4, complete sequence | *Klebsiella pneumoniae* | 11852 | 18580 | 100% | 0 | 100 | 10060 | 11970 | CP054766.1 |
|  | *Klebsiella pneumoniae* strain KP58 plasmid pKP58-4, complete sequence | *Klebsiella pneumoniae* | 11852 | 18580 | 100% | 0 | 100 | 10060 | 11970 | CP041377.1 |
|  | *Klebsiella pneumoniae* strain L39_2 plasmid p5_L39, complete sequence | *Klebsiella pneumoniae* | 11852 | 18580 | 100% | 0 | 100 | 10060 | 11970 | CP033958.1 |
|  | *Klebsiella pneumoniae* isolate 991178e0-b809-11e8-aae5-3c4a9275d6c8 genome assembly, chromosome: 1 | *Klebsiella pneumoniae* | 11852 | 18579 | 100% | 0 | 100 | 10060 | 5570095 | LR596809.1 |
|  | *Klebsiella pneumoniae* isolate 98fb0f42-b809-11e8-aae5-3c4a9275d6c8 genome assembly, chromosome: 1 | *Klebsiella pneumoniae* | 11852 | 18579 | 100% | 0 | 100 | 10060 | 5716474 | LR596807.1 |
|  | *Klebsiella pneumoniae* strain CR-HvKP4 plasmid pCR-HvKP4-p4, complete sequence | *Klebsiella pneumoniae* | 11852 | 18579 | 100% | 0 | 100 | 10060 | 11970 | CP040543.1 |
|  | *Klebsiella pneumoniae* strain CR-HvKP5 plasmid pCR-HvKP5-p4, complete sequence | *Klebsiella pneumoniae* | 11852 | 18579 | 100% | 0 | 100 | 10060 | 11970 | CP040549.1 |
|  | *Klebsiella pneumoniae* strain CR-HvKP1 plasmid pCR-HvKP1-p4, complete sequence | *Klebsiella pneumoniae* | 11852 | 18579 | 100% | 0 | 100 | 10060 | 11970 | CP040537.1 |
|  | *Klebsiella pneumoniae* strain SCKP020009 plasmid p1_020009, complete sequence | *Klebsiella pneumoniae* | 11852 | 18580 | 100% | 0 | 100 | 10060 | 11970 | CP038005.1 |
|  | *Klebsiella pneumoniae* strain K64 plasmid pColRNAI-5, complete sequence | *Klebsiella pneumoniae* | 11852 | 18557 | 99% | 0 | 100 | 10060 | 11934 | CP102395.1 |
|  | *Klebsiella pneumoniae* strain S270v plasmid pS270V-3, complete sequence | *Klebsiella pneumoniae* | 11852 | 18579 | 100% | 0 | 100 | 10060 | 11970 | CP102195.1 |
|  | *Klebsiella pneumoniae* strain hvKP340 plasmid unnamed5, complete sequence | *Klebsiella pneumoniae* | 11852 | 18579 | 100% | 0 | 100 | 10060 | 11970 | CP101781.1 |
|  | *Klebsiella pneumoniae* strain hvKP323 plasmid unnamed3, complete sequence | *Klebsiella pneumoniae* | 11852 | 18557 | 99% | 0 | 100 | 10060 | 11970 | CP101773.1 |
|  | *Klebsiella pneumoniae* strain hvKP841 plasmid unnamed3, complete sequence | *Klebsiella pneumoniae* | 11852 | 18579 | 100% | 0 | 100 | 10060 | 11970 | CP101787.1 |
|  | *Klebsiella pneumoniae* strain hvKP319 plasmid unnamed4, complete sequence | *Klebsiella pneumoniae* | 11852 | 18579 | 100% | 0 | 100 | 10060 | 11970 | CP101768.1 |
|  | *Klebsiella pneumoniae* strain IR12243_1 plasmid unnamed1, complete sequence | *Klebsiella pneumoniae* | 11852 | 18580 | 100% | 0 | 100 | 10060 | 12798 | CP097661.1 |
|  | *Klebsiella pneumoniae* *subsp.* pneumoniae strain DD02280 plasmid pDD02280-3 | *Klebsiella pneumoniae* *subsp.* pneumoniae | 11852 | 18568 | 99% | 0 | 100 | 10060 | 91826 | CP087626.1 |
|  | *Klebsiella pneumoniae* strain KP15 plasmid unnamed3, complete sequence | *Klebsiella pneumoniae* | 11852 | 18579 | 100% | 0 | 100 | 10060 | 99065 | CP087145.1 |
|  | *Klebsiella pneumoniae* strain KP16 plasmid unnamed4, complete sequence | *Klebsiella pneumoniae* | 11852 | 18580 | 100% | 0 | 100 | 10060 | 11970 | CP087150.1 |
|  | *Klebsiella pneumoniae* strain KP14 plasmid unnamed4, complete sequence | *Klebsiella pneumoniae* | 11852 | 18580 | 100% | 0 | 100 | 10060 | 11970 | CP087155.1 |
|  | *Escherichia coli* strain CR-HvKP4TC-2 plasmid pCR-HvKP4TC-2_p4, complete sequence | *Escherichia coli* | 11852 | 18580 | 100% | 0 | 100 | 10060 | 11970 | OM001477.1 |
|  | *Klebsiella pneumoniae* strain XH1507 plasmid pXH1507-5, complete sequence | *Klebsiella pneumoniae* | 11852 | 18557 | 99% | 0 | 100 | 10060 | 11970 | CP092798.1 |
|  | *Klebsiella pneumoniae* strain KP200731214 plasmid pKP-5 | *Klebsiella pneumoniae* | 11852 | 18580 | 100% | 0 | 100 | 10060 | 11970 | CP084748.1 |
|  | *Klebsiella pneumoniae* strain 1864 plasmid p1864-4, complete sequence | *Klebsiella pneumoniae* | 11852 | 18575 | 99% | 0 | 100 | 10060 | 11970 | CP084496.1 |
|  | *Klebsiella pneumoniae* strain 19PDR22 plasmid p7, complete sequence | *Klebsiella pneumoniae* | 11852 | 18579 | 100% | 0 | 100 | 10060 | 11970 | CP076551.1 |
|  | *Klebsiella pneumoniae* strain DD521 plasmid pDD521.4, complete sequence | *Klebsiella pneumoniae* | 11852 | 18579 | 100% | 0 | 100 | 10060 | 11969 | CP075320.1 |
|  | *Escherichia coli* strain CR-HvKP5TC plasmid pCR-HvKP5TC_Vir-p4, complete sequence | *Escherichia coli* | 11852 | 18579 | 100% | 0 | 100 | 10060 | 190129 | MW598247.1 |
|  | *Escherichia coli* strain CR-HvKP4TC plasmid pCR-HvKP4TC_Vir-p4, complete sequence | *Escherichia coli* | 11852 | 18621 | 100% | 0 | 100 | 10060 | 190130 | MW598245.1 |
|  | *Escherichia coli* strain CR-HvKP1TC plasmid pCR-HvKP1TC_Vir-p4, complete sequence | *Escherichia coli* | 11852 | 18579 | 100% | 0 | 100 | 10060 | 190129 | MW598240.1 |
|  | *Klebsiella pneumoniae* strain BSI058 plasmid pBSI058-KPC2 | *Klebsiella pneumoniae* | 11852 | 18579 | 100% | 0 | 100 | 10060 | 114712 | MT269836.1 |
|  | *Klebsiella pneumoniae* strain KP1517 plasmid unnamed3, complete sequence | *Klebsiella pneumoniae* | 11852 | 18580 | 100% | 0 | 100 | 10060 | 11970 | CP072466.1 |
|  | *Klebsiella pneumoniae* strain WCHKP090329 plasmid p2_090329, complete sequence | *Klebsiella pneumoniae* | 11852 | 18579 | 100% | 0 | 100 | 10060 | 11970 | CP066521.1 |
|  | *Klebsiella pneumoniae* strain JX-CR-hvKP-3 plasmid pJX3-4, complete sequence | *Klebsiella pneumoniae* | 11852 | 18580 | 100% | 0 | 100 | 10060 | 11970 | CP064245.1 |
|  | *Klebsiella pneumoniae* strain JX-CR-hvKP-1 plasmid pJX1-4, complete sequence | *Klebsiella pneumoniae* | 11852 | 18579 | 100% | 0 | 100 | 10060 | 11970 | CP064256.1 |
|  | *Klebsiella pneumoniae* strain FRPDR plasmid pFRPDR_5, complete sequence | *Klebsiella pneumoniae* | 11852 | 18579 | 100% | 0 | 100 | 10060 | 11970 | CP063764.1 |
|  | *Escherichia coli* strain CR-HvKP1TC-3 plasmid pCR-HvKP1TC-3_p4, complete sequence | *Escherichia coli* | 11849 | 18575 | 99% | 0 | 100 | 10060 | 11970 | OM001473.1 |
|  | *Klebsiella pneumoniae* *subsp.* pneumoniae strain RJBSI76 plasmid pRJBSI76-4, complete sequence | *Klebsiella pneumoniae* *subsp.* pneumoniae | 11847 | 18573 | 100% | 0 | 99.98 | 10060 | 10206 | CP068693.1 |
|  | *Klebsiella pneumoniae* strain 37 plasmid pKP37_5, complete sequence | *Klebsiella pneumoniae* | 11845 | 18573 | 100% | 0 | 99.98 | 10060 | 11969 | CP082757.1 |
|  | *Klebsiella pneumoniae* strain FDAARGOS_443 plasmid unnamed4, complete sequence | *Klebsiella pneumoniae* | 11843 | 18571 | 100% | 0 | 99.97 | 10060 | 11972 | CP023932.1 |
|  | *Escherichia coli* strain CR-HvKP5TC-5 plasmid pCR-HvKP5TC-5_p4, complete sequence | *Escherichia coli* | 11841 | 18568 | 99% | 0 | 100 | 10060 | 11970 | OM001479.1 |
| pCR-hvKP132-KPC-P1 | *Klebsiella pneumoniae* strain 140253 plasmid pKPC2_140253, complete sequence | *Klebsiella pneumoniae* | 1.69E+05 | 3.87E+05 | 100% | 0 | 100 | 154719 | 155793 | CP097627.1 |
|  | *Klebsiella pneumoniae* strain WCHKP115038 plasmid pKPC2_115038, complete sequence | *Klebsiella pneumoniae* | 1.43E+05 | 4.03E+05 | 99% | 0 | 100 | 154719 | 153486 | CP043603.1 |
|  | *Klebsiella pneumoniae* strain WCHKP090050 plasmid pKPC2_090050, complete sequence | *Klebsiella pneumoniae* | 1.43E+05 | 4.05E+05 | 100% | 0 | 100 | 154719 | 154724 | CP043370.1 |
|  | *Klebsiella pneumoniae* *subsp.* pneumoniae strain WCHKP020120 plasmid pKPC2_020120, complete sequence | *Klebsiella pneumoniae* *subsp.* pneumoniae | 1.43E+05 | 4.10E+05 | 100% | 0 | 100 | 154719 | 154719 | CP043358.1 |
|  | *Klebsiella pneumoniae* strain FZKP4523 plasmid pKPC-2_FZKP4523, complete sequence | *Klebsiella pneumoniae* | 1.43E+05 | 4.10E+05 | 91% | 0 | 99.98 | 154719 | 141697 | CP101534.1 |
|  | *Klebsiella pneumoniae* strain WCHKP090374 plasmid pKPC2_090374, complete sequence | *Klebsiella pneumoniae* | 1.43E+05 | 4.31E+05 | 100% | 0 | 99.97 | 154719 | 154728 | CP066536.1 |
|  | *Klebsiella pneumoniae* strain C2660 plasmid pC2660-3-KPC, complete sequence | *Klebsiella pneumoniae* | 1.43E+05 | 3.91E+05 | 99% | 0 | 99.97 | 154719 | 153556 | CP039810.1 |
|  | *Klebsiella pneumoniae* *subsp.* pneumoniae strain WCHKP015093 plasmid pKPC2_015093, complete sequence | *Klebsiella pneumoniae* *subsp.* pneumoniae | 1.43E+05 | 4.21E+05 | 100% | 0 | 99.94 | 154719 | 154724 | CP036301.1 |
|  | *Klebsiella pneumoniae* strain WCHKP090045 plasmid pKPC2_090045, complete sequence | *Klebsiella pneumoniae* | 1.43E+05 | 4.05E+05 | 100% | 0 | 99.94 | 154719 | 154724 | CP043366.1 |
|  | *Klebsiella pneumoniae* strain WCHKP115011 plasmid pKPC2_115011, complete sequence | *Klebsiella pneumoniae* | 1.43E+05 | 4.65E+05 | 97% | 0 | 99.92 | 154719 | 157388 | CP089954.1 |
|  | *Klebsiella pneumoniae* strain WCHKP649 plasmid pKPC2_095649, complete sequence | *Klebsiella pneumoniae* | 1.34E+05 | 4.27E+05 | 100% | 0 | 100 | 154719 | 156099 | CP026584.1 |
|  | *Klebsiella pneumoniae* strain WCHKP115069 plasmid pKPC2_115069, complete sequence | *Klebsiella pneumoniae* | 1.34E+05 | 4.13E+05 | 100% | 0 | 99.99 | 154719 | 154986 | CP033404.1 |
|  | *Klebsiella pneumoniae* strain WCHKP020037 plasmid pKPC2_020037, complete sequence | *Klebsiella pneumoniae* | 1.27E+05 | 4.72E+05 | 100% | 0 | 99.96 | 154719 | 172770 | CP036372.1 |
|  | *Klebsiella pneumoniae* strain WCHKP2 plasmid pKPC2_020002, complete sequence | *Klebsiella pneumoniae* | 1.27E+05 | 4.76E+05 | 100% | 0 | 99.96 | 154719 | 177516 | CP028541.2 |
|  | *Klebsiella pneumoniae* strain A1750 plasmid pA1750-KPC, complete sequence | *Klebsiella pneumoniae* | 1.27E+05 | 2.80E+05 | 61% | 0 | 99.96 | 154719 | 111536 | MT108207.1 |
|  | *Klebsiella pneumoniae* strain XHKP6 plasmid pXHKP6-1, complete sequence | *Klebsiella pneumoniae* | 1.27E+05 | 4.83E+05 | 100% | 0 | 99.96 | 154719 | 180300 | CP066888.1 |
|  | *Klebsiella pneumoniae* *subsp.* pneumoniae strain DD01754 plasmid pDD01754-2, complete sequence | *Klebsiella pneumoniae* *subsp.* pneumoniae | 1.27E+05 | 4.76E+05 | 100% | 0 | 99.96 | 154719 | 182556 | CP087647.1 |
|  | *Klebsiella pneumoniae* strain JX-CR-hvKP-2 plasmid pJX2-2, complete sequence | *Klebsiella pneumoniae* | 1.27E+05 | 4.40E+05 | 100% | 0 | 99.96 | 154719 | 168025 | CP064248.1 |
|  | *Klebsiella pneumoniae* strain XHKP75 plasmid pXHKP75-2, complete sequence | *Klebsiella pneumoniae* | 1.27E+05 | 4.37E+05 | 100% | 0 | 99.96 | 154719 | 163729 | CP066897.1 |
|  | *Klebsiella pneumoniae* strain XHKP309 plasmid pXHKP309-1, complete sequence | *Klebsiella pneumoniae* | 1.27E+05 | 5.04E+05 | 96% | 0 | 99.96 | 154719 | 178237 | CP066901.1 |
|  | *Klebsiella pneumoniae* strain FDAARGOS_444 plasmid unnamed2 | *Klebsiella pneumoniae* | 1.27E+05 | 4.96E+05 | 100% | 0 | 99.95 | 154719 | 187926 | CP023942.1 |
|  | *Klebsiella pneumoniae* strain KP1878 plasmid p1878-163k, complete sequence | *Klebsiella pneumoniae* | 1.27E+05 | 4.40E+05 | 97% | 0 | 99.93 | 154719 | 163348 | CP072998.1 |
|  | *Klebsiella pneumoniae* strain KP1880 plasmid pKPC1880, complete sequence | *Klebsiella pneumoniae* | 1.27E+05 | 4.35E+05 | 100% | 0 | 99.92 | 154719 | 168960 | CP061347.1 |
|  | *Klebsiella pneumoniae* strain 675920 plasmid p675920-1, complete sequence | *Klebsiella pneumoniae* | 1.27E+05 | 4.24E+05 | 99% | 0 | 99.91 | 154719 | 163995 | MF133495.1 |
|  | *Klebsiella pneumoniae* strain XHKPN083 plasmid pXHKPN083-2, complete sequence | *Klebsiella pneumoniae* | 1.25E+05 | 3.70E+05 | 76% | 0 | 99.98 | 154719 | 122553 | CP066912.1 |
|  | *Klebsiella pneumoniae* strain BSI014 plasmid pBSI014-KPC2 | *Klebsiella pneumoniae* | 1.24E+05 | 4.72E+05 | 100% | 0 | 99.97 | 154719 | 170701 | MT269822.1 |
|  | *Klebsiella pneumoniae* strain L388 plasmid pKPC-L388 | *Klebsiella pneumoniae* | 1.24E+05 | 3.45E+05 | 86% | 0 | 99.97 | 154719 | 145851 | CP029225.1 |
|  | *Klebsiella pneumoniae* strain HvKp-su1 plasmid unnamed1, complete sequence | *Klebsiella pneumoniae* | 1.18E+05 | 3.44E+05 | 83% | 0 | 99.99 | 154719 | 133346 | CP092718.1 |
|  | *Klebsiella pneumoniae* strain 9489 plasmid pBJ9489-KPC | *Klebsiella pneumoniae* | 1.18E+05 | 3.20E+05 | 82% | 0 | 99.99 | 154719 | 132246 | MN821371.1 |
|  | *Klebsiella pneumoniae* strain F1 plasmid pF1_1, complete sequence | *Klebsiella pneumoniae* | 1.17E+05 | 4.21E+05 | 96% | 0 | 99.97 | 154719 | 164510 | CP026131.1 |
|  | *Klebsiella pneumoniae* strain KP137060 plasmid unnamed2, complete sequence | *Klebsiella pneumoniae* | 1.16E+05 | 3.31E+05 | 85% | 0 | 99.9 | 154719 | 146878 | MW218143.1 |
|  | *Klebsiella pneumoniae* strain 49088 plasmid p49088-279.2, complete sequence | *Klebsiella pneumoniae* | 1.16E+05 | 4.67E+05 | 98% | 0 | 99.92 | 154719 | 279210 | CP089000.1 |
|  | *Klebsiella pneumoniae* *subsp.* pneumoniae strain SCKP020079 plasmid pKPC2_020079, complete sequence | *Klebsiella pneumoniae* *subsp.* pneumoniae | 1.16E+05 | 3.57E+05 | 84% | 0 | 99.97 | 154719 | 146790 | CP029381.1 |
|  | *Klebsiella pneumoniae* strain 381810-51 plasmid p181051-KPC, complete sequence | *Klebsiella pneumoniae* | 1.16E+05 | 2.70E+05 | 64% | 0 | 99.97 | 154719 | 303071 | MT920903.1 |
|  | *Klebsiella pneumoniae* strain F726925 plasmid pF726925-1, complete sequence | *Klebsiella pneumoniae* | 1.16E+05 | 4.72E+05 | 100% | 0 | 99.97 | 154719 | 172862 | CP081821.1 |
|  | *Klebsiella pneumoniae* strain 20049 plasmid p20049-KPC, complete sequence | *Klebsiella pneumoniae* | 1.13E+05 | 3.77E+05 | 87% | 0 | 99.97 | 154719 | 151653 | MF168404.1 |
|  | *Klebsiella pneumoniae* *subsp.* pneumoniae strain DD01304 plasmid pDD01304-2, complete sequence | *Klebsiella pneumoniae* *subsp.* pneumoniae | 1.12E+05 | 4.14E+05 | 100% | 0 | 99.99 | 154719 | 169136 | CP087608.1 |
|  | *Klebsiella pneumoniae* strain SH12 plasmid pSH12_KPC, complete sequence | *Klebsiella pneumoniae* | 1.12E+05 | 4.45E+05 | 97% | 0 | 99.99 | 154719 | 167468 | CP040835.1 |
|  | *Klebsiella pneumoniae* strain BSI054 plasmid pBSI054-KPC2 | *Klebsiella pneumoniae* | 1.12E+05 | 4.64E+05 | 100% | 0 | 99.99 | 154719 | 168038 | MT269833.1 |
|  | *Klebsiella pneumoniae* strain BSI052 plasmid pBSI052-KPC2 | *Klebsiella pneumoniae* | 1.12E+05 | 4.50E+05 | 100% | 0 | 99.99 | 154719 | 165819 | MT269832.1 |
|  | *Klebsiella pneumoniae* strain JX-CR-hvKP-1 plasmid pJX1-1, complete sequence | *Klebsiella pneumoniae* | 1.12E+05 | 4.39E+05 | 99% | 0 | 99.98 | 154719 | 167834 | CP064253.1 |
|  | *Klebsiella pneumoniae* strain 12139 plasmid p12139-KPC, complete sequence | *Klebsiella pneumoniae* | 1.12E+05 | 4.44E+05 | 99% | 0 | 99.98 | 154719 | 169424 | MF168403.1 |
|  | *Klebsiella pneumoniae* strain IR5077_1 plasmid unnamed3, complete sequence | *Klebsiella pneumoniae* | 1.12E+05 | 2.63E+05 | 54% | 0 | 99.98 | 154719 | 292919 | CP097673.1 |
|  | *Klebsiella pneumoniae* strain A1708 plasmid pA1708-KPC, complete sequence | *Klebsiella pneumoniae* | 1.12E+05 | 4.69E+05 | 97% | 0 | 99.98 | 154719 | 173280 | MT810354.1 |
|  | *Klebsiella pneumoniae* strain 911021 plasmid p911021-KPC, complete sequence | *Klebsiella pneumoniae* | 1.12E+05 | 4.58E+05 | 99% | 0 | 99.97 | 154719 | 169824 | MK036888.1 |
|  | *Klebsiella pneumoniae* strain 33367 plasmid p33367_KPC2, complete sequence | *Klebsiella pneumoniae* | 1.12E+05 | 3.96E+05 | 87% | 0 | 99.97 | 154719 | 150096 | CP099415.1 |
|  | *Klebsiella pneumoniae* strain 2014042281 plasmid p42281-KPC, complete sequence | *Klebsiella pneumoniae* | 1.12E+05 | 3.27E+05 | 76% | 0 | 99.97 | 154719 | 115305 | MT810369.1 |
|  | *Klebsiella pneumoniae* strain CRKP78R plasmid p3, complete sequence | *Klebsiella pneumoniae* | 1.12E+05 | 4.03E+05 | 87% | 0 | 99.97 | 154719 | 149407 | CP066256.1 |
|  | *Klebsiella pneumoniae* strain KPN361 plasmid pKPN361-1, complete sequence | *Klebsiella pneumoniae* | 1.12E+05 | 4.40E+05 | 99% | 0 | 99.97 | 154719 | 169824 | CP053017.1 |
|  | *Klebsiella pneumoniae* strain 64917 plasmid p64917-KPC, complete sequence | *Klebsiella pneumoniae* | 1.12E+05 | 4.44E+05 | 99% | 0 | 99.96 | 154719 | 169419 | MF168405.1 |
|  | *Klebsiella pneumoniae* strain CRKP-30 plasmid pCRKP-30_KPC, complete sequence | *Klebsiella pneumoniae* | 1.12E+05 | 4.35E+05 | 96% | 0 | 99.96 | 154719 | 158524 | CP102634.1 |
|  | *Klebsiella pneumoniae* strain 08291 plasmid pW08291-KPC, complete sequence | *Klebsiella pneumoniae* | 1.12E+05 | 4.39E+05 | 99% | 0 | 99.96 | 154719 | 169804 | MN842295.1 |
|  | *Klebsiella pneumoniae* strain 246421 plasmid p246421-KPC, complete sequence | *Klebsiella pneumoniae* | 1.11E+05 | 3.86E+05 | 84% | 0 | 99.98 | 154719 | 145209 | MT810356.1 |
|  | Citrobacter werkmanii strain LYYSPS2 plasmid pLYYSPS2-3, complete sequence | Citrobacter werkmanii | 1.11E+05 | 1.85E+05 | 49% | 0 | 99.98 | 154719 | 87232 | MZ342958.1 |
|  | *Escherichia coli* strain HNEC55 plasmid pHNEC55, complete sequence | *Escherichia coli* | 1.11E+05 | 1.89E+05 | 53% | 0 | 99.97 | 154719 | 81498 | KT879914.1 |
|  | *Klebsiella pneumoniae* *subsp.* pneumoniae strain SH9 plasmid pSH9-CTX-TEM, complete sequence | *Klebsiella pneumoniae* *subsp.* pneumoniae | 1.11E+05 | 2.23E+05 | 53% | 0 | 99.96 | 154719 | 98684 | MH255829.1 |
|  | *Escherichia coli* strain XD35 plasmid pXD35004, complete sequence | *Escherichia coli* | 1.10E+05 | 1.91E+05 | 55% | 0 | 99.86 | 154719 | 85891 | CP089137.1 |
|  | *Klebsiella pneumoniae* strain WCHKP3 plasmid pKPC2_020003, complete sequence | *Klebsiella pneumoniae* | 1.10E+05 | 3.94E+05 | 100% | 0 | 99.98 | 154719 | 154957 | CP031720.1 |
|  | *Escherichia coli* strain 7A8 plasmid pHN7A8, complete sequence | *Escherichia coli* | 1.09E+05 | 1.98E+05 | 56% | 0 | 99.99 | 154719 | 76878 | JN232517.1 |
|  | Enterobacter cloacae strain CBG15936 plasmid pTEM-CBG, complete sequence | Enterobacter cloacae | 1.09E+05 | 1.84E+05 | 56% | 0 | 99.98 | 154719 | 75044 | CP046117.1 |
|  | *Escherichia coli* strain HNC02 plasmid pHNHNC02, complete sequence | *Escherichia coli* | 1.09E+05 | 1.95E+05 | 53% | 0 | 99.98 | 154719 | 76869 | MG197497.1 |
|  | *Escherichia coli* strain HZMPC32 plasmid pHNMPC32, complete sequence | *Escherichia coli* | 1.09E+05 | 1.81E+05 | 53% | 0 | 99.96 | 154719 | 74768 | MG197499.1 |
|  | *Klebsiella pneumoniae* p477Kp plasmid, complete sequence | *Klebsiella pneumoniae* | 1.09E+05 | 1.85E+05 | 56% | 0 | 99.96 | 154719 | 74768 | LN897475.2 |
|  | *Klebsiella pneumoniae* p397Kp plasmid, complete sequence | *Klebsiella pneumoniae* | 1.09E+05 | 1.98E+05 | 56% | 0 | 99.96 | 154719 | 76863 | LN897474.2 |
|  | *Klebsiella pneumoniae* strain BSI047 plasmid pBSI047-KPC2 | *Klebsiella pneumoniae* | 1.09E+05 | 3.99E+05 | 88% | 0 | 99.93 | 154719 | 139571 | MT269830.1 |
|  | *Klebsiella pneumoniae* strain SWU01 plasmid unnamed, complete sequence | *Klebsiella pneumoniae* | 1.09E+05 | 4.24E+05 | 95% | 0 | 99.97 | 154719 | 162552 | CP018455.1 |
|  | *Klebsiella pneumoniae* *subsp.* pneumoniae strain SH2 plasmid pSH2-85K-MDR, complete sequence | *Klebsiella pneumoniae* *subsp.* pneumoniae | 1.08E+05 | 3.60E+05 | 85% | 0 | 99.96 | 154719 | 149033 | MH643792.1 |
|  | *Klebsiella pneumoniae* strain IR12197_1 plasmid unnamed4, complete sequence | *Klebsiella pneumoniae* | 1.06E+05 | 2.94E+05 | 78% | 0 | 99.97 | 154719 | 159393 | CP097707.1 |
|  | *Klebsiella pneumoniae* *subsp.* pneumoniae strain RJBSI76-pV plasmid pRJBSI76-pV-3, complete sequence | *Klebsiella pneumoniae* *subsp.* pneumoniae | 1.06E+05 | 3.86E+05 | 94% | 0 | 99.99 | 154719 | 184751 | CP068687.1 |
|  | *Klebsiella pneumoniae* *subsp.* pneumoniae strain RJBSI76 plasmid pRJBSI76-3, complete sequence | *Klebsiella pneumoniae* *subsp.* pneumoniae | 1.06E+05 | 3.86E+05 | 94% | 0 | 99.99 | 154719 | 184748 | CP068692.1 |
|  | *Klebsiella pneumoniae* *subsp.* pneumoniae strain SH9 plasmid pSH9-KPC, complete sequence | *Klebsiella pneumoniae* *subsp.* pneumoniae | 1.05E+05 | 3.13E+05 | 53% | 0 | 99.91 | 154719 | 113941 | MH255827.1 |
|  | *Klebsiella pneumoniae* strain 150040X1B1 plasmid pCTXM65_150040X1B1, complete sequence | *Klebsiella pneumoniae* | 1.04E+05 | 4.10E+05 | 94% | 0 | 99.99 | 154719 | 149214 | CP101727.1 |
|  | *Klebsiella pneumoniae* strain WCHKP090357 plasmid pKPC2_090357, complete sequence | *Klebsiella pneumoniae* | 1.04E+05 | 4.07E+05 | 97% | 0 | 99.99 | 154719 | 149116 | CP066524.1 |
|  | *Klebsiella pneumoniae* strain CDI694 plasmid pCDI694-140.8, complete sequence | *Klebsiella pneumoniae* | 1.04E+05 | 3.51E+05 | 85% | 0 | 99.97 | 154719 | 140828 | CP077777.1 |
|  | *Klebsiella pneumoniae* strain KP18-2079 plasmid pKP18-2079_KPC, complete sequence | *Klebsiella pneumoniae* | 1.04E+05 | 1.86E+05 | 51% | 0 | 99.95 | 154719 | 186564 | MT090959.1 |
|  | *Klebsiella pneumoniae* strain 20150420184 plasmid p420184-KPC, complete sequence | *Klebsiella pneumoniae* | 1.03E+05 | 3.21E+05 | 73% | 0 | 99.98 | 154719 | 108236 | MT810359.1 |
|  | *Escherichia coli* strain OW1E2 plasmid pOW1E2a, complete sequence | *Escherichia coli* | 1.02E+05 | 2.24E+05 | 53% | 0 | 99.98 | 154719 | 108766 | CP067246.1 |
|  | *Escherichia coli* strain NT1N25 plasmid pNT1N25-76kb, complete sequence | *Escherichia coli* | 1.02E+05 | 1.93E+05 | 49% | 0 | 99.97 | 154719 | 76891 | CP075483.1 |
|  | *Klebsiella pneumoniae* strain 135077 plasmid p1_135077, complete sequence | *Klebsiella pneumoniae* | 1.02E+05 | 3.66E+05 | 91% | 0 | 100 | 154719 | 139526 | CP073293.1 |
|  | *Escherichia coli* strain fEC.1 plasmid pfEC.1-3, complete sequence | *Escherichia coli* | 1.02E+05 | 1.80E+05 | 50% | 0 | 99.95 | 154719 | 78319 | OK605583.1 |
|  | Escherichia fergusonii strain EFCF056 plasmid pEF02, complete sequence | Escherichia fergusonii | 1.02E+05 | 2.16E+05 | 53% | 0 | 99.9 | 154719 | 90871 | CP040807.1 |
|  | *Klebsiella pneumoniae* strain 8695 plasmid pFK8695-KPC-33, complete sequence | *Klebsiella pneumoniae* | 1.02E+05 | 3.83E+05 | 85% | 0 | 99.97 | 154719 | 143980 | CP085890.1 |
|  | *Klebsiella pneumoniae* strain 116753 plasmid p116753-KPC, complete sequence | *Klebsiella pneumoniae* | 1.01E+05 | 2.22E+05 | 50% | 0 | 99.64 | 154719 | 137873 | MN891682.1 |
|  | *Klebsiella pneumoniae* strain KP19-3138 plasmid pKP19-3138-4, complete sequence | *Klebsiella pneumoniae* | 1.01E+05 | 2.23E+05 | 49% | 0 | 99.64 | 154719 | 95171 | CP090620.1 |
|  | *Klebsiella pneumoniae* strain 7849 plasmid pKP7849_KPC, complete sequence | *Klebsiella pneumoniae* | 1.01E+05 | 3.95E+05 | 87% | 0 | 99.99 | 154719 | 148977 | MW478298.1 |
|  | *Escherichia coli* strain HNEC46 plasmid PHNEC46, complete sequence | *Escherichia coli* | 1.01E+05 | 1.41E+05 | 50% | 0 | 99.96 | 154719 | 74046 | KX503323.1 |
|  | *Klebsiella pneumoniae* *subsp.* pneumoniae strain HA2 plasmid pHA2-23-KPC, complete sequence | *Klebsiella pneumoniae* *subsp.* pneumoniae | 1.01E+05 | 3.59E+05 | 86% | 0 | 99.98 | 154719 | 148749 | MH643789.1 |
|  | *Klebsiella pneumoniae* strain Kp36 plasmid unnamed2, complete sequence | *Klebsiella pneumoniae* | 98769 | 3.81E+05 | 83% | 0 | 99.99 | 154719 | 142228 | CP047194.1 |
|  | *Escherichia coli* MH13-051M plasmid pMH13-051M_1 DNA, complete genome | *Escherichia coli* | 96529 | 2.23E+05 | 51% | 0 | 98.96 | 154719 | 111544 | AP018572.2 |
|  | *Klebsiella pneumoniae* strain CY814036 plasmid pCY814036-KPC2, complete sequence | *Klebsiella pneumoniae* | 95188 | 3.77E+05 | 90% | 0 | 99.99 | 154719 | 140105 | CP093153.1 |
|  | *Klebsiella pneumoniae* strain F127 plasmid pF127_1, complete sequence | *Klebsiella pneumoniae* | 92616 | 4.10E+05 | 96% | 0 | 99.96 | 154719 | 164501 | CP026141.1 |
|  | *Klebsiella pneumoniae* strain HZMPC43 plasmid pHNMPC43, complete sequence | *Klebsiella pneumoniae* | 92300 | 1.65E+05 | 47% | 0 | 99.98 | 154719 | 69666 | MG197501.1 |
|  | *Klebsiella pneumoniae* strain HZMPC51-2 plasmid pHNMPC51, complete sequence | *Klebsiella pneumoniae* | 92294 | 1.65E+05 | 47% | 0 | 99.98 | 154719 | 69654 | MG197500.1 |
|  | *Klebsiella pneumoniae* strain BSI074 plasmid pBSI074-KPC2 | *Klebsiella pneumoniae* | 92233 | 3.36E+05 | 82% | 0 | 99.98 | 154719 | 135737 | MT269848.1 |
|  | *Klebsiella pneumoniae* strain CRKP66R plasmid pCRKP66R-3, complete sequence | *Klebsiella pneumoniae* | 91175 | 3.35E+05 | 78% | 0 | 99.97 | 154719 | 128675 | CP063835.1 |
|  | *Klebsiella pneumoniae* plasmid pvirhs3_HS-142_NODE2, complete sequence | *Klebsiella pneumoniae* | 90821 | 2.01E+05 | 49% | 0 | 99.95 | 154719 | 130295 | OM975893.1 |
|  | *Klebsiella pneumoniae* strain 21072329 plasmid p21072329_1, complete sequence | *Klebsiella pneumoniae* | 89498 | 4.45E+05 | 93% | 0 | 99.96 | 154719 | 173108 | CP095235.1 |
|  | *Klebsiella pneumoniae* strain KPN35 plasmid pKPN35-1KPC, complete sequence | *Klebsiella pneumoniae* | 88322 | 3.96E+05 | 93% | 0 | 99.97 | 154719 | 153841 | MT920905.1 |
|  | *Klebsiella pneumoniae* strain JNKPN26 plasmid pJNKPN26_KPC, complete sequence | *Klebsiella pneumoniae* | 87619 | 3.26E+05 | 75% | 0 | 99.98 | 154719 | 126203 | MZ546615.1 |
|  | *Klebsiella pneumoniae* strain JNKPN26 plasmid pJNKPN26_KPC, complete sequence | *Klebsiella pneumoniae* | 87619 | 3.18E+05 | 75% | 0 | 99.98 | 154719 | 126203 | CP090204.1 |
| pCR-hvKP132-P2 | *Klebsiella pneumoniae* strain WCHKP090050 plasmid p1_090050, complete sequence | *Klebsiella pneumoniae* | 18399 | 18579 | 100% | 0 | 100 | 10060 | 10060 | CP043371.1 |
|  | *Klebsiella pneumoniae* strain LSH-KPN148 plasmid pLSH-KPN148-3, complete sequence | *Klebsiella pneumoniae* | 18205 | 18579 | 100% | 0 | 100 | 10060 | 10060 | CP040125.1 |
|  | *Klebsiella pneumoniae* strain KP18-2050 plasmid pKP18-2050-3, complete sequence | *Klebsiella pneumoniae* | 18000 | 18197 | 100% | 0 | 99.33 | 10060 | 10049 | CP082022.1 |
|  | *Klebsiella pneumoniae* *subsp.* pneumoniae strain DD02162 plasmid pDD02162-4, complete sequence | *Klebsiella pneumoniae* *subsp.* pneumoniae | 17932 | 18579 | 100% | 0 | 100 | 10060 | 10060 | CP087622.1 |
|  | *Klebsiella pneumoniae* strain FDAARGOS_444 plasmid unnamed3, complete sequence | *Klebsiella pneumoniae* | 17921 | 18573 | 100% | 0 | 99.99 | 10060 | 10061 | CP023944.1 |
|  | *Klebsiella pneumoniae* strain WCHKP115011 plasmid p2_115011, complete sequence | *Klebsiella pneumoniae* | 17821 | 18579 | 100% | 0 | 100 | 10060 | 10060 | CP089957.1 |
|  | *Klebsiella pneumoniae* strain SW1780 plasmid pB, complete sequence | *Klebsiella pneumoniae* | 17821 | 18579 | 100% | 0 | 100 | 10060 | 10060 | CP073304.1 |
|  | *Klebsiella pneumoniae* strain WCHKP7E2 plasmid p1_085072, complete sequence | *Klebsiella pneumoniae* | 17501 | 18579 | 100% | 0 | 100 | 10060 | 10060 | CP028801.2 |
|  | *Klebsiella pneumoniae* strain KP167 plasmid pKP167-10, complete sequence | *Klebsiella pneumoniae* | 17472 | 18579 | 100% | 0 | 100 | 10060 | 10060 | CP098761.1 |
|  | *Klebsiella pneumoniae* strain 135077 plasmid p2_135077, complete sequence | *Klebsiella pneumoniae* | 17472 | 18579 | 100% | 0 | 100 | 10060 | 10060 | CP073294.1 |
|  | *Klebsiella pneumoniae* strain KPN142 plasmid pn142_2, complete sequence | *Klebsiella pneumoniae* | 17398 | 18579 | 100% | 0 | 100 | 10060 | 10060 | CP053877.1 |
|  | *Klebsiella pneumoniae* strain XHKP6 plasmid pXHKP6-2, complete sequence | *Klebsiella pneumoniae* | 17248 | 18614 | 100% | 0 | 99.99 | 10060 | 10098 | CP066889.1 |
|  | *Klebsiella pneumoniae* strain KP19-2029 plasmid pKP19-2029-Col, complete sequence | *Klebsiella pneumoniae* | 17224 | 18579 | 100% | 0 | 100 | 10060 | 10060 | CP047162.1 |
|  | *Klebsiella pneumoniae* strain ZRKP03 plasmid unnamed4, complete sequence | *Klebsiella pneumoniae* | 17163 | 18546 | 100% | 0 | 99.94 | 10060 | 10060 | CP050346.1 |
|  | *Klebsiella pneumoniae* strain WCHKP090045 plasmid p1_090045, complete sequence | *Klebsiella pneumoniae* | 17047 | 18579 | 100% | 0 | 100 | 10060 | 10060 | CP043367.1 |
|  | *Klebsiella pneumoniae* strain KP4863 plasmid pKP4863-2, complete sequence |  | 17016 | 34033 | 100% | 0 | 97.21 | 10060 | 20110 | CP102843.1 |
|  | *Klebsiella pneumoniae* GSU10-3 plasmid pGSU10-3-4 DNA, complete genome | *Klebsiella pneumoniae* | 16750 | 18579 | 100% | 0 | 100 | 10060 | 10060 | AP018675.1 |
|  | *Klebsiella pneumoniae* *subsp.* pneumoniae strain kpn-hnqyy plasmid unnamed3, complete sequence | *Klebsiella pneumoniae* *subsp.* pneumoniae | 16560 | 18579 | 100% | 0 | 100 | 10060 | 10060 | CP074119.1 |
|  | *Klebsiella pneumoniae* strain 21080237 plasmid p21080237_6, complete sequence | *Klebsiella pneumoniae* | 16541 | 18573 | 100% | 0 | 99.99 | 10060 | 10060 | CP095246.1 |
|  | *Klebsiella pneumoniae* strain BSI073 plasmid pBSI073-KPC2 | *Klebsiella pneumoniae* | 16366 | 18594 | 100% | 0 | 100 | 10060 | 118923 | MT269846.1 |
|  | *Klebsiella pneumoniae* strain KP18-1 plasmid pKP18-1-2, complete sequence | *Klebsiella pneumoniae* | 16070 | 18245 | 99% | 0 | 99.82 | 10060 | 10049 | CP082002.1 |
|  | *Klebsiella pneumoniae* strain XHKP53 plasmid pXHKP53-2, complete sequence | *Klebsiella pneumoniae* | 16026 | 18064 | 100% | 0 | 99.35 | 10060 | 10013 | CP066893.1 |
|  | *Klebsiella pneumoniae* isolate 91eed288-b809-11e8-aae5-3c4a9275d6c8 genome assembly, chromosome: 1 | *Klebsiella pneumoniae* | 15993 | 19203 | 100% | 0 | 100 | 10060 | 5510473 | LR596808.1 |
|  | *Klebsiella pneumoniae* strain 21072329 plasmid p21072329_4, complete sequence | *Klebsiella pneumoniae* | 15911 | 18579 | 100% | 0 | 100 | 10060 | 10060 | CP095238.1 |
|  | *Klebsiella pneumoniae* strain WCHKP8F4 plasmid p1_095084, complete sequence | *Klebsiella pneumoniae* | 15645 | 18579 | 100% | 0 | 100 | 10060 | 10060 | CP027065.3 |
|  | *Klebsiella pneumoniae* strain 140253 plasmid p3_140253, complete sequence | *Klebsiella pneumoniae* | 15640 | 18579 | 100% | 0 | 100 | 10060 | 10060 | CP097630.1 |
|  | *Klebsiella pneumoniae* *subsp.* pneumoniae strain SCKP020143 plasmid p3_020143, complete sequence | *Klebsiella pneumoniae* *subsp.* pneumoniae | 15603 | 18579 | 100% | 0 | 100 | 10060 | 10060 | CP028545.2 |
|  | *Klebsiella pneumoniae* strain 1632 plasmid p1632-3, complete sequence | *Klebsiella pneumoniae* | 15570 | 18579 | 100% | 0 | 100 | 10060 | 10060 | CP084500.1 |
|  | *Klebsiella pneumoniae* plasmid unnamed, complete sequence | *Klebsiella pneumoniae* | 15477 | 18579 | 100% | 0 | 100 | 10060 | 10060 | MK181634.1 |
|  | *Klebsiella pneumoniae* *subsp.* pneumoniae strain WCHKP020120 plasmid p2_020120, complete sequence | *Klebsiella pneumoniae* *subsp.* pneumoniae | 15418 | 18579 | 100% | 0 | 100 | 10060 | 10060 | CP043360.1 |
|  | Serratia marcescens Sm4 plasmid pSm4Col DNA, complete sequence | Serratia marcescens | 15407 | 18579 | 100% | 0 | 100 | 10060 | 10060 | LC543864.1 |
|  | *Klebsiella pneumoniae* strain WCHKP649 plasmid p1_095649, complete sequence | *Klebsiella pneumoniae* | 15271 | 18579 | 100% | 0 | 100 | 10060 | 10060 | CP026582.3 |
|  | *Klebsiella pneumoniae* strain 160111 plasmid p10K_L111, complete sequence | *Klebsiella pneumoniae* | 15139 | 18579 | 100% | 0 | 100 | 10060 | 10060 | CP030130.1 |
|  | *Klebsiella pneumoniae* *subsp.* pneumoniae strain DD01754 plasmid pDD01754-4, complete sequence | *Klebsiella pneumoniae* *subsp.* pneumoniae | 15080 | 18540 | 100% | 0 | 100 | 10060 | 10061 | CP087649.1 |
|  | *Klebsiella pneumoniae* strain LSH-KPN25 plasmid pLSH-KPN25-2, complete sequence | *Klebsiella pneumoniae* | 15066 | 18579 | 100% | 0 | 100 | 10060 | 10060 | CP040181.1 |
|  | *Klebsiella pneumoniae* strain XHKPN391 plasmid pXHKPN391-2, complete sequence | *Klebsiella pneumoniae* | 14951 | 18579 | 100% | 0 | 100 | 10060 | 10060 | CP066917.1 |
|  | *Klebsiella pneumoniae* strain JX-CR-hvKP-2 plasmid pJX2-4, complete sequence | *Klebsiella pneumoniae* | 14931 | 18579 | 100% | 0 | 100 | 10060 | 10060 | CP064250.1 |
|  | *Klebsiella pneumoniae* *subsp.* pneumoniae strain KP21 plasmid p21_2, complete sequence | *Klebsiella pneumoniae* *subsp.* pneumoniae | 14925 | 18573 | 100% | 0 | 99.99 | 10060 | 10061 | CP101544.1 |
|  | *Klebsiella pneumoniae* strain KPCZA02 plasmid pKPCZA02_1, complete sequence | *Klebsiella pneumoniae* | 14272 | 18577 | 99% | 0 | 100 | 10060 | 10059 | CP058227.1 |
|  | *Klebsiella pneumoniae* strain KP18-2073 plasmid pKP18-2073-3, complete sequence | *Klebsiella pneumoniae* | 14209 | 18197 | 100% | 0 | 100 | 10060 | 10049 | CP082027.1 |
|  | *Klebsiella pneumoniae* *subsp.* pneumoniae strain WCHKP015093 plasmid p2_015093, complete sequence | *Klebsiella pneumoniae* *subsp.* pneumoniae | 14113 | 18579 | 100% | 0 | 100 | 10060 | 10060 | CP036303.1 |
|  | *Klebsiella pneumoniae* strain HvKp-su1 plasmid unnamed2, complete sequence | *Klebsiella pneumoniae* | 14087 | 18581 | 100% | 0 | 100 | 10060 | 10061 | CP092719.1 |
|  | *Klebsiella pneumoniae* strain GD21SC417 plasmid pHNGS471-4, complete sequence | *Klebsiella pneumoniae* | 14079 | 18579 | 100% | 0 | 100 | 10060 | 10060 | CP089513.1 |
|  | *Klebsiella pneumoniae* strain KPN23 plasmid pKPN23_2, complete sequence | *Klebsiella pneumoniae* | 14078 | 18579 | 100% | 0 | 100 | 10060 | 10060 | CP089864.1 |
|  | *Klebsiella pneumoniae* strain ZRKP04 plasmid unnamed4, complete sequence | *Klebsiella pneumoniae* | 13884 | 18191 | 100% | 0 | 99.12 | 10060 | 10049 | CP050340.1 |
|  | *Klebsiella pneumoniae* strain KP55 plasmid pKPC-5504, complete sequence | *Klebsiella pneumoniae* | 13780 | 18572 | 100% | 0 | 99.99 | 10060 | 10059 | OL891654.1 |
|  | *Klebsiella pneumoniae* *subsp.* pneumoniae strain WCHKP020039 plasmid p1_020039, complete sequence | *Klebsiella pneumoniae* *subsp.* pneumoniae | 13605 | 18579 | 100% | 0 | 100 | 10060 | 10060 | CP043346.1 |
|  | *Klebsiella pneumoniae* strain KP69 plasmid p69-3, complete sequence | *Klebsiella pneumoniae* | 13598 | 18579 | 100% | 0 | 100 | 10060 | 10060 | CP025459.1 |
|  | *Klebsiella pneumoniae* strain ZRKP02 plasmid unnamed4, complete sequence | *Klebsiella pneumoniae* | 13411 | 18579 | 100% | 0 | 100 | 10060 | 10060 | CP050352.1 |
|  | *Klebsiella pneumoniae* strain WCHKP020030 plasmid p1_020030, complete sequence | *Klebsiella pneumoniae* | 13092 | 18579 | 100% | 0 | 100 | 10060 | 10060 | CP028788.2 |
|  | *Klebsiella pneumoniae* strain KP2509 plasmid pKP2509-5, complete sequence | *Klebsiella pneumoniae* | 13027 | 18579 | 100% | 0 | 100 | 10060 | 10060 | CP065951.1 |
|  | *Klebsiella pneumoniae* *subsp.* pneumoniae strain DD02172 plasmid pDD02172-5, complete sequence | *Klebsiella pneumoniae* *subsp.* pneumoniae | 12802 | 18579 | 100% | 0 | 100 | 10060 | 10060 | CP087616.1 |
|  | *Klebsiella pneumoniae* strain C789 plasmid unnamed1, complete sequence | *Klebsiella pneumoniae* | 12589 | 18573 | 100% | 0 | 100 | 10060 | 10061 | CP034418.1 |
|  | *Klebsiella pneumoniae* strain WCHKP3 plasmid p2_020003, complete sequence | *Klebsiella pneumoniae* | 12484 | 18579 | 100% | 0 | 100 | 10060 | 10060 | CP031718.1 |
|  | *Klebsiella pneumoniae* strain WCHKP115069 plasmid p2_115069, complete sequence | *Klebsiella pneumoniae* | 12438 | 18579 | 100% | 0 | 100 | 10060 | 10060 | CP033403.1 |
|  | *Klebsiella pneumoniae* strain KP46 plasmid pKP46_5, complete sequence | *Klebsiella pneumoniae* | 12412 | 18579 | 100% | 0 | 100 | 10060 | 10060 | CP090131.1 |
|  | *Klebsiella pneumoniae* strain KP18-8 plasmid pKP18-8-4, complete sequence | *Klebsiella pneumoniae* | 12227 | 18573 | 100% | 0 | 99.98 | 10060 | 10060 | CP082008.1 |
|  | *Klebsiella pneumoniae* strain WCHKP2 plasmid p1_020002, complete sequence | *Klebsiella pneumoniae* | 12028 | 18579 | 100% | 0 | 100 | 10060 | 10060 | CP028539.3 |
|  | *Klebsiella pneumoniae* strain SH12 plasmid pSH12_3, complete sequence | *Klebsiella pneumoniae* | 11810 | 18579 | 100% | 0 | 100 | 10060 | 10060 | CP040836.1 |
|  | *Klebsiella pneumoniae* strain JX-CR-hvKP-7 plasmid pJX7-4, complete sequence | *Klebsiella pneumoniae* | 11801 | 18579 | 100% | 0 | 100 | 10060 | 10060 | CP064227.1 |
|  | *Klebsiella pneumoniae* strain FO15 plasmid unnamed4, complete sequence | *Klebsiella pneumoniae* | 11795 | 27510 | 100% | 0 | 99.98 | 10060 | 14945 | CP073006.1 |
|  | *Klebsiella pneumoniae* strain 16HN-263 plasmid p16HN-263_2, complete sequence | *Klebsiella pneumoniae* | 11771 | 18579 | 100% | 0 | 100 | 10060 | 10060 | CP045265.1 |
|  | *Klebsiella pneumoniae* strain KP20194c4 plasmid pKP20194c4-p4, complete sequence | *Klebsiella pneumoniae* | 11716 | 18458 | 100% | 0 | 100 | 10060 | 11971 | CP054748.1 |
|  | *Klebsiella pneumoniae* strain KP18-2079 plasmid pKP18-2079_11kb, complete sequence | *Klebsiella pneumoniae* | 11716 | 18580 | 100% | 0 | 99.98 | 10060 | 11970 | MT090962.1 |
|  | *Klebsiella pneumoniae* strain WCHKP36 plasmid p2_020036, complete sequence | *Klebsiella pneumoniae* | 11716 | 18595 | 100% | 0 | 100 | 10060 | 12746 | CP028580.2 |
|  | *Klebsiella pneumoniae* strain L482 plasmid p5_L382, complete sequence | *Klebsiella pneumoniae* | 11716 | 18580 | 100% | 0 | 100 | 10060 | 11970 | CP033964.1 |
|  | *Klebsiella pneumoniae* isolate 97706988-b809-11e8-aae5-3c4a9275d6c8 genome assembly, chromosome: 1 | *Klebsiella pneumoniae* | 11716 | 19114 | 100% | 0 | 100 | 10060 | 5652578 | LR596813.1 |
|  | *Klebsiella pneumoniae* strain KP0079 plasmid pKP0079-4, complete sequence |  | 11716 | 37160 | 100% | 0 | 99.98 | 10060 | 23940 | CP102840.1 |
|  | *Klebsiella pneumoniae* strain S270v plasmid pS270V-3, complete sequence | *Klebsiella pneumoniae* | 11716 | 18579 | 100% | 0 | 100 | 10060 | 11970 | CP102195.1 |
|  | *Klebsiella pneumoniae* strain S234 plasmid pS234-3, complete sequence | *Klebsiella pneumoniae* | 11716 | 18580 | 100% | 0 | 99.98 | 10060 | 11970 | CP102189.1 |
|  | *Klebsiella pneumoniae* strain hvKP340 plasmid unnamed5, complete sequence | *Klebsiella pneumoniae* | 11716 | 18579 | 100% | 0 | 100 | 10060 | 11970 | CP101781.1 |
|  | *Klebsiella pneumoniae* strain hvKP841 plasmid unnamed3, complete sequence | *Klebsiella pneumoniae* | 11716 | 18579 | 100% | 0 | 100 | 10060 | 11970 | CP101787.1 |
|  | *Klebsiella pneumoniae* strain hvKP319 plasmid unnamed4, complete sequence | *Klebsiella pneumoniae* | 11716 | 18579 | 100% | 0 | 100 | 10060 | 11970 | CP101768.1 |
|  | *Klebsiella pneumoniae* strain KP15 plasmid unnamed3, complete sequence | *Klebsiella pneumoniae* | 11716 | 18579 | 100% | 0 | 100 | 10060 | 99065 | CP087145.1 |
|  | *Escherichia coli* strain CR-HvKP1TC-3 plasmid pCR-HvKP1TC-3_p4, complete sequence | *Escherichia coli* | 11716 | 18575 | 99% | 0 | 100 | 10060 | 11970 | OM001473.1 |
|  | *Klebsiella pneumoniae* strain WSCRKP plasmid pWSCRKP-4, complete sequence | *Klebsiella pneumoniae* | 11716 | 18594 | 100% | 0 | 100 | 10060 | 15931 | CP091072.1 |
|  | *Klebsiella pneumoniae* strain KP697 plasmid unnamed4, complete sequence | *Klebsiella pneumoniae* | 11716 | 18580 | 100% | 0 | 100 | 10060 | 11970 | CP066155.1 |
|  | *Klebsiella pneumoniae* *subsp.* pneumoniae strain KPN857 plasmid pD, complete sequence | *Klebsiella pneumoniae* *subsp.* pneumoniae | 11716 | 18580 | 100% | 0 | 99.98 | 10060 | 11970 | CP090436.1 |
|  | *Klebsiella pneumoniae* strain LZKP00003 plasmid pZR1, complete sequence | *Klebsiella pneumoniae* | 11716 | 18580 | 100% | 0 | 100 | 10060 | 11970 | CP089992.1 |
|  | *Klebsiella pneumoniae* strain 50700 plasmid p50700-12.0, complete sequence | *Klebsiella pneumoniae* | 11716 | 18393 | 100% | 0 | 100 | 10060 | 11972 | CP088993.1 |
|  | *Klebsiella pneumoniae* strain 8695 plasmid p4, complete sequence | *Klebsiella pneumoniae* | 11716 | 37104 | 100% | 0 | 100 | 10060 | 23957 | CP085893.1 |
|  | *Klebsiella pneumoniae* strain 12 plasmid pKP12_5, complete sequence | *Klebsiella pneumoniae* | 11716 | 18558 | 100% | 0 | 99.98 | 10060 | 11970 | CP082769.1 |
|  | *Klebsiella pneumoniae* strain KP18-2113 plasmid pKP18-2113-3, complete sequence | *Klebsiella pneumoniae* | 11716 | 18580 | 100% | 0 | 100 | 10060 | 11969 | CP082028.1 |
|  | *Klebsiella pneumoniae* strain KP-CT77 plasmid pCT77-tetA, complete sequence | *Klebsiella pneumoniae* | 11716 | 18537 | 100% | 0 | 99.98 | 10060 | 92645 | CP080306.1 |
|  | *Klebsiella pneumoniae* strain 19PDR22 plasmid p7, complete sequence | *Klebsiella pneumoniae* | 11716 | 18579 | 100% | 0 | 100 | 10060 | 11970 | CP076551.1 |
|  | *Klebsiella pneumoniae* strain DD521 plasmid pDD521.4, complete sequence | *Klebsiella pneumoniae* | 11716 | 18579 | 100% | 0 | 99.98 | 10060 | 11969 | CP075320.1 |
|  | *Escherichia coli* strain CR-HvKP5TC plasmid pCR-HvKP5TC_Vir-p4, complete sequence | *Escherichia coli* | 11716 | 18579 | 100% | 0 | 100 | 10060 | 190129 | MW598247.1 |
|  | *Escherichia coli* strain CR-HvKP3TC plasmid pCR-HvKP3TC_p4, complete sequence | *Escherichia coli* | 11716 | 18580 | 100% | 0 | 100 | 10060 | 11970 | MW598243.1 |
|  | *Escherichia coli* strain CR-HvKP1TC plasmid pCR-HvKP1TC_Vir-p4, complete sequence | *Escherichia coli* | 11716 | 18579 | 100% | 0 | 100 | 10060 | 190129 | MW598240.1 |
|  | *Klebsiella pneumoniae* strain CR-HvKP3 plasmid p4-CR-HvKP3, complete sequence | *Klebsiella pneumoniae* | 11716 | 18580 | 100% | 0 | 100 | 10060 | 11970 | MW598236.1 |
|  | *Klebsiella pneumoniae* strain KP55 plasmid pKP55_5, complete sequence | *Klebsiella pneumoniae* | 11716 | 18580 | 100% | 0 | 100 | 10060 | 11970 | CP055299.1 |
|  | *Klebsiella pneumoniae* strain BSI058 plasmid pBSI058-KPC2 | *Klebsiella pneumoniae* | 11716 | 18579 | 100% | 0 | 100 | 10060 | 114712 | MT269836.1 |
|  | *Klebsiella pneumoniae* strain 015625 plasmid p2_015625, complete sequence | *Klebsiella pneumoniae* | 11716 | 17042 | 91% | 0 | 99.98 | 10060 | 11904 | CP033392.2 |
|  | *Klebsiella pneumoniae* *subsp.* pneumoniae strain RJBSI76 plasmid pRJBSI76-4, complete sequence | *Klebsiella pneumoniae* *subsp.* pneumoniae | 11716 | 18573 | 100% | 0 | 99.98 | 10060 | 10206 | CP068693.1 |
|  | *Klebsiella pneumoniae* strain WCHKP090361 plasmid p2_090361, complete sequence | *Klebsiella pneumoniae* | 11716 | 18580 | 100% | 0 | 99.98 | 10060 | 11970 | CP066532.1 |
|  | *Klebsiella pneumoniae* strain WCHKP090329 plasmid p2_090329, complete sequence | *Klebsiella pneumoniae* | 11716 | 18579 | 100% | 0 | 99.98 | 10060 | 11970 | CP066521.1 |
|  | *Klebsiella pneumoniae* strain CRKP52R plasmid pCRKP52R-4-tetA, complete sequence | *Klebsiella pneumoniae* | 11716 | 18557 | 100% | 0 | 99.98 | 10060 | 99066 | CP066252.1 |
|  | *Klebsiella pneumoniae* strain JX-CR-hvKP-1 plasmid pJX1-4, complete sequence | *Klebsiella pneumoniae* | 11716 | 18579 | 100% | 0 | 100 | 10060 | 11970 | CP064256.1 |
|  | *Klebsiella pneumoniae* strain JX-CR-hvKP-4 plasmid pJX4-4, complete sequence | *Klebsiella pneumoniae* | 11716 | 18580 | 100% | 0 | 100 | 10060 | 11970 | CP064239.1 |
|  | *Klebsiella pneumoniae* strain FRPDR plasmid pFRPDR_5, complete sequence | *Klebsiella pneumoniae* | 11716 | 18579 | 100% | 0 | 100 | 10060 | 11970 | CP063764.1 |
|  | *Klebsiella pneumoniae* strain KP18-238 plasmid pKP18-238-4, complete sequence | *Klebsiella pneumoniae* | 10334 | 20668 | 100% | 0 | 100 | 10060 | 11192 | CP082017.1 |
| pCR-hvKP132-P3 | *Klebsiella pneumoniae* strain RIVM_C014947 plasmid pRIVM_C014947_4 | *Klebsiella pneumoniae* | 10305 | 10305 | 99% | 0 | 99.96 | 5596 | 5596 | MT560067.1 |
|  | *Klebsiella pneumoniae* *subsp.* pneumoniae strain SCKP020079 plasmid p2_020079, complete sequence | *Klebsiella pneumoniae* *subsp.* pneumoniae | 10299 | 10299 | 99% | 0 | 100 | 5596 | 5596 | CP029380.1 |
|  | *Klebsiella pneumoniae* strain WCHKP040035 plasmid p2_040035, complete sequence | *Klebsiella pneumoniae* | 10298 | 10298 | 99% | 0 | 100 | 5596 | 5596 | CP028795.2 |
|  | *Klebsiella pneumoniae* *subsp.* pneumoniae strain DD02280 plasmid pDD02280-4, complete sequence | *Klebsiella pneumoniae* *subsp.* pneumoniae | 10238 | 10336 | 100% | 0 | 100 | 5596 | 5596 | CP087627.1 |
|  | *Klebsiella pneumoniae* strain KP20194c5 plasmid pKP20194c5-p5, complete sequence | *Klebsiella pneumoniae* | 10159 | 10330 | 100% | 0 | 99.98 | 5596 | 5596 | CP054743.1 |
|  | *Klebsiella pneumoniae* strain 150040X1B1 plasmid p2_150040X1B1, complete sequence | *Klebsiella pneumoniae* | 10122 | 10336 | 100% | 0 | 100 | 5596 | 5596 | CP101730.1 |
|  | *Klebsiella pneumoniae* strain WCHKP8F4 plasmid p2_095084, complete sequence | *Klebsiella pneumoniae* | 9982 | 10336 | 100% | 0 | 100 | 5596 | 5596 | CP027066.3 |
|  | *Klebsiella pneumoniae* strain WCHKP020115 plasmid p2_020115, complete sequence | *Klebsiella pneumoniae* | 9961 | 10336 | 100% | 0 | 100 | 5596 | 5596 | CP043356.1 |
|  | *Klebsiella pneumoniae* strain WCHKP090374 plasmid p2_090374, complete sequence | *Klebsiella pneumoniae* | 9958 | 10328 | 100% | 0 | 99.98 | 5596 | 5595 | CP066538.1 |
|  | *Klebsiella pneumoniae* isolate 392 genome assembly, plasmid: P4 | *Klebsiella pneumoniae* | 9939 | 10293 | 100% | 0 | 99.91 | 5596 | 5599 | OW848883.1 |
|  | *Klebsiella pneumoniae* isolate 392 genome assembly, plasmid: P3 | *Klebsiella pneumoniae* | 9939 | 10293 | 100% | 0 | 99.91 | 5596 | 5599 | OW849089.1 |
|  | *Klebsiella pneumoniae* strain KP20194a plasmid pKP20194a-p5, complete sequence | *Klebsiella pneumoniae* | 9925 | 10330 | 100% | 0 | 99.98 | 5596 | 5596 | CP054785.1 |
|  | *Klebsiella pneumoniae* *subsp.* pneumoniae strain WCHKP020120 plasmid p3_020120, complete sequence | *Klebsiella pneumoniae* *subsp.* pneumoniae | 9893 | 10336 | 100% | 0 | 100 | 5596 | 5596 | CP043361.1 |
|  | *Klebsiella pneumoniae* isolate 91eed288-b809-11e8-aae5-3c4a9275d6c8 genome assembly, chromosome: 1 | *Klebsiella pneumoniae* | 9889 | 10761 | 100% | 0 | 99.98 | 5596 | 5510473 | LR596808.1 |
|  | *Klebsiella pneumoniae* strain SH12 plasmid pSH12_5, complete sequence | *Klebsiella pneumoniae* | 9889 | 10336 | 100% | 0 | 100 | 5596 | 5596 | CP040838.1 |
|  | *Klebsiella pneumoniae* strain JX-CR-hvKP-10 plasmid pJX10-4, complete sequence | *Klebsiella pneumoniae* | 9873 | 10336 | 100% | 0 | 100 | 5596 | 5596 | CP064262.1 |
|  | *Klebsiella pneumoniae* isolate 392 genome assembly, plasmid: P4 | *Klebsiella pneumoniae* | 9793 | 10293 | 100% | 0 | 99.91 | 5596 | 5599 | OW849049.1 |
|  | *Klebsiella pneumoniae* strain ZRKP01 plasmid unnamed5, complete sequence | *Klebsiella pneumoniae* | 9736 | 10336 | 100% | 0 | 100 | 5596 | 5596 | CP050359.1 |
|  | *Klebsiella pneumoniae* strain KPWX136 plasmid pF, complete sequence | *Klebsiella pneumoniae* | 9705 | 10336 | 100% | 0 | 100 | 5596 | 5596 | CP069175.1 |
|  | *Klebsiella pneumoniae* strain KP20194a2 plasmid pKP20194a2-p5, complete sequence | *Klebsiella pneumoniae* | 9686 | 10330 | 100% | 0 | 99.98 | 5596 | 5596 | CP054779.1 |
|  | *Klebsiella pneumoniae* strain FK 6768 plasmid unnamed5, complete sequence | *Klebsiella pneumoniae* | 9655 | 10336 | 100% | 0 | 100 | 5596 | 5596 | CP065559.1 |
|  | *Klebsiella pneumoniae* strain hvKP841 plasmid unnamed5, complete sequence | *Klebsiella pneumoniae* | 9638 | 10336 | 100% | 0 | 100 | 5596 | 5596 | CP101789.1 |
|  | *Klebsiella pneumoniae* strain WCHKP090045 plasmid p2_090045, complete sequence | *Klebsiella pneumoniae* | 9627 | 10336 | 100% | 0 | 100 | 5596 | 5596 | CP043368.1 |
|  | *Klebsiella pneumoniae* strain CP19 plasmid unnamed2, complete sequence | *Klebsiella pneumoniae* | 9511 | 10330 | 100% | 0 | 100 | 5596 | 5597 | CP073353.1 |
|  | *Klebsiella pneumoniae* strain WCHKP020037 plasmid p3_020037, complete sequence | *Klebsiella pneumoniae* | 9448 | 10336 | 100% | 0 | 100 | 5596 | 5596 | CP036375.1 |
|  | *Klebsiella pneumoniae* strain WCHKP090357 plasmid p3_090357, complete sequence | *Klebsiella pneumoniae* | 9382 | 10336 | 100% | 0 | 100 | 5596 | 5596 | CP066527.1 |
|  | *Klebsiella pneumoniae* strain 21080937 plasmid p21080937_9, complete sequence | *Klebsiella pneumoniae* | 9317 | 10336 | 100% | 0 | 100 | 5596 | 5596 | CP095262.1 |
|  | *Klebsiella pneumoniae* strain XH1508 plasmid pXH1508-5, complete sequence | *Klebsiella pneumoniae* | 9243 | 10336 | 100% | 0 | 100 | 5596 | 5596 | CP092791.1 |
|  | *Klebsiella pneumoniae* strain KP-CT77 plasmid unnamed2, complete sequence | *Klebsiella pneumoniae* | 9178 | 10336 | 100% | 0 | 100 | 5596 | 5596 | CP080308.1 |
|  | *Klebsiella pneumoniae* strain KP20194c3 plasmid pKP20194c3-p5, complete sequence | *Klebsiella pneumoniae* | 9140 | 10330 | 100% | 0 | 99.98 | 5596 | 5596 | CP054755.1 |
|  | *Klebsiella pneumoniae* strain hvKP319 plasmid unnamed5, complete sequence | *Klebsiella pneumoniae* | 9140 | 10336 | 100% | 0 | 100 | 5596 | 5596 | CP101769.1 |
|  | *Klebsiella pneumoniae* strain WCHKP3 plasmid p3_020003, complete sequence | *Klebsiella pneumoniae* | 9081 | 10336 | 100% | 0 | 100 | 5596 | 5596 | CP031719.1 |
|  | *Klebsiella pneumoniae* strain KP20194c plasmid pKP20194c-p5, complete sequence | *Klebsiella pneumoniae* | 8914 | 10330 | 100% | 0 | 100 | 5596 | 5596 | CP054761.1 |
|  | *Klebsiella pneumoniae* strain CRKP66R plasmid pCRKP66R-6, complete sequence | *Klebsiella pneumoniae* | 8914 | 10336 | 100% | 0 | 100 | 5596 | 5596 | CP063838.1 |
|  | *Klebsiella pneumoniae* strain ZRKP03 plasmid unnamed5, complete sequence | *Klebsiella pneumoniae* | 8909 | 10336 | 100% | 0 | 100 | 5596 | 5596 | CP050347.1 |
|  | *Klebsiella pneumoniae* strain 21091025 plasmid p21091025_4, complete sequence | *Klebsiella pneumoniae* | 8876 | 10336 | 100% | 0 | 100 | 5596 | 5596 | CP095268.1 |
|  | *Klebsiella pneumoniae* strain KP1878 plasmid p1878-6k, complete sequence | *Klebsiella pneumoniae* | 8874 | 10343 | 100% | 0 | 99.98 | 5596 | 6796 | CP073000.1 |
|  | *Klebsiella pneumoniae* strain 16HN-263 plasmid p16HN-263_3, complete sequence | *Klebsiella pneumoniae* | 8837 | 10336 | 100% | 0 | 100 | 5596 | 5596 | CP045266.1 |
|  | *Klebsiella pneumoniae* strain KP51248 plasmid p51248_2, complete sequence | *Klebsiella pneumoniae* | 8817 | 10182 | 100% | 0 | 99.57 | 5596 | 5598 | CP070566.1 |
|  | *Klebsiella pneumoniae* strain S234 plasmid pS234-5, complete sequence | *Klebsiella pneumoniae* | 8807 | 10336 | 100% | 0 | 100 | 5596 | 5596 | CP102191.1 |
|  | *Klebsiella pneumoniae* strain KP20194b2 plasmid pKP20194b2-p5, complete sequence | *Klebsiella pneumoniae* | 8765 | 10330 | 100% | 0 | 99.98 | 5596 | 5596 | CP054767.1 |
|  | *Klebsiella pneumoniae* strain 140253 plasmid p4_140253, complete sequence | *Klebsiella pneumoniae* | 8719 | 10328 | 100% | 0 | 100 | 5596 | 5595 | CP097631.1 |
|  | *Klebsiella pneumoniae* isolate 13 genome assembly, plasmid: P5 | *Klebsiella pneumoniae* | 8698 | 10184 | 100% | 0 | 99.64 | 5596 | 5602 | OW968430.1 |
|  | *Klebsiella pneumoniae* strain KP18-2079 plasmid pKP18-2079_5kb, complete sequence | *Klebsiella pneumoniae* | 8610 | 10336 | 100% | 0 | 100 | 5596 | 5596 | MT090963.1 |
|  | *Klebsiella pneumoniae* strain CR-HvKP3 plasmid p5-CR-HvKP3, complete sequence | *Klebsiella pneumoniae* | 8588 | 10336 | 100% | 0 | 100 | 5596 | 5596 | MW598235.1 |
|  | *Klebsiella pneumoniae* strain CR-HvKP5 plasmid pCR-HvKP5-p5, complete sequence | *Klebsiella pneumoniae* | 8580 | 10336 | 100% | 0 | 100 | 5596 | 5596 | CP040550.1 |
|  | *Klebsiella pneumoniae* *subsp.* pneumoniae strain DD01304 plasmid pDD01304-4, complete sequence | *Klebsiella pneumoniae* *subsp.* pneumoniae | 8541 | 10336 | 100% | 0 | 100 | 5596 | 5596 | CP087610.1 |
|  | *Klebsiella pneumoniae* strain 36 plasmid pKP36_6, complete sequence | *Klebsiella pneumoniae* | 8530 | 10336 | 100% | 0 | 100 | 5596 | 5596 | CP082764.1 |
|  | *Klebsiella pneumoniae* *subsp.* pneumoniae strain DD02341 plasmid pDD02341-4, complete sequence | *Klebsiella pneumoniae* *subsp.* pneumoniae | 8525 | 10336 | 100% | 0 | 100 | 5596 | 5596 | CP087633.1 |
|  | *Klebsiella pneumoniae* strain XH1507 plasmid pXH1507-6, complete sequence | *Klebsiella pneumoniae* | 8525 | 10336 | 100% | 0 | 100 | 5596 | 5596 | CP092799.1 |
|  | *Klebsiella pneumoniae* strain FO15 plasmid unnamed2, complete sequence | *Klebsiella pneumoniae* | 8466 | 17396 | 100% | 0 | 100 | 5596 | 9417 | CP073004.1 |
|  | *Klebsiella pneumoniae* strain 21072329 plasmid p21072329_5, complete sequence | *Klebsiella pneumoniae* | 8395 | 10336 | 100% | 0 | 100 | 5596 | 5596 | CP095239.1 |
|  | *Klebsiella pneumoniae* strain KP69 plasmid p69-4, complete sequence | *Klebsiella pneumoniae* | 8390 | 10336 | 100% | 0 | 100 | 5596 | 5596 | CP025460.1 |
|  | *Klebsiella pneumoniae* strain JX-CR-hvKP-9 plasmid pJX9-5, complete sequence | *Klebsiella pneumoniae* | 8375 | 10336 | 100% | 0 | 100 | 5596 | 5596 | CP064216.1 |
|  | *Klebsiella pneumoniae* *subsp.* pneumoniae strain KP65 plasmid p65_3, complete sequence | *Klebsiella pneumoniae* *subsp.* pneumoniae | 8370 | 10330 | 100% | 0 | 99.98 | 5596 | 5596 | CP101566.1 |
|  | *Klebsiella pneumoniae* strain XHKP502 plasmid pXHKP502-5, complete sequence | *Klebsiella pneumoniae* | 8370 | 10330 | 100% | 0 | 99.98 | 5596 | 5596 | CP066909.1 |
|  | *Klebsiella pneumoniae* strain KP58 plasmid pKP58-5, complete sequence | *Klebsiella pneumoniae* | 8307 | 10336 | 100% | 0 | 100 | 5596 | 5596 | CP041378.1 |
|  | Citrobacter koseri ATCC BAA-895 plasmid pCKO2, complete sequence | Citrobacter koseri ATCC BAA-895 | 8283 | 10190 | 100% | 0 | 99.6 | 5596 | 5601 | CP000824.1 |
|  | *Klebsiella pneumoniae* *subsp.* pneumoniae strain DD02391 plasmid pDD02391-5, complete sequence | *Klebsiella pneumoniae* *subsp.* pneumoniae | 8268 | 10336 | 100% | 0 | 100 | 5596 | 5596 | CP087644.1 |
|  | *Klebsiella pneumoniae* *subsp.* pneumoniae strain WCHKP015093 plasmid p3_015093, complete sequence | *Klebsiella pneumoniae* *subsp.* pneumoniae | 8255 | 10336 | 100% | 0 | 100 | 5596 | 5596 | CP036304.1 |
|  | *Klebsiella pneumoniae* *subsp.* pneumoniae strain SCKP020143 plasmid p4_020143, complete sequence | *Klebsiella pneumoniae* *subsp.* pneumoniae | 8226 | 10336 | 100% | 0 | 100 | 5596 | 5596 | CP028546.2 |
|  | *Klebsiella pneumoniae* strain F726925 plasmid pF726925-4, complete sequence | *Klebsiella pneumoniae* | 8218 | 10336 | 100% | 0 | 100 | 5596 | 5596 | CP081824.1 |
|  | *Klebsiella pneumoniae* strain CDI694 plasmid pCDI694-5.6, complete sequence | *Klebsiella pneumoniae* | 8207 | 10336 | 100% | 0 | 100 | 5596 | 5596 | CP077774.1 |
|  | *Klebsiella pneumoniae* strain WCHKP2080 plasmid p2_095080, complete sequence | *Klebsiella pneumoniae* | 8013 | 10336 | 100% | 0 | 100 | 5596 | 5596 | CP036364.1 |
|  | *Klebsiella pneumoniae* strain JX-CR-hvKP-7 plasmid pJX7-5, complete sequence | *Klebsiella pneumoniae* | 8006 | 10336 | 100% | 0 | 100 | 5596 | 5596 | CP064228.1 |
|  | *Klebsiella pneumoniae* strain KP18-1 plasmid pKP18-1-3, complete sequence | *Klebsiella pneumoniae* | 7997 | 10336 | 100% | 0 | 100 | 5596 | 5596 | CP082000.1 |
|  | *Klebsiella pneumoniae* strain KP18-41 plasmid pKP18-41-3, complete sequence | *Klebsiella pneumoniae* | 7995 | 10336 | 100% | 0 | 100 | 5596 | 5596 | CP082009.1 |
|  | *Klebsiella pneumoniae* strain ARLG-4861 plasmid pC592_5, complete sequence | *Klebsiella pneumoniae* | 7960 | 9032 | 99% | 0 | 99.7 | 5596 | 5584 | CP067621.1 |
|  | *Klebsiella pneumoniae* strain KPA9853 plasmid pA9853_1, complete sequence | *Klebsiella pneumoniae* | 7941 | 10182 | 100% | 0 | 99.54 | 5596 | 5598 | CP070601.1 |
|  | *Klebsiella pneumoniae* strain F44 plasmid p44-4, complete sequence | *Klebsiella pneumoniae* | 7932 | 10336 | 100% | 0 | 100 | 5596 | 5596 | CP025465.1 |
|  | *Klebsiella pneumoniae* strain KP55 plasmid pKP55_6, complete sequence | *Klebsiella pneumoniae* | 7930 | 10336 | 100% | 0 | 100 | 5596 | 5596 | CP055300.1 |
|  | *Klebsiella pneumoniae* strain KP20194c4 plasmid pKP20194c4-p5, complete sequence | *Klebsiella pneumoniae* | 7891 | 10330 | 100% | 0 | 99.98 | 5596 | 5596 | CP054749.1 |
|  | *Klebsiella pneumoniae* strain 135077 plasmid p3_135077, complete sequence | *Klebsiella pneumoniae* | 7878 | 10336 | 100% | 0 | 100 | 5596 | 5596 | CP073295.1 |
|  | *Klebsiella pneumoniae* isolate 392 genome assembly, plasmid: P4 | *Klebsiella pneumoniae* | 7873 | 10299 | 100% | 0 | 99.91 | 5596 | 5598 | OW848890.1 |
|  | *Klebsiella pneumoniae* strain K191663 plasmid unnamed1, complete sequence | *Klebsiella pneumoniae* | 7851 | 10336 | 100% | 0 | 100 | 5596 | 5596 | CP080354.1 |
|  | *Klebsiella pneumoniae* strain WCHKP2 plasmid p2_020002, complete sequence | *Klebsiella pneumoniae* | 7840 | 10336 | 100% | 0 | 100 | 5596 | 5596 | CP028540.3 |
|  | *Klebsiella pneumoniae* isolate 97706988-b809-11e8-aae5-3c4a9275d6c8 genome assembly, chromosome: 1 | *Klebsiella pneumoniae* | 7799 | 11066 | 100% | 0 | 100 | 5596 | 5652578 | LR596813.1 |
|  | *Klebsiella pneumoniae* strain BJCFK909 plasmid p4s2, complete sequence | *Klebsiella pneumoniae* | 7782 | 10336 | 100% | 0 | 100 | 5596 | 5596 | CP034127.1 |
|  | *Klebsiella pneumoniae* strain KP-C76 plasmid unnamed1, complete sequence | *Klebsiella pneumoniae* | 7753 | 10336 | 100% | 0 | 100 | 5596 | 5596 | CP080302.1 |
|  | *Klebsiella pneumoniae* strain CR-HvKP4 plasmid pCR-HvKP4-p5, complete sequence | *Klebsiella pneumoniae* | 7666 | 10336 | 100% | 0 | 100 | 5596 | 5596 | CP040544.1 |
|  | *Klebsiella pneumoniae* isolate 392 genome assembly, plasmid: P4 | *Klebsiella pneumoniae* | 7646 | 10293 | 100% | 0 | 99.88 | 5596 | 5599 | OW849056.1 |
|  | *Klebsiella pneumoniae* strain L39_2 plasmid p6_L39, complete sequence | *Klebsiella pneumoniae* | 7607 | 10336 | 100% | 0 | 100 | 5596 | 5596 | CP033959.1 |
|  | *Klebsiella pneumoniae* strain 160111 plasmid p5.6K_L111, complete sequence | *Klebsiella pneumoniae* | 7600 | 10336 | 100% | 0 | 100 | 5596 | 5596 | CP030131.1 |
|  | *Klebsiella pneumoniae* strain KP55 plasmid pKPC-5505, complete sequence | *Klebsiella pneumoniae* | 7496 | 10336 | 100% | 0 | 100 | 5596 | 5596 | OL891655.1 |
|  | *Klebsiella pneumoniae* strain KP18-2172 plasmid pKP18-2172-3, complete sequence | *Klebsiella pneumoniae* | 7491 | 10336 | 100% | 0 | 100 | 5596 | 5596 | CP082036.1 |
|  | *Klebsiella pneumoniae* strain ZRKP04 plasmid unnamed5, complete sequence | *Klebsiella pneumoniae* | 7478 | 10336 | 100% | 0 | 100 | 5596 | 5596 | CP050341.1 |
|  | *Klebsiella pneumoniae* strain 37 plasmid pKP37_6, complete sequence | *Klebsiella pneumoniae* | 7459 | 10336 | 100% | 0 | 100 | 5596 | 5596 | CP082758.1 |
|  | *Klebsiella pneumoniae* *subsp.* pneumoniae strain KP21 plasmid p21_3, complete sequence | *Klebsiella pneumoniae* *subsp.* pneumoniae | 7452 | 10336 | 100% | 0 | 100 | 5596 | 5596 | CP101545.1 |
|  | *Klebsiella pneumoniae* strain KPN142 plasmid pn142_3, complete sequence | *Klebsiella pneumoniae* | 7341 | 10336 | 100% | 0 | 100 | 5596 | 5596 | CP053878.1 |
|  | *Klebsiella pneumoniae* strain KP-426 plasmid unnamed2, complete sequence | *Klebsiella pneumoniae* | 7336 | 10336 | 100% | 0 | 100 | 5596 | 5596 | CP080315.1 |
|  | *Klebsiella pneumoniae* strain 49088 plasmid p49088-6.796, complete sequence | *Klebsiella pneumoniae* | 7302 | 10344 | 100% | 0 | 99.97 | 5596 | 6796 | CP088998.1 |
|  | *Klebsiella pneumoniae* strain WSCRKP plasmid pWSCRKP-5, complete sequence | *Klebsiella pneumoniae* | 7276 | 10336 | 100% | 0 | 100 | 5596 | 5596 | CP091073.1 |
|  | *Klebsiella pneumoniae* isolate 99060032-b809-11e8-aae5-3c4a9275d6c8 genome assembly, chromosome: 1 | *Klebsiella pneumoniae* | 7232 | 10334 | 99% | 0 | 100 | 5596 | 5479501 | LR596812.1 |
|  | *Klebsiella pneumoniae* strain WCHKP115068 plasmid p3_115068, complete sequence | *Klebsiella pneumoniae* | 7208 | 10336 | 100% | 0 | 100 | 5596 | 5596 | CP036370.1 |
|  | *Klebsiella pneumoniae* strain KP19-2196 plasmid pKP19-2196-4, complete sequence | *Klebsiella pneumoniae* | 7167 | 10336 | 100% | 0 | 100 | 5596 | 5596 | CP082040.1 |
|  | *Klebsiella pneumoniae* *subsp.* pneumoniae strain DD01635 plasmid pDD01635-5, complete sequence | *Klebsiella pneumoniae* *subsp.* pneumoniae | 7081 | 10336 | 100% | 0 | 100 | 5596 | 5596 | CP087662.1 |
|  | *Klebsiella pneumoniae* strain WCHKP090050 plasmid p2_090050, complete sequence | *Klebsiella pneumoniae* | 7003 | 10336 | 100% | 0 | 100 | 5596 | 5596 | CP043372.1 |
|  | *Klebsiella pneumoniae* strain KPC-2 plasmid pKP169-P6, complete sequence | *Klebsiella pneumoniae* | 6944 | 10330 | 100% | 0 | 100 | 5596 | 5596 | CP078128.1 |
|  | *Klebsiella pneumoniae* strain FZKP4523 plasmid p2_FZKP4523, complete sequence | *Klebsiella pneumoniae* | 6938 | 10328 | 100% | 0 | 100 | 5596 | 5595 | CP101536.1 |
|  | Pseudomonas aeruginosa strain ZPPH1 plasmid p5, complete sequence | Pseudomonas aeruginosa | 5225 | 5438 | 100% | 0 | 99.89 | 5596 | 2953 | CP077993.1 |
| pCR-hvKP132-P4 | Pseudomonas aeruginosa strain ZPPH29 plasmid p3, complete sequence | Pseudomonas aeruginosa | 5147 | 5449 | 100% | 0 | 99.96 | 2953 | 2953 | CP077980.1 |
|  | Serratia marcescens strain 3024 plasmid pXQ, complete sequence | Serratia marcescens | 5003 | 5455 | 100% | 0 | 100 | 2953 | 2953 | CP047684.1 |
|  | Pseudomonas aeruginosa isolate P33 plasmid pP33-4, complete sequence | Pseudomonas aeruginosa | 4994 | 5438 | 100% | 0 | 99.89 | 2953 | 2953 | CP065416.1 |
|  | Serratia marcescens strain 2838 plasmid pXQ, complete sequence | Serratia marcescens | 4285 | 5455 | 100% | 0 | 100 | 2953 | 2953 | CP047687.1 |
|  | Serratia marcescens strain C110 plasmid pXQ, complete sequence | Serratia marcescens | 4194 | 5455 | 100% | 0 | 100 | 2953 | 2953 | CP047693.1 |
|  | Serratia marcescens strain 1140- plasmid pXQ, complete sequence | Serratia marcescens | 3742 | 5455 | 100% | 0 | 100 | 2953 | 2953 | CP047690.1 |
|  | Pseudomonas aeruginosa strain ZPPH14 plasmid p3, complete sequence | Pseudomonas aeruginosa | 3742 | 5455 | 100% | 0 | 100 | 2953 | 2953 | CP077983.1 |
|  | Serratia marcescens strain 4201 plasmid pXQ, complete sequence | Serratia marcescens | 3733 | 5455 | 100% | 0 | 100 | 2953 | 2953 | CP047681.1 |
|  | Salmonella enterica strain 628 plasmid p628-3, complete sequence | Salmonella enterica | 3452 | 5449 | 100% | 0 | 99.95 | 2953 | 2953 | CP091552.1 |
|  | Pseudomonas aeruginosa strain SRRSH1120 plasmid p2, complete sequence | Pseudomonas aeruginosa | 3393 | 5455 | 100% | 0 | 100 | 2953 | 2953 | CP078001.1 |
|  | Pseudomonas aeruginosa strain ZPPH33 plasmid p5, complete sequence | Pseudomonas aeruginosa | 3225 | 5438 | 100% | 0 | 99.94 | 2953 | 2953 | CP077976.1 |
|  | Salmonella enterica *subsp.* enterica serovar Dessau strain KUFSE-SAL0043 chromosome | Salmonella enterica *subsp.* enterica serovar Dessau | 2881 | 5451 | 100% | 0 | 100 | 2953 | 5099177 | CP043765.1 |
|  | Pseudomonas aeruginosa strain FAHZU31 plasmid p3, complete sequence | Pseudomonas aeruginosa | 2796 | 5449 | 100% | 0 | 99.93 | 2953 | 2953 | CP078011.1 |
|  | Salmonella enterica *subsp.* enterica serovar Montevideo strain R17.4849 plasmid pR17.4849_3.0k, complete sequence | Salmonella enterica *subsp.* enterica serovar Montevideo | 1792 | 4326 | 93% | 0 | 98.71 | 2953 | 2965 | CP100750.1 |
|  | *Escherichia coli* strain RH-045-MS chromosome | *Escherichia coli* | 1668 | 1668 | 36% | 0 | 95.02 | 2953 | 6042336 | CP050203.1 |
|  | Synthetic construct clone pEH256, complete sequence | synthetic construct | 601 | 763 | 36% | 2.00E-166 | 78.92 | 2953 | 7509 | MT024803.1 |
|  | Synthetic construct clone pEH161, complete sequence | synthetic construct | 601 | 763 | 36% | 2.00E-166 | 78.92 | 2953 | 5610 | MT024802.1 |
|  | Synthetic construct clone pEH159, complete sequence | synthetic construct | 601 | 763 | 36% | 2.00E-166 | 78.92 | 2953 | 5223 | MT024801.1 |
|  | Synthetic construct clone pEH158, complete sequence | synthetic construct | 601 | 763 | 36% | 2.00E-166 | 78.92 | 2953 | 5266 | MT024800.1 |
|  | Synthetic construct clone pEH157, complete sequence | synthetic construct | 601 | 763 | 36% | 2.00E-166 | 78.92 | 2953 | 5528 | MT024799.1 |
|  | Synthetic construct clone pEH155, complete sequence | synthetic construct | 601 | 763 | 36% | 2.00E-166 | 78.92 | 2953 | 5580 | MT024798.1 |
|  | Synthetic construct clone pEH154, complete sequence | synthetic construct | 601 | 763 | 36% | 2.00E-166 | 78.92 | 2953 | 5741 | MT024797.1 |
|  | Synthetic construct clone pEH148, complete sequence | synthetic construct | 601 | 763 | 36% | 2.00E-166 | 78.92 | 2953 | 5638 | MT024796.1 |
|  | Synthetic construct clone pEH147, complete sequence | synthetic construct | 601 | 763 | 36% | 2.00E-166 | 78.92 | 2953 | 6351 | MT024795.1 |
|  | Synthetic construct clone pEH137, complete sequence | synthetic construct | 601 | 763 | 36% | 2.00E-166 | 78.92 | 2953 | 5218 | MT024794.1 |
|  | Synthetic construct clone pEH136, complete sequence | synthetic construct | 601 | 763 | 36% | 2.00E-166 | 78.92 | 2953 | 5203 | MT024793.1 |
|  | Synthetic construct clone pEH134, complete sequence | synthetic construct | 601 | 763 | 36% | 2.00E-166 | 78.92 | 2953 | 5807 | MT024792.1 |
|  | Synthetic construct clone pEH083, complete sequence | synthetic construct | 601 | 763 | 36% | 2.00E-166 | 78.92 | 2953 | 5610 | MT024791.1 |
|  | Synthetic construct clone pEH052, complete sequence | synthetic construct | 601 | 763 | 36% | 2.00E-166 | 78.92 | 2953 | 7046 | MT024790.1 |
|  | Synthetic construct clone pEH042, complete sequence | synthetic construct | 601 | 763 | 36% | 2.00E-166 | 78.92 | 2953 | 5579 | MT024789.1 |
|  | Cloning vector pBBR5pemIK-pBAD, complete sequence | Cloning vector pBBR5pemIK-pBAD | 601 | 763 | 36% | 2.00E-166 | 78.92 | 2953 | 8590 | MN044104.1 |
|  | Cloning vector pBBR5pemIK-pKan, complete sequence | Cloning vector pBBR5pemIK-pKan | 601 | 763 | 36% | 2.00E-166 | 78.92 | 2953 | 7309 | MN044103.1 |
|  | Cloning vector pBBR4pemIK-GW, complete sequence | Cloning vector pBBR4pemIK-GW | 601 | 763 | 36% | 2.00E-166 | 78.92 | 2953 | 7368 | MN044102.1 |
|  | Cloning vector pBBR4pemIK, complete sequence | Cloning vector pBBR4pemIK | 601 | 763 | 36% | 2.00E-166 | 78.92 | 2953 | 5655 | MN044101.1 |
|  | Cloning vector pMBLcas9-mcr-1, complete sequence | Cloning vector pMBLcas9-mcr-1 | 601 | 763 | 35% | 2.00E-166 | 79.29 | 2953 | 11496 | MK637406.1 |
|  | Cloning vector pMBLcas9, complete sequence | Cloning vector pMBLcas9 | 601 | 763 | 35% | 2.00E-166 | 79.29 | 2953 | 10955 | MK637405.1 |
|  | Cloning vector yTREX, complete sequence | Cloning vector yTREX | 601 | 763 | 35% | 2.00E-166 | 79.29 | 2953 | 10548 | MK416190.1 |
|  | Cloning vector pPS-BR, complete sequence | Cloning vector pPS-BR | 601 | 763 | 36% | 2.00E-166 | 78.92 | 2953 | 6929 | MH539767.1 |
|  | Cloning vector pBBR1MCS-Erm, complete sequence | Cloning vector pBBR1MCS-Erm | 601 | 763 | 36% | 2.00E-166 | 78.92 | 2953 | 4811 | KY368389.1 |
|  | Cloning vector pBBR1-aceEF-lpd, complete sequence | Cloning vector pBBR1-aceEF-lpd | 601 | 763 | 36% | 2.00E-166 | 78.92 | 2953 | 10763 | MH651726.1 |
|  | Cloning vector pBBR1-acs, complete sequence | Cloning vector pBBR1-acs | 601 | 763 | 36% | 2.00E-166 | 78.92 | 2953 | 6628 | MH651725.1 |
|  | Cloning vector pBBR1-pgk, complete sequence | Cloning vector pBBR1-pgk | 601 | 763 | 36% | 2.00E-166 | 78.92 | 2953 | 5833 | MH651724.1 |
|  | Expression vector pYB8895, complete sequence | Expression vector pYB8895 | 601 | 763 | 35% | 2.00E-166 | 79.25 | 2953 | 4523 | OL333432.1 |
|  | Cloning vector pMiniTn5-Gm, complete sequence | Cloning vector pMiniTn5-Gm | 601 | 763 | 35% | 2.00E-166 | 79.29 | 2953 | 4484 | OL449775.1 |
|  | Cloning vector pMiniTn5-Km, complete sequence | Cloning vector pMiniTn5-Km | 601 | 763 | 35% | 2.00E-166 | 79.29 | 2953 | 4916 | OL449774.1 |
|  | Cloning vector pETS220-BIATlux, complete sequence | Cloning vector pETS220-BIATlux | 601 | 763 | 36% | 2.00E-166 | 78.92 | 2953 | 10677 | MW117147.1 |
|  | Cloning vector pCG403, complete sequence | Cloning vector pCG403 | 484 | 646 | 26% | 3.00E-131 | 81.37 | 2953 | 5982 | MN744967.1 |
|  | Cloning vector pCG402, complete sequence | Cloning vector pCG402 | 484 | 646 | 26% | 3.00E-131 | 81.37 | 2953 | 5269 | MN744966.1 |
|  | Cloning vector pCG401, complete sequence | Cloning vector pCG401 | 484 | 646 | 26% | 3.00E-131 | 81.37 | 2953 | 7974 | MN744965.1 |
|  | Cloning vector pCG303, complete sequence | Cloning vector pCG303 | 484 | 646 | 26% | 3.00E-131 | 81.37 | 2953 | 5970 | MN744964.1 |
|  | Cloning vector pCG302, complete sequence | Cloning vector pCG302 | 484 | 646 | 26% | 3.00E-131 | 81.37 | 2953 | 5281 | MN744963.1 |
|  | Cloning vector pCG301, complete sequence | Cloning vector pCG301 | 484 | 646 | 26% | 3.00E-131 | 81.37 | 2953 | 7974 | MN744962.1 |
|  | Cloning vector pCG103, complete sequence | Cloning vector pCG103 | 484 | 646 | 26% | 3.00E-131 | 81.37 | 2953 | 4931 | MN744961.1 |
|  | Cloning vector pCG102, complete sequence | Cloning vector pCG102 | 484 | 646 | 26% | 3.00E-131 | 81.37 | 2953 | 4937 | MN744960.1 |
|  | Cloning vector pCG101, complete sequence | Cloning vector pCG101 | 484 | 646 | 26% | 3.00E-131 | 81.37 | 2953 | 4919 | MN744959.1 |
|  | Promoter probe vector pNG1, complete sequence | Promoter probe vector pNG1 | 484 | 646 | 26% | 3.00E-131 | 81.37 | 2953 | 8446 | MK836322.1 |
|  | Cloning Vector pCG18.HlyC, complete sequence | Cloning Vector pCG18.HlyC | 484 | 646 | 25% | 3.00E-131 | 81.44 | 2953 | 8122 | MG702581.1 |
|  | Cloning Vector pCG18.HlyA, complete sequence | Cloning Vector pCG18.HlyA | 484 | 646 | 25% | 3.00E-131 | 81.44 | 2953 | 7803 | MG702580.1 |
|  | Cloning Vector pCG18.HF, complete sequence | Cloning Vector pCG18.HF | 484 | 646 | 25% | 3.00E-131 | 81.44 | 2953 | 7639 | MG702579.1 |
|  | Cloning Vector pCG18.Hem, complete sequence | Cloning Vector pCG18.Hem | 484 | 646 | 25% | 3.00E-131 | 81.44 | 2953 | 8078 | MG702578.1 |
|  | Cloning Vector pCG18.glr1, complete sequence | Cloning Vector pCG18.glr1 | 484 | 646 | 25% | 3.00E-131 | 81.44 | 2953 | 7568 | MG702577.1 |
|  | Cloning Vector pCG18, complete sequence | Cloning Vector pCG18 | 484 | 646 | 25% | 3.00E-131 | 81.44 | 2953 | 7543 | MG702576.1 |
|  | Cloning Vector pCG6, complete sequence | Cloning Vector pCG6 | 484 | 646 | 25% | 3.00E-131 | 81.44 | 2953 | 7318 | MH734181.1 |
|  | Vector pHC_pUC_ori, complete sequence | Vector pHC_pUC_ori | 484 | 646 | 25% | 3.00E-131 | 81.44 | 2953 | 4807 | MH238457.1 |
|  | Vector pLC_pBBR1_ori, complete sequence | Vector pLC_pBBR1_ori | 484 | 646 | 25% | 3.00E-131 | 81.44 | 2953 | 5147 | MH238456.1 |
|  | Cloning Vector pHyp4s.His, complete sequence | Cloning Vector pHyp4s.His | 484 | 646 | 25% | 3.00E-131 | 81.44 | 2953 | 6328 | MZ215990.1 |
|  | Cloning Vector pHyp4s.MBP, complete sequence | Cloning Vector pHyp4s.MBP | 484 | 646 | 25% | 3.00E-131 | 81.44 | 2953 | 7408 | MZ215989.1 |
|  | Cloning Vector pHyp4s.GST, complete sequence | Cloning Vector pHyp4s.GST | 484 | 646 | 25% | 3.00E-131 | 81.44 | 2953 | 6961 | MZ215988.1 |
|  | Cloning Vector pHyp4s.TrxA, complete sequence | Cloning Vector pHyp4s.TrxA | 484 | 646 | 25% | 3.00E-131 | 81.44 | 2953 | 6634 | MZ215987.1 |
|  | Cloning Vector pHyp4s.Myc, complete sequence | Cloning Vector pHyp4s.Myc | 484 | 646 | 25% | 3.00E-131 | 81.44 | 2953 | 6311 | MZ215986.1 |
|  | Expression vector p2BBAD-phaP1-uspA-rpoN, complete sequence | Expression vector p2BBAD-phaP1-uspA-rpoN | 484 | 646 | 26% | 3.00E-131 | 81.37 | 2953 | 7828 | OL331259.1 |
|  | Expression vector p2BBAD-rpoN, complete sequence | Expression vector p2BBAD-rpoN | 484 | 646 | 26% | 3.00E-131 | 81.37 | 2953 | 6774 | OL331258.1 |
|  | Expression vector p2BBAD-uspA, complete sequence | Expression vector p2BBAD-uspA | 484 | 646 | 26% | 3.00E-131 | 81.37 | 2953 | 6858 | OL331257.1 |
|  | Expression vector p2BBAD-phaP2, complete sequence | Expression vector p2BBAD-phaP2 | 484 | 646 | 26% | 3.00E-131 | 81.37 | 2953 | 6990 | OL331256.1 |
|  | Expression vector p2BBAD-phaP1, complete sequence | Expression vector p2BBAD-phaP1 | 484 | 646 | 26% | 3.00E-131 | 81.37 | 2953 | 7002 | OL331255.1 |
|  | Expression vector p2BBAD-phaB2-phaC2, complete sequence | Expression vector p2BBAD-phaB2-phaC2 | 484 | 646 | 26% | 3.00E-131 | 81.37 | 2953 | 8946 | OL331254.1 |
|  | Expression vector p2BBAD, complete sequence | Expression vector p2BBAD | 484 | 646 | 26% | 3.00E-131 | 81.37 | 2953 | 6417 | OL331253.1 |
|  | Cloning vector pHyp4s kanamycin resistance protein gene, complete cds | Cloning vector pHyp4s | 484 | 646 | 25% | 3.00E-131 | 81.44 | 2953 | 7597 | MW132123.1 |
|  | Cloning vector pTonBs kanamycin resistance protein gene, complete cds | Cloning vector pTonBs | 484 | 646 | 25% | 3.00E-131 | 81.44 | 2953 | 7597 | MW132122.1 |
|  | Cloning vector pHyp1s kanamycin resistance protein gene, complete cds | Cloning vector pHyp1s | 484 | 646 | 25% | 3.00E-131 | 81.44 | 2953 | 7591 | MW132121.1 |
|  | Cloning vector pCopB kanamycin resistance protein gene, complete cds | Cloning vector pCopB | 484 | 646 | 25% | 3.00E-131 | 81.44 | 2953 | 7318 | MW132120.1 |
|  | Cloning vector pABCTrans kanamycin resistance protein gene, complete cds | Cloning vector pABCTrans | 484 | 646 | 25% | 3.00E-131 | 81.44 | 2953 | 7318 | MW132119.1 |
|  | Cloning vector pMXKDX kanamycin resistance protein gene, complete cds | Cloning vector pMXKDX | 484 | 646 | 25% | 3.00E-131 | 81.44 | 2953 | 7318 | MW132118.1 |
|  | Cloning vector pHyp9 kanamycin resistance protein gene, complete cds | Cloning vector pHyp9 | 484 | 646 | 25% | 3.00E-131 | 81.44 | 2953 | 7318 | MW132117.1 |
|  | Cloning vector pPerox kanamycin resistance protein gene, complete cds | Cloning vector pPerox | 484 | 646 | 25% | 3.00E-131 | 81.44 | 2953 | 7318 | MW132116.1 |
|  | Cloning vector pHyp8 kanamycin resistance protein gene, complete cds | Cloning vector pHyp8 | 484 | 646 | 25% | 3.00E-131 | 81.44 | 2953 | 7318 | MW132115.1 |
|  | Cloning vector pTonB3 kanamycin resistance protein gene, complete cds | Cloning vector pTonB3 | 484 | 646 | 25% | 3.00E-131 | 81.44 | 2953 | 7318 | MW132114.1 |
|  | Cloning vector pHyp6 kanamycin resistance protein gene, complete cds | Cloning vector pHyp6 | 484 | 646 | 25% | 3.00E-131 | 81.44 | 2953 | 7318 | MW132113.1 |
|  | Cloning vector pALys kanamycin resistance protein gene, complete cds | Cloning vector pALys | 484 | 646 | 25% | 3.00E-131 | 81.44 | 2953 | 7318 | MW132112.1 |
|  | Cloning vector pHyp5 kanamycin resistance protein gene, complete cds | Cloning vector pHyp5 | 484 | 646 | 25% | 3.00E-131 | 81.44 | 2953 | 7318 | MW132111.1 |
|  | Cloning vector pHyp4 kanamycin resistance protein gene, complete cds | Cloning vector pHyp4 | 484 | 646 | 25% | 3.00E-131 | 81.44 | 2953 | 7318 | MW132110.1 |
|  | Cloning vector pPIsom kanamycin resistance protein gene, complete cds | Cloning vector pPIsom | 484 | 646 | 25% | 3.00E-131 | 81.44 | 2953 | 7318 | MW132109.1 |
|  | Cloning vector pAChan kanamycin resistance protein gene, complete cds | Cloning vector pAChan | 484 | 646 | 25% | 3.00E-131 | 81.44 | 2953 | 7318 | MW132108.1 |
|  | Cloning vector pHyp3 kanamycin resistance protein gene, complete cds | Cloning vector pHyp3 | 484 | 646 | 25% | 3.00E-131 | 81.44 | 2953 | 7318 | MW132107.1 |
|  | Cloning vector pTonB2 kanamycin resistance protein gene, complete cds | Cloning vector pTonB2 | 484 | 646 | 25% | 3.00E-131 | 81.44 | 2953 | 7318 | MW132106.1 |
|  | Cloning vector pHyp2 kanamycin resistance protein gene, complete cds | Cloning vector pHyp2 | 484 | 646 | 25% | 3.00E-131 | 81.44 | 2953 | 7315 | MW132105.1 |
|  | Cloning vector pHyp1 kanamycin resistance protein gene, complete cds | Cloning vector pHyp1 | 484 | 646 | 25% | 3.00E-131 | 81.44 | 2953 | 7312 | MW132104.1 |
|  | Cloning vector pGGTP kanamycin resistance protein gene, complete cds | Cloning vector pGGTP | 484 | 646 | 25% | 3.00E-131 | 81.44 | 2953 | 7294 | MW132103.1 |
|  | Cloning vector pDCP kanamycin resistance protein gene, complete cds | Cloning vector pDCP | 484 | 646 | 25% | 3.00E-131 | 81.44 | 2953 | 7318 | MW132102.1 |
| pCR-hvKP173-Vir-P1 | *Klebsiella pneumoniae* *subsp.* pneumoniae strain SH2 plasmid pSH2-KPC, complete sequence | *Klebsiella pneumoniae* *subsp.* pneumoniae | 1.98E+05 | 4.27E+05 | 100% | 0 | 99.78 | 178873 | 302845 | MH643791.1 |
|  | *Klebsiella pneumoniae* strain L388 plasmid p1-L388 | *Klebsiella pneumoniae* | 1.65E+05 | 4.05E+05 | 100% | 0 | 99.98 | 178873 | 217870 | CP029221.1 |
|  | *Klebsiella pneumoniae* *subsp.* pneumoniae strain SCKP020079 plasmid pVir_020079, complete sequence | *Klebsiella pneumoniae* *subsp.* pneumoniae | 1.65E+05 | 4.29E+05 | 100% | 0 | 99.98 | 178873 | 178741 | CP029383.2 |
|  | *Klebsiella pneumoniae* strain CRKP78R plasmid p2, complete sequence | *Klebsiella pneumoniae* | 1.65E+05 | 4.05E+05 | 100% | 0 | 99.97 | 178873 | 215966 | CP066255.1 |
|  | *Klebsiella pneumoniae* *subsp.* pneumoniae strain HA2 plasmid pHA2-23-vir, complete sequence | *Klebsiella pneumoniae* *subsp.* pneumoniae | 1.63E+05 | 4.13E+05 | 100% | 0 | 99.67 | 178873 | 217926 | MH643788.1 |
|  | *Klebsiella pneumoniae* *subsp.* pneumoniae strain DD01845 plasmid pDD01845-1, complete sequence | *Klebsiella pneumoniae* *subsp.* pneumoniae | 1.30E+05 | 4.09E+05 | 100% | 0 | 99.97 | 178873 | 219069 | CP087664.1 |
|  | *Klebsiella pneumoniae* *subsp.* pneumoniae strain DD01635 plasmid pDD01635-1, complete sequence | *Klebsiella pneumoniae* *subsp.* pneumoniae | 1.30E+05 | 4.09E+05 | 100% | 0 | 99.97 | 178873 | 219068 | CP087658.1 |
|  | *Klebsiella pneumoniae* strain 33367 plasmid p33367_VIR, complete sequence | *Klebsiella pneumoniae* | 1.30E+05 | 4.03E+05 | 100% | 0 | 99.96 | 178873 | 215836 | CP099414.1 |
|  | *Klebsiella pneumoniae* strain C2582 plasmid pVir_C2582 | *Klebsiella pneumoniae* | 1.30E+05 | 4.31E+05 | 100% | 0 | 99.96 | 178873 | 216779 | CP079209.1 |
|  | *Klebsiella pneumoniae* strain C789 plasmid pVir-CR-hvKP-C789, complete sequence | *Klebsiella pneumoniae* | 1.30E+05 | 4.05E+05 | 100% | 0 | 99.97 | 178873 | 215950 | CP034416.1 |
|  | *Klebsiella pneumoniae* strain JX-CR-hvKP-4 plasmid pJX4-1, complete sequence | *Klebsiella pneumoniae* | 1.30E+05 | 4.29E+05 | 100% | 0 | 99.94 | 178873 | 219405 | CP064236.1 |
|  | *Klebsiella pneumoniae* strain CDI694 plasmid pCDI694-216.6, complete sequence | *Klebsiella pneumoniae* | 1.30E+05 | 4.30E+05 | 100% | 0 | 99.95 | 178873 | 216645 | CP077778.1 |
|  | *Klebsiella pneumoniae* strain 9949 plasmid unnamed1, complete sequence | *Klebsiella pneumoniae* | 1.30E+05 | 4.65E+05 | 100% | 0 | 99.9 | 178873 | 237416 | CP050281.1 |
|  | *Klebsiella pneumoniae* strain QL24 plasmid pKPN-QL24, complete sequence | *Klebsiella pneumoniae* | 1.29E+05 | 3.96E+05 | 100% | 0 | 99.77 | 178873 | 215940 | MH263654.1 |
|  | *Klebsiella pneumoniae* strain XJ-K1 plasmid unnamed1, complete sequence | *Klebsiella pneumoniae* | 1.28E+05 | 4.13E+05 | 94% | 0 | 99.99 | 178873 | 207409 | CP032164.1 |
|  | *Klebsiella pneumoniae* strain KP2648 plasmid pKP2648-Vir, complete sequence | *Klebsiella pneumoniae* | 1.28E+05 | 4.30E+05 | 100% | 0 | 99.99 | 178873 | 216902 | CP072558.1 |
|  | *Klebsiella pneumoniae* strain Kp36 plasmid unnamed1, complete sequence | *Klebsiella pneumoniae* | 1.28E+05 | 4.34E+05 | 100% | 0 | 99.99 | 178873 | 219800 | CP047193.1 |
|  | *Escherichia coli* strain EC2648-Vir1 plasmid pVir-fusion, complete sequence | *Escherichia coli* | 1.28E+05 | 4.38E+05 | 100% | 0 | 99.99 | 178873 | 255611 | CP083611.1 |
|  | *Klebsiella pneumoniae* strain XJ-K2 plasmid unnamed1, complete sequence | *Klebsiella pneumoniae* | 1.28E+05 | 4.35E+05 | 100% | 0 | 99.99 | 178873 | 219775 | CP032241.1 |
|  | *Klebsiella pneumoniae* strain JNKPN26 plasmid pJNKPN26_Vir, complete sequence | *Klebsiella pneumoniae* | 1.27E+05 | 4.39E+05 | 100% | 0 | 99.92 | 178873 | 220975 | CP090206.1 |
|  | *Klebsiella pneumoniae* strain JX-CR-hvKP-7 plasmid pJX7-1, complete sequence | *Klebsiella pneumoniae* | 1.26E+05 | 4.33E+05 | 100% | 0 | 99.95 | 178873 | 219796 | CP064224.1 |
|  | *Klebsiella pneumoniae* strain KP18-2079 plasmid pKP18-2079_vir, complete sequence | *Klebsiella pneumoniae* | 1.24E+05 | 2.67E+05 | 67% | 0 | 99.91 | 178873 | 182326 | MT090958.1 |
|  | *Klebsiella pneumoniae* strain FK 6768 plasmid unnamed1, complete sequence | *Klebsiella pneumoniae* | 1.24E+05 | 4.32E+05 | 99% | 0 | 99.99 | 178873 | 219459 | CP065555.1 |
|  | *Klebsiella pneumoniae* strain JNKPN26 plasmid pJNKPN26_Vir | *Klebsiella pneumoniae* | 1.24E+05 | 4.39E+05 | 100% | 0 | 99.99 | 178873 | 220975 | MZ546617.1 |
|  | *Klebsiella pneumoniae* strain XHKP502 plasmid pXHKP502-1, complete sequence | *Klebsiella pneumoniae* | 1.14E+05 | 4.47E+05 | 100% | 0 | 99.96 | 178873 | 221472 | CP066905.1 |
|  | *Escherichia coli* strain EC2648-Vir2 plasmid pKP2648-VirV, complete sequence | *Escherichia coli* | 1.10E+05 | 4.31E+05 | 99% | 0 | 99.97 | 178873 | 222599 | CP083992.1 |
|  | *Klebsiella pneumoniae* strain F16KP0082 plasmid pF16KP0082-1, complete sequence | *Klebsiella pneumoniae* | 1.07E+05 | 3.98E+05 | 98% | 0 | 99.95 | 178873 | 232142 | CP052163.1 |
|  | *Klebsiella pneumoniae* strain BSI030 plasmid pBSI030_vf | *Klebsiella pneumoniae* | 1.07E+05 | 3.99E+05 | 96% | 0 | 99.92 | 178873 | 227912 | MT269852.1 |
|  | *Klebsiella pneumoniae* *subsp.* pneumoniae strain SH9 plasmid pSH9-VIR, complete sequence | *Klebsiella pneumoniae* *subsp.* pneumoniae | 1.07E+05 | 3.14E+05 | 77% | 0 | 99.87 | 178873 | 188437 | MH255828.1 |
|  | *Klebsiella pneumoniae* strain BSI074 plasmid pBSI074_vf | *Klebsiella pneumoniae* | 1.05E+05 | 4.14E+05 | 100% | 0 | 100 | 178873 | 218366 | MT269847.1 |
|  | *Klebsiella pneumoniae* strain FRPDR plasmid pFRPDR_1, complete sequence | *Klebsiella pneumoniae* | 1.05E+05 | 4.33E+05 | 100% | 0 | 99.99 | 178873 | 219933 | CP063760.1 |
|  | *Klebsiella pneumoniae* strain 8695 plasmid pFK8695-rmpA, complete sequence | *Klebsiella pneumoniae* | 1.05E+05 | 3.38E+05 | 76% | 0 | 99.99 | 178873 | 150052 | CP085891.1 |
|  | *Klebsiella pneumoniae* strain CRKP66R plasmid pCRKP66R-2, complete sequence | *Klebsiella pneumoniae* | 1.05E+05 | 4.04E+05 | 100% | 0 | 99.99 | 178873 | 215999 | CP063834.1 |
|  | *Klebsiella pneumoniae* strain 50700 plasmid p50700-217.1, complete sequence | *Klebsiella pneumoniae* | 1.05E+05 | 4.06E+05 | 100% | 0 | 99.98 | 178873 | 217070 | CP088996.1 |
|  | *Klebsiella pneumoniae* strain KP1878 plasmid p1878-217k, complete sequence | *Klebsiella pneumoniae* | 99906 | 4.09E+05 | 97% | 0 | 99.94 | 178873 | 217255 | CP072997.1 |
|  | *Klebsiella pneumoniae* strain WSCRKP plasmid pWSCRKP-1, complete sequence | *Klebsiella pneumoniae* | 99773 | 4.41E+05 | 100% | 0 | 100 | 178873 | 221776 | CP091069.1 |
|  | *Klebsiella pneumoniae* strain CRKP52R plasmid p2, complete sequence | *Klebsiella pneumoniae* | 98207 | 3.60E+05 | 84% | 0 | 99.95 | 178873 | 193221 | CP066250.1 |
|  | *Klebsiella pneumoniae* strain NMI9546/19 plasmid p9546/19_1, complete sequence | *Klebsiella pneumoniae* | 84874 | 4.24E+05 | 98% | 0 | 99.98 | 178873 | 235674 | ON081625.1 |
|  | *Klebsiella pneumoniae* strain 49088 plasmid p49088-279.2, complete sequence | *Klebsiella pneumoniae* | 72657 | 2.62E+05 | 66% | 0 | 99.89 | 178873 | 279210 | CP089000.1 |
|  | *Klebsiella pneumoniae* strain JX-CR-hvKP-12 plasmid pJX12-1, complete sequence | *Klebsiella pneumoniae* | 72469 | 4.02E+05 | 96% | 0 | 99.98 | 178873 | 230835 | CP064206.1 |
|  | *Klebsiella pneumoniae* strain TH12908 chromosome, complete genome | *Klebsiella pneumoniae* | 72448 | 4.15E+05 | 96% | 0 | 99.97 | 178873 | 5692842 | CP087123.1 |
|  | *Klebsiella pneumoniae* strain 11492 plasmid unnamed, complete sequence | *Klebsiella pneumoniae* | 72445 | 3.36E+05 | 77% | 0 | 99.97 | 178873 | 193176 | MF993442.1 |
|  | *Klebsiella pneumoniae* strain MAR14-456 plasmid pMAR14456-IncHI1B, complete sequence | *Klebsiella pneumoniae* | 72445 | 3.89E+05 | 98% | 0 | 99.97 | 178873 | 232362 | CP063278.1 |
|  | *Klebsiella pneumoniae* strain SA12 plasmid pKp_SA12_1, complete sequence | *Klebsiella pneumoniae* | 72423 | 3.77E+05 | 96% | 0 | 99.96 | 178873 | 230142 | CP084867.1 |
|  | *Klebsiella pneumoniae* strain AP8555 plasmid pAP855, complete sequence | *Klebsiella pneumoniae* | 72400 | 3.83E+05 | 94% | 0 | 99.95 | 178873 | 357837 | CP035384.1 |
|  | *Klebsiella pneumoniae* strain 49210 plasmid p49210-229.3, complete sequence | *Klebsiella pneumoniae* | 72382 | 3.80E+05 | 98% | 0 | 99.94 | 178873 | 229288 | CP089030.1 |
|  | *Klebsiella pneumoniae* strain 3214 plasmid pVIR_3214, complete sequence | *Klebsiella pneumoniae* | 72376 | 3.93E+05 | 96% | 0 | 99.94 | 178873 | 231431 | CP028852.1 |
|  | *Klebsiella pneumoniae* strain JS187-vir plasmid unnamed4, complete sequence | *Klebsiella pneumoniae* | 72362 | 3.69E+05 | 94% | 0 | 99.92 | 178873 | 226668 | MZ475703.1 |
|  | *Klebsiella pneumoniae* strain F16KP0002 plasmid pF16KP0002-1, complete sequence | *Klebsiella pneumoniae* | 72356 | 3.83E+05 | 98% | 0 | 99.92 | 178873 | 229504 | CP052204.1 |
|  | *Klebsiella pneumoniae* *subsp.* pneumoniae strain R210-2 plasmid pR210-2-vir, complete sequence | *Klebsiella pneumoniae* *subsp.* pneumoniae | 72354 | 3.99E+05 | 96% | 0 | 99.93 | 178873 | 229648 | CP034083.1 |
|  | *Klebsiella pneumoniae* strain NTUH-K2044-CR plasmid unnamed1, complete sequence | *Klebsiella pneumoniae* | 72354 | 3.86E+05 | 94% | 0 | 99.92 | 178873 | 227511 | MZ475709.1 |
|  | *Escherichia coli* strain J53-vir plasmid unnamed1, partial sequence | *Escherichia coli* | 72354 | 3.96E+05 | 94% | 0 | 99.92 | 178873 | 229894 | MZ475697.1 |
|  | *Klebsiella pneumoniae* strain KPN236 plasmid unnamed1, complete sequence | *Klebsiella pneumoniae* | 72351 | 3.86E+05 | 96% | 0 | 99.92 | 178873 | 231163 | CP072493.1 |
|  | *Klebsiella pneumoniae* strain ARLG-3254 plasmid pC308_1, complete sequence | *Klebsiella pneumoniae* | 72349 | 3.60E+05 | 94% | 0 | 99.92 | 178873 | 223720 | CP067778.1 |
|  | Klebsiella aerogenes strain NCTC9644 genome assembly, plasmid: 4 | Klebsiella aerogenes | 72347 | 3.77E+05 | 94% | 0 | 99.92 | 178873 | 224165 | LR134257.1 |
|  | *Klebsiella pneumoniae* strain SP4663 plasmid pIncHI1B_vir, complete sequence | *Klebsiella pneumoniae* | 72345 | 3.81E+05 | 96% | 0 | 99.93 | 178873 | 228289 | CP069366.1 |
|  | *Klebsiella pneumoniae* strain Kpn 1693 plasmid pKpn1693-Vir, complete sequence | *Klebsiella pneumoniae* | 72343 | 3.19E+05 | 76% | 0 | 99.93 | 178873 | 192347 | CP047596.1 |
|  | *Klebsiella pneumoniae* strain 11420 plasmid p11420-HVKP, complete sequence | *Klebsiella pneumoniae* | 72334 | 4.04E+05 | 96% | 0 | 99.91 | 178873 | 229796 | CP026024.1 |
|  | *Klebsiella pneumoniae* strain B17KP0067 plasmid pB17KP0067-1, complete sequence | *Klebsiella pneumoniae* | 72317 | 3.86E+05 | 98% | 0 | 99.92 | 178873 | 231026 | CP052495.1 |
|  | *Klebsiella pneumoniae* *subsp.* pneumoniae strain SC-7 plasmid pSC7-vir, complete sequence | *Klebsiella pneumoniae* *subsp.* pneumoniae | 72310 | 3.50E+05 | 94% | 0 | 99.9 | 178873 | 236809 | CP030270.1 |
|  | *Klebsiella pneumoniae* strain F17KP0012 plasmid pF17KP0012-1, complete sequence | *Klebsiella pneumoniae* | 72288 | 3.83E+05 | 98% | 0 | 99.9 | 178873 | 229470 | CP052144.1 |
|  | *Klebsiella pneumoniae* strain KP-Zhen plasmid unnamed1, complete sequence | *Klebsiella pneumoniae* | 72253 | 3.53E+05 | 94% | 0 | 99.89 | 178873 | 222711 | CP053863.1 |
|  | *Klebsiella pneumoniae* *subsp.* pneumoniae strain RJF999 plasmid pRJF999, complete sequence | *Klebsiella pneumoniae* *subsp.* pneumoniae | 72247 | 4.04E+05 | 96% | 0 | 99.89 | 178873 | 228907 | CP014011.1 |
|  | *Klebsiella pneumoniae* strain KPC-2 plasmid pKP169-P1, complete sequence | *Klebsiella pneumoniae* | 71603 | 4.22E+05 | 98% | 0 | 99.98 | 178873 | 221577 | CP078124.1 |
|  | *Klebsiella pneumoniae* *subsp.* pneumoniae strain 2566_Kpn plasmid phvKpST147_NDM-1_2566, complete sequence | *Klebsiella pneumoniae* *subsp.* pneumoniae | 70162 | 2.69E+05 | 65% | 0 | 99.95 | 178873 | 350403 | MW911671.1 |
|  | *Klebsiella pneumoniae* strain KP-PA plasmid pPAPIvir, complete sequence | *Klebsiella pneumoniae* | 70155 | 2.91E+05 | 66% | 0 | 99.94 | 178873 | 382377 | CP084985.1 |
|  | *Klebsiella pneumoniae* *subsp.* pneumoniae strain MRSN752019 plasmid pSI646A-ARMA-Vir-NDM, complete sequence | *Klebsiella pneumoniae* *subsp.* pneumoniae | 70149 | 2.75E+05 | 65% | 0 | 99.94 | 178873 | 374716 | CP084395.1 |
|  | *Klebsiella pneumoniae* strain KpvST147B_SE1_1_NDM plasmid pKpvST147B_virulence, complete sequence | *Klebsiella pneumoniae* | 70144 | 2.66E+05 | 65% | 0 | 99.94 | 178873 | 339117 | CP040726.1 |
|  | *Klebsiella pneumoniae* strain 1970_kpn plasmid phvKpST147_NDM-29, complete sequence | *Klebsiella pneumoniae* | 70118 | 2.54E+05 | 61% | 0 | 99.92 | 178873 | 311937 | CP066856.1 |
|  | *Klebsiella pneumoniae* strain KP-1Pi plasmid pKP-1PI_HIB-FIB, complete sequence | *Klebsiella pneumoniae* | 70112 | 2.66E+05 | 65% | 0 | 99.92 | 178873 | 340568 | CP071028.1 |
|  | *Klebsiella pneumoniae* *subsp.* pneumoniae strain 1657 plasmid phvKpST395_NDM1_1657, complete sequence | *Klebsiella pneumoniae* *subsp.* pneumoniae | 70105 | 2.63E+05 | 66% | 0 | 99.92 | 178873 | 388104 | CP072809.1 |
|  | *Klebsiella pneumoniae* strain KP-135LU plasmid pKP-135LU_HIB-FIB, complete sequence | *Klebsiella pneumoniae* | 70103 | 2.68E+05 | 65% | 0 | 99.92 | 178873 | 341276 | CP070891.1 |
|  | *Klebsiella pneumoniae* strain NMI7902/18 plasmid p7902/18_1, complete sequence | *Klebsiella pneumoniae* | 70077 | 2.55E+05 | 64% | 0 | 99.9 | 178873 | 310832 | ON081624.1 |
|  | *Klebsiella pneumoniae* strain NMI5419/18 plasmid p5419/18_1, complete sequence | *Klebsiella pneumoniae* | 70077 | 2.63E+05 | 65% | 0 | 99.9 | 178873 | 349155 | ON081623.1 |
|  | *Klebsiella pneumoniae* *subsp.* pneumoniae strain MRSN752165 plasmid pSI0739-ARMA-Vir, complete sequence | *Klebsiella pneumoniae* *subsp.* pneumoniae | 70075 | 2.51E+05 | 65% | 0 | 99.9 | 178873 | 335489 | CP074088.1 |
|  | *Klebsiella pneumoniae* *subsp.* pneumoniae strain KpvST383_NDM_OXA-48 plasmid pKpvST383L, complete sequence | *Klebsiella pneumoniae* *subsp.* pneumoniae | 70061 | 2.87E+05 | 65% | 0 | 99.91 | 178873 | 372826 | CP034201.2 |
|  | *Klebsiella pneumoniae* *subsp.* pneumoniae strain 2471_Kpn plasmid phvKpST874_NDM-1_2471, complete sequence | *Klebsiella pneumoniae* *subsp.* pneumoniae | 70048 | 2.67E+05 | 65% | 0 | 99.89 | 178873 | 335922 | MW911668.1 |
|  | *Klebsiella pneumoniae* strain NMI4313/18 plasmid p4313/18_1, complete sequence | *Klebsiella pneumoniae* | 70033 | 2.69E+05 | 65% | 0 | 99.88 | 178873 | 358573 | ON081621.1 |
|  | *Klebsiella pneumoniae* *subsp.* pneumoniae strain 1659 plasmid phvKpST147_NDM1_1659, complete sequence | *Klebsiella pneumoniae* *subsp.* pneumoniae | 70011 | 2.69E+05 | 65% | 0 | 99.87 | 178873 | 350476 | CP072810.1 |
|  | *Klebsiella pneumoniae* strain KP-26Pi plasmid pKP-26PI_HIB-FIB, complete sequence | *Klebsiella pneumoniae* | 70003 | 2.65E+05 | 64% | 0 | 99.88 | 178873 | 318691 | CP072926.1 |
|  | *Klebsiella pneumoniae* strain KP-12Pi plasmid pKP-12PI_HIB-FIB, complete sequence | *Klebsiella pneumoniae* | 69906 | 2.66E+05 | 65% | 0 | 99.83 | 178873 | 341914 | CP072918.1 |
|  | *Klebsiella pneumoniae* strain C2244 plasmid pCRHV-C2244, complete sequence | *Klebsiella pneumoniae* | 69510 | 4.15E+05 | 96% | 0 | 99.99 | 178873 | 293391 | MT644086.1 |
|  | *Klebsiella pneumoniae* strain GZ-1 plasmid unnamed1, complete sequence | *Klebsiella pneumoniae* | 69501 | 3.91E+05 | 86% | 0 | 99.99 | 178873 | 198126 | CP031935.1 |
|  | *Klebsiella pneumoniae* strain 21080237 plasmid p21080237_1, complete sequence | *Klebsiella pneumoniae* | 69433 | 3.75E+05 | 84% | 0 | 99.99 | 178873 | 188001 | CP095241.1 |
|  | *Klebsiella pneumoniae* strain CRKP-35 plasmid pCRKP-35_Vir, complete sequence | *Klebsiella pneumoniae* | 69309 | 3.93E+05 | 86% | 0 | 99.9 | 178873 | 198127 | CP102638.1 |
|  | *Klebsiella pneumoniae* *subsp.* pneumoniae strain HA2 plasmid pHA2-23-vir, complete sequence | *Klebsiella pneumoniae* *subsp.* pneumoniae | 68958 | 4.09E+05 | 99% | 0 | 99.73 | 178873 | 214938 | MH643786.1 |
|  | *Klebsiella pneumoniae* strain 108 plasmid pBJ108-HI3, complete sequence | *Klebsiella pneumoniae* | 68443 | 4.09E+05 | 96% | 0 | 99.7 | 178873 | 211194 | MW013144.1 |
|  | *Klebsiella pneumoniae* strain 21080937 plasmid p21080937_1, complete sequence | *Klebsiella pneumoniae* | 67606 | 2.88E+05 | 69% | 0 | 99.99 | 178873 | 157481 | CP095254.1 |
|  | *Klebsiella pneumoniae* strain IR5086 plasmid unnamed2, complete sequence | *Klebsiella pneumoniae* | 67115 | 2.21E+05 | 44% | 0 | 99.95 | 178873 | 266729 | CP061977.1 |
|  | *Klebsiella pneumoniae* strain JX-CR-hvKP-11 plasmid pJX11-1, complete sequence | *Klebsiella pneumoniae* | 67110 | 3.89E+05 | 96% | 0 | 99.96 | 178873 | 228028 | CP064209.1 |
|  | *Klebsiella pneumoniae* strain KPN115 plasmid pKPN115_1, complete sequence | *Klebsiella pneumoniae* | 67093 | 4.02E+05 | 95% | 0 | 99.95 | 178873 | 234056 | CP089860.1 |
|  | *Klebsiella pneumoniae* strain P1428 chromosome, complete genome | *Klebsiella pneumoniae* | 67091 | 4.19E+05 | 94% | 0 | 99.95 | 178873 | 5633290 | CP017994.1 |
|  | *Klebsiella pneumoniae* strain ED23 plasmid unamed, complete sequence | *Klebsiella pneumoniae* | 66611 | 3.62E+05 | 87% | 0 | 99.75 | 178873 | 212770 | CP016815.1 |
|  | *Klebsiella pneumoniae* strain JX-CR-hvKP-8 plasmid pJX8-1, complete sequence | *Klebsiella pneumoniae* | 66236 | 4.27E+05 | 100% | 0 | 99.97 | 178873 | 217869 | CP064218.1 |
|  | *Klebsiella pneumoniae* *subsp.* pneumoniae strain DD01653 plasmid pDD01653-1, complete sequence | *Klebsiella pneumoniae* *subsp.* pneumoniae | 66183 | 4.14E+05 | 100% | 0 | 99.97 | 178873 | 220277 | CP087652.1 |
|  | *Klebsiella pneumoniae* *subsp.* pneumoniae strain 2512_Kpn plasmid phvKpST395_NDM-1_2512, complete sequence | *Klebsiella pneumoniae* *subsp.* pneumoniae | 65861 | 2.89E+05 | 66% | 0 | 99.92 | 178873 | 422708 | MW911670.1 |
|  | *Klebsiella pneumoniae* *subsp.* pneumoniae strain KUH-KPNHVF1 plasmid unnamed, complete sequence | *Klebsiella pneumoniae* *subsp.* pneumoniae | 65824 | 3.69E+05 | 96% | 0 | 99.94 | 178873 | 229024 | CP047676.1 |
|  | *Klebsiella pneumoniae* *subsp.* pneumoniae strain KUH-KPNHVL1 plasmid unnamed, complete sequence | *Klebsiella pneumoniae* *subsp.* pneumoniae | 65819 | 3.69E+05 | 96% | 0 | 99.94 | 178873 | 229024 | CP047678.1 |
|  | *Klebsiella pneumoniae* strain 332306 plasmid p332306-HI3, complete sequence | *Klebsiella pneumoniae* | 65522 | 3.31E+05 | 78% | 0 | 99.92 | 178873 | 317970 | MK413722.1 |
|  | *Klebsiella pneumoniae* strain K194 plasmid pK194-P1, complete sequence | *Klebsiella pneumoniae* | 65270 | 4.26E+05 | 99% | 0 | 100 | 178873 | 241781 | CP102436.1 |
| pCR-hvKP173-Vir-P2 | *Klebsiella pneumoniae* strain ZY393 plasmid pZY1393-2, complete sequence | *Klebsiella pneumoniae* | 1.52E+05 | 2.89E+05 | 77% | 0 | 99.97 | 144880 | 134874 | CP076673.1 |
|  | *Klebsiella pneumoniae* *subsp.* pneumoniae strain SCKP020079 plasmid pKPC2_020079, complete sequence | *Klebsiella pneumoniae* *subsp.* pneumoniae | 1.36E+05 | 3.89E+05 | 100% | 0 | 100 | 144880 | 146790 | CP029381.1 |
|  | *Klebsiella pneumoniae* strain F1 plasmid pF1_1, complete sequence | *Klebsiella pneumoniae* | 1.34E+05 | 4.04E+05 | 93% | 0 | 99.99 | 144880 | 164510 | CP026131.1 |
|  | *Klebsiella pneumoniae* strain HvKp-su1 plasmid unnamed1, complete sequence | *Klebsiella pneumoniae* | 1.34E+05 | 3.69E+05 | 93% | 0 | 99.99 | 144880 | 133346 | CP092718.1 |
|  | *Klebsiella pneumoniae* strain FDAARGOS_444 plasmid unnamed2 | *Klebsiella pneumoniae* | 1.34E+05 | 4.33E+05 | 93% | 0 | 99.98 | 144880 | 187926 | CP023942.1 |
|  | *Klebsiella pneumoniae* strain BSI014 plasmid pBSI014-KPC2 | *Klebsiella pneumoniae* | 1.34E+05 | 4.39E+05 | 93% | 0 | 99.95 | 144880 | 170701 | MT269822.1 |
|  | *Klebsiella pneumoniae* strain L388 plasmid pKPC-L388 | *Klebsiella pneumoniae* | 1.24E+05 | 3.68E+05 | 98% | 0 | 100 | 144880 | 145851 | CP029225.1 |
|  | *Klebsiella pneumoniae* strain 9489 plasmid pBJ9489-KPC | *Klebsiella pneumoniae* | 1.24E+05 | 3.37E+05 | 92% | 0 | 99.99 | 144880 | 132246 | MN821371.1 |
|  | *Klebsiella pneumoniae* strain XHKPN083 plasmid pXHKPN083-2, complete sequence | *Klebsiella pneumoniae* | 1.16E+05 | 3.30E+05 | 63% | 0 | 99.99 | 144880 | 122553 | CP066912.1 |
|  | *Klebsiella pneumoniae* strain WCHKP115069 plasmid pKPC2_115069, complete sequence | *Klebsiella pneumoniae* | 1.16E+05 | 3.59E+05 | 89% | 0 | 99.98 | 144880 | 154986 | CP033404.1 |
|  | *Klebsiella pneumoniae* *subsp.* pneumoniae strain WCHKP015093 plasmid pKPC2_015093, complete sequence | *Klebsiella pneumoniae* *subsp.* pneumoniae | 1.16E+05 | 3.75E+05 | 89% | 0 | 99.97 | 144880 | 154724 | CP036301.1 |
|  | *Klebsiella pneumoniae* strain WCHKP649 plasmid pKPC2_095649, complete sequence | *Klebsiella pneumoniae* | 1.16E+05 | 3.77E+05 | 89% | 0 | 99.97 | 144880 | 156099 | CP026584.1 |
|  | *Klebsiella pneumoniae* strain WCHKP115038 plasmid pKPC2_115038, complete sequence | *Klebsiella pneumoniae* | 1.16E+05 | 3.52E+05 | 88% | 0 | 99.97 | 144880 | 153486 | CP043603.1 |
|  | *Klebsiella pneumoniae* strain WCHKP090050 plasmid pKPC2_090050, complete sequence | *Klebsiella pneumoniae* | 1.16E+05 | 3.54E+05 | 88% | 0 | 99.97 | 144880 | 154724 | CP043370.1 |
|  | *Klebsiella pneumoniae* strain WCHKP090045 plasmid pKPC2_090045, complete sequence | *Klebsiella pneumoniae* | 1.16E+05 | 3.54E+05 | 88% | 0 | 99.97 | 144880 | 154724 | CP043366.1 |
|  | *Klebsiella pneumoniae* *subsp.* pneumoniae strain WCHKP020120 plasmid pKPC2_020120, complete sequence | *Klebsiella pneumoniae* *subsp.* pneumoniae | 1.16E+05 | 3.64E+05 | 88% | 0 | 99.97 | 144880 | 154719 | CP043358.1 |
|  | *Klebsiella pneumoniae* strain WCHKP090374 plasmid pKPC2_090374, complete sequence | *Klebsiella pneumoniae* | 1.16E+05 | 3.76E+05 | 89% | 0 | 99.96 | 144880 | 154728 | CP066536.1 |
|  | *Klebsiella pneumoniae* strain 140253 plasmid pKPC2_140253, complete sequence | *Klebsiella pneumoniae* | 1.16E+05 | 3.55E+05 | 89% | 0 | 99.96 | 144880 | 155793 | CP097627.1 |
|  | *Klebsiella pneumoniae* strain F127 plasmid pF127_1, complete sequence | *Klebsiella pneumoniae* | 1.11E+05 | 3.84E+05 | 93% | 0 | 99.97 | 144880 | 164501 | CP026141.1 |
|  | *Klebsiella pneumoniae* strain BSI047 plasmid pBSI047-KPC2 | *Klebsiella pneumoniae* | 1.09E+05 | 3.60E+05 | 79% | 0 | 99.95 | 144880 | 139571 | MT269830.1 |
|  | *Klebsiella pneumoniae* strain KP137060 plasmid unnamed2, complete sequence | *Klebsiella pneumoniae* | 1.08E+05 | 3.51E+05 | 97% | 0 | 99.93 | 144880 | 146878 | MW218143.1 |
|  | *Klebsiella pneumoniae* strain SWU01 plasmid unnamed, complete sequence | *Klebsiella pneumoniae* | 1.07E+05 | 4.04E+05 | 93% | 0 | 99.99 | 144880 | 162552 | CP018455.1 |
|  | *Klebsiella pneumoniae* strain 20049 plasmid p20049-KPC, complete sequence | *Klebsiella pneumoniae* | 1.07E+05 | 4.14E+05 | 99% | 0 | 100 | 144880 | 151653 | MF168404.1 |
|  | *Klebsiella pneumoniae* strain 381810-51 plasmid p181051-KPC, complete sequence | *Klebsiella pneumoniae* | 1.07E+05 | 2.70E+05 | 68% | 0 | 99.99 | 144880 | 303071 | MT920903.1 |
|  | *Klebsiella pneumoniae* strain WCHKP115011 plasmid pKPC2_115011, complete sequence | *Klebsiella pneumoniae* | 1.07E+05 | 4.20E+05 | 86% | 0 | 99.97 | 144880 | 157388 | CP089954.1 |
|  | *Klebsiella pneumoniae* strain C2660 plasmid pC2660-3-KPC, complete sequence | *Klebsiella pneumoniae* | 1.07E+05 | 3.40E+05 | 88% | 0 | 99.97 | 144880 | 153556 | CP039810.1 |
|  | *Klebsiella pneumoniae* *subsp.* pneumoniae strain RJBSI76-pV plasmid pRJBSI76-pV-3, complete sequence | *Klebsiella pneumoniae* *subsp.* pneumoniae | 1.06E+05 | 3.68E+05 | 90% | 0 | 99.97 | 144880 | 184751 | CP068687.1 |
|  | *Klebsiella pneumoniae* *subsp.* pneumoniae strain RJBSI76 plasmid pRJBSI76-3, complete sequence | *Klebsiella pneumoniae* *subsp.* pneumoniae | 1.06E+05 | 3.68E+05 | 90% | 0 | 99.97 | 144880 | 184748 | CP068692.1 |
|  | *Klebsiella pneumoniae* strain JNKPN26 plasmid pJNKPN26_KPC, complete sequence | *Klebsiella pneumoniae* | 1.06E+05 | 3.53E+05 | 88% | 0 | 99.99 | 144880 | 126203 | CP090204.1 |
|  | *Klebsiella pneumoniae* strain F44 plasmid p44-2, complete sequence | *Klebsiella pneumoniae* | 1.05E+05 | 4.22E+05 | 89% | 0 | 99.97 | 144880 | 161580 | CP025463.1 |
|  | *Klebsiella pneumoniae* strain BSI055 plasmid pBSI055-KPC2 | *Klebsiella pneumoniae* | 1.04E+05 | 3.73E+05 | 78% | 0 | 100 | 144880 | 142890 | MT269834.1 |
|  | *Klebsiella pneumoniae* strain BSI032 plasmid pBSI032-KPC2 | *Klebsiella pneumoniae* | 1.04E+05 | 3.94E+05 | 78% | 0 | 100 | 144880 | 132173 | MT269827.1 |
|  | *Klebsiella pneumoniae* strain BSI010 plasmid pBSI010-KPC2 | *Klebsiella pneumoniae* | 1.04E+05 | 3.83E+05 | 78% | 0 | 100 | 144880 | 146268 | MT269820.1 |
|  | *Klebsiella pneumoniae* strain 675920 plasmid p675920-1, complete sequence | *Klebsiella pneumoniae* | 1.04E+05 | 3.94E+05 | 92% | 0 | 100 | 144880 | 163995 | MF133495.1 |
|  | *Klebsiella pneumoniae* strain 33367 plasmid p33367_KPC2, complete sequence | *Klebsiella pneumoniae* | 1.04E+05 | 4.28E+05 | 100% | 0 | 99.99 | 144880 | 150096 | CP099415.1 |
|  | *Klebsiella pneumoniae* strain 2014042281 plasmid p42281-KPC, complete sequence | *Klebsiella pneumoniae* | 1.04E+05 | 2.89E+05 | 65% | 0 | 99.99 | 144880 | 115305 | MT810369.1 |
|  | *Klebsiella pneumoniae* strain 49088 plasmid p49088-279.2, complete sequence | *Klebsiella pneumoniae* | 1.04E+05 | 4.35E+05 | 92% | 0 | 99.99 | 144880 | 279210 | CP089000.1 |
|  | *Klebsiella pneumoniae* strain CRKP78R plasmid p3, complete sequence | *Klebsiella pneumoniae* | 1.04E+05 | 4.33E+05 | 99% | 0 | 99.99 | 144880 | 149407 | CP066256.1 |
|  | *Klebsiella pneumoniae* strain KP1880 plasmid pKPC1880, complete sequence | *Klebsiella pneumoniae* | 1.04E+05 | 3.99E+05 | 93% | 0 | 99.99 | 144880 | 168960 | CP061347.1 |
|  | *Klebsiella pneumoniae* strain IR5077_1 plasmid unnamed3, complete sequence | *Klebsiella pneumoniae* | 1.04E+05 | 2.81E+05 | 59% | 0 | 99.99 | 144880 | 292919 | CP097673.1 |
|  | *Klebsiella pneumoniae* strain A1708 plasmid pA1708-KPC, complete sequence | *Klebsiella pneumoniae* | 1.04E+05 | 4.57E+05 | 96% | 0 | 99.99 | 144880 | 173280 | MT810354.1 |
|  | *Klebsiella pneumoniae* strain F726925 plasmid pF726925-1, complete sequence | *Klebsiella pneumoniae* | 1.04E+05 | 4.36E+05 | 93% | 0 | 99.99 | 144880 | 172862 | CP081821.1 |
|  | *Klebsiella pneumoniae* strain 911021 plasmid p911021-KPC, complete sequence | *Klebsiella pneumoniae* | 1.04E+05 | 4.32E+05 | 95% | 0 | 99.99 | 144880 | 169824 | MK036888.1 |
|  | *Klebsiella pneumoniae* *subsp.* pneumoniae strain SH9 plasmid pSH9-CTX-TEM, complete sequence | *Klebsiella pneumoniae* *subsp.* pneumoniae | 1.04E+05 | 2.15E+05 | 54% | 0 | 99.99 | 144880 | 98684 | MH255829.1 |
|  | *Klebsiella pneumoniae* strain KPN361 plasmid pKPN361-1, complete sequence | *Klebsiella pneumoniae* | 1.04E+05 | 4.22E+05 | 94% | 0 | 99.99 | 144880 | 169824 | CP053017.1 |
|  | *Escherichia coli* strain HNEC55 plasmid pHNEC55, complete sequence | *Escherichia coli* | 1.04E+05 | 1.84E+05 | 53% | 0 | 99.99 | 144880 | 81498 | KT879914.1 |
|  | *Klebsiella pneumoniae* strain SH12 plasmid pSH12_KPC, complete sequence | *Klebsiella pneumoniae* | 1.04E+05 | 4.17E+05 | 92% | 0 | 99.98 | 144880 | 167468 | CP040835.1 |
|  | *Klebsiella pneumoniae* strain BSI054 plasmid pBSI054-KPC2 | *Klebsiella pneumoniae* | 1.04E+05 | 4.31E+05 | 93% | 0 | 99.98 | 144880 | 168038 | MT269833.1 |
|  | *Klebsiella pneumoniae* strain BSI052 plasmid pBSI052-KPC2 | *Klebsiella pneumoniae* | 1.04E+05 | 4.16E+05 | 93% | 0 | 99.98 | 144880 | 165819 | MT269832.1 |
|  | *Klebsiella pneumoniae* strain WCHKP2 plasmid pKPC2_020002, complete sequence | *Klebsiella pneumoniae* | 1.04E+05 | 4.37E+05 | 93% | 0 | 99.98 | 144880 | 177516 | CP028541.2 |
|  | Citrobacter werkmanii strain LYYSPS2 plasmid pLYYSPS2-3, complete sequence | Citrobacter werkmanii | 1.04E+05 | 1.80E+05 | 49% | 0 | 99.98 | 144880 | 87232 | MZ342958.1 |
|  | *Klebsiella pneumoniae* *subsp.* pneumoniae strain DD01754 plasmid pDD01754-2, complete sequence | *Klebsiella pneumoniae* *subsp.* pneumoniae | 1.04E+05 | 4.50E+05 | 93% | 0 | 99.98 | 144880 | 182556 | CP087647.1 |
|  | *Klebsiella pneumoniae* *subsp.* pneumoniae strain DD01304 plasmid pDD01304-2, complete sequence | *Klebsiella pneumoniae* *subsp.* pneumoniae | 1.04E+05 | 3.88E+05 | 93% | 0 | 99.98 | 144880 | 169136 | CP087608.1 |
|  | *Klebsiella pneumoniae* strain JX-CR-hvKP-2 plasmid pJX2-2, complete sequence | *Klebsiella pneumoniae* | 1.04E+05 | 4.06E+05 | 93% | 0 | 99.98 | 144880 | 168025 | CP064248.1 |
|  | *Klebsiella pneumoniae* strain WCHKP020037 plasmid pKPC2_020037, complete sequence | *Klebsiella pneumoniae* | 1.04E+05 | 4.41E+05 | 93% | 0 | 99.98 | 144880 | 172770 | CP036372.1 |
|  | *Klebsiella pneumoniae* strain 08291 plasmid pW08291-KPC, complete sequence | *Klebsiella pneumoniae* | 1.04E+05 | 4.09E+05 | 94% | 0 | 99.98 | 144880 | 169804 | MN842295.1 |
|  | *Klebsiella pneumoniae* strain JX-CR-hvKP-1 plasmid pJX1-1, complete sequence | *Klebsiella pneumoniae* | 1.03E+05 | 4.05E+05 | 92% | 0 | 99.97 | 144880 | 167834 | CP064253.1 |
|  | *Klebsiella pneumoniae* strain 12139 plasmid p12139-KPC, complete sequence | *Klebsiella pneumoniae* | 1.03E+05 | 4.22E+05 | 95% | 0 | 99.96 | 144880 | 169424 | MF168403.1 |
|  | *Klebsiella pneumoniae* strain 7849 plasmid pKP7849_KPC, complete sequence | *Klebsiella pneumoniae* | 1.03E+05 | 4.23E+05 | 99% | 0 | 99.96 | 144880 | 148977 | MW478298.1 |
|  | *Klebsiella pneumoniae* strain CRKP-30 plasmid pCRKP-30_KPC, complete sequence |  | 1.03E+05 | 4.01E+05 | 90% | 0 | 99.94 | 144880 | 158524 | CP102634.1 |
|  | *Klebsiella pneumoniae* strain 64917 plasmid p64917-KPC, complete sequence | *Klebsiella pneumoniae* | 1.03E+05 | 4.21E+05 | 95% | 0 | 99.94 | 144880 | 169419 | MF168405.1 |
|  | *Escherichia coli* strain XD35 plasmid pXD35004, complete sequence | *Escherichia coli* | 1.03E+05 | 1.92E+05 | 56% | 0 | 99.87 | 144880 | 85891 | CP089137.1 |
|  | *Klebsiella pneumoniae* strain 246421 plasmid p246421-KPC, complete sequence | *Klebsiella pneumoniae* | 1.02E+05 | 3.75E+05 | 82% | 0 | 99.99 | 144880 | 145209 | MT810356.1 |
|  | *Escherichia coli* strain HZMPC32 plasmid pHNMPC32, complete sequence | *Escherichia coli* | 1.02E+05 | 1.76E+05 | 53% | 0 | 99.99 | 144880 | 74768 | MG197499.1 |
|  | *Escherichia coli* strain HNC02 plasmid pHNHNC02, complete sequence | *Escherichia coli* | 1.02E+05 | 1.90E+05 | 53% | 0 | 99.98 | 144880 | 76869 | MG197497.1 |
|  | *Klebsiella pneumoniae* p477Kp plasmid, complete sequence | *Klebsiella pneumoniae* | 1.02E+05 | 1.79E+05 | 56% | 0 | 99.98 | 144880 | 74768 | LN897475.2 |
|  | *Klebsiella pneumoniae* p397Kp plasmid, complete sequence | *Klebsiella pneumoniae* | 1.02E+05 | 1.94E+05 | 56% | 0 | 99.98 | 144880 | 76863 | LN897474.2 |
|  | Enterobacter cloacae strain CBG15936 plasmid pTEM-CBG, complete sequence | Enterobacter cloacae | 1.02E+05 | 1.78E+05 | 56% | 0 | 99.97 | 144880 | 75044 | CP046117.1 |
|  | *Escherichia coli* strain 7A8 plasmid pHN7A8, complete sequence | *Escherichia coli* | 1.02E+05 | 1.93E+05 | 56% | 0 | 99.96 | 144880 | 76878 | JN232517.1 |
|  | *Klebsiella pneumoniae* *subsp.* pneumoniae strain HA2 plasmid pHA2-23-KPC, complete sequence | *Klebsiella pneumoniae* *subsp.* pneumoniae | 1.01E+05 | 3.83E+05 | 99% | 0 | 99.99 | 144880 | 148749 | MH643789.1 |
|  | *Klebsiella pneumoniae* *subsp.* pneumoniae strain SH2 plasmid pSH2-85K-MDR, complete sequence | *Klebsiella pneumoniae* *subsp.* pneumoniae | 99332 | 3.84E+05 | 98% | 0 | 100 | 144880 | 149033 | MH643792.1 |
|  | *Klebsiella pneumoniae* strain FZKP4523 plasmid pKPC-2_FZKP4523, complete sequence | *Klebsiella pneumoniae* | 99020 | 3.71E+05 | 83% | 0 | 99.98 | 144880 | 141697 | CP101534.1 |
|  | *Klebsiella pneumoniae* strain Kp36 plasmid unnamed2, complete sequence | *Klebsiella pneumoniae* | 98785 | 4.16E+05 | 97% | 0 | 99.99 | 144880 | 142228 | CP047194.1 |
|  | *Klebsiella pneumoniae* strain IR12197_1 plasmid unnamed4, complete sequence | *Klebsiella pneumoniae* | 97703 | 3.05E+05 | 84% | 0 | 99.98 | 144880 | 159393 | CP097707.1 |
|  | *Klebsiella pneumoniae* strain QL24 plasmid pKPC-QL24, complete sequence | *Klebsiella pneumoniae* | 96641 | 3.27E+05 | 88% | 0 | 99.94 | 144880 | 126126 | MH263653.1 |
|  | *Klebsiella pneumoniae* strain 21080534 plasmid p21080534_1, complete sequence | *Klebsiella pneumoniae* | 96630 | 3.29E+05 | 86% | 0 | 100 | 144880 | 210914 | CP095248.1 |
|  | [*Klebsiella pneumoniae* KP18-3-8] pKP18-3-8_KPC plasmid, complete cds | *Klebsiella pneumoniae* | 96235 | 2.89E+05 | 73% | 0 | 100 | 144880 | 130717 | MT232812.1 |
|  | *Klebsiella pneumoniae* strain JNKPN26 plasmid pJNKPN26_KPC, complete sequence | *Klebsiella pneumoniae* | 95746 | 3.60E+05 | 88% | 0 | 99.99 | 144880 | 126203 | MZ546615.1 |
|  | *Klebsiella pneumoniae* strain KP18-2079 plasmid pKP18-2079_KPC, complete sequence | *Klebsiella pneumoniae* | 94928 | 2.09E+05 | 62% | 0 | 99.98 | 144880 | 186564 | MT090959.1 |
|  | *Klebsiella pneumoniae* strain CDI694 plasmid pCDI694-140.8, complete sequence | *Klebsiella pneumoniae* | 94918 | 3.78E+05 | 96% | 0 | 99.97 | 144880 | 140828 | CP077777.1 |
|  | *Klebsiella pneumoniae* strain 8695 plasmid pFK8695-KPC-33, complete sequence | *Klebsiella pneumoniae* | 94917 | 4.12E+05 | 96% | 0 | 99.97 | 144880 | 143980 | CP085890.1 |
|  | *Klebsiella pneumoniae* strain 150040X1B1 plasmid pCTXM65_150040X1B1, complete sequence | *Klebsiella pneumoniae* | 94911 | 3.63E+05 | 83% | 0 | 99.97 | 144880 | 149214 | CP101727.1 |
|  | *Klebsiella pneumoniae* strain WCHKP090357 plasmid pKPC2_090357, complete sequence | *Klebsiella pneumoniae* | 94911 | 3.52E+05 | 86% | 0 | 99.97 | 144880 | 149116 | CP066524.1 |
|  | *Escherichia coli* strain OW1E2 plasmid pOW1E2a, complete sequence | *Escherichia coli* | 94872 | 2.19E+05 | 53% | 0 | 99.96 | 144880 | 108766 | CP067246.1 |
|  | *Klebsiella pneumoniae* strain BSI074 plasmid pBSI074-KPC2 | *Klebsiella pneumoniae* | 94870 | 3.62E+05 | 93% | 0 | 99.97 | 144880 | 135737 | MT269848.1 |
|  | *Escherichia coli* strain fEC.1 plasmid pfEC.1-3, complete sequence | *Escherichia coli* | 94821 | 1.75E+05 | 49% | 0 | 99.94 | 144880 | 78319 | OK605583.1 |
|  | *Escherichia coli* strain NT1N25 plasmid pNT1N25-76kb, complete sequence | *Escherichia coli* | 94821 | 1.89E+05 | 48% | 0 | 99.93 | 144880 | 76891 | CP075483.1 |
|  | *Klebsiella pneumoniae* strain HS2536 plasmid pZHKPC2, complete sequence | *Klebsiella pneumoniae* | 94784 | 2.96E+05 | 77% | 0 | 100 | 144880 | 109901 | OM928503.1 |
|  | *Klebsiella pneumoniae* plasmid p1512-KPC, complete sequence | *Klebsiella pneumoniae* | 94678 | 3.22E+05 | 72% | 0 | 99.97 | 144880 | 117697 | MF918372.1 |
|  | *Escherichia coli* strain HNEC46 plasmid PHNEC46, complete sequence | *Escherichia coli* | 93533 | 1.33E+05 | 50% | 0 | 99.99 | 144880 | 74046 | KX503323.1 |
|  | *Klebsiella pneumoniae* strain 135077 plasmid p1_135077, complete sequence | *Klebsiella pneumoniae* | 93397 | 3.16E+05 | 80% | 0 | 99.97 | 144880 | 139526 | CP073293.1 |
|  | *Klebsiella pneumoniae* strain HZMPC51-2 plasmid pHNMPC51, complete sequence | *Klebsiella pneumoniae* | 92283 | 1.60E+05 | 47% | 0 | 99.99 | 144880 | 69654 | MG197500.1 |
|  | *Klebsiella pneumoniae* strain HZMPC43 plasmid pHNMPC43, complete sequence | *Klebsiella pneumoniae* | 92233 | 1.60E+05 | 47% | 0 | 99.97 | 144880 | 69666 | MG197501.1 |
|  | Escherichia fergusonii strain EFCF056 plasmid pEF02, complete sequence | Escherichia fergusonii | 92152 | 2.11E+05 | 51% | 0 | 99.94 | 144880 | 90871 | CP040807.1 |
|  | *Klebsiella pneumoniae* strain 116753 plasmid p116753-KPC, complete sequence | *Klebsiella pneumoniae* | 91281 | 2.18E+05 | 51% | 0 | 99.62 | 144880 | 137873 | MN891682.1 |
|  | *Klebsiella pneumoniae* strain KP19-3138 plasmid pKP19-3138-4, complete sequence | *Klebsiella pneumoniae* | 91275 | 2.16E+05 | 50% | 0 | 99.62 | 144880 | 95171 | CP090620.1 |
|  | *Klebsiella pneumoniae* strain CRKP66R plasmid pCRKP66R-3, complete sequence | *Klebsiella pneumoniae* | 91225 | 3.57E+05 | 88% | 0 | 99.99 | 144880 | 128675 | CP063835.1 |
|  | *Escherichia coli* MH13-051M plasmid pMH13-051M_1 DNA, complete genome | *Escherichia coli* | 89942 | 2.23E+05 | 51% | 0 | 99.14 | 144880 | 111544 | AP018572.2 |
|  | *Klebsiella pneumoniae* *subsp.* pneumoniae strain KPN857 plasmid pB, complete sequence | *Klebsiella pneumoniae* *subsp.* pneumoniae | 87002 | 3.28E+05 | 78% | 0 | 100 | 144880 | 134873 | CP090434.1 |
|  | *Klebsiella pneumoniae* strain CY814036 plasmid pCY814036-KPC2, complete sequence | *Klebsiella pneumoniae* | 86158 | 3.55E+05 | 88% | 0 | 99.99 | 144880 | 140105 | CP093153.1 |
| pCR-hvKP173-KPC-P3 | *Klebsiella pneumoniae* strain KP20194b2 plasmid pKP20194b2-p3, complete sequence | *Klebsiella pneumoniae* | 80524 | 1.84E+05 | 99% | 0 | 100 | 87872 | 87095 | CP054765.1 |
|  | *Klebsiella pneumoniae* strain KP18-2079 plasmid pKP18-2079_tetA, complete sequence | *Klebsiella pneumoniae* | 80524 | 1.75E+05 | 97% | 0 | 100 | 87872 | 84699 | MT090960.1 |
|  | *Klebsiella pneumoniae* strain L39_2 plasmid p4_L39, complete sequence | *Klebsiella pneumoniae* | 80524 | 1.84E+05 | 99% | 0 | 100 | 87872 | 87095 | CP033957.1 |
|  | *Klebsiella pneumoniae* strain BJCFK909 plasmid p3s1, complete sequence | *Klebsiella pneumoniae* | 80524 | 1.81E+05 | 97% | 0 | 100 | 87872 | 85665 | CP034126.1 |
|  | *Klebsiella pneumoniae* *subsp.* pneumoniae strain SCKP020079 plasmid pLAP2_020079, complete sequence | *Klebsiella pneumoniae* *subsp.* pneumoniae | 80524 | 1.80E+05 | 96% | 0 | 100 | 87872 | 85185 | CP029382.1 |
|  | *Klebsiella pneumoniae* strain L388 plasmid p4-L388 | *Klebsiella pneumoniae* | 80524 | 1.84E+05 | 99% | 0 | 100 | 87872 | 87095 | CP029223.1 |
|  | *Klebsiella pneumoniae* strain 675920 plasmid p675920-2, complete sequence | *Klebsiella pneumoniae* | 80524 | 1.60E+05 | 92% | 0 | 100 | 87872 | 79372 | MF133496.1 |
|  | *Klebsiella pneumoniae* strain K64 plasmid pTET-4, complete sequence | *Klebsiella pneumoniae* | 80524 | 1.75E+05 | 97% | 0 | 100 | 87872 | 84876 | CP102394.1 |
|  | *Klebsiella pneumoniae* strain S234 plasmid pS234-2, complete sequence | *Klebsiella pneumoniae* | 80524 | 2.13E+05 | 100% | 0 | 100 | 87872 | 146760 | CP102188.1 |
|  | *Klebsiella pneumoniae* strain hvKP340 plasmid unnamed7, complete sequence | *Klebsiella pneumoniae* | 80524 | 1.75E+05 | 97% | 0 | 100 | 87872 | 84876 | CP101783.1 |
|  | *Klebsiella pneumoniae* strain hvKP323 plasmid unnamed2, complete sequence | *Klebsiella pneumoniae* | 80524 | 1.75E+05 | 97% | 0 | 100 | 87872 | 84876 | CP101772.1 |
|  | *Klebsiella pneumoniae* strain hvKP841 plasmid unnamed4, complete sequence | *Klebsiella pneumoniae* | 80524 | 1.75E+05 | 97% | 0 | 100 | 87872 | 84876 | CP101788.1 |
|  | *Klebsiella pneumoniae* strain hvKP319 plasmid unnamed3, complete sequence | *Klebsiella pneumoniae* | 80524 | 1.75E+05 | 97% | 0 | 100 | 87872 | 84876 | CP101767.1 |
|  | *Klebsiella pneumoniae* *subsp.* pneumoniae strain DD02280 plasmid pDD02280-3 | *Klebsiella pneumoniae* *subsp.* pneumoniae | 80524 | 1.69E+05 | 94% | 0 | 100 | 87872 | 91826 | CP087626.1 |
|  | *Klebsiella pneumoniae* strain KP15 plasmid unnamed3, complete sequence | *Klebsiella pneumoniae* | 80524 | 1.87E+05 | 99% | 0 | 100 | 87872 | 99065 | CP087145.1 |
|  | *Klebsiella pneumoniae* strain KP16 plasmid unnamed3, complete sequence | *Klebsiella pneumoniae* | 80524 | 1.84E+05 | 99% | 0 | 100 | 87872 | 87095 | CP087149.1 |
|  | *Klebsiella pneumoniae* strain KP14 plasmid unnamed3, complete sequence | *Klebsiella pneumoniae* | 80524 | 1.84E+05 | 99% | 0 | 100 | 87872 | 87095 | CP087154.1 |
|  | *Klebsiella pneumoniae* strain XH1507 plasmid pXH1507-3, complete sequence | *Klebsiella pneumoniae* | 80524 | 1.84E+05 | 99% | 0 | 100 | 87872 | 87095 | CP092796.1 |
|  | *Klebsiella pneumoniae* strain XH1508 plasmid pXH1508-3, complete sequence | *Klebsiella pneumoniae* | 80524 | 1.84E+05 | 99% | 0 | 100 | 87872 | 87095 | CP092789.1 |
|  | *Klebsiella pneumoniae* *subsp.* pneumoniae strain KPN857 plasmid pC, complete sequence | *Klebsiella pneumoniae* *subsp.* pneumoniae | 80524 | 1.84E+05 | 99% | 0 | 100 | 87872 | 87095 | CP090435.1 |
|  | *Klebsiella pneumoniae* strain KP200731214 plasmid pKP-QnrS1 | *Klebsiella pneumoniae* | 80524 | 1.70E+05 | 94% | 0 | 100 | 87872 | 82121 | CP084747.1 |
|  | *Klebsiella pneumoniae* strain 12 plasmid pKP12_4, complete sequence | *Klebsiella pneumoniae* | 80524 | 1.84E+05 | 99% | 0 | 100 | 87872 | 87095 | CP082768.1 |
|  | Klebsiella sp. P1954 plasmid p00003, complete sequence | Klebsiella sp. P1954 | 80524 | 1.84E+05 | 99% | 0 | 100 | 87872 | 87045 | CP073375.1 |
|  | *Klebsiella pneumoniae* strain KPWX136 plasmid pD, complete sequence | *Klebsiella pneumoniae* | 80524 | 1.84E+05 | 99% | 0 | 100 | 87872 | 87095 | CP069176.1 |
|  | *Klebsiella pneumoniae* strain CRKP78R plasmid pCRKP78R-4-tetA, complete sequence | *Klebsiella pneumoniae* | 80524 | 1.84E+05 | 98% | 0 | 100 | 87872 | 86962 | CP066257.1 |
|  | *Klebsiella pneumoniae* strain JX-CR-hvKP-10 plasmid pJX10-3, complete sequence | *Klebsiella pneumoniae* | 80524 | 1.35E+05 | 83% | 0 | 100 | 87872 | 77551 | CP064261.1 |
|  | *Klebsiella pneumoniae* strain JX-CR-hvKP-4 plasmid pJX4-3, complete sequence | *Klebsiella pneumoniae* | 80524 | 1.84E+05 | 99% | 0 | 100 | 87872 | 87095 | CP064238.1 |
|  | *Klebsiella pneumoniae* strain M911-1 plasmid pM911-1.1, complete sequence | *Klebsiella pneumoniae* | 80524 | 1.50E+05 | 84% | 0 | 100 | 87872 | 75711 | CP064130.1 |
|  | *Klebsiella pneumoniae* strain h2 plasmid pKPC-h2, complete sequence | *Klebsiella pneumoniae* | 80524 | 1.35E+05 | 82% | 0 | 100 | 87872 | 77551 | MT550691.1 |
|  | *Klebsiella pneumoniae* *subsp.* pneumoniae strain SH2 plasmid pSH2-vir, complete sequence | *Klebsiella pneumoniae* *subsp.* pneumoniae | 80524 | 1.80E+05 | 96% | 0 | 100 | 87872 | 85185 | MH643790.1 |
|  | *Klebsiella pneumoniae* strain 16ZR-187 plasmid p16ZR-187-IncFII-83-R, complete sequence | *Klebsiella pneumoniae* | 80524 | 1.73E+05 | 96% | 0 | 100 | 87872 | 83551 | MN182748.1 |
|  | *Klebsiella pneumoniae* strain S270v plasmid pS270V-4, complete sequence | *Klebsiella pneumoniae* | 80518 | 1.84E+05 | 99% | 0 | 100 | 87872 | 87095 | CP102194.1 |
|  | *Klebsiella pneumoniae* strain FRPDR plasmid pFRPDR_4, complete sequence | *Klebsiella pneumoniae* | 80518 | 1.15E+05 | 69% | 0 | 100 | 87872 | 61413 | CP063763.1 |
|  | *Klebsiella pneumoniae* strain KP1064WHY plasmid p3, complete sequence | *Klebsiella pneumoniae* | 80489 | 1.82E+05 | 97% | 0 | 99.98 | 87872 | 124300 | CP084708.1 |
|  | *Klebsiella pneumoniae* strain 8695 plasmid pFK8695-tetA, complete sequence | *Klebsiella pneumoniae* | 80487 | 1.84E+05 | 99% | 0 | 99.98 | 87872 | 87106 | CP085892.1 |
|  | *Klebsiella pneumoniae* isolate 98fb0f42-b809-11e8-aae5-3c4a9275d6c8 genome assembly, chromosome: 1 | *Klebsiella pneumoniae* | 80441 | 1.91E+05 | 94% | 0 | 99.96 | 87872 | 5716474 | LR596807.1 |
|  | *Klebsiella pneumoniae* strain XJ-K2 plasmid unnamed3, complete sequence | *Klebsiella pneumoniae* | 80439 | 1.75E+05 | 97% | 0 | 99.97 | 87872 | 84855 | CP032243.1 |
|  | *Klebsiella pneumoniae* strain 9949 plasmid unnamed3, complete sequence | *Klebsiella pneumoniae* | 80395 | 2.47E+05 | 99% | 0 | 99.95 | 87872 | 114400 | CP050283.1 |
|  | *Klebsiella pneumoniae* strain KP18-3-8 plasmid pKP18-3-8-IncFII, complete sequence | *Klebsiella pneumoniae* | 80328 | 1.84E+05 | 99% | 0 | 100 | 87872 | 87095 | MT035876.1 |
|  | *Klebsiella pneumoniae* strain KP58 plasmid pKP58-3, complete sequence | *Klebsiella pneumoniae* | 80328 | 1.84E+05 | 99% | 0 | 100 | 87872 | 87095 | CP041376.1 |
|  | *Klebsiella pneumoniae* *subsp.* pneumoniae strain DD02391 plasmid pDD02391-3, complete sequence | *Klebsiella pneumoniae* *subsp.* pneumoniae | 80328 | 1.84E+05 | 99% | 0 | 100 | 87872 | 87095 | CP087642.1 |
|  | *Klebsiella pneumoniae* strain 21080237 plasmid p21080237_3, complete sequence | *Klebsiella pneumoniae* | 80328 | 1.84E+05 | 99% | 0 | 100 | 87872 | 87095 | CP095243.1 |
|  | *Klebsiella pneumoniae* strain KP697 plasmid unnamed3, complete sequence | *Klebsiella pneumoniae* | 80328 | 1.84E+05 | 99% | 0 | 100 | 87872 | 87095 | CP066154.1 |
|  | *Klebsiella pneumoniae* strain 37 plasmid pKP37_4, complete sequence | *Klebsiella pneumoniae* | 80328 | 1.84E+05 | 99% | 0 | 100 | 87872 | 87095 | CP082756.1 |
|  | *Klebsiella pneumoniae* strain 36 plasmid pKP36_4, complete sequence | *Klebsiella pneumoniae* | 80328 | 1.84E+05 | 99% | 0 | 100 | 87872 | 87095 | CP082762.1 |
|  | *Klebsiella pneumoniae* strain GZ-1 plasmid unnamed3, complete sequence | *Klebsiella pneumoniae* | 80328 | 1.84E+05 | 99% | 0 | 100 | 87872 | 87094 | CP031937.1 |
|  | *Klebsiella pneumoniae* strain CRKP66R plasmid pCRKP66R-4-tetA, complete sequence | *Klebsiella pneumoniae* | 80322 | 1.84E+05 | 99% | 0 | 100 | 87872 | 87095 | CP063836.1 |
|  | *Klebsiella pneumoniae* strain KP20194c4 plasmid pKP20194c4-p3, complete sequence | *Klebsiella pneumoniae* | 80317 | 1.84E+05 | 99% | 0 | 100 | 87872 | 87095 | CP054747.1 |
|  | *Klebsiella pneumoniae* strain KP20194d plasmid pKP20194d-p3, complete sequence | *Klebsiella pneumoniae* | 80317 | 1.84E+05 | 99% | 0 | 100 | 87872 | 87095 | CP054735.1 |
|  | *Klebsiella pneumoniae* strain KP20194c plasmid pKP20194c-p3, complete sequence | *Klebsiella pneumoniae* | 80317 | 1.84E+05 | 99% | 0 | 100 | 87872 | 87095 | CP054759.1 |
|  | *Klebsiella pneumoniae* strain KPK3 plasmid pKPK3-3, complete sequence | *Klebsiella pneumoniae* | 80280 | 1.84E+05 | 99% | 0 | 99.98 | 87872 | 87091 | CP090346.1 |
|  | *Klebsiella pneumoniae* strain 9630 plasmid unnamed3, complete sequence | *Klebsiella pneumoniae* | 80265 | 1.64E+05 | 83% | 0 | 99.91 | 87872 | 93415 | CP050288.1 |
|  | *Klebsiella pneumoniae* strain 10553 plasmid unnamed4, complete sequence | *Klebsiella pneumoniae* | 79977 | 1.70E+05 | 83% | 0 | 99.87 | 87872 | 96437 | CP050279.1 |
|  | Klebsiella michiganensis strain KO_408 plasmid pKO_2, complete sequence | Klebsiella michiganensis | 76374 | 1.17E+05 | 67% | 0 | 99.99 | 87872 | 62120 | CP091472.1 |
|  | *Klebsiella pneumoniae* strain 205880 plasmid p205880-qnrS | *Klebsiella pneumoniae* | 76358 | 1.27E+05 | 73% | 0 | 99.98 | 87872 | 65112 | MF190368.1 |
|  | *Klebsiella pneumoniae* strain JX-CR-hvKP-3 plasmid pJX3-3, complete sequence | *Klebsiella pneumoniae* | 74775 | 1.84E+05 | 99% | 0 | 100 | 87872 | 87095 | CP064244.1 |
|  | *Klebsiella pneumoniae* strain Kp36 plasmid unnamed3, complete sequence | *Klebsiella pneumoniae* | 73422 | 1.84E+05 | 99% | 0 | 99.95 | 87872 | 87117 | CP047195.1 |
|  | *Klebsiella pneumoniae* strain KPC-2 plasmid pKP169-P3, complete sequence | *Klebsiella pneumoniae* | 73335 | 1.42E+05 | 79% | 0 | 99.99 | 87872 | 95399 | CP078125.1 |
|  | Klebsiella aerogenes strain E20 plasmid pE20-qnrS, complete sequence | Klebsiella aerogenes | 73320 | 1.40E+05 | 75% | 0 | 99.98 | 87872 | 106790 | MG288684.1 |
|  | *Klebsiella pneumoniae* strain KP46 plasmid pKP46_1_KPC, complete sequence | *Klebsiella pneumoniae* | 73313 | 1.14E+05 | 65% | 0 | 99.98 | 87872 | 103807 | CP090127.1 |
|  | Enterobacter hormaechei strain cre46 plasmid unnamed3, complete sequence | Enterobacter hormaechei | 73294 | 1.64E+05 | 70% | 0 | 99.97 | 87872 | 95623 | CP039387.1 |
|  | *Klebsiella pneumoniae* isolate Kp_Goe_154414 plasmid pKp_Goe_414-4, complete sequence | *Klebsiella pneumoniae* | 70676 | 1.69E+05 | 93% | 0 | 99.97 | 87872 | 81641 | CP018341.1 |
|  | *Klebsiella pneumoniae* strain KP120 plasmid pKP120-92Kb, complete sequence | *Klebsiella pneumoniae* | 70637 | 2.00E+05 | 93% | 0 | 100 | 87872 | 92237 | CP060745.1 |
|  | *Klebsiella pneumoniae* E328 plasmid pE328_IMP6 DNA, complete sequence | *Klebsiella pneumoniae* | 69588 | 1.40E+05 | 86% | 0 | 99.99 | 87872 | 96042 | AP022370.1 |
|  | Klebsiella variicola strain X39 plasmid pX39-8, complete sequence | Klebsiella variicola | 69462 | 1.35E+05 | 75% | 0 | 99.97 | 87872 | 72224 | CP023985.1 |
|  | Shigella flexneri strain STEFF_12 plasmid unnamed2, complete sequence | Shigella flexneri | 69235 | 1.47E+05 | 86% | 0 | 100 | 87872 | 82001 | CP055221.1 |
|  | *Klebsiella pneumoniae* strain TH164 plasmid pTH164-3, complete sequence | *Klebsiella pneumoniae* | 68197 | 1.20E+05 | 72% | 0 | 99.95 | 87872 | 73685 | CP035213.1 |
|  | *Klebsiella pneumoniae* *subsp.* pneumoniae strain 90CM2 plasmid p90CM2-64k, complete sequence | *Klebsiella pneumoniae* *subsp.* pneumoniae | 68070 | 1.14E+05 | 69% | 0 | 99.99 | 87872 | 64683 | CP071824.1 |
|  | *Klebsiella pneumoniae* strain FDAARGOS_1330 plasmid unnamed1, complete sequence | *Klebsiella pneumoniae* | 68048 | 1.22E+05 | 75% | 0 | 99.98 | 87872 | 67308 | CP070178.1 |
|  | *Escherichia coli* strain E-T654 plasmid pE-T602-KPC-2, complete sequence | *Escherichia coli* | 68046 | 1.27E+05 | 75% | 0 | 99.98 | 87872 | 73083 | CP090290.1 |
|  | *Klebsiella pneumoniae* strain LH94 plasmid pLH94-3, complete sequence | *Klebsiella pneumoniae* | 68044 | 1.02E+05 | 61% | 0 | 99.98 | 87872 | 58460 | CP035205.1 |
|  | Klebsiella quasipneumoniae strain TH114 plasmid pTH114-3, complete sequence | Klebsiella quasipneumoniae | 67965 | 1.04E+05 | 63% | 0 | 99.95 | 87872 | 59814 | CP035208.1 |
|  | *Klebsiella pneumoniae* strain 57 plasmid pKP57-4, complete sequence | *Klebsiella pneumoniae* | 67762 | 1.26E+05 | 73% | 0 | 99.98 | 87872 | 72637 | CP088128.1 |
|  | *Klebsiella pneumoniae* strain MYKLB95 plasmid MYKLB95-1, complete sequence | *Klebsiella pneumoniae* | 67274 | 1.35E+05 | 74% | 0 | 99.98 | 87872 | 99493 | MH341574.1 |
|  | *Klebsiella pneumoniae* *subsp.* pneumoniae strain SH2 plasmid pSH2-KPC, complete sequence | *Klebsiella pneumoniae* *subsp.* pneumoniae | 67267 | 1.92E+05 | 97% | 0 | 99.99 | 87872 | 302845 | MH643791.1 |
|  | *Klebsiella pneumoniae* strain 50700 plasmid p50700-83.4, complete sequence | *Klebsiella pneumoniae* | 67189 | 1.72E+05 | 96% | 0 | 99.99 | 87872 | 83442 | CP088994.1 |
|  | *Klebsiella pneumoniae* strain ZY393 plasmid pZY1393-3, complete sequence | *Klebsiella pneumoniae* | 64353 | 1.75E+05 | 97% | 0 | 100 | 87872 | 84768 | CP076674.1 |
|  | *Klebsiella pneumoniae* strain 21091025 plasmid p21091025_2, complete sequence | *Klebsiella pneumoniae* | 64353 | 1.75E+05 | 97% | 0 | 100 | 87872 | 84876 | CP095266.1 |
|  | *Klebsiella pneumoniae* strain QS17-0161 plasmid pMR0617tem, complete sequence | *Klebsiella pneumoniae* | 62092 | 1.38E+05 | 81% | 0 | 99.98 | 87872 | 77269 | CP024461.1 |
|  | Klebsiella grimontii strain 2481359 plasmid p2481359-2, complete sequence | Klebsiella grimontii | 60953 | 1.45E+05 | 81% | 0 | 100 | 87872 | 75780 | CP067382.1 |
|  | Klebsiella aerogenes strain KAE3SP plasmid pKAE3SP-1, complete sequence | Klebsiella aerogenes | 60429 | 1.69E+05 | 93% | 0 | 100 | 87872 | 81641 | CP082899.1 |
|  | *Klebsiella pneumoniae* strain KPN43G plasmid pKPN43G-qnrS, complete sequence | *Klebsiella pneumoniae* | 60395 | 1.17E+05 | 67% | 0 | 99.98 | 87872 | 62120 | CP082210.1 |
|  | Klebsiella quasipneumoniae strain L22 plasmid pL22-4, complete sequence | Klebsiella quasipneumoniae | 59610 | 1.27E+05 | 74% | 0 | 99.94 | 87872 | 66154 | CP031261.1 |
|  | *Klebsiella pneumoniae* strain kp5152 plasmid p3, complete sequence | *Klebsiella pneumoniae* | 58046 | 1.73E+05 | 96% | 0 | 100 | 87872 | 83551 | CP090465.1 |
|  | *Klebsiella pneumoniae* *subsp.* pneumoniae strain KPCTRSRTH01 plasmid unnamed3, complete sequence | *Klebsiella pneumoniae* *subsp.* pneumoniae | 56889 | 1.23E+05 | 70% | 0 | 99.97 | 87872 | 70501 | CP041095.1 |
|  | *Klebsiella pneumoniae* strain CDI694 plasmid pCDI694-87.0, complete sequence | *Klebsiella pneumoniae* | 56392 | 1.83E+05 | 99% | 0 | 100 | 87872 | 86980 | CP077776.1 |
|  | *Klebsiella pneumoniae* strain F726925 plasmid pF726925-2, complete sequence | *Klebsiella pneumoniae* | 56034 | 1.71E+05 | 94% | 0 | 100 | 87872 | 82468 | CP081822.1 |
|  | *Klebsiella pneumoniae* isolate 991178e0-b809-11e8-aae5-3c4a9275d6c8 genome assembly, chromosome: 1 | *Klebsiella pneumoniae* | 55023 | 1.47E+05 | 80% | 0 | 99.98 | 87872 | 5570095 | LR596809.1 |
|  | *Klebsiella pneumoniae* strain XHKP502 plasmid pXHKP502-3, complete sequence | *Klebsiella pneumoniae* | 54735 | 1.80E+05 | 96% | 0 | 100 | 87872 | 85185 | CP066907.1 |
|  | *Klebsiella pneumoniae* strain JX-CR-hvKP-9 plasmid pJX9-4, complete sequence | *Klebsiella pneumoniae* | 54735 | 1.35E+05 | 83% | 0 | 100 | 87872 | 77551 | CP064215.1 |
|  | *Klebsiella pneumoniae* strain JX-CR-hvKP-8 plasmid pJX8-2, complete sequence | *Klebsiella pneumoniae* | 54702 | 2.13E+05 | 100% | 0 | 100 | 87872 | 146760 | CP064219.1 |
|  | *Klebsiella pneumoniae* strain JX-CR-hvKP-7 plasmid pJX7-2, complete sequence | *Klebsiella pneumoniae* | 53022 | 1.86E+05 | 94% | 0 | 100 | 87872 | 133575 | CP064225.1 |
|  | *Klebsiella pneumoniae* strain CRKP52R plasmid pCRKP52R-4-tetA, complete sequence | *Klebsiella pneumoniae* | 49498 | 1.87E+05 | 99% | 0 | 99.99 | 87872 | 99066 | CP066252.1 |
|  | *Klebsiella pneumoniae* strain KP-C76 plasmid pC76-tetA, complete sequence | *Klebsiella pneumoniae* | 48942 | 1.72E+05 | 91% | 0 | 100 | 87872 | 80673 | CP080300.1 |
|  | *Klebsiella pneumoniae* isolate 11 genome assembly, plasmid: P3 | *Klebsiella pneumoniae* | 48564 | 67523 | 37% | 0 | 99.95 | 87872 | 50791 | OW969941.1 |
|  | Raoultella ornithinolytica plasmid pWP8-W19-CRE-01_3 DNA, complete genome, strain: WP8-W19-CRE-01 | Raoultella ornithinolytica | 48551 | 1.74E+05 | 93% | 0 | 99.99 | 87872 | 86933 | AP022271.1 |
|  | Klebsiella aerogenes strain NY1688 plasmid pNY1688-6, complete sequence | Klebsiella aerogenes | 48366 | 1.17E+05 | 67% | 0 | 99.98 | 87872 | 62120 | CP094278.1 |
|  | *Klebsiella pneumoniae* strain FK 6768 plasmid unnamed2, complete sequence | *Klebsiella pneumoniae* | 48200 | 1.61E+05 | 89% | 0 | 99.69 | 87872 | 77979 | CP065556.1 |
|  | *Klebsiella pneumoniae* isolate 975dbbbc-b809-11e8-aae5-3c4a9275d6c8 genome assembly, chromosome: 1 | *Klebsiella pneumoniae* | 47936 | 1.86E+05 | 96% | 0 | 100 | 87872 | 5634920 | LR596814.1 |
|  | *Klebsiella pneumoniae* isolate 97706988-b809-11e8-aae5-3c4a9275d6c8 genome assembly, chromosome: 1 | *Klebsiella pneumoniae* | 47936 | 1.86E+05 | 96% | 0 | 100 | 87872 | 5652578 | LR596813.1 |
| pCR-hvKP173-P4 | *Klebsiella pneumoniae* strain 36 plasmid pKP36_5, complete sequence | *Klebsiella pneumoniae* | 22083 | 22083 | 99% | 0 | 100 | 11970 | 11970 | CP082763.1 |
|  | *Klebsiella pneumoniae* strain CRKP66R plasmid pCRKP66R-5, complete sequence | *Klebsiella pneumoniae* | 22077 | 22077 | 99% | 0 | 100 | 11970 | 11970 | CP063837.1 |
|  | *Klebsiella pneumoniae* strain KP20194c plasmid pKP20194c-p4, complete sequence | *Klebsiella pneumoniae* | 22075 | 22075 | 99% | 0 | 99.99 | 11970 | 11970 | CP054760.1 |
|  | *Klebsiella pneumoniae* strain KP20194a plasmid pKP20194a-p4, complete sequence | *Klebsiella pneumoniae* | 22074 | 22074 | 99% | 0 | 99.99 | 11970 | 11970 | CP054784.1 |
|  | *Klebsiella pneumoniae* strain C2414 plasmid pC2414-4, complete sequence | *Klebsiella pneumoniae* | 22072 | 22072 | 99% | 0 | 100 | 11970 | 11970 | CP039822.1 |
|  | *Klebsiella pneumoniae* *subsp.* pneumoniae strain DD01635 plasmid pDD01635-4, complete sequence | *Klebsiella pneumoniae* *subsp.* pneumoniae | 22064 | 22064 | 99% | 0 | 100 | 11970 | 11970 | CP087661.1 |
|  | *Klebsiella pneumoniae* strain C2660 plasmid pC2660-5, complete sequence | *Klebsiella pneumoniae* | 22061 | 22061 | 99% | 0 | 99.99 | 11970 | 11970 | CP039812.1 |
|  | *Klebsiella pneumoniae* strain 21080937 plasmid p21080937_6, complete sequence | *Klebsiella pneumoniae* | 22061 | 22061 | 99% | 0 | 100 | 11970 | 11970 | CP095259.1 |
|  | *Klebsiella pneumoniae* *subsp.* pneumoniae strain DD01845 plasmid pDD01845-4, complete sequence | *Klebsiella pneumoniae* *subsp.* pneumoniae | 22053 | 22053 | 99% | 0 | 99.99 | 11970 | 11970 | CP087667.1 |
|  | *Klebsiella pneumoniae* strain 8695 plasmid p4, complete sequence | *Klebsiella pneumoniae* | 22044 | 44121 | 100% | 0 | 99.89 | 11970 | 23957 | CP085893.1 |
|  | *Klebsiella pneumoniae* strain CDI694 plasmid pCDI694-12.0, complete sequence | *Klebsiella pneumoniae* | 21861 | 21861 | 99% | 0 | 99.67 | 11970 | 11972 | CP077775.1 |
|  | *Klebsiella pneumoniae* strain 21091025 plasmid p21091025_3, complete sequence | *Klebsiella pneumoniae* | 21608 | 43319 | 100% | 0 | 99.27 | 11970 | 23759 | CP095267.1 |
|  | *Escherichia coli* strain CR-HvKP5TC-5 plasmid pCR-HvKP5TC-5_p4, complete sequence | *Escherichia coli* | 20753 | 22106 | 100% | 0 | 100 | 11970 | 11970 | OM001479.1 |
|  | *Klebsiella pneumoniae* strain KPWX136 plasmid pE, complete sequence | *Klebsiella pneumoniae* | 20751 | 22101 | 100% | 0 | 99.99 | 11970 | 11970 | CP069174.1 |
|  | *Klebsiella pneumoniae* strain Kp36 plasmid unnamed4, complete sequence | *Klebsiella pneumoniae* | 19908 | 22074 | 100% | 0 | 99.86 | 11970 | 12001 | CP047196.1 |
|  | *Klebsiella pneumoniae* strain KP20194f plasmid pKP20194f-p4, complete sequence | *Klebsiella pneumoniae* | 19645 | 22101 | 100% | 0 | 99.99 | 11970 | 11970 | CP054724.1 |
|  | *Klebsiella pneumoniae* *subsp.* pneumoniae strain DD01653 plasmid pDD01653-4, complete sequence | *Klebsiella pneumoniae* *subsp.* pneumoniae | 19642 | 22095 | 100% | 0 | 99.98 | 11970 | 11970 | CP087655.1 |
|  | *Klebsiella pneumoniae* strain KP58 plasmid pKP58-4, complete sequence | *Klebsiella pneumoniae* | 19396 | 22101 | 100% | 0 | 99.99 | 11970 | 11970 | CP041377.1 |
|  | *Klebsiella pneumoniae* *subsp.* pneumoniae strain DD02391 plasmid pDD02391-4, complete sequence | *Klebsiella pneumoniae* *subsp.* pneumoniae | 19014 | 21724 | 100% | 0 | 99.36 | 11970 | 11959 | CP087643.1 |
|  | *Klebsiella pneumoniae* strain XHKP502 plasmid pXHKP502-4, complete sequence | *Klebsiella pneumoniae* | 18766 | 22095 | 100% | 0 | 99.98 | 11970 | 11971 | CP066908.1 |
|  | *Klebsiella pneumoniae* strain WCHKP090361 plasmid p2_090361, complete sequence | *Klebsiella pneumoniae* | 18349 | 22106 | 100% | 0 | 100 | 11970 | 11970 | CP066532.1 |
|  | *Klebsiella pneumoniae* strain KP18-2079 plasmid pKP18-2079_11kb, complete sequence | *Klebsiella pneumoniae* | 18329 | 22101 | 100% | 0 | 99.99 | 11970 | 11970 | MT090962.1 |
|  | *Klebsiella pneumoniae* strain 9949 plasmid unnamed4, complete sequence | *Klebsiella pneumoniae* | 18144 | 29934 | 100% | 0 | 99.97 | 11970 | 16223 | CP050284.1 |
|  | *Klebsiella pneumoniae* strain 37 plasmid pKP37_5, complete sequence | *Klebsiella pneumoniae* | 18066 | 22093 | 100% | 0 | 99.98 | 11970 | 11969 | CP082757.1 |
|  | *Klebsiella pneumoniae* strain FK 6768 plasmid unnamed4, complete sequence | *Klebsiella pneumoniae* | 17516 | 21957 | 100% | 0 | 99.68 | 11970 | 12001 | CP065558.1 |
|  | *Klebsiella pneumoniae* strain KP1064WHY plasmid p4, complete sequence | *Klebsiella pneumoniae* | 17256 | 22008 | 100% | 0 | 99.82 | 11970 | 11972 | CP084709.1 |
|  | *Escherichia coli* strain CR-HvKP3TC plasmid pCR-HvKP3TC_p4, complete sequence | *Escherichia coli* | 16982 | 22106 | 100% | 0 | 100 | 11970 | 11970 | MW598243.1 |
|  | *Klebsiella pneumoniae* strain CR-HvKP3 plasmid p4-CR-HvKP3, complete sequence | *Klebsiella pneumoniae* | 16982 | 22106 | 100% | 0 | 100 | 11970 | 11970 | MW598236.1 |
|  | *Klebsiella pneumoniae* strain KP697 plasmid unnamed4, complete sequence | *Klebsiella pneumoniae* | 16973 | 22101 | 100% | 0 | 99.99 | 11970 | 11970 | CP066155.1 |
|  | *Klebsiella pneumoniae* strain KP18-2113 plasmid pKP18-2113-3, complete sequence | *Klebsiella pneumoniae* | 16957 | 22088 | 100% | 0 | 99.97 | 11970 | 11969 | CP082028.1 |
|  | *Klebsiella pneumoniae* strain KP20194c4 plasmid pKP20194c4-p4, complete sequence | *Klebsiella pneumoniae* | 16842 | 21979 | 100% | 0 | 99.75 | 11970 | 11971 | CP054748.1 |
|  | *Klebsiella pneumoniae* strain KP20194b2 plasmid pKP20194b2-p4, complete sequence | *Klebsiella pneumoniae* | 16809 | 22101 | 100% | 0 | 99.99 | 11970 | 11970 | CP054766.1 |
|  | *Klebsiella pneumoniae* strain KP200731214 plasmid pKP-5 | *Klebsiella pneumoniae* | 16720 | 22106 | 100% | 0 | 100 | 11970 | 11970 | CP084748.1 |
|  | *Klebsiella pneumoniae* strain KP18-238 plasmid pKP18-238-3, complete sequence | *Klebsiella pneumoniae* | 16687 | 21724 | 100% | 0 | 99.28 | 11970 | 11959 | CP082013.1 |
|  | *Klebsiella pneumoniae* strain 12 plasmid pKP12_5, complete sequence | *Klebsiella pneumoniae* | 15653 | 22084 | 100% | 0 | 100 | 11970 | 11970 | CP082769.1 |
|  | *Escherichia coli* strain CR-HvKP4TC plasmid pCR-HvKP4TC_Vir-p4, complete sequence | *Escherichia coli* | 15034 | 25688 | 100% | 0 | 100 | 11970 | 190130 | MW598245.1 |
|  | *Escherichia coli* strain CR-HvKP4TC-2 plasmid pCR-HvKP4TC-2_p4, complete sequence | *Escherichia coli* | 14994 | 22106 | 100% | 0 | 100 | 11970 | 11970 | OM001477.1 |
|  | *Klebsiella pneumoniae* strain KP1517 plasmid unnamed3, complete sequence | *Klebsiella pneumoniae* | 14922 | 22106 | 100% | 0 | 100 | 11970 | 11970 | CP072466.1 |
|  | *Klebsiella pneumoniae* strain LZKP00003 plasmid pZR1, complete sequence | *Klebsiella pneumoniae* | 14698 | 22106 | 100% | 0 | 100 | 11970 | 11970 | CP089992.1 |
|  | *Klebsiella pneumoniae* strain FDAARGOS_443 plasmid unnamed4, complete sequence | *Klebsiella pneumoniae* | 14683 | 22097 | 100% | 0 | 99.97 | 11970 | 11972 | CP023932.1 |
|  | *Klebsiella pneumoniae* strain JX-CR-hvKP-3 plasmid pJX3-4, complete sequence | *Klebsiella pneumoniae* | 14534 | 22106 | 100% | 0 | 100 | 11970 | 11970 | CP064245.1 |
|  | *Klebsiella pneumoniae* strain KP20194a2 plasmid pKP20194a2-p4, complete sequence | *Klebsiella pneumoniae* | 14314 | 22046 | 100% | 0 | 99.83 | 11970 | 15931 | CP054778.1 |
|  | *Klebsiella pneumoniae* strain KP55 plasmid pKP55_5, complete sequence | *Klebsiella pneumoniae* | 14299 | 22106 | 100% | 0 | 100 | 11970 | 11970 | CP055299.1 |
|  | *Klebsiella pneumoniae* strain L482 plasmid p5_L382, complete sequence | *Klebsiella pneumoniae* | 14296 | 22106 | 100% | 0 | 100 | 11970 | 11970 | CP033964.1 |
|  | *Klebsiella pneumoniae* strain SCKP020009 plasmid p1_020009, complete sequence | *Klebsiella pneumoniae* | 14283 | 22106 | 100% | 0 | 100 | 11970 | 11970 | CP038005.1 |
|  | *Klebsiella pneumoniae* strain L39_2 plasmid p5_L39, complete sequence | *Klebsiella pneumoniae* | 14277 | 22101 | 100% | 0 | 99.99 | 11970 | 11970 | CP033958.1 |
|  | *Klebsiella pneumoniae* strain KP16 plasmid unnamed4, complete sequence | *Klebsiella pneumoniae* | 14277 | 22101 | 100% | 0 | 99.99 | 11970 | 11970 | CP087150.1 |
|  | *Klebsiella pneumoniae* strain KP14 plasmid unnamed4, complete sequence | *Klebsiella pneumoniae* | 14277 | 22101 | 100% | 0 | 99.99 | 11970 | 11970 | CP087155.1 |
|  | *Klebsiella pneumoniae* strain WSCRKP plasmid pWSCRKP-4, complete sequence | *Klebsiella pneumoniae* | 14235 | 22115 | 100% | 0 | 99.99 | 11970 | 15931 | CP091072.1 |
|  | *Klebsiella pneumoniae* strain KP15 plasmid unnamed3, complete sequence | *Klebsiella pneumoniae* | 14044 | 25657 | 100% | 0 | 99.97 | 11970 | 99065 | CP087145.1 |
|  | *Klebsiella pneumoniae* strain hvKP323 plasmid unnamed3, complete sequence | *Klebsiella pneumoniae* | 14039 | 22106 | 100% | 0 | 100 | 11970 | 11970 | CP101773.1 |
|  | *Klebsiella pneumoniae* strain XH1507 plasmid pXH1507-5, complete sequence | *Klebsiella pneumoniae* | 14039 | 22106 | 100% | 0 | 100 | 11970 | 11970 | CP092798.1 |
|  | *Klebsiella pneumoniae* strain CRKP52R plasmid pCRKP52R-4-tetA, complete sequence | *Klebsiella pneumoniae* | 14028 | 25641 | 100% | 0 | 99.93 | 11970 | 99066 | CP066252.1 |
|  | *Escherichia coli* strain CR-HvKP5TC plasmid pCR-HvKP5TC_Vir-p4, complete sequence | *Escherichia coli* | 14026 | 25646 | 100% | 0 | 99.93 | 11970 | 190129 | MW598247.1 |
|  | *Escherichia coli* strain CR-HvKP1TC plasmid pCR-HvKP1TC_Vir-p4, complete sequence | *Escherichia coli* | 14026 | 25646 | 100% | 0 | 99.93 | 11970 | 190129 | MW598240.1 |
|  | *Klebsiella pneumoniae* strain BSI058 plasmid pBSI058-KPC2 | *Klebsiella pneumoniae* | 14024 | 25644 | 100% | 0 | 99.91 | 11970 | 114712 | MT269836.1 |
|  | *Klebsiella pneumoniae* strain 1864 plasmid p1864-4, complete sequence | *Klebsiella pneumoniae* | 14020 | 22106 | 100% | 0 | 100 | 11970 | 11970 | CP084496.1 |
|  | *Klebsiella pneumoniae* strain KP-CT77 plasmid pCT77-tetA, complete sequence | *Klebsiella pneumoniae* | 14007 | 25620 | 100% | 0 | 99.88 | 11970 | 92645 | CP080306.1 |
|  | *Klebsiella pneumoniae* strain WCHKP115068 plasmid p2_115068, complete sequence | *Klebsiella pneumoniae* | 13825 | 22121 | 100% | 0 | 100 | 11970 | 12746 | CP036369.1 |
|  | *Klebsiella pneumoniae* strain WCHKP020098 plasmid p2_020098, complete sequence | *Klebsiella pneumoniae* | 13444 | 22106 | 100% | 0 | 100 | 11970 | 11970 | CP036308.1 |
|  | *Klebsiella pneumoniae* strain JX-CR-hvKP-1 plasmid pJX1-4, complete sequence | *Klebsiella pneumoniae* | 13345 | 22106 | 100% | 0 | 100 | 11970 | 11970 | CP064256.1 |
|  | *Klebsiella pneumoniae* JM45 plasmid p2, complete sequence | *Klebsiella pneumoniae* JM45 | 13274 | 16372 | 80% | 0 | 97.22 | 11970 | 12207 | CP006658.1 |
|  | *Klebsiella pneumoniae* isolate 307 genome assembly, plasmid: P3 | *Klebsiella pneumoniae* | 12912 | 19526 | 80% | 0 | 97.31 | 11970 | 34959 | OW967853.1 |
|  | *Klebsiella pneumoniae* isolate 307 genome assembly, plasmid: P3 | *Klebsiella pneumoniae* | 12912 | 19526 | 80% | 0 | 97.31 | 11970 | 34959 | OW967206.1 |
|  | *Klebsiella pneumoniae* isolate 307 genome assembly, plasmid: P3 | *Klebsiella pneumoniae* | 12912 | 19526 | 80% | 0 | 97.31 | 11970 | 34959 | OW849062.1 |
|  | *Klebsiella pneumoniae* strain RIVM_C019006 plasmid pRIVM_C019006_3, complete sequence | *Klebsiella pneumoniae* | 12912 | 19522 | 80% | 0 | 97.3 | 11970 | 34959 | CP068923.1 |
|  | *Klebsiella pneumoniae* strain WCHKP090329 plasmid p2_090329, complete sequence | *Klebsiella pneumoniae* | 12912 | 22106 | 100% | 0 | 100 | 11970 | 11970 | CP066521.1 |
|  | *Klebsiella pneumoniae* strain DD521 plasmid pDD521.4, complete sequence | *Klebsiella pneumoniae* | 12896 | 22088 | 100% | 0 | 99.97 | 11970 | 11969 | CP075320.1 |
|  | *Klebsiella pneumoniae* strain IR12243_1 plasmid unnamed1, complete sequence | *Klebsiella pneumoniae* | 12700 | 22124 | 100% | 0 | 99.99 | 11970 | 12798 | CP097661.1 |
|  | *Klebsiella pneumoniae* strain K64 plasmid pColRNAI-5, complete sequence | *Klebsiella pneumoniae* | 12626 | 20693 | 99% | 0 | 96.8 | 11970 | 11934 | CP102395.1 |
|  | *Klebsiella pneumoniae* strain S234 plasmid pS234-3, complete sequence | *Klebsiella pneumoniae* | 12183 | 22101 | 100% | 0 | 99.98 | 11970 | 11970 | CP102189.1 |
|  | *Klebsiella pneumoniae* strain L491 plasmid p4-L491 | *Klebsiella pneumoniae* | 11875 | 22106 | 100% | 0 | 100 | 11970 | 11970 | CP029229.1 |
|  | *Klebsiella pneumoniae* strain L388 plasmid p5-L388 | *Klebsiella pneumoniae* | 11869 | 22101 | 100% | 0 | 99.98 | 11970 | 11970 | CP029224.1 |
|  | *Klebsiella pneumoniae* *subsp.* pneumoniae strain KPN857 plasmid pD, complete sequence | *Klebsiella pneumoniae* *subsp.* pneumoniae | 11854 | 22101 | 100% | 0 | 99.98 | 11970 | 11970 | CP090436.1 |
|  | *Klebsiella pneumoniae* strain JX-CR-hvKP-4 plasmid pJX4-4, complete sequence | *Klebsiella pneumoniae* | 11701 | 22106 | 100% | 0 | 100 | 11970 | 11970 | CP064239.1 |
|  | *Escherichia coli* strain CR-HvKP1TC-3 plasmid pCR-HvKP1TC-3_p4, complete sequence | *Escherichia coli* | 11620 | 22106 | 100% | 0 | 100 | 11970 | 11970 | OM001473.1 |
|  | *Klebsiella pneumoniae* strain WCHKP36 plasmid p2_020036, complete sequence | *Klebsiella pneumoniae* | 11616 | 22122 | 100% | 0 | 100 | 11970 | 12746 | CP028580.2 |
|  | *Klebsiella pneumoniae* strain hvKP340 plasmid unnamed5, complete sequence | *Klebsiella pneumoniae* | 11616 | 22106 | 100% | 0 | 100 | 11970 | 11970 | CP101781.1 |
|  | *Klebsiella pneumoniae* strain hvKP841 plasmid unnamed3, complete sequence | *Klebsiella pneumoniae* | 11616 | 22106 | 100% | 0 | 100 | 11970 | 11970 | CP101787.1 |
|  | *Klebsiella pneumoniae* strain 19PDR22 plasmid p7, complete sequence | *Klebsiella pneumoniae* | 11616 | 22106 | 100% | 0 | 100 | 11970 | 11970 | CP076551.1 |
|  | *Klebsiella pneumoniae* strain S270v plasmid pS270V-3, complete sequence | *Klebsiella pneumoniae* | 11611 | 22101 | 100% | 0 | 99.98 | 11970 | 11970 | CP102195.1 |
|  | *Klebsiella pneumoniae* strain hvKP319 plasmid unnamed4, complete sequence | *Klebsiella pneumoniae* | 11611 | 22101 | 100% | 0 | 99.98 | 11970 | 11970 | CP101768.1 |
|  | *Klebsiella pneumoniae* strain FRPDR plasmid pFRPDR_5, complete sequence | *Klebsiella pneumoniae* | 11611 | 22101 | 100% | 0 | 99.98 | 11970 | 11970 | CP063764.1 |
|  | *Klebsiella pneumoniae* strain CR-HvKP4 plasmid pCR-HvKP4-p4, complete sequence | *Klebsiella pneumoniae* | 11548 | 22106 | 100% | 0 | 100 | 11970 | 11970 | CP040543.1 |
|  | *Klebsiella pneumoniae* strain CR-HvKP5 plasmid pCR-HvKP5-p4, complete sequence | *Klebsiella pneumoniae* | 11527 | 22106 | 100% | 0 | 100 | 11970 | 11970 | CP040549.1 |
|  | *Klebsiella pneumoniae* strain CR-HvKP1 plasmid pCR-HvKP1-p4, complete sequence | *Klebsiella pneumoniae* | 11514 | 22106 | 100% | 0 | 100 | 11970 | 11970 | CP040537.1 |
|  | *Klebsiella pneumoniae* strain 50700 plasmid p50700-12.0, complete sequence | *Klebsiella pneumoniae* | 11420 | 21920 | 100% | 0 | 99.46 | 11970 | 11972 | CP088993.1 |
|  | *Klebsiella pneumoniae* isolate 991178e0-b809-11e8-aae5-3c4a9275d6c8 genome assembly, chromosome: 1 | *Klebsiella pneumoniae* | 10707 | 24599 | 98% | 0 | 100 | 11970 | 5570095 | LR596809.1 |
|  | *Klebsiella pneumoniae* isolate 98fb0f42-b809-11e8-aae5-3c4a9275d6c8 genome assembly, chromosome: 1 | *Klebsiella pneumoniae* | 10707 | 25085 | 93% | 0 | 100 | 11970 | 5716474 | LR596807.1 |
|  | *Klebsiella pneumoniae* *subsp.* pneumoniae strain RJBSI76 plasmid pRJBSI76-4, complete sequence | *Klebsiella pneumoniae* *subsp.* pneumoniae | 10623 | 18843 | 85% | 0 | 99.98 | 11970 | 10206 | CP068693.1 |
|  | *Klebsiella pneumoniae* isolate 15 genome assembly, plasmid: P2 | *Klebsiella pneumoniae* | 10495 | 15990 | 80% | 0 | 96.82 | 11970 | 11955 | OW969695.1 |
|  | *Klebsiella pneumoniae* strain 150040X1B1 plasmid p1_150040X1B1, complete sequence | *Klebsiella pneumoniae* | 10490 | 18580 | 84% | 0 | 100 | 11970 | 10060 | CP101729.1 |
|  | *Klebsiella pneumoniae* *subsp.* pneumoniae strain KP29 plasmid p29_3, complete sequence | *Klebsiella pneumoniae* *subsp.* pneumoniae | 10490 | 18580 | 84% | 0 | 100 | 11970 | 10060 | CP101570.1 |
|  | *Klebsiella pneumoniae* strain FZKP4523 plasmid p1_FZKP4523, complete sequence | *Klebsiella pneumoniae* | 10490 | 18580 | 84% | 0 | 100 | 11970 | 10060 | CP101535.1 |
|  | *Klebsiella pneumoniae* strain XHKP75 plasmid pXHKP75-3, complete sequence | *Klebsiella pneumoniae* | 10490 | 18580 | 84% | 0 | 100 | 11970 | 10060 | CP066898.1 |
|  | *Klebsiella pneumoniae* strain XHKPN391 plasmid pXHKPN391-2, complete sequence | *Klebsiella pneumoniae* | 10490 | 18579 | 84% | 0 | 100 | 11970 | 10060 | CP066917.1 |
|  | *Klebsiella pneumoniae* strain HvKp-su1 plasmid unnamed2, complete sequence | *Klebsiella pneumoniae* | 10490 | 18582 | 84% | 0 | 100 | 11970 | 10061 | CP092719.1 |
|  | *Klebsiella pneumoniae* *subsp.* pneumoniae strain DD02280 plasmid pDD02280-3 | *Klebsiella pneumoniae* *subsp.* pneumoniae | 10490 | 18568 | 83% | 0 | 100 | 11970 | 91826 | CP087626.1 |
|  | *Klebsiella pneumoniae* *subsp.* pneumoniae strain DD02172 plasmid pDD02172-5, complete sequence | *Klebsiella pneumoniae* *subsp.* pneumoniae | 10490 | 18580 | 84% | 0 | 100 | 11970 | 10060 | CP087616.1 |
|  | *Klebsiella pneumoniae* *subsp.* pneumoniae strain WCHKP020039 plasmid p1_020039, complete sequence | *Klebsiella pneumoniae* *subsp.* pneumoniae | 10490 | 18580 | 84% | 0 | 100 | 11970 | 10060 | CP043346.1 |
| pCR-hvKP173-P5 | *Klebsiella pneumoniae* strain KP18-238 plasmid pKP18-238-4, complete sequence | *Klebsiella pneumoniae* | 10334 | 20671 | 100% | 0 | 100 | 5596 | 11192 | CP082017.1 |
|  | *Klebsiella pneumoniae* strain F44 plasmid p44-4, complete sequence | *Klebsiella pneumoniae* | 10198 | 10336 | 100% | 0 | 100 | 5596 | 5596 | CP025465.1 |
|  | *Klebsiella pneumoniae* *subsp.* pneumoniae strain SCKP020143 plasmid p4_020143, complete sequence | *Klebsiella pneumoniae* *subsp.* pneumoniae | 10178 | 10336 | 100% | 0 | 100 | 5596 | 5596 | CP028546.2 |
|  | *Klebsiella pneumoniae* *subsp.* pneumoniae strain WCHKP015093 plasmid p3_015093, complete sequence | *Klebsiella pneumoniae* *subsp.* pneumoniae | 10148 | 10336 | 100% | 0 | 100 | 5596 | 5596 | CP036304.1 |
|  | *Klebsiella pneumoniae* strain 135077 plasmid p3_135077, complete sequence | *Klebsiella pneumoniae* | 10144 | 10336 | 100% | 0 | 100 | 5596 | 5596 | CP073295.1 |
|  | *Klebsiella pneumoniae* *subsp.* pneumoniae strain DD02391 plasmid pDD02391-5, complete sequence | *Klebsiella pneumoniae* *subsp.* pneumoniae | 10135 | 10336 | 100% | 0 | 100 | 5596 | 5596 | CP087644.1 |
|  | *Klebsiella pneumoniae* strain K191663 plasmid unnamed1, complete sequence | *Klebsiella pneumoniae* | 10117 | 10336 | 100% | 0 | 100 | 5596 | 5596 | CP080354.1 |
|  | *Klebsiella pneumoniae* isolate 97706988-b809-11e8-aae5-3c4a9275d6c8 genome assembly, chromosome: 1 | *Klebsiella pneumoniae* | 10065 | 11066 | 100% | 0 | 100 | 5596 | 5652578 | LR596813.1 |
|  | *Klebsiella pneumoniae* strain JX-CR-hvKP-9 plasmid pJX9-5, complete sequence | *Klebsiella pneumoniae* | 10028 | 10336 | 100% | 0 | 100 | 5596 | 5596 | CP064216.1 |
|  | *Klebsiella pneumoniae* *subsp.* pneumoniae strain KP65 plasmid p65_3, complete sequence | *Klebsiella pneumoniae* *subsp.* pneumoniae | 10022 | 10330 | 100% | 0 | 99.98 | 5596 | 5596 | CP101566.1 |
|  | *Klebsiella pneumoniae* strain XHKP502 plasmid pXHKP502-5, complete sequence | *Klebsiella pneumoniae* | 10022 | 10330 | 100% | 0 | 99.98 | 5596 | 5596 | CP066909.1 |
|  | *Klebsiella pneumoniae* strain KP-C76 plasmid unnamed1, complete sequence | *Klebsiella pneumoniae* | 10019 | 10336 | 100% | 0 | 100 | 5596 | 5596 | CP080302.1 |
|  | *Klebsiella pneumoniae* strain KP69 plasmid p69-4, complete sequence | *Klebsiella pneumoniae* | 10013 | 10336 | 100% | 0 | 100 | 5596 | 5596 | CP025460.1 |
|  | *Klebsiella pneumoniae* strain 21072329 plasmid p21072329_5, complete sequence | *Klebsiella pneumoniae* | 10008 | 10336 | 100% | 0 | 100 | 5596 | 5596 | CP095239.1 |
|  | *Klebsiella pneumoniae* strain CR-HvKP4 plasmid pCR-HvKP4-p5, complete sequence | *Klebsiella pneumoniae* | 9932 | 10336 | 100% | 0 | 100 | 5596 | 5596 | CP040544.1 |
|  | *Klebsiella pneumoniae* isolate 392 genome assembly, plasmid: P4 | *Klebsiella pneumoniae* | 9895 | 10293 | 100% | 0 | 99.85 | 5596 | 5599 | OW849056.1 |
|  | *Klebsiella pneumoniae* *subsp.* pneumoniae strain DD02341 plasmid pDD02341-4, complete sequence | *Klebsiella pneumoniae* *subsp.* pneumoniae | 9878 | 10336 | 100% | 0 | 100 | 5596 | 5596 | CP087633.1 |
|  | *Klebsiella pneumoniae* strain XH1507 plasmid pXH1507-6, complete sequence | *Klebsiella pneumoniae* | 9878 | 10336 | 100% | 0 | 100 | 5596 | 5596 | CP092799.1 |
|  | Citrobacter koseri ATCC BAA-895 plasmid pCKO2, complete sequence | Citrobacter koseri ATCC BAA-895 | 9878 | 10190 | 100% | 0 | 99.5 | 5596 | 5601 | CP000824.1 |
|  | *Klebsiella pneumoniae* strain 160111 plasmid p5.6K_L111, complete sequence | *Klebsiella pneumoniae* | 9865 | 10336 | 100% | 0 | 100 | 5596 | 5596 | CP030131.1 |
|  | *Klebsiella pneumoniae* strain CR-HvKP5 plasmid pCR-HvKP5-p5, complete sequence | *Klebsiella pneumoniae* | 9823 | 10336 | 100% | 0 | 100 | 5596 | 5596 | CP040550.1 |
|  | *Klebsiella pneumoniae* strain KP18-2079 plasmid pKP18-2079_5kb, complete sequence | *Klebsiella pneumoniae* | 9793 | 10336 | 100% | 0 | 100 | 5596 | 5596 | MT090963.1 |
|  | *Klebsiella pneumoniae* strain KP18-2172 plasmid pKP18-2172-3, complete sequence | *Klebsiella pneumoniae* | 9756 | 10336 | 100% | 0 | 100 | 5596 | 5596 | CP082036.1 |
|  | *Klebsiella pneumoniae* strain ZRKP04 plasmid unnamed5, complete sequence | *Klebsiella pneumoniae* | 9744 | 10336 | 100% | 0 | 100 | 5596 | 5596 | CP050341.1 |
|  | *Klebsiella pneumoniae* strain 37 plasmid pKP37_6, complete sequence | *Klebsiella pneumoniae* | 9725 | 10336 | 100% | 0 | 100 | 5596 | 5596 | CP082758.1 |
|  | *Klebsiella pneumoniae* *subsp.* pneumoniae strain KP21 plasmid p21_3, complete sequence | *Klebsiella pneumoniae* *subsp.* pneumoniae | 9718 | 10336 | 100% | 0 | 100 | 5596 | 5596 | CP101545.1 |
|  | *Klebsiella pneumoniae* strain KPN142 plasmid pn142_3, complete sequence | *Klebsiella pneumoniae* | 9607 | 10336 | 100% | 0 | 100 | 5596 | 5596 | CP053878.1 |
|  | *Klebsiella pneumoniae* strain WSCRKP plasmid pWSCRKP-5, complete sequence | *Klebsiella pneumoniae* | 9542 | 10336 | 100% | 0 | 100 | 5596 | 5596 | CP091073.1 |
|  | *Klebsiella pneumoniae* strain 21091025 plasmid p21091025_4, complete sequence | *Klebsiella pneumoniae* | 9527 | 10336 | 100% | 0 | 100 | 5596 | 5596 | CP095268.1 |
|  | *Klebsiella pneumoniae* isolate 99060032-b809-11e8-aae5-3c4a9275d6c8 genome assembly, chromosome: 1 | *Klebsiella pneumoniae* | 9498 | 10334 | 99% | 0 | 100 | 5596 | 5479501 | LR596812.1 |
|  | *Klebsiella pneumoniae* strain CRKP66R plasmid pCRKP66R-6, complete sequence | *Klebsiella pneumoniae* | 9489 | 10336 | 100% | 0 | 100 | 5596 | 5596 | CP063838.1 |
|  | *Klebsiella pneumoniae* strain KP20194c plasmid pKP20194c-p5, complete sequence | *Klebsiella pneumoniae* | 9483 | 10330 | 100% | 0 | 99.98 | 5596 | 5596 | CP054761.1 |
|  | *Klebsiella pneumoniae* strain WCHKP115068 plasmid p3_115068, complete sequence | *Klebsiella pneumoniae* | 9474 | 10336 | 100% | 0 | 100 | 5596 | 5596 | CP036370.1 |
|  | *Klebsiella pneumoniae* strain hvKP319 plasmid unnamed5, complete sequence | *Klebsiella pneumoniae* | 9263 | 10336 | 100% | 0 | 100 | 5596 | 5596 | CP101769.1 |
|  | *Klebsiella pneumoniae* strain KP-CT77 plasmid unnamed2, complete sequence | *Klebsiella pneumoniae* | 9225 | 10336 | 100% | 0 | 100 | 5596 | 5596 | CP080308.1 |
|  | *Klebsiella pneumoniae* strain XH1508 plasmid pXH1508-5, complete sequence | *Klebsiella pneumoniae* | 9160 | 10336 | 100% | 0 | 100 | 5596 | 5596 | CP092791.1 |
|  | *Klebsiella pneumoniae* strain KP18-3-8 plasmid pKP18-3-8_5_KB, complete sequence | *Klebsiella pneumoniae* | 9138 | 10336 | 100% | 0 | 100 | 5596 | 5596 | MT035878.1 |
|  | *Klebsiella pneumoniae* strain 21080937 plasmid p21080937_9, complete sequence | *Klebsiella pneumoniae* | 9086 | 10336 | 100% | 0 | 100 | 5596 | 5596 | CP095262.1 |
|  | *Klebsiella pneumoniae* strain WCHKP090357 plasmid p3_090357, complete sequence | *Klebsiella pneumoniae* | 9022 | 10336 | 100% | 0 | 100 | 5596 | 5596 | CP066527.1 |
|  | *Klebsiella pneumoniae* strain WCHKP020098 plasmid p3_020098, complete sequence | *Klebsiella pneumoniae* | 9005 | 10336 | 100% | 0 | 100 | 5596 | 5596 | CP036309.1 |
|  | *Klebsiella pneumoniae* strain SCKP020009 plasmid p2_020009, complete sequence | *Klebsiella pneumoniae* | 8999 | 10330 | 100% | 0 | 99.98 | 5596 | 5596 | CP038006.1 |
|  | *Klebsiella pneumoniae* strain ARLG-4861 plasmid pC592_5, complete sequence | *Klebsiella pneumoniae* | 8994 | 8994 | 99% | 0 | 95.84 | 5596 | 5584 | CP067621.1 |
|  | *Klebsiella pneumoniae* strain KPCZA02 plasmid pKPCZA02_3, complete sequence | *Klebsiella pneumoniae* | 8986 | 10334 | 99% | 0 | 100 | 5596 | 5595 | CP058229.1 |
|  | *Klebsiella pneumoniae* strain WCHKP020037 plasmid p3_020037, complete sequence | *Klebsiella pneumoniae* | 8955 | 10336 | 100% | 0 | 100 | 5596 | 5596 | CP036375.1 |
|  | *Klebsiella pneumoniae* strain 19PDR22 plasmid p8, complete sequence | *Klebsiella pneumoniae* | 8944 | 10330 | 100% | 0 | 99.98 | 5596 | 5596 | CP076552.1 |
|  | *Klebsiella pneumoniae* strain FRPDR plasmid pFRPDR_6, complete sequence | *Klebsiella pneumoniae* | 8879 | 10336 | 100% | 0 | 100 | 5596 | 5596 | CP063765.1 |
|  | *Klebsiella pneumoniae* strain 6712.08 plasmid p4-6712.08, complete sequence | *Klebsiella pneumoniae* | 8835 | 10182 | 100% | 0 | 99.47 | 5596 | 5598 | CP083004.1 |
|  | *Klebsiella pneumoniae* strain hvKP841 plasmid unnamed5, complete sequence | *Klebsiella pneumoniae* | 8765 | 10336 | 100% | 0 | 100 | 5596 | 5596 | CP101789.1 |
|  | *Klebsiella pneumoniae* strain FK 6768 plasmid unnamed5, complete sequence | *Klebsiella pneumoniae* | 8748 | 10336 | 100% | 0 | 100 | 5596 | 5596 | CP065559.1 |
|  | *Klebsiella pneumoniae* strain KP47693 plasmid p47693_1, complete sequence | *Klebsiella pneumoniae* | 8709 | 10097 | 100% | 0 | 99.46 | 5596 | 5585 | CP070527.1 |
|  | *Klebsiella pneumoniae* strain XHKP53 plasmid pXHKP53-3, complete sequence | *Klebsiella pneumoniae* | 8685 | 9926 | 100% | 0 | 99.07 | 5596 | 5567 | CP066894.1 |
|  | *Klebsiella pneumoniae* *subsp.* pneumoniae strain WCHKP020039 plasmid p2_020039, complete sequence | *Klebsiella pneumoniae* *subsp.* pneumoniae | 8680 | 10336 | 100% | 0 | 100 | 5596 | 5596 | CP043347.1 |
|  | *Klebsiella pneumoniae* *subsp.* pneumoniae strain DD01653 plasmid pDD01653-5, complete sequence | *Klebsiella pneumoniae* *subsp.* pneumoniae | 8643 | 10336 | 100% | 0 | 100 | 5596 | 5596 | CP087656.1 |
|  | *Klebsiella pneumoniae* strain KP20194f plasmid pKP20194f-p5, complete sequence | *Klebsiella pneumoniae* | 8634 | 10330 | 100% | 0 | 99.98 | 5596 | 5596 | CP054725.1 |
|  | *Klebsiella pneumoniae* strain WCHKP36 plasmid p3_020036, complete sequence | *Klebsiella pneumoniae* | 8591 | 10336 | 100% | 0 | 100 | 5596 | 5596 | CP028581.2 |
|  | *Klebsiella pneumoniae* *subsp.* pneumoniae strain DD02297 plasmid pDD02297-4, complete sequence | *Klebsiella pneumoniae* *subsp.* pneumoniae | 8540 | 10336 | 100% | 0 | 100 | 5596 | 5596 | CP087638.1 |
|  | *Klebsiella pneumoniae* strain SH12 plasmid pSH12_5, complete sequence | *Klebsiella pneumoniae* | 8514 | 10336 | 100% | 0 | 100 | 5596 | 5596 | CP040838.1 |
|  | *Klebsiella pneumoniae* strain KP18-2050 plasmid pKP18-2050-4, complete sequence | *Klebsiella pneumoniae* | 8462 | 10336 | 100% | 0 | 100 | 5596 | 5596 | CP082018.1 |
|  | *Klebsiella pneumoniae* *subsp.* pneumoniae strain KP29 plasmid p29_4, complete sequence | *Klebsiella pneumoniae* *subsp.* pneumoniae | 8447 | 10336 | 100% | 0 | 100 | 5596 | 5596 | CP101571.1 |
|  | *Klebsiella pneumoniae* strain JX-CR-hvKP-4 plasmid pJX4-5, complete sequence | *Klebsiella pneumoniae* | 8447 | 10336 | 100% | 0 | 100 | 5596 | 5596 | CP064240.1 |
|  | *Klebsiella pneumoniae* strain WCHKP020115 plasmid p2_020115, complete sequence | *Klebsiella pneumoniae* | 8442 | 10336 | 100% | 0 | 100 | 5596 | 5596 | CP043356.1 |
|  | *Klebsiella pneumoniae* isolate 91eed288-b809-11e8-aae5-3c4a9275d6c8 genome assembly, chromosome: 1 | *Klebsiella pneumoniae* | 8438 | 10761 | 100% | 0 | 100 | 5596 | 5510473 | LR596808.1 |
|  | *Klebsiella pneumoniae* strain WCHKP090374 plasmid p2_090374, complete sequence | *Klebsiella pneumoniae* | 8431 | 10328 | 100% | 0 | 99.98 | 5596 | 5595 | CP066538.1 |
|  | *Klebsiella pneumoniae* strain WCHKP8F4 plasmid p2_095084, complete sequence | *Klebsiella pneumoniae* | 8421 | 10336 | 100% | 0 | 100 | 5596 | 5596 | CP027066.3 |
|  | *Klebsiella pneumoniae* *subsp.* pneumoniae strain DD01754 plasmid pDD01754-5, complete sequence | *Klebsiella pneumoniae* *subsp.* pneumoniae | 8287 | 10336 | 100% | 0 | 100 | 5596 | 5596 | CP087650.1 |
|  | *Klebsiella pneumoniae* strain WCHKP115038 plasmid p2_115038, complete sequence | *Klebsiella pneumoniae* | 8287 | 10336 | 100% | 0 | 100 | 5596 | 5596 | CP043607.1 |
|  | *Klebsiella pneumoniae* *subsp.* pneumoniae strain KPN857 plasmid pE, complete sequence | *Klebsiella pneumoniae* *subsp.* pneumoniae | 8165 | 10336 | 100% | 0 | 100 | 5596 | 5596 | CP090437.1 |
|  | *Klebsiella pneumoniae* strain C789 plasmid unnamed2, complete sequence | *Klebsiella pneumoniae* | 8137 | 10336 | 100% | 0 | 100 | 5596 | 5596 | CP034419.1 |
|  | *Klebsiella pneumoniae* strain FDAARGOS_443 plasmid unnamed5, complete sequence | *Klebsiella pneumoniae* | 8117 | 10336 | 100% | 0 | 100 | 5596 | 5596 | CP023936.1 |
|  | *Klebsiella pneumoniae* strain WCHKP040035 plasmid p2_040035, complete sequence | *Klebsiella pneumoniae* | 8106 | 10336 | 100% | 0 | 100 | 5596 | 5596 | CP028795.2 |
|  | *Klebsiella pneumoniae* *subsp.* pneumoniae strain SCKP020079 plasmid p2_020079, complete sequence | *Klebsiella pneumoniae* *subsp.* pneumoniae | 8104 | 10336 | 100% | 0 | 100 | 5596 | 5596 | CP029380.1 |
|  | *Klebsiella pneumoniae* strain L482 plasmid p6_L382, complete sequence | *Klebsiella pneumoniae* | 8058 | 10336 | 100% | 0 | 100 | 5596 | 5596 | CP033965.1 |
|  | *Klebsiella pneumoniae* strain RIVM_C014947 plasmid pRIVM_C014947_4 | *Klebsiella pneumoniae* | 8050 | 10325 | 100% | 0 | 100 | 5596 | 5596 | MT560067.1 |
|  | *Klebsiella pneumoniae* *subsp.* pneumoniae strain DD02280 plasmid pDD02280-4, complete sequence | *Klebsiella pneumoniae* *subsp.* pneumoniae | 7973 | 10336 | 100% | 0 | 100 | 5596 | 5596 | CP087627.1 |
|  | *Klebsiella pneumoniae* strain KP20194c5 plasmid pKP20194c5-p5, complete sequence | *Klebsiella pneumoniae* | 7899 | 10330 | 100% | 0 | 100 | 5596 | 5596 | CP054743.1 |
|  | *Klebsiella pneumoniae* strain 150040X1B1 plasmid p2_150040X1B1, complete sequence | *Klebsiella pneumoniae* | 7856 | 10336 | 100% | 0 | 100 | 5596 | 5596 | CP101730.1 |
|  | Citrobacter koseri strain FDAARGOS_1029 plasmid unnamed1, complete sequence | Citrobacter koseri | 7836 | 10190 | 100% | 0 | 99.44 | 5596 | 5601 | CP066090.1 |
|  | *Klebsiella pneumoniae* strain WCHKP649 plasmid p2_095649, complete sequence | *Klebsiella pneumoniae* | 7834 | 10336 | 100% | 0 | 100 | 5596 | 5596 | CP026583.3 |
|  | *Klebsiella pneumoniae* strain CR-HvKP2 plasmid p5-CR-HvKP2, complete sequence | *Klebsiella pneumoniae* | 7786 | 10336 | 100% | 0 | 100 | 5596 | 5596 | MW598249.1 |
|  | *Klebsiella pneumoniae* strain WCHKP090329 plasmid p3_090329, complete sequence | *Klebsiella pneumoniae* | 7742 | 10336 | 100% | 0 | 100 | 5596 | 5596 | CP066522.1 |
|  | *Klebsiella pneumoniae* strain hvKP323 plasmid unnamed1, complete sequence | *Klebsiella pneumoniae* | 7705 | 10336 | 100% | 0 | 100 | 5596 | 5596 | CP101771.1 |
|  | *Klebsiella pneumoniae* strain CR-HvKP1 plasmid pCR-HvKP1-p5, complete sequence | *Klebsiella pneumoniae* | 7692 | 10336 | 100% | 0 | 100 | 5596 | 5596 | CP040538.1 |
|  | *Klebsiella pneumoniae* isolate 392 genome assembly, plasmid: P4 | *Klebsiella pneumoniae* | 7690 | 10293 | 100% | 0 | 99.95 | 5596 | 5599 | OW848883.1 |
|  | *Klebsiella pneumoniae* isolate 392 genome assembly, plasmid: P3 | *Klebsiella pneumoniae* | 7690 | 10293 | 100% | 0 | 99.95 | 5596 | 5599 | OW849089.1 |
|  | *Klebsiella pneumoniae* strain K64 plasmid p6, complete sequence | *Klebsiella pneumoniae* | 7677 | 10330 | 100% | 0 | 99.98 | 5596 | 5596 | CP102396.1 |
|  | *Klebsiella pneumoniae* strain KP20194a plasmid pKP20194a-p5, complete sequence | *Klebsiella pneumoniae* | 7664 | 10330 | 100% | 0 | 100 | 5596 | 5596 | CP054785.1 |
|  | *Klebsiella pneumoniae* *subsp.* pneumoniae strain WCHKP020120 plasmid p3_020120, complete sequence | *Klebsiella pneumoniae* *subsp.* pneumoniae | 7627 | 10336 | 100% | 0 | 100 | 5596 | 5596 | CP043361.1 |
|  | *Klebsiella pneumoniae* strain 50700 plasmid p50700-5.596, complete sequence | *Klebsiella pneumoniae* | 7607 | 10336 | 100% | 0 | 100 | 5596 | 5596 | CP088992.1 |
|  | *Klebsiella pneumoniae* strain JX-CR-hvKP-10 plasmid pJX10-4, complete sequence | *Klebsiella pneumoniae* | 7607 | 10336 | 100% | 0 | 100 | 5596 | 5596 | CP064262.1 |
|  | *Klebsiella pneumoniae* isolate 392 genome assembly, plasmid: P4 | *Klebsiella pneumoniae* | 7544 | 10293 | 100% | 0 | 99.95 | 5596 | 5599 | OW849049.1 |
|  | *Klebsiella pneumoniae* strain FDAARGOS_1328 plasmid unnamed5, complete sequence | *Klebsiella pneumoniae* | 7515 | 9034 | 100% | 0 | 95.18 | 5596 | 5585 | CP070085.1 |
|  | *Klebsiella pneumoniae* strain ZRKP01 plasmid unnamed5, complete sequence | *Klebsiella pneumoniae* | 7470 | 10336 | 100% | 0 | 100 | 5596 | 5596 | CP050359.1 |
|  | *Klebsiella pneumoniae* strain KPWX136 plasmid pF, complete sequence | *Klebsiella pneumoniae* | 7439 | 10336 | 100% | 0 | 100 | 5596 | 5596 | CP069175.1 |
|  | *Klebsiella pneumoniae* strain KP20194a2 plasmid pKP20194a2-p5, complete sequence | *Klebsiella pneumoniae* | 7426 | 10330 | 100% | 0 | 100 | 5596 | 5596 | CP054779.1 |
|  | *Klebsiella pneumoniae* strain WCHKP090045 plasmid p2_090045, complete sequence | *Klebsiella pneumoniae* | 7361 | 10336 | 100% | 0 | 100 | 5596 | 5596 | CP043368.1 |
|  | *Klebsiella pneumoniae* strain CP19 plasmid unnamed2, complete sequence | *Klebsiella pneumoniae* | 7245 | 10330 | 100% | 0 | 100 | 5596 | 5597 | CP073353.1 |
|  | *Klebsiella pneumoniae* strain 1632 plasmid p1632-4, complete sequence | *Klebsiella pneumoniae* | 7204 | 10336 | 100% | 0 | 100 | 5596 | 5596 | CP084501.1 |
|  | *Klebsiella pneumoniae* strain KPN361 plasmid pKPN361-4, complete sequence | *Klebsiella pneumoniae* | 6881 | 10336 | 100% | 0 | 100 | 5596 | 5596 | CP053020.1 |
|  | *Klebsiella pneumoniae* strain KP20194c3 plasmid pKP20194c3-p5, complete sequence | *Klebsiella pneumoniae* | 6879 | 10330 | 100% | 0 | 100 | 5596 | 5596 | CP054755.1 |
|  | *Klebsiella pneumoniae* strain 015625 plasmid p3_015625, complete sequence | *Klebsiella pneumoniae* | 6879 | 10336 | 100% | 0 | 100 | 5596 | 5596 | CP033393.2 |
| pCR-hvKP221-Vir-P1 | *Klebsiella pneumoniae* *subsp.* pneumoniae strain SH2 plasmid pSH2-KPC, complete sequence | *Klebsiella pneumoniae* *subsp.* pneumoniae | 1.58E+05 | 3.56E+05 | 99% | 0 | 99.73 | 145168 | 302845 | MH643791.1 |
|  | *Klebsiella pneumoniae* strain XHKP502 plasmid pXHKP502-1, complete sequence | *Klebsiella pneumoniae* | 1.40E+05 | 3.76E+05 | 99% | 0 | 99.97 | 145168 | 221472 | CP066905.1 |
|  | *Klebsiella pneumoniae* strain FK 6768 plasmid unnamed1, complete sequence | *Klebsiella pneumoniae* | 1.24E+05 | 3.65E+05 | 99% | 0 | 99.98 | 145168 | 219459 | CP065555.1 |
|  | *Klebsiella pneumoniae* strain XJ-K2 plasmid unnamed1, complete sequence | *Klebsiella pneumoniae* | 1.24E+05 | 3.66E+05 | 99% | 0 | 99.97 | 145168 | 219775 | CP032241.1 |
|  | *Klebsiella pneumoniae* strain JNKPN26 plasmid pJNKPN26_Vir, complete sequence | *Klebsiella pneumoniae* | 1.24E+05 | 3.69E+05 | 99% | 0 | 99.91 | 145168 | 220975 | CP090206.1 |
|  | *Escherichia coli* strain EC2648-Vir1 plasmid pVir-fusion, complete sequence | *Escherichia coli* | 1.17E+05 | 3.69E+05 | 99% | 0 | 99.99 | 145168 | 255611 | CP083611.1 |
|  | *Escherichia coli* strain EC2648-Vir2 plasmid pKP2648-VirV, complete sequence | *Escherichia coli* | 1.10E+05 | 3.64E+05 | 99% | 0 | 99.99 | 145168 | 222599 | CP083992.1 |
|  | *Klebsiella pneumoniae* strain CRKP78R plasmid p2, complete sequence | *Klebsiella pneumoniae* | 98200 | 3.36E+05 | 99% | 0 | 100 | 145168 | 215966 | CP066255.1 |
|  | *Klebsiella pneumoniae* *subsp.* pneumoniae strain SCKP020079 plasmid pVir_020079, complete sequence | *Klebsiella pneumoniae* *subsp.* pneumoniae | 98143 | 3.34E+05 | 90% | 0 | 99.98 | 145168 | 178741 | CP029383.2 |
|  | *Klebsiella pneumoniae* strain L388 plasmid p1-L388 | *Klebsiella pneumoniae* | 98143 | 3.36E+05 | 99% | 0 | 99.98 | 145168 | 217870 | CP029221.1 |
|  | *Klebsiella pneumoniae* strain KP1878 plasmid p1878-217k, complete sequence | *Klebsiella pneumoniae* | 98122 | 3.48E+05 | 98% | 0 | 99.97 | 145168 | 217255 | CP072997.1 |
|  | *Klebsiella pneumoniae* strain BSI030 plasmid pBSI030_vf | *Klebsiella pneumoniae* | 98063 | 3.32E+05 | 95% | 0 | 99.94 | 145168 | 227912 | MT269852.1 |
|  | *Klebsiella pneumoniae* strain F16KP0082 plasmid pF16KP0082-1, complete sequence | *Klebsiella pneumoniae* | 98039 | 3.32E+05 | 98% | 0 | 99.95 | 145168 | 232142 | CP052163.1 |
|  | *Klebsiella pneumoniae* strain CRKP52R plasmid p2, complete sequence | *Klebsiella pneumoniae* | 98034 | 3.43E+05 | 99% | 0 | 99.95 | 145168 | 193221 | CP066250.1 |
|  | *Klebsiella pneumoniae* strain JNKPN26 plasmid pJNKPN26_Vir | *Klebsiella pneumoniae* | 97886 | 3.69E+05 | 99% | 0 | 99.89 | 145168 | 220975 | MZ546617.1 |
|  | *Klebsiella pneumoniae* *subsp.* pneumoniae strain SH9 plasmid pSH9-VIR, complete sequence | *Klebsiella pneumoniae* *subsp.* pneumoniae | 97880 | 3.10E+05 | 92% | 0 | 99.88 | 145168 | 188437 | MH255828.1 |
|  | *Klebsiella pneumoniae* *subsp.* pneumoniae strain HA2 plasmid pHA2-23-vir, complete sequence | *Klebsiella pneumoniae* *subsp.* pneumoniae | 96976 | 3.46E+05 | 99% | 0 | 99.59 | 145168 | 217926 | MH643788.1 |
|  | *Klebsiella pneumoniae* strain JX-CR-hvKP-4 plasmid pJX4-1, complete sequence | *Klebsiella pneumoniae* | 95020 | 3.61E+05 | 99% | 0 | 100 | 145168 | 219405 | CP064236.1 |
|  | *Klebsiella pneumoniae* *subsp.* pneumoniae strain RJF999 plasmid pRJF999, complete sequence | *Klebsiella pneumoniae* *subsp.* pneumoniae | 95014 | 3.44E+05 | 96% | 0 | 100 | 145168 | 228907 | CP014011.1 |
|  | *Klebsiella pneumoniae* strain 11420 plasmid p11420-HVKP, complete sequence | *Klebsiella pneumoniae* | 95011 | 3.44E+05 | 96% | 0 | 99.99 | 145168 | 229796 | CP026024.1 |
|  | *Klebsiella pneumoniae* strain GZ-1 plasmid unnamed1, complete sequence | *Klebsiella pneumoniae* | 95011 | 3.68E+05 | 99% | 0 | 99.99 | 145168 | 198126 | CP031935.1 |
|  | *Klebsiella pneumoniae* strain 21080237 plasmid p21080237_1, complete sequence | *Klebsiella pneumoniae* | 94942 | 3.61E+05 | 99% | 0 | 99.99 | 145168 | 188001 | CP095241.1 |
|  | *Klebsiella pneumoniae* strain 9949 plasmid unnamed1, complete sequence | *Klebsiella pneumoniae* | 94845 | 3.97E+05 | 99% | 0 | 99.94 | 145168 | 237416 | CP050281.1 |
|  | *Klebsiella pneumoniae* *subsp.* pneumoniae strain HA2 plasmid pHA2-23-vir, complete sequence | *Klebsiella pneumoniae* *subsp.* pneumoniae | 94457 | 3.46E+05 | 99% | 0 | 99.91 | 145168 | 214938 | MH643786.1 |
|  | *Klebsiella pneumoniae* strain Kp36 plasmid unnamed1, complete sequence | *Klebsiella pneumoniae* | 93360 | 3.65E+05 | 99% | 0 | 99.95 | 145168 | 219800 | CP047193.1 |
|  | *Klebsiella pneumoniae* strain S270v plasmid pS270V-1, complete sequence | *Klebsiella pneumoniae* | 88804 | 3.35E+05 | 99% | 0 | 100 | 145168 | 221246 | CP102193.1 |
|  | *Klebsiella pneumoniae* strain XJ-K1 plasmid unnamed1, complete sequence | *Klebsiella pneumoniae* | 83973 | 3.52E+05 | 96% | 0 | 99.98 | 145168 | 207409 | CP032164.1 |
|  | *Klebsiella pneumoniae* strain KP2648 plasmid pKP2648-Vir, complete sequence | *Klebsiella pneumoniae* | 82461 | 3.62E+05 | 99% | 0 | 99.99 | 145168 | 216902 | CP072558.1 |
|  | *Klebsiella pneumoniae* strain BSI074 plasmid pBSI074_vf | *Klebsiella pneumoniae* | 73217 | 3.44E+05 | 99% | 0 | 99.99 | 145168 | 218366 | MT269847.1 |
|  | *Klebsiella pneumoniae* strain C789 plasmid pVir-CR-hvKP-C789, complete sequence | *Klebsiella pneumoniae* | 73183 | 3.36E+05 | 99% | 0 | 99.97 | 145168 | 215950 | CP034416.1 |
|  | *Klebsiella pneumoniae* strain 33367 plasmid p33367_VIR, complete sequence | *Klebsiella pneumoniae* | 73183 | 3.34E+05 | 99% | 0 | 99.97 | 145168 | 215836 | CP099414.1 |
|  | *Klebsiella pneumoniae* strain C2582 plasmid pVir_C2582 | *Klebsiella pneumoniae* | 73183 | 3.62E+05 | 99% | 0 | 99.97 | 145168 | 216779 | CP079209.1 |
|  | *Klebsiella pneumoniae* strain CRKP66R plasmid pCRKP66R-2, complete sequence | *Klebsiella pneumoniae* | 73183 | 3.36E+05 | 99% | 0 | 99.97 | 145168 | 215999 | CP063834.1 |
|  | *Klebsiella pneumoniae* strain KPC-2 plasmid pKP169-P1, complete sequence | *Klebsiella pneumoniae* | 73178 | 3.51E+05 | 98% | 0 | 99.97 | 145168 | 221577 | CP078124.1 |
|  | *Klebsiella pneumoniae* strain 50700 plasmid p50700-217.1, complete sequence | *Klebsiella pneumoniae* | 73165 | 3.38E+05 | 99% | 0 | 99.96 | 145168 | 217070 | CP088996.1 |
|  | *Klebsiella pneumoniae* strain FRPDR plasmid pFRPDR_1, complete sequence | *Klebsiella pneumoniae* | 73139 | 3.65E+05 | 99% | 0 | 99.95 | 145168 | 219933 | CP063760.1 |
|  | *Klebsiella pneumoniae* strain CDI694 plasmid pCDI694-216.6, complete sequence | *Klebsiella pneumoniae* | 73085 | 3.62E+05 | 99% | 0 | 99.93 | 145168 | 216645 | CP077778.1 |
|  | *Klebsiella pneumoniae* strain 8695 plasmid pFK8695-rmpA, complete sequence | *Klebsiella pneumoniae* | 72993 | 2.94E+05 | 79% | 0 | 99.99 | 145168 | 150052 | CP085891.1 |
|  | *Klebsiella pneumoniae* strain QL24 plasmid pKPN-QL24, complete sequence | *Klebsiella pneumoniae* | 72236 | 3.28E+05 | 99% | 0 | 99.54 | 145168 | 215940 | MH263654.1 |
|  | *Klebsiella pneumoniae* strain C2244 plasmid pCRHV-C2244, complete sequence | *Klebsiella pneumoniae* | 69710 | 3.60E+05 | 98% | 0 | 100 | 145168 | 293391 | MT644086.1 |
|  | *Klebsiella pneumoniae* strain 108 plasmid pBJ108-HI3, complete sequence | *Klebsiella pneumoniae* | 68449 | 3.52E+05 | 97% | 0 | 99.7 | 145168 | 211194 | MW013144.1 |
|  | *Klebsiella pneumoniae* strain JX-CR-hvKP-11 plasmid pJX11-1, complete sequence | *Klebsiella pneumoniae* | 67115 | 3.29E+05 | 96% | 0 | 99.96 | 145168 | 228028 | CP064209.1 |
|  | *Klebsiella pneumoniae* strain KPN115 plasmid pKPN115_1, complete sequence | *Klebsiella pneumoniae* | 67099 | 3.42E+05 | 95% | 0 | 99.95 | 145168 | 234056 | CP089860.1 |
|  | *Klebsiella pneumoniae* strain P1428 chromosome, complete genome | *Klebsiella pneumoniae* | 67097 | 3.57E+05 | 94% | 0 | 99.95 | 145168 | 5633290 | CP017994.1 |
|  | *Klebsiella pneumoniae* strain ED23 plasmid unamed, complete sequence | *Klebsiella pneumoniae* | 66617 | 3.01E+05 | 85% | 0 | 99.75 | 145168 | 212770 | CP016815.1 |
|  | *Klebsiella pneumoniae* strain SP4663 plasmid pIncHI1B_vir, complete sequence | *Klebsiella pneumoniae* | 66550 | 3.20E+05 | 96% | 0 | 99.95 | 145168 | 228289 | CP069366.1 |
|  | *Klebsiella pneumoniae* strain JX-CR-hvKP-7 plasmid pJX7-1, complete sequence | *Klebsiella pneumoniae* | 66428 | 3.65E+05 | 99% | 0 | 99.97 | 145168 | 219796 | CP064224.1 |
|  | *Klebsiella pneumoniae* strain JX-CR-hvKP-8 plasmid pJX8-1, complete sequence | *Klebsiella pneumoniae* | 66428 | 3.59E+05 | 99% | 0 | 99.97 | 145168 | 217869 | CP064218.1 |
|  | *Klebsiella pneumoniae* strain JX-CR-hvKP-3 plasmid pJX3-1, complete sequence | *Klebsiella pneumoniae* | 66399 | 3.68E+05 | 99% | 0 | 99.95 | 145168 | 221242 | CP064242.1 |
|  | *Klebsiella pneumoniae* *subsp.* pneumoniae strain KUH-KPNHVF1 plasmid unnamed, complete sequence | *Klebsiella pneumoniae* *subsp.* pneumoniae | 65830 | 3.13E+05 | 96% | 0 | 99.94 | 145168 | 229024 | CP047676.1 |
|  | *Klebsiella pneumoniae* *subsp.* pneumoniae strain KUH-KPNHVL1 plasmid unnamed, complete sequence | *Klebsiella pneumoniae* *subsp.* pneumoniae | 65824 | 3.13E+05 | 96% | 0 | 99.94 | 145168 | 229024 | CP047678.1 |
|  | *Klebsiella pneumoniae* strain C1398 plasmid pVir-CR-hvKP-C1398, complete sequence | *Klebsiella pneumoniae* | 65732 | 3.23E+05 | 88% | 0 | 99.98 | 145168 | 274540 | CP034421.1 |
|  | *Klebsiella pneumoniae* strain KP-Zhen plasmid unnamed1, complete sequence | *Klebsiella pneumoniae* | 65686 | 2.93E+05 | 94% | 0 | 99.86 | 145168 | 222711 | CP053863.1 |
|  | *Klebsiella pneumoniae* strain IR12061_1 plasmid unnamed1, complete sequence | *Klebsiella pneumoniae* | 65167 | 3.22E+05 | 88% | 0 | 99.91 | 145168 | 274502 | CP097680.1 |
|  | *Klebsiella pneumoniae* strain 79 plasmid pZZ79-HI3, complete sequence | *Klebsiella pneumoniae* | 65167 | 3.20E+05 | 87% | 0 | 99.91 | 145168 | 259691 | MW013143.1 |
|  | *Klebsiella pneumoniae* strain KP18-2079 plasmid pKP18-2079_vir, complete sequence | *Klebsiella pneumoniae* | 63996 | 2.16E+05 | 66% | 0 | 99.88 | 145168 | 182326 | MT090958.1 |
|  | *Klebsiella pneumoniae* strain KP267 plasmid pVir-CR-HvKP267, complete sequence | *Klebsiella pneumoniae* | 63956 | 3.61E+05 | 99% | 0 | 99.97 | 145168 | 233470 | MG053312.1 |
|  | *Klebsiella pneumoniae* strain KP20194a plasmid pKP20194a-p1, complete sequence | *Klebsiella pneumoniae* | 63804 | 3.28E+05 | 97% | 0 | 99.99 | 145168 | 195031 | CP054781.1 |
|  | *Klebsiella pneumoniae* strain KP20194a2 plasmid pKP20194a2-p1, complete sequence | *Klebsiella pneumoniae* | 63804 | 3.28E+05 | 97% | 0 | 99.99 | 145168 | 195030 | CP054775.1 |
|  | *Klebsiella pneumoniae* strain KP20194b plasmid pKP20194b-p1, complete sequence | *Klebsiella pneumoniae* | 63804 | 3.28E+05 | 97% | 0 | 99.99 | 145168 | 195034 | CP054769.1 |
|  | *Klebsiella pneumoniae* strain KP20194c4 plasmid pKP20194c4-p1, complete sequence | *Klebsiella pneumoniae* | 63804 | 3.28E+05 | 97% | 0 | 99.99 | 145168 | 195025 | CP054745.1 |
|  | *Klebsiella pneumoniae* strain KP20194c5 plasmid pKP20194c5-p1, complete sequence | *Klebsiella pneumoniae* | 63804 | 3.28E+05 | 97% | 0 | 99.99 | 145168 | 195034 | CP054739.1 |
|  | *Klebsiella pneumoniae* strain KP20194c3 plasmid pKP20194c3-p1, complete sequence | *Klebsiella pneumoniae* | 63804 | 3.28E+05 | 97% | 0 | 99.99 | 145168 | 195028 | CP054751.1 |
|  | *Klebsiella pneumoniae* strain KP20194d plasmid pKP20194d-p1, complete sequence | *Klebsiella pneumoniae* | 63804 | 3.25E+05 | 97% | 0 | 99.99 | 145168 | 194896 | CP054733.1 |
|  | *Klebsiella pneumoniae* strain KP20194e plasmid pKP20194e-p1, complete sequence | *Klebsiella pneumoniae* | 63804 | 3.28E+05 | 97% | 0 | 99.99 | 145168 | 195027 | CP054727.1 |
|  | *Klebsiella pneumoniae* strain KP20194f plasmid pKP20194f-p1, complete sequence | *Klebsiella pneumoniae* | 63804 | 3.25E+05 | 97% | 0 | 99.99 | 145168 | 194901 | CP054721.1 |
|  | *Klebsiella pneumoniae* strain KP20194c plasmid pKP20194c-p1, complete sequence | *Klebsiella pneumoniae* | 63804 | 3.28E+05 | 97% | 0 | 99.99 | 145168 | 195024 | CP054757.1 |
|  | *Klebsiella pneumoniae* strain KP20194b2 plasmid pKP20194b2-p1, complete sequence | *Klebsiella pneumoniae* | 63804 | 3.29E+05 | 97% | 0 | 99.99 | 145168 | 195077 | CP054763.1 |
|  | *Klebsiella pneumoniae* strain KP58 plasmid pKP58-1, complete sequence | *Klebsiella pneumoniae* | 63804 | 3.42E+05 | 99% | 0 | 99.99 | 145168 | 197415 | CP041374.1 |
|  | *Klebsiella pneumoniae* strain L39_2 plasmid p2_L39, complete sequence | *Klebsiella pneumoniae* | 63804 | 3.69E+05 | 99% | 0 | 99.99 | 145168 | 198087 | CP033955.1 |
|  | *Klebsiella pneumoniae* strain KP15 plasmid unnamed1, complete sequence | *Klebsiella pneumoniae* | 63804 | 3.44E+05 | 99% | 0 | 99.99 | 145168 | 197415 | CP087143.1 |
|  | *Klebsiella pneumoniae* strain KP16 plasmid unnamed1, complete sequence | *Klebsiella pneumoniae* | 63804 | 3.44E+05 | 99% | 0 | 99.99 | 145168 | 197415 | CP087147.1 |
|  | *Klebsiella pneumoniae* strain KP14 plasmid unnamed1, complete sequence | *Klebsiella pneumoniae* | 63804 | 3.44E+05 | 99% | 0 | 99.99 | 145168 | 197415 | CP087152.1 |
|  | *Klebsiella pneumoniae* strain XH1507 plasmid pXH1507-1, complete sequence | *Klebsiella pneumoniae* | 63804 | 3.47E+05 | 99% | 0 | 99.99 | 145168 | 198615 | CP092794.1 |
|  | *Klebsiella pneumoniae* strain XH1508 plasmid pXH1508-1, complete sequence | *Klebsiella pneumoniae* | 63804 | 3.47E+05 | 99% | 0 | 99.99 | 145168 | 198615 | CP092787.1 |
|  | *Klebsiella pneumoniae* strain KP-CT77 plasmid pCT77-Vir, complete sequence | *Klebsiella pneumoniae* | 63804 | 3.31E+05 | 96% | 0 | 99.99 | 145168 | 186446 | CP080304.1 |
|  | *Klebsiella pneumoniae* strain KP55 plasmid pKP55_1, complete sequence | *Klebsiella pneumoniae* | 63804 | 3.44E+05 | 99% | 0 | 99.99 | 145168 | 197415 | CP055295.1 |
|  | *Klebsiella pneumoniae* *subsp.* pneumoniae strain DD01653 plasmid pDD01653-1, complete sequence | *Klebsiella pneumoniae* *subsp.* pneumoniae | 63799 | 3.44E+05 | 99% | 0 | 99.99 | 145168 | 220277 | CP087652.1 |
|  | *Escherichia coli* strain EC600 plasmid p16ZR-187-fusion, complete sequence | *Escherichia coli* | 63771 | 3.45E+05 | 99% | 0 | 99.98 | 145168 | 265165 | MN182750.1 |
|  | *Klebsiella pneumoniae* strain 16ZR-187 plasmid p16ZR-187-IncHI1-197-Vir, complete sequence | *Klebsiella pneumoniae* | 63771 | 3.44E+05 | 99% | 0 | 99.98 | 145168 | 197416 | MN182749.1 |
|  | *Klebsiella pneumoniae* strain kp5152 plasmid p1, complete sequence | *Klebsiella pneumoniae* | 63767 | 3.15E+05 | 89% | 0 | 99.97 | 145168 | 176459 | CP090463.1 |
|  | *Klebsiella pneumoniae* strain KP200731214 plasmid pKP-1 | *Klebsiella pneumoniae* | 63767 | 3.44E+05 | 99% | 0 | 99.97 | 145168 | 193244 | CP084744.1 |
|  | *Klebsiella pneumoniae* strain JX-CR-hvKP-10 plasmid pJX10-1, complete sequence | *Klebsiella pneumoniae* | 63767 | 3.94E+05 | 100% | 0 | 99.97 | 145168 | 394328 | CP064259.1 |
|  | *Klebsiella pneumoniae* strain JX-CR-hvKP-9 plasmid pJX9-1, complete sequence | *Klebsiella pneumoniae* | 63767 | 3.84E+05 | 100% | 0 | 99.97 | 145168 | 277720 | CP064212.1 |
|  | *Klebsiella pneumoniae* strain KP18-3-8 plasmid pKP18-3-8_KPC_vir, complete sequence | *Klebsiella pneumoniae* | 63762 | 3.65E+05 | 100% | 0 | 99.97 | 145168 | 228158 | MT035874.1 |
|  | *Klebsiella pneumoniae* strain BJCFK909 plasmid p1b1, complete sequence | *Klebsiella pneumoniae* | 63762 | 3.76E+05 | 99% | 0 | 99.97 | 145168 | 200216 | CP034124.1 |
|  | *Klebsiella pneumoniae* strain WSCRKP plasmid pWSCRKP-1, complete sequence | *Klebsiella pneumoniae* | 63762 | 3.71E+05 | 99% | 0 | 99.97 | 145168 | 221776 | CP091069.1 |
|  | *Klebsiella pneumoniae* strain K64 plasmid pVir-1, complete sequence | *Klebsiella pneumoniae* | 63753 | 2.97E+05 | 96% | 0 | 99.97 | 145168 | 193821 | CP102391.1 |
|  | *Klebsiella pneumoniae* *subsp.* pneumoniae strain DD02280 plasmid pDD02280-1, complete sequence | *Klebsiella pneumoniae* *subsp.* pneumoniae | 63753 | 3.48E+05 | 99% | 0 | 99.97 | 145168 | 198608 | CP087624.1 |
|  | *Klebsiella pneumoniae* strain KP697 plasmid unnamed1, complete sequence | *Klebsiella pneumoniae* | 63753 | 3.44E+05 | 99% | 0 | 99.97 | 145168 | 197408 | CP066152.1 |
|  | *Klebsiella pneumoniae* strain 37 plasmid pKP37-vir, complete sequence | *Klebsiella pneumoniae* | 63753 | 3.44E+05 | 99% | 0 | 99.97 | 145168 | 193370 | CP082754.1 |
|  | *Klebsiella pneumoniae* strain 36 plasmid pKP36-vir, complete sequence | *Klebsiella pneumoniae* | 63753 | 3.44E+05 | 99% | 0 | 99.97 | 145168 | 197408 | CP082760.1 |
|  | *Klebsiella pneumoniae* strain 12 plasmid pKP12-vir, complete sequence | *Klebsiella pneumoniae* | 63753 | 3.73E+05 | 99% | 0 | 99.97 | 145168 | 199279 | CP082766.1 |
|  | *Klebsiella pneumoniae* strain KP-C76 plasmid pC76-Vir, complete sequence | *Klebsiella pneumoniae* | 63753 | 3.44E+05 | 99% | 0 | 99.97 | 145168 | 197407 | CP080298.1 |
|  | *Klebsiella pneumoniae* *subsp.* pneumoniae strain DD01845 plasmid pDD01845-1, complete sequence | *Klebsiella pneumoniae* *subsp.* pneumoniae | 63747 | 3.40E+05 | 99% | 0 | 99.97 | 145168 | 219069 | CP087664.1 |
|  | *Klebsiella pneumoniae* *subsp.* pneumoniae strain DD01635 plasmid pDD01635-1, complete sequence | *Klebsiella pneumoniae* *subsp.* pneumoniae | 63747 | 3.40E+05 | 99% | 0 | 99.97 | 145168 | 219068 | CP087658.1 |
|  | *Klebsiella pneumoniae* strain JX-CR-hvKP-12 plasmid pJX12-1, complete sequence | *Klebsiella pneumoniae* | 63747 | 3.43E+05 | 96% | 0 | 99.97 | 145168 | 230835 | CP064206.1 |
|  | *Klebsiella pneumoniae* strain NTUH-K2044-CR plasmid unnamed1, complete sequence | *Klebsiella pneumoniae* | 63745 | 3.25E+05 | 94% | 0 | 99.96 | 145168 | 227511 | MZ475709.1 |
|  | *Klebsiella pneumoniae* strain JS187-vir plasmid unnamed4, complete sequence | *Klebsiella pneumoniae* | 63745 | 3.09E+05 | 94% | 0 | 99.96 | 145168 | 226668 | MZ475703.1 |
|  | *Escherichia coli* strain J53-vir plasmid unnamed1, partial sequence | *Escherichia coli* | 63745 | 3.35E+05 | 94% | 0 | 99.96 | 145168 | 229894 | MZ475697.1 |
| pCR-hvKP221-Vir-P2 | *Escherichia coli* strain elppa10 plasmid unnamed1, complete sequence | *Escherichia coli* | 1.77E+05 | 2.48E+05 | 78% | 0 | 99.87 | 140816 | 149801 | CP083479.1 |
|  | *Escherichia coli* strain Z0117EC0036 plasmid pZ0117EC0036-1, complete sequence | *Escherichia coli* | 1.64E+05 | 2.99E+05 | 96% | 0 | 99.82 | 140816 | 157136 | CP098215.1 |
|  | *Escherichia coli* strain D6 plasmid A, complete genome | *Escherichia coli* | 1.60E+05 | 3.36E+05 | 100% | 0 | 99.86 | 140816 | 199494 | CP010149.1 |
|  | *Escherichia coli* plasmid pWP2-W18-ESBL-08_1 DNA, complete genome, strain: WP2-W18-ESBL-08 | *Escherichia coli* | 1.42E+05 | 2.02E+05 | 70% | 0 | 99.83 | 140816 | 105973 | AP021934.1 |
|  | *Escherichia coli* strain 2016C-3878 plasmid pMCR1-PA, complete sequence | *Escherichia coli* | 1.40E+05 | 2.76E+05 | 73% | 0 | 99.74 | 140816 | 276880 | CP029748.1 |
|  | *Escherichia coli* strain AR Bank #0349 plasmid pAR349, complete sequence | *Escherichia coli* | 1.40E+05 | 2.83E+05 | 73% | 0 | 99.71 | 140816 | 278067 | CP041997.1 |
|  | *Escherichia coli* strain 154AHL.1 plasmid p1, complete sequence | *Escherichia coli* | 1.40E+05 | 2.10E+05 | 65% | 0 | 99.68 | 140816 | 132199 | CP059989.1 |
|  | *Escherichia coli* strain FDAARGOS_1250 plasmid unnamed2, complete sequence | *Escherichia coli* | 1.40E+05 | 2.23E+05 | 66% | 0 | 99.69 | 140816 | 135042 | CP069572.1 |
|  | *Escherichia coli* strain AMSCJX04 plasmid pAMPD02, complete sequence | *Escherichia coli* | 1.37E+05 | 2.16E+05 | 74% | 0 | 99.76 | 140816 | 129018 | CP058310.1 |
|  | *Escherichia coli* plasmid pWP2-W18-CRE-03_1 DNA, complete genome, strain: WP2-W18-CRE-03 | *Escherichia coli* | 1.37E+05 | 2.29E+05 | 76% | 0 | 99.8 | 140816 | 158754 | AP021915.1 |
|  | *Escherichia coli* strain D3 plasmid A, complete genome | *Escherichia coli* | 1.35E+05 | 2.48E+05 | 84% | 0 | 99.79 | 140816 | 174041 | CP010141.1 |
|  | *Escherichia coli* strain EC9682 plasmid pEC9682-1, complete sequence | *Escherichia coli* | 1.35E+05 | 2.24E+05 | 78% | 0 | 99.79 | 140816 | 146265 | CP095272.1 |
|  | *Escherichia coli* strain 4410-1 genome assembly, plasmid: RCS79_p | *Escherichia coli* | 1.35E+05 | 2.49E+05 | 80% | 0 | 99.78 | 140816 | 168237 | LT985293.1 |
|  | *Escherichia coli* strain 13P460A plasmid p13P460A-2, complete sequence | *Escherichia coli* | 1.35E+05 | 2.53E+05 | 84% | 0 | 99.75 | 140816 | 169505 | CP021087.1 |
|  | *Escherichia coli* PCN033 plasmid p3PCN033, complete sequence | *Escherichia coli* PCN033 | 1.34E+05 | 2.55E+05 | 78% | 0 | 99.8 | 140816 | 161511 | CP006635.1 |
|  | *Escherichia coli* strain TREC8 plasmid pTREC8, complete sequence | *Escherichia coli* | 1.34E+05 | 2.02E+05 | 65% | 0 | 99.74 | 140816 | 118200 | MN158991.1 |
|  | *Escherichia coli* strain TREC9 plasmid pTREC9, complete sequence | *Escherichia coli* | 1.33E+05 | 2.10E+05 | 63% | 0 | 99.57 | 140816 | 128316 | MN158992.1 |
|  | *Escherichia coli* strain MRSN388634 plasmid pMR0516mcr, complete sequence | *Escherichia coli* | 1.33E+05 | 2.34E+05 | 70% | 0 | 99.72 | 140816 | 225069 | KX276657.1 |
|  | *Escherichia coli* strain 13P484A plasmid p13P484A-2, complete sequence | *Escherichia coli* | 1.33E+05 | 2.63E+05 | 83% | 0 | 99.85 | 140816 | 149520 | CP019282.1 |
|  | *Escherichia coli* strain WF5-29 plasmid pWF5-29, complete sequence | *Escherichia coli* | 1.32E+05 | 3.26E+05 | 85% | 0 | 99.76 | 140816 | 395758 | MG385063.1 |
|  | Shigella flexneri strain SWHIN_107 plasmid unnamed1, complete sequence | Shigella flexneri | 1.30E+05 | 2.97E+05 | 85% | 0 | 99.83 | 140816 | 171874 | CP055100.1 |
|  | *Escherichia coli* strain HXH-1 plasmid pHXH-1, complete sequence | *Escherichia coli* | 1.26E+05 | 1.99E+05 | 69% | 0 | 99.82 | 140816 | 124747 | MH202955.1 |
|  | *Klebsiella pneumoniae* isolate 11 genome assembly, plasmid: P1 | *Klebsiella pneumoniae* | 1.26E+05 | 1.94E+05 | 53% | 0 | 99.83 | 140816 | 157270 | OW849389.1 |
|  | *Klebsiella pneumoniae* isolate 11 genome assembly, plasmid: P1 | *Klebsiella pneumoniae* | 1.26E+05 | 2.13E+05 | 56% | 0 | 99.83 | 140816 | 171913 | OW849284.1 |
|  | *Klebsiella pneumoniae* isolate 11 genome assembly, plasmid: P1 | *Klebsiella pneumoniae* | 1.26E+05 | 2.17E+05 | 56% | 0 | 99.83 | 140816 | 173773 | OW849278.1 |
|  | *Klebsiella pneumoniae* isolate 11 genome assembly, plasmid: P1 | *Klebsiella pneumoniae* | 1.26E+05 | 2.17E+05 | 56% | 0 | 99.83 | 140816 | 173773 | OW849251.1 |
|  | *Escherichia coli* TUM18530 plasmid pMTY18530-2 DNA, complete sequence | *Escherichia coli* | 1.26E+05 | 1.78E+05 | 63% | 0 | 99.8 | 140816 | 115543 | AP023192.1 |
|  | *Escherichia coli* strain EC13 plasmid pEC13-2, complete sequence | *Escherichia coli* | 1.26E+05 | 2.46E+05 | 76% | 0 | 99.79 | 140816 | 145694 | CP060929.1 |
|  | *Escherichia coli* strain PK12 plasmid pYLPK12, complete sequence | *Escherichia coli* | 1.25E+05 | 2.16E+05 | 70% | 0 | 99.71 | 140816 | 129015 | CP074032.1 |
|  | *Escherichia coli* strain RHB02-C02 plasmid pRHB02-C02_2, complete sequence | *Escherichia coli* | 1.25E+05 | 2.07E+05 | 65% | 0 | 99.71 | 140816 | 116772 | CP058079.1 |
|  | *Escherichia coli* strain RHB02-C10 plasmid pRHB02-C10_2, complete sequence | *Escherichia coli* | 1.25E+05 | 2.07E+05 | 65% | 0 | 99.71 | 140816 | 116772 | CP058071.1 |
|  | *Escherichia coli* strain 28.1 plasmid p1, complete sequence | *Escherichia coli* | 1.25E+05 | 2.05E+05 | 65% | 0 | 99.71 | 140816 | 132062 | CP059932.1 |
|  | *Escherichia coli* strain 101.3 plasmid p1, complete sequence | *Escherichia coli* | 1.23E+05 | 2.02E+05 | 65% | 0 | 99.65 | 140816 | 116752 | CP059945.1 |
|  | *Escherichia coli* strain EC21Z-147 plasmid pEC21Z147-128K, complete sequence | *Escherichia coli* | 1.21E+05 | 2.18E+05 | 72% | 0 | 99.81 | 140816 | 127578 | CP101212.1 |
|  | *Escherichia coli* strain EC931 plasmid pEC931_1, complete sequence | *Escherichia coli* | 1.21E+05 | 2.34E+05 | 79% | 0 | 99.71 | 140816 | 159690 | CP049119.1 |
|  | *Escherichia coli* strain Z0117EC0148 plasmid pZ0117EC0148-1, complete sequence | *Escherichia coli* | 1.19E+05 | 2.36E+05 | 81% | 0 | 99.77 | 140816 | 121118 | CP098179.1 |
|  | *Escherichia coli* strain HB-Coli0 plasmid unnamed1, complete sequence | *Escherichia coli* | 1.15E+05 | 2.43E+05 | 78% | 0 | 99.83 | 140816 | 151137 | CP020934.1 |
|  | *Escherichia coli* strain elppa8 plasmid unnamed2, complete sequence | *Escherichia coli* | 1.08E+05 | 1.89E+05 | 64% | 0 | 99.73 | 140816 | 96208 | CP083493.1 |
|  | *Escherichia coli* strain 92944 plasmid p92944-mph, complete sequence | *Escherichia coli* | 1.06E+05 | 2.68E+05 | 88% | 0 | 99.92 | 140816 | 147163 | MG838205.1 |
|  | *Escherichia coli* strain CVM N18EC0432 plasmid pN18EC0432-2, complete sequence | *Escherichia coli* | 1.03E+05 | 2.11E+05 | 75% | 0 | 99.02 | 140816 | 137411 | CP048296.1 |
|  | *Escherichia coli* strain WCHEC040047 plasmid p1_040047, complete sequence | *Escherichia coli* | 98979 | 2.03E+05 | 63% | 0 | 99.8 | 140816 | 110724 | CP043297.1 |
|  | *Escherichia coli* strain CREC-544 plasmid pCREC-544_1, complete sequence | *Escherichia coli* | 98931 | 2.16E+05 | 65% | 0 | 99.76 | 140816 | 122937 | CP024827.1 |
|  | *Escherichia coli* strain 14EC020 plasmid p14EC020b, complete sequence | *Escherichia coli* | 96217 | 2.79E+05 | 84% | 0 | 99.88 | 140816 | 166233 | CP024140.1 |
|  | *Escherichia coli* strain XJW9B277 plasmid pHNXJB277, complete sequence | *Escherichia coli* | 95574 | 2.28E+05 | 70% | 0 | 99.8 | 140816 | 133299 | CP068043.1 |
|  | *Escherichia coli* strain AH01 plasmid pAH01-4, complete sequence | *Escherichia coli* | 90662 | 2.50E+05 | 83% | 0 | 99.76 | 140816 | 145790 | CP055255.1 |
|  | Enterobacteriaceae bacterium strain T11 plasmid pTRE-2011 clone contig_1 | Enterobacteriaceae bacterium | 90317 | 98098 | 35% | 0 | 99.62 | 140816 | 49473 | KX863571.1 |
|  | Shigella flexneri strain SWHIN_104 plasmid unnamed1, complete sequence | Shigella flexneri | 90261 | 3.00E+05 | 86% | 0 | 99.75 | 140816 | 177080 | CP055110.1 |
|  | *Escherichia coli* strain EC 1250 plasmid pME9, partial sequence | *Escherichia coli* | 88337 | 2.25E+05 | 78% | 0 | 99.69 | 140816 | 136946 | MT868885.1 |
|  | *Escherichia coli* strain EC_J_9 plasmid pOQX_J9_2, complete sequence | *Escherichia coli* | 86295 | 2.27E+05 | 73% | 0 | 99.87 | 140816 | 140375 | CP075063.1 |
|  | *Escherichia coli* strain EC16 plasmid pEC16-NDM-5, complete sequence | *Escherichia coli* | 86139 | 2.43E+05 | 81% | 0 | 99.74 | 140816 | 145550 | CP074121.1 |
|  | *Escherichia coli* strain W60 plasmid pECW601, complete sequence | *Escherichia coli* | 86128 | 2.35E+05 | 81% | 0 | 99.73 | 140816 | 140410 | CP058343.1 |
|  | *Escherichia coli* strain E-T306 plasmid punnamed1, complete sequence | *Escherichia coli* | 86086 | 2.67E+05 | 83% | 0 | 99.71 | 140816 | 157658 | CP090283.1 |
|  | *Escherichia coli* strain BR02-DEC chromosome | *Escherichia coli* | 85934 | 2.18E+05 | 81% | 0 | 99.73 | 140816 | 5025753 | CP035320.1 |
|  | *Escherichia coli* strain BR03-DEC chromosome | *Escherichia coli* | 85934 | 2.23E+05 | 81% | 0 | 99.73 | 140816 | 5038603 | CP035321.1 |
|  | *Escherichia coli* strain EC14 plasmid pEC14-2, complete sequence | *Escherichia coli* | 85846 | 2.12E+05 | 68% | 0 | 99.7 | 140816 | 159110 | CP060925.1 |
|  | *Escherichia coli* TUM18780 plasmid pMTY18780-2 DNA, complete sequence | *Escherichia coli* | 85840 | 1.83E+05 | 63% | 0 | 99.69 | 140816 | 115013 | AP023199.1 |
|  | Shigella flexneri strain STEFF_10 plasmid unnamed1, complete sequence | Shigella flexneri | 85818 | 2.34E+05 | 74% | 0 | 99.68 | 140816 | 125749 | CP055195.1 |
|  | *Escherichia coli* strain BR07-DEC chromosome | *Escherichia coli* | 85796 | 2.06E+05 | 77% | 0 | 99.68 | 140816 | 5052397 | CP035322.1 |
|  | *Escherichia coli* strain EC3 plasmid pEC3-2, complete sequence | *Escherichia coli* | 85704 | 2.22E+05 | 77% | 0 | 99.73 | 140816 | 140376 | CP060980.1 |
|  | *Escherichia coli* strain EC17GD31 plasmid pGD31-F1928, complete sequence | *Escherichia coli* | 85576 | 3.19E+05 | 66% | 0 | 99.2 | 140816 | 245305 | CP031295.1 |
|  | *Escherichia coli* strain 0143I clone contig 0143I-ColB-ColM | *Escherichia coli* | 85493 | 1.79E+05 | 60% | 0 | 99.56 | 140816 | 102890 | MK878525.1 |
|  | *Escherichia coli* strain RHB02-C06 plasmid pRHB02-C06_2, complete sequence | *Escherichia coli* | 85493 | 2.01E+05 | 65% | 0 | 99.56 | 140816 | 113309 | CP058075.1 |
|  | *Escherichia coli* strain PM22 plasmid pYLPM22a, complete sequence | *Escherichia coli* | 85488 | 1.99E+05 | 66% | 0 | 99.56 | 140816 | 120320 | CP074020.1 |
|  | *Escherichia coli* strain 537-20 plasmid p537-20_1, complete sequence | *Escherichia coli* | 82646 | 1.89E+05 | 68% | 0 | 99.6 | 140816 | 126021 | CP091535.1 |
|  | *Escherichia coli* strain XH993 plasmid pXH993, complete sequence | *Escherichia coli* | 81222 | 2.59E+05 | 68% | 0 | 99.79 | 140816 | 349248 | CP019360.1 |
|  | *Escherichia coli* strain ECJXMCRH7 plasmid pTBH7P2, complete sequence | *Escherichia coli* | 81201 | 2.34E+05 | 70% | 0 | 99.78 | 140816 | 130221 | CP067344.1 |
|  | *Escherichia coli* strain RHB41-C22 plasmid pRHB41-C22_2, complete sequence | *Escherichia coli* | 80492 | 1.91E+05 | 69% | 0 | 99.36 | 140816 | 150657 | CP056956.1 |
|  | *Escherichia coli* strain 258E plasmid pEC258-1, complete sequence | *Escherichia coli* | 79835 | 1.04E+05 | 37% | 0 | 99.73 | 140816 | 50185 | CP097096.1 |
|  | *Escherichia coli* strain TREC1 plasmid pTREC1, complete sequence | *Escherichia coli* | 79320 | 2.09E+05 | 63% | 0 | 99.55 | 140816 | 128358 | MN158989.1 |
|  | *Escherichia coli* strain 13KWH46 plasmid p13KWH46-1, complete sequence | *Escherichia coli* | 78633 | 2.31E+05 | 77% | 0 | 99.81 | 140816 | 162357 | CP019251.1 |
|  | *Escherichia coli* strain RH-024-WU chromosome | *Escherichia coli* | 77554 | 2.45E+05 | 82% | 0 | 99.71 | 140816 | 6442898 | CP050201.1 |
|  | *Escherichia coli* strain p10A plasmid p10A_p1, complete sequence | *Escherichia coli* | 76967 | 2.16E+05 | 64% | 0 | 99.67 | 140816 | 143163 | CP049082.1 |
|  | *Escherichia coli* strain EC4 plasmid pEC4-2, complete sequence | *Escherichia coli* | 76661 | 2.03E+05 | 61% | 0 | 99.75 | 140816 | 112054 | CP060975.1 |
|  | *Escherichia coli* strain EC014 plasmid pEC014-2, complete sequence | *Escherichia coli* | 76373 | 2.51E+05 | 68% | 0 | 99.9 | 140816 | 154516 | MW317021.1 |
|  | *Escherichia coli* THO-010 plasmid pTHO-010-3 DNA, complete sequence | *Escherichia coli* | 75915 | 2.03E+05 | 62% | 0 | 99.77 | 140816 | 111439 | AP022543.1 |
|  | *Escherichia coli* strain 13C1079T plasmid p13C1079T-1, complete sequence | *Escherichia coli* | 75582 | 1.77E+05 | 55% | 0 | 99.85 | 140816 | 125272 | CP019268.1 |
|  | *Escherichia coli* strain T28R plasmid pT28R-2, complete sequence | *Escherichia coli* | 74978 | 2.80E+05 | 85% | 0 | 99.76 | 140816 | 161057 | CP049355.1 |
|  | *Escherichia coli* strain EC7 plasmid pEC7-2, complete sequence | *Escherichia coli* | 74759 | 2.20E+05 | 65% | 0 | 99.77 | 140816 | 128054 | CP060964.1 |
|  | *Escherichia coli* strain LP50-1 plasmid pLP50-1-101kb, complete sequence | *Escherichia coli* | 73796 | 2.14E+05 | 61% | 0 | 99.65 | 140816 | 101973 | CP101859.1 |
|  | *Escherichia coli* plasmid pCERC3, complete sequence | *Escherichia coli* | 73791 | 2.19E+05 | 74% | 0 | 99.7 | 140816 | 144344 | KR827684.1 |
|  | *Escherichia coli* strain A241 plasmid pA241-TEM, complete sequence | *Escherichia coli* | 73713 | 2.16E+05 | 75% | 0 | 99.66 | 140816 | 144685 | MN807689.1 |
|  | *Escherichia coli* strain 1-80206 plasmid p1-80206, complete sequence | *Escherichia coli* | 72810 | 2.14E+05 | 66% | 0 | 99.63 | 140816 | 139086 | MW633522.1 |
|  | *Escherichia coli* strain ESBL3277 plasmid pESBL3277-IncF, complete sequence | *Escherichia coli* | 72759 | 2.14E+05 | 66% | 0 | 99.69 | 140816 | 133148 | MW390539.1 |
|  | *Escherichia coli* plasmid Carbapenemase(NDM-4)_IncFIB, complete sequence | *Escherichia coli* | 72411 | 1.91E+05 | 60% | 0 | 99.74 | 140816 | 150273 | CP050167.1 |
|  | *Escherichia coli* strain Ec40743 plasmid unnamed1, complete sequence | *Escherichia coli* | 72013 | 2.65E+05 | 84% | 0 | 99.94 | 140816 | 122471 | CP041920.1 |
|  | *Escherichia coli* strain A130 plasmid pA130-TEM, complete sequence | *Escherichia coli* | 71869 | 2.43E+05 | 84% | 0 | 99.88 | 140816 | 122510 | MN816372.1 |
|  | *Escherichia coli* strain TH9F11 plasmid pHNTH9F11-1, complete sequence | *Escherichia coli* | 71492 | 2.87E+05 | 74% | 0 | 99.94 | 140816 | 407456 | CP054192.1 |
|  | *Escherichia coli* plasmid pCOV28B clone COV28B_c2 | *Escherichia coli* | 71285 | 74492 | 29% | 0 | 99.39 | 140816 | 87664 | MG649029.1 |
|  | *Escherichia coli* strain SJC33 plasmid pSJC33-1, complete sequence | *Escherichia coli* | 70674 | 2.64E+05 | 71% | 0 | 99.95 | 140816 | 153055 | CP080256.1 |
|  | *Escherichia coli* strain 56.2 plasmid p1, complete sequence | *Escherichia coli* | 70053 | 2.11E+05 | 72% | 0 | 99.92 | 140816 | 133421 | CP059923.1 |
|  | *Escherichia coli* strain T16RC plasmid pT16RC-1, complete sequence | *Escherichia coli* | 69734 | 1.89E+05 | 54% | 0 | 99.98 | 140816 | 108982 | MN848327.1 |
|  | *Escherichia coli* strain T16R plasmid pT16R-2, complete sequence | *Escherichia coli* | 69734 | 2.83E+05 | 88% | 0 | 99.98 | 140816 | 172892 | CP046718.1 |
|  | *Escherichia coli* strain EC11 plasmid plas2, complete sequence | *Escherichia coli* | 69641 | 2.28E+05 | 74% | 0 | 99.71 | 140816 | 134098 | CP073362.1 |
|  | *Escherichia coli* strain 14406 plasmid p14406-FII, complete sequence | *Escherichia coli* | 69461 | 2.92E+05 | 84% | 0 | 99.74 | 140816 | 178333 | MN823988.1 |
|  | *Escherichia coli* O126:H45 strain MIN10 plasmid pMUB-MIN10-1, complete sequence | *Escherichia coli* O126:H45 | 69372 | 2.16E+05 | 78% | 0 | 99.62 | 140816 | 146908 | CP069678.1 |
|  | *Escherichia coli* isolate 162 genome assembly, plasmid: P1 | *Escherichia coli* | 69364 | 2.20E+05 | 78% | 0 | 99.61 | 140816 | 145050 | OW849378.1 |
|  | *Escherichia coli* strain FORC_082 plasmid pFORC82_2, complete sequence | *Escherichia coli* | 68589 | 1.35E+05 | 50% | 0 | 99.74 | 140816 | 101404 | CP026643.1 |
|  | Shigella flexneri strain STEFF_18 plasmid unnamed1, complete sequence | Shigella flexneri | 67516 | 2.15E+05 | 68% | 0 | 99.67 | 140816 | 122007 | CP055180.1 |
|  | Shigella flexneri strain STEFF_24 plasmid unnamed1, complete sequence | Shigella flexneri | 67516 | 2.25E+05 | 67% | 0 | 99.67 | 140816 | 125110 | CP055168.1 |
|  | *Escherichia coli* strain SCZE5 plasmid pSCZE2 | *Escherichia coli* | 66486 | 2.42E+05 | 69% | 0 | 99.83 | 140816 | 143259 | CP051224.1 |
| pCR-hvKP221-KPC-P3 | *Klebsiella pneumoniae* strain JNKPN26 plasmid pJNKPN26_KPC, complete sequence | *Klebsiella pneumoniae* | 1.02E+05 | 3.33E+05 | 85% | 0 | 99.96 | 136943 | 126203 | CP090204.1 |
|  | *Klebsiella pneumoniae* strain C789 plasmid pKPC-CR-hvKP-C789, complete sequence | *Klebsiella pneumoniae* | 92636 | 3.51E+05 | 86% | 0 | 99.96 | 136943 | 128299 | CP034417.1 |
|  | *Klebsiella pneumoniae* strain JNKPN26 plasmid pJNKPN26_KPC, complete sequence | *Klebsiella pneumoniae* | 92636 | 3.35E+05 | 85% | 0 | 99.96 | 136943 | 126203 | MZ546615.1 |
|  | *Klebsiella pneumoniae* strain 50700 plasmid p50700-140.6, complete sequence | *Klebsiella pneumoniae* | 92636 | 3.44E+05 | 91% | 0 | 99.96 | 136943 | 140550 | CP088995.1 |
|  | *Klebsiella pneumoniae* strain CRKP78R plasmid p3, complete sequence | *Klebsiella pneumoniae* | 92636 | 4.04E+05 | 95% | 0 | 99.96 | 136943 | 149407 | CP066256.1 |
|  | *Klebsiella pneumoniae* strain KP137060 plasmid unnamed2, complete sequence | *Klebsiella pneumoniae* | 92610 | 3.37E+05 | 94% | 0 | 99.95 | 136943 | 146878 | MW218143.1 |
|  | *Klebsiella pneumoniae* strain QL24 plasmid pKPC-QL24, complete sequence | *Klebsiella pneumoniae* | 91587 | 3.11E+05 | 85% | 0 | 99.95 | 136943 | 126126 | MH263653.1 |
|  | *Klebsiella pneumoniae* strain 20049 plasmid p20049-KPC, complete sequence | *Klebsiella pneumoniae* | 91404 | 3.95E+05 | 98% | 0 | 99.9 | 136943 | 151653 | MF168404.1 |
|  | *Klebsiella pneumoniae* strain Kp36 plasmid unnamed2, complete sequence | *Klebsiella pneumoniae* | 88734 | 3.94E+05 | 95% | 0 | 100 | 136943 | 142228 | CP047194.1 |
|  | *Klebsiella pneumoniae* *subsp.* pneumoniae strain SCKP020079 plasmid pKPC2_020079, complete sequence | *Klebsiella pneumoniae* *subsp.* pneumoniae | 88734 | 3.74E+05 | 94% | 0 | 100 | 136943 | 146790 | CP029381.1 |
|  | *Klebsiella pneumoniae* strain 33367 plasmid p33367_KPC2, complete sequence | *Klebsiella pneumoniae* | 88734 | 3.97E+05 | 95% | 0 | 100 | 136943 | 150096 | CP099415.1 |
|  | *Klebsiella pneumoniae* strain 7849 plasmid pKP7849_KPC, complete sequence | *Klebsiella pneumoniae* | 88734 | 3.96E+05 | 95% | 0 | 100 | 136943 | 148977 | MW478298.1 |
|  | *Klebsiella pneumoniae* strain 2014042281 plasmid p42281-KPC, complete sequence | *Klebsiella pneumoniae* | 88734 | 2.73E+05 | 64% | 0 | 100 | 136943 | 115305 | MT810369.1 |
|  | *Klebsiella pneumoniae* strain 911021 plasmid p911021-KPC, complete sequence | *Klebsiella pneumoniae* | 88728 | 4.04E+05 | 91% | 0 | 100 | 136943 | 169824 | MK036888.1 |
|  | *Klebsiella pneumoniae* strain WCHKP115069 plasmid pKPC2_115069, complete sequence | *Klebsiella pneumoniae* | 88728 | 3.54E+05 | 88% | 0 | 100 | 136943 | 154986 | CP033404.1 |
|  | *Klebsiella pneumoniae* strain FDAARGOS_444 plasmid unnamed2 | *Klebsiella pneumoniae* | 88728 | 4.04E+05 | 88% | 0 | 100 | 136943 | 187926 | CP023942.1 |
|  | *Klebsiella pneumoniae* strain IR5077_1 plasmid unnamed3, complete sequence | *Klebsiella pneumoniae* | 88728 | 2.62E+05 | 58% | 0 | 100 | 136943 | 292919 | CP097673.1 |
|  | *Klebsiella pneumoniae* *subsp.* pneumoniae strain DD01754 plasmid pDD01754-2, complete sequence | *Klebsiella pneumoniae* *subsp.* pneumoniae | 88728 | 4.03E+05 | 89% | 0 | 100 | 136943 | 182556 | CP087647.1 |
|  | *Klebsiella pneumoniae* *subsp.* pneumoniae strain DD01304 plasmid pDD01304-2, complete sequence | *Klebsiella pneumoniae* *subsp.* pneumoniae | 88728 | 3.62E+05 | 89% | 0 | 100 | 136943 | 169136 | CP087608.1 |
|  | *Klebsiella pneumoniae* strain 246421 plasmid p246421-KPC, complete sequence | *Klebsiella pneumoniae* | 88728 | 3.41E+05 | 77% | 0 | 100 | 136943 | 145209 | MT810356.1 |
|  | *Klebsiella pneumoniae* strain A1708 plasmid pA1708-KPC, complete sequence | *Klebsiella pneumoniae* | 88728 | 4.25E+05 | 92% | 0 | 100 | 136943 | 173280 | MT810354.1 |
|  | *Klebsiella pneumoniae* strain F726925 plasmid pF726925-1, complete sequence | *Klebsiella pneumoniae* | 88728 | 4.06E+05 | 89% | 0 | 100 | 136943 | 172862 | CP081821.1 |
|  | *Klebsiella pneumoniae* strain JX-CR-hvKP-2 plasmid pJX2-2, complete sequence | *Klebsiella pneumoniae* | 88728 | 3.84E+05 | 89% | 0 | 100 | 136943 | 168025 | CP064248.1 |
|  | *Klebsiella pneumoniae* *subsp.* pneumoniae strain SH2 plasmid pSH2-85K-MDR, complete sequence | *Klebsiella pneumoniae* *subsp.* pneumoniae | 88728 | 3.84E+05 | 95% | 0 | 100 | 136943 | 149033 | MH643792.1 |
|  | *Klebsiella pneumoniae* *subsp.* pneumoniae strain HA2 plasmid pHA2-23-KPC, complete sequence | *Klebsiella pneumoniae* *subsp.* pneumoniae | 88728 | 3.82E+05 | 95% | 0 | 100 | 136943 | 148749 | MH643789.1 |
|  | *Klebsiella pneumoniae* strain KP18-2079 plasmid pKP18-2079_KPC, complete sequence | *Klebsiella pneumoniae* | 88727 | 1.89E+05 | 57% | 0 | 100 | 136943 | 186564 | MT090959.1 |
|  | *Klebsiella pneumoniae* strain BSI074 plasmid pBSI074-KPC2 | *Klebsiella pneumoniae* | 88727 | 3.41E+05 | 91% | 0 | 100 | 136943 | 135737 | MT269848.1 |
|  | *Klebsiella pneumoniae* strain WCHKP020037 plasmid pKPC2_020037, complete sequence | *Klebsiella pneumoniae* | 88723 | 4.16E+05 | 89% | 0 | 99.99 | 136943 | 172770 | CP036372.1 |
|  | *Klebsiella pneumoniae* strain F1 plasmid pF1_1, complete sequence | *Klebsiella pneumoniae* | 88723 | 3.88E+05 | 93% | 0 | 99.99 | 136943 | 164510 | CP026131.1 |
|  | *Klebsiella pneumoniae* strain L388 plasmid pKPC-L388 | *Klebsiella pneumoniae* | 88723 | 3.49E+05 | 94% | 0 | 99.99 | 136943 | 145851 | CP029225.1 |
|  | *Klebsiella pneumoniae* strain 675920 plasmid p675920-1, complete sequence | *Klebsiella pneumoniae* | 88723 | 3.68E+05 | 88% | 0 | 99.99 | 136943 | 163995 | MF133495.1 |
|  | *Klebsiella pneumoniae* strain SH12 plasmid pSH12_KPC, complete sequence | *Klebsiella pneumoniae* | 88723 | 3.95E+05 | 88% | 0 | 99.99 | 136943 | 167468 | CP040835.1 |
|  | *Klebsiella pneumoniae* strain BSI054 plasmid pBSI054-KPC2 | *Klebsiella pneumoniae* | 88723 | 4.00E+05 | 89% | 0 | 99.99 | 136943 | 168038 | MT269833.1 |
|  | *Klebsiella pneumoniae* strain BSI052 plasmid pBSI052-KPC2 | *Klebsiella pneumoniae* | 88723 | 3.86E+05 | 89% | 0 | 99.99 | 136943 | 165819 | MT269832.1 |
|  | *Klebsiella pneumoniae* strain KPN361 plasmid pKPN361-1, complete sequence | *Klebsiella pneumoniae* | 88723 | 4.01E+05 | 91% | 0 | 99.99 | 136943 | 169824 | CP053017.1 |
|  | *Klebsiella pneumoniae* strain SWU01 plasmid unnamed, complete sequence | *Klebsiella pneumoniae* | 88721 | 3.86E+05 | 92% | 0 | 99.99 | 136943 | 162552 | CP018455.1 |
|  | Enterobacter cloacae strain CBG15936 plasmid pTEM-CBG, complete sequence | Enterobacter cloacae | 88717 | 1.67E+05 | 55% | 0 | 99.99 | 136943 | 75044 | CP046117.1 |
|  | *Escherichia coli* strain HZMPC32 plasmid pHNMPC32, complete sequence | *Escherichia coli* | 88717 | 1.63E+05 | 53% | 0 | 99.99 | 136943 | 74768 | MG197499.1 |
|  | *Klebsiella pneumoniae* strain WCHKP2 plasmid pKPC2_020002, complete sequence | *Klebsiella pneumoniae* | 88717 | 4.19E+05 | 89% | 0 | 99.99 | 136943 | 177516 | CP028541.2 |
|  | *Klebsiella pneumoniae* strain 381810-51 plasmid p181051-KPC, complete sequence | *Klebsiella pneumoniae* | 88717 | 2.38E+05 | 65% | 0 | 99.99 | 136943 | 303071 | MT920903.1 |
|  | *Klebsiella pneumoniae* strain HZMPC51-2 plasmid pHNMPC51, complete sequence | *Klebsiella pneumoniae* | 88716 | 1.54E+05 | 49% | 0 | 99.99 | 136943 | 69654 | MG197500.1 |
|  | *Klebsiella pneumoniae* strain IR12197_1 plasmid unnamed4, complete sequence | *Klebsiella pneumoniae* | 88716 | 2.78E+05 | 79% | 0 | 99.99 | 136943 | 159393 | CP097707.1 |
|  | *Klebsiella pneumoniae* *subsp.* pneumoniae strain SH9 plasmid pSH9-CTX-TEM, complete sequence | *Klebsiella pneumoniae* *subsp.* pneumoniae | 88712 | 2.01E+05 | 52% | 0 | 99.99 | 136943 | 98684 | MH255829.1 |
|  | *Escherichia coli* strain HNEC55 plasmid pHNEC55, complete sequence | *Escherichia coli* | 88712 | 1.80E+05 | 55% | 0 | 99.99 | 136943 | 81498 | KT879914.1 |
|  | *Klebsiella pneumoniae* strain 49088 plasmid p49088-279.2, complete sequence | *Klebsiella pneumoniae* | 88712 | 3.98E+05 | 88% | 0 | 99.99 | 136943 | 279210 | CP089000.1 |
|  | *Klebsiella pneumoniae* strain KP1880 plasmid pKPC1880, complete sequence | *Klebsiella pneumoniae* | 88712 | 3.91E+05 | 90% | 0 | 99.99 | 136943 | 168960 | CP061347.1 |
|  | *Klebsiella pneumoniae* *subsp.* pneumoniae strain WCHKP015093 plasmid pKPC2_015093, complete sequence | *Klebsiella pneumoniae* *subsp.* pneumoniae | 88701 | 3.63E+05 | 88% | 0 | 99.98 | 136943 | 154724 | CP036301.1 |
|  | *Klebsiella pneumoniae* strain WCHKP649 plasmid pKPC2_095649, complete sequence | *Klebsiella pneumoniae* | 88701 | 3.66E+05 | 88% | 0 | 99.98 | 136943 | 156099 | CP026584.1 |
|  | *Klebsiella pneumoniae* strain HvKp-su1 plasmid unnamed1, complete sequence | *Klebsiella pneumoniae* | 88701 | 3.51E+05 | 93% | 0 | 99.98 | 136943 | 133346 | CP092718.1 |
|  | *Klebsiella pneumoniae* strain 140253 plasmid pKPC2_140253, complete sequence | *Klebsiella pneumoniae* | 88701 | 3.40E+05 | 88% | 0 | 99.98 | 136943 | 155793 | CP097627.1 |
|  | *Klebsiella pneumoniae* strain WCHKP115038 plasmid pKPC2_115038, complete sequence | *Klebsiella pneumoniae* | 88701 | 3.40E+05 | 87% | 0 | 99.98 | 136943 | 153486 | CP043603.1 |
|  | *Klebsiella pneumoniae* strain WCHKP090050 plasmid pKPC2_090050, complete sequence | *Klebsiella pneumoniae* | 88701 | 3.42E+05 | 88% | 0 | 99.98 | 136943 | 154724 | CP043370.1 |
|  | *Klebsiella pneumoniae* strain WCHKP090045 plasmid pKPC2_090045, complete sequence | *Klebsiella pneumoniae* | 88701 | 3.42E+05 | 88% | 0 | 99.98 | 136943 | 154724 | CP043366.1 |
|  | *Klebsiella pneumoniae* strain WCHKP115011 plasmid pKPC2_115011, complete sequence | *Klebsiella pneumoniae* | 88701 | 4.07E+05 | 88% | 0 | 99.98 | 136943 | 157388 | CP089954.1 |
|  | *Klebsiella pneumoniae* strain WCHKP090374 plasmid pKPC2_090374, complete sequence | *Klebsiella pneumoniae* | 88701 | 3.63E+05 | 88% | 0 | 99.98 | 136943 | 154728 | CP066536.1 |
|  | *Klebsiella pneumoniae* *subsp.* pneumoniae strain WCHKP020120 plasmid pKPC2_020120, complete sequence | *Klebsiella pneumoniae* *subsp.* pneumoniae | 88701 | 3.54E+05 | 88% | 0 | 99.98 | 136943 | 154719 | CP043358.1 |
|  | *Klebsiella pneumoniae* strain 9489 plasmid pBJ9489-KPC | *Klebsiella pneumoniae* | 88701 | 3.27E+05 | 92% | 0 | 99.98 | 136943 | 132246 | MN821371.1 |
|  | *Klebsiella pneumoniae* strain C2660 plasmid pC2660-3-KPC, complete sequence | *Klebsiella pneumoniae* | 88695 | 3.29E+05 | 88% | 0 | 99.98 | 136943 | 153556 | CP039810.1 |
|  | *Escherichia coli* strain HNC02 plasmid pHNHNC02, complete sequence | *Escherichia coli* | 88695 | 1.79E+05 | 54% | 0 | 99.98 | 136943 | 76869 | MG197497.1 |
|  | *Klebsiella pneumoniae* strain XHKPN083 plasmid pXHKPN083-2, complete sequence | *Klebsiella pneumoniae* | 88695 | 2.95E+05 | 61% | 0 | 99.98 | 136943 | 122553 | CP066912.1 |
|  | Citrobacter werkmanii strain LYYSPS2 plasmid pLYYSPS2-3, complete sequence | Citrobacter werkmanii | 88695 | 1.80E+05 | 55% | 0 | 99.98 | 136943 | 87232 | MZ342958.1 |
|  | *Klebsiella pneumoniae* strain 150040X1B1 plasmid pCTXM65_150040X1B1, complete sequence | *Klebsiella pneumoniae* | 88693 | 3.38E+05 | 82% | 0 | 99.98 | 136943 | 149214 | CP101727.1 |
|  | *Klebsiella pneumoniae* strain WCHKP090357 plasmid pKPC2_090357, complete sequence | *Klebsiella pneumoniae* | 88693 | 3.39E+05 | 85% | 0 | 99.98 | 136943 | 149116 | CP066524.1 |
|  | *Klebsiella pneumoniae* p477Kp plasmid, complete sequence | *Klebsiella pneumoniae* | 88690 | 1.66E+05 | 55% | 0 | 99.98 | 136943 | 74768 | LN897475.2 |
|  | *Klebsiella pneumoniae* p397Kp plasmid, complete sequence | *Klebsiella pneumoniae* | 88690 | 1.83E+05 | 56% | 0 | 99.98 | 136943 | 76863 | LN897474.2 |
|  | *Klebsiella pneumoniae* strain JX-CR-hvKP-1 plasmid pJX1-1, complete sequence | *Klebsiella pneumoniae* | 88690 | 3.84E+05 | 89% | 0 | 99.98 | 136943 | 167834 | CP064253.1 |
|  | *Klebsiella pneumoniae* strain 08291 plasmid pW08291-KPC, complete sequence | *Klebsiella pneumoniae* | 88684 | 3.85E+05 | 90% | 0 | 99.98 | 136943 | 169804 | MN842295.1 |
|  | *Klebsiella pneumoniae* strain CRKP-30 plasmid pCRKP-30_KPC, complete sequence |  | 88684 | 3.81E+05 | 88% | 0 | 99.98 | 136943 | 158524 | CP102634.1 |
|  | *Klebsiella pneumoniae* *subsp.* pneumoniae strain RJBSI76-pV plasmid pRJBSI76-pV-3, complete sequence | *Klebsiella pneumoniae* *subsp.* pneumoniae | 88684 | 3.52E+05 | 88% | 0 | 99.98 | 136943 | 184751 | CP068687.1 |
|  | *Klebsiella pneumoniae* *subsp.* pneumoniae strain RJBSI76 plasmid pRJBSI76-3, complete sequence | *Klebsiella pneumoniae* *subsp.* pneumoniae | 88684 | 3.52E+05 | 88% | 0 | 99.98 | 136943 | 184748 | CP068692.1 |
|  | *Escherichia coli* strain 7A8 plasmid pHN7A8, complete sequence | *Escherichia coli* | 88684 | 1.82E+05 | 56% | 0 | 99.98 | 136943 | 76878 | JN232517.1 |
|  | *Klebsiella pneumoniae* strain CDI694 plasmid pCDI694-140.8, complete sequence | *Klebsiella pneumoniae* | 88682 | 3.48E+05 | 91% | 0 | 99.98 | 136943 | 140828 | CP077777.1 |
|  | *Escherichia coli* strain OW1E2 plasmid pOW1E2a, complete sequence | *Escherichia coli* | 88682 | 2.12E+05 | 54% | 0 | 99.98 | 136943 | 108766 | CP067246.1 |
|  | *Klebsiella pneumoniae* strain 8695 plasmid pFK8695-KPC-33, complete sequence | *Klebsiella pneumoniae* | 88680 | 3.79E+05 | 91% | 0 | 99.97 | 136943 | 143980 | CP085890.1 |
|  | *Escherichia coli* strain fEC.1 plasmid pfEC.1-3, complete sequence | *Escherichia coli* | 88677 | 1.71E+05 | 51% | 0 | 99.98 | 136943 | 78319 | OK605583.1 |
|  | *Escherichia coli* strain NT1N25 plasmid pNT1N25-76kb, complete sequence | *Escherichia coli* | 88671 | 1.84E+05 | 51% | 0 | 99.97 | 136943 | 76891 | CP075483.1 |
|  | *Klebsiella pneumoniae* strain HZMPC43 plasmid pHNMPC43, complete sequence | *Klebsiella pneumoniae* | 88666 | 1.54E+05 | 49% | 0 | 99.97 | 136943 | 69666 | MG197501.1 |
|  | *Klebsiella pneumoniae* strain 12139 plasmid p12139-KPC, complete sequence | *Klebsiella pneumoniae* | 88642 | 3.95E+05 | 91% | 0 | 99.96 | 136943 | 169424 | MF168403.1 |
|  | *Klebsiella pneumoniae* strain BSI014 plasmid pBSI014-KPC2 | *Klebsiella pneumoniae* | 88610 | 4.06E+05 | 88% | 0 | 99.94 | 136943 | 170701 | MT269822.1 |
|  | *Klebsiella pneumoniae* strain 64917 plasmid p64917-KPC, complete sequence | *Klebsiella pneumoniae* | 88592 | 3.94E+05 | 91% | 0 | 99.94 | 136943 | 169419 | MF168405.1 |
|  | Escherichia fergusonii strain EFCF056 plasmid pEF02, complete sequence | Escherichia fergusonii | 88584 | 2.04E+05 | 54% | 0 | 99.94 | 136943 | 90871 | CP040807.1 |
|  | *Escherichia coli* strain XD35 plasmid pXD35004, complete sequence | *Escherichia coli* | 88359 | 1.85E+05 | 57% | 0 | 99.86 | 136943 | 85891 | CP089137.1 |
|  | *Klebsiella pneumoniae* strain 116753 plasmid p116753-KPC, complete sequence | *Klebsiella pneumoniae* | 87713 | 2.17E+05 | 53% | 0 | 99.61 | 136943 | 137873 | MN891682.1 |
|  | *Klebsiella pneumoniae* strain KP19-3138 plasmid pKP19-3138-4, complete sequence | *Klebsiella pneumoniae* | 87707 | 2.14E+05 | 52% | 0 | 99.61 | 136943 | 95171 | CP090620.1 |
|  | *Escherichia coli* strain HNEC46 plasmid PHNEC46, complete sequence | *Escherichia coli* | 87284 | 1.29E+05 | 51% | 0 | 99.99 | 136943 | 74046 | KX503323.1 |
|  | *Klebsiella pneumoniae* strain 135077 plasmid p1_135077, complete sequence | *Klebsiella pneumoniae* | 87190 | 3.08E+05 | 82% | 0 | 99.99 | 136943 | 139526 | CP073293.1 |
|  | *Klebsiella pneumoniae* strain FRPDR plasmid pFRPDR_KPC, complete sequence | *Klebsiella pneumoniae* | 84804 | 2.69E+05 | 65% | 0 | 99.99 | 136943 | 107554 | CP063761.1 |
|  | *Klebsiella pneumoniae* strain C2244 plasmid pCRHV-C2244, complete sequence | *Klebsiella pneumoniae* | 84753 | 2.36E+05 | 52% | 0 | 99.96 | 136943 | 293391 | MT644086.1 |
|  | *Klebsiella pneumoniae* strain 12085 plasmid p12085-KPC, complete sequence | *Klebsiella pneumoniae* | 84228 | 3.60E+05 | 74% | 0 | 99.95 | 136943 | 142041 | MN842292.1 |
|  | *Klebsiella pneumoniae* strain A1750 plasmid pA1750-KPC, complete sequence | *Klebsiella pneumoniae* | 84226 | 2.58E+05 | 59% | 0 | 99.95 | 136943 | 111536 | MT108207.1 |
|  | *Klebsiella pneumoniae* strain XHKP6 plasmid pXHKP6-1, complete sequence | *Klebsiella pneumoniae* | 84226 | 4.38E+05 | 95% | 0 | 99.95 | 136943 | 180300 | CP066888.1 |
|  | *Klebsiella pneumoniae* strain XHKP75 plasmid pXHKP75-2, complete sequence | *Klebsiella pneumoniae* | 84221 | 3.69E+05 | 88% | 0 | 99.95 | 136943 | 163729 | CP066897.1 |
|  | *Klebsiella pneumoniae* strain XHKP309 plasmid pXHKP309-1, complete sequence | *Klebsiella pneumoniae* | 84221 | 4.47E+05 | 88% | 0 | 99.95 | 136943 | 178237 | CP066901.1 |
|  | *Klebsiella pneumoniae* strain 20150420184 plasmid p420184-KPC, complete sequence | *Klebsiella pneumoniae* | 84188 | 3.06E+05 | 76% | 0 | 99.94 | 136943 | 108236 | MT810359.1 |
|  | *Klebsiella pneumoniae* strain WCHKP3 plasmid pKPC2_020003, complete sequence | *Klebsiella pneumoniae* | 84182 | 3.26E+05 | 88% | 0 | 99.94 | 136943 | 154957 | CP031720.1 |
[truncated: 50,892 more chars]
