# Supplementary material for: Phenotypic and genomic characterization of ST11-K1 CR-hvKP with highly homologous blaKPC-2-bearing plasmids in China
Source: mSystems. 2024 Nov 18;9(12):e01101-24. doi: 10.1128/msystems.01101-24 (PMC11651102; doi:10.1128/msystems.01101-24)
Supplement: Table S3 — The function of resistance genes and virulence genes detected in CR-hvKP221, CR-hvKP005, CR-hvKP006, CR-hvKP26, CR-hvKP128, CR-hvKP132, and CR-hvKP173 strains. [file msystems.01101-24-s0004.docx]

**Table S3-1** The function of resistance genes detected in CR-hvKP221, CR-hvKP005, CR-hvKP006, CR-hvKP26, CR-hvKP128, CR-hvKP132, and CR-hvKP173 stains.

|  | Gene | Gene_accession no. | | Class | | Phenotype | | | PMID | Mechanism of resistance |
| --- | --- | --- | --- | --- | --- | --- | --- | --- | --- | --- |
|  | *OqxB* | OqxB_1_EU370913 | | Amphenicol, Quinolone, Quaternary, Ammonium Compounds, Folate pathway antagonist | | Chloramphenicol, Benzylkonium Chloride, Cetylpyridinium Chloride, Nalidixic acid, Ciprofloxacin, Trimethoprim | | | 18440636 | Efflux pump Must be in an operon with oqxA,phenotype differs based on genomic location of the operon PMID 25801572,also nitrofurantoin resistance PMID 26552976 |
|  | *otr(B)* | otr(B)_1_AF079900 | | Tetracycline | | Doxycycline, Tetracycline | | | 9867793 | Increased efflux |
|  | *optrA* | optrA_14_KX620941 | | Oxazolidinone, Amphenicol | | Linezolid, Chloramphenicol, Florfenicol | | | Unpublished, 27006457 | Target protection |
|  | *tet(T)* | tet(T)_1_L42544 | | Tetracycline | | Doxycycline, Tetracycline, Minocycline | | | 8980765 | Target protection |
|  | *sul4* | sul4_1_MG649393 | | Folate pathway antagonist | | Sulfamethoxazole | | | 29246178 | Purine synthesis |
|  | *OqxA* | OqxA_1_EU370913 | | Amphenicol, Quinolone, Quaternary Ammonium Compounds, Folate pathway antagonist | | Chloramphenicol, Benzylkonium Chloride, Cetylpyridinium Chloride, Nalidixic acid, Ciprofloxacin, Trimethoprim | | | 18440636 | Efflux pump |
|  | *ClpL* | ClpL_1_CP023753 | | Heat | | Temperature | | | 29104933 | ATP-dependant proteas |
|  | *penA* | penA_1_AF515059 | | Beta-lactam | | Unknown Beta-lactam | | | unpublished | Enzymatic inactivation |
|  | *cmlV* | cmlV_1_U09991 | | Amphenicol | | Chloramphenicol | | | 7592948 | Increased efflux |
|  | *formA* | formA_1_X73835 | | Aldehyde | | Formaldehyde | | | 8891129 | Enzymatic degradation |
|  | *qepA4* | qepA4_1_KX580704 | | Quinolone | | Ciprofloxacin | | | 29075330 | Increased efflux |
|  | *bla*_SHV-11_ | blaSHV-11_1_X98101 | | Beta-lactam | | Amoxicillin, Ampicillin, Cephalothin, Piperacillin, Ticarcillin | | | 9145849 | Enzymatic inactivation |
|  | *poxtA* | poxtA_1_MF095097 | | Amphenicol, Oxazolidinone, Tetracycline | | Chloramphenicol, Florfenicol, Linezolid, Doxycycline, Tetracycline | | | 27073268 | Target protection |
|  | *tetB(60)* | tetB(60)_1_KX000273 | | Tetracycline | | Doxycycline, Tetracycline, Tigecycline | | | unpublished | Increased efflux |
|  | *mcr-8* | mcr-8_1_MG736312 | | Polymyxin | | Colistin | | | 29970891 | Target modification |
|  | *tetB(46）* | tetB(46)_1_HQ652506 | | Tetracycline | | Doxycycline, Tetracycline, Tigecycline | | | 22941900 | Increased efflux |
|  | *tetA(60)* | tetA(60)_1_KX000272 | | Tetracycline | | Doxycycline, Tetracycline, Tigecycline | | | unpublished | Increased efflux |
|  | *VanA* | VanA_bc_1_Y15704 | | Glycopeptide | | Vancomycin, Teicoplanin | | | 9687406 | Target modification |
|  | *tet(34)* | tet(34)_1_AB061440 | | Tetracycline | | Tetracycline | | | 11959596 | Enzymatic inactivation |
|  | *fosA* | fosA_6_ACZD01000244 | | Fosfomycin | | Fosfomycin | | | unpublished | Enzymatic inactivation |
|  | *mupB* | mupB_1_JQ231224 | | Pseudomonic acid | | Mupirocin | | | 22252810 | Isoleucyl tRNA synthetase |
|  | *bla*_KPC-2_ | blaKPC-2_1_AY034847 | | Beta-lactam | | Amoxicillin, Amoxicillin+Clavulanic acid, Ampicillin, Ampicillin+Clavulanic acid, Aztreonam, Cefepime, Cefotaxime, Cefoxitin, Ceftazidime, Ertapenem, Imipenem, Meropenem, Piperacillin, Piperacillin+Tazobactam, Ticarcillin, Ticarcillin+Clavulanic acid | | | 12615876, 11257029 | Enzymatic inactivation |
|  | *bla*_SHV-12_ | blaSHV-12_1_KF976405 | | Beta-lactam | | Amoxicillin, Ampicillin, Aztreonam, Cefepime, Cefotaxime, Ceftazidime, Ceftriaxone, Piperacillin, Ticarcillin | | | 26038408, 16678922 | Enzymatic inactivation |
|  | *bla*_CTX-M-65_ | blaCTX-M-65_1_EF418608 | | Beta-lactam | | Amoxicillin, Ampicillin, Aztreonam, Cefepime, Cefotaxime, Ceftazidime, Ceftriaxone, Piperacillin, Ticarcillin | | | Unpublished | Enzymatic inactivation |
|  | *poxtA_1* | poxtA_1_MF095097 | | Amphenicol, Oxazolidinone, Tetracycline | | Chloramphenicol, Florfenicol, Linezolid, Doxycycline, Tetracycline | | | 27073268 | Target protection |
|  | *tetB(46)* | tetB(46)_1_HQ652506 | | Tetracycline | | Doxycycline, Tetracycline, Tigecycline | | | 22941900 | Increased efflux |
|  | *catA2* | catA2_1_X53796 | | Amphenicol | | Chloramphenicol | | | 2268278 | Enzymatic inactivation |
|  | *sul1* | sul1_26_AY524415 | | Folate pathway antagonist | | Sulfamethoxazole | | | 18753343 | Purine synthesis |
|  | *aadA2* | aadA2_1_NC_010870 | | Aminoglycoside | | Spectinomycin, Streptomycin | | | 17526756 | Enzymatic modification |
|  | *bla*_TEM-1D_ | blaTEM-1D_1_AF188200 | | Beta-lactam | | Amoxicillin, Ampicillin, Cephalothin, Piperacillin, Ticarcillin | | | Unpublished | Enzymatic inactivation |
|  | *rmtB* | rmtB_1_AB103506 | | Aminoglycoside | | Amikacin, Gentamicin, Tobramycin, Kanamycin, Arbekacin, Sisomicin, Isepamicin | | | 14742200 | Target protection |
|  | poxtA_ | poxtA_1_MF095097 | | Amphenicol, Oxazolidinone, Tetracycline | | Chloramphenicol, Florfenicol, Linezolid, Doxycycline, Tetracycline | | | 27073268 | Target protection |
|  | dfrA14 | dfrA14_1_KF921535 | | Folate pathway antagonist | | Trimethoprim | | | 25161650 | Purine synthesis |
|  | *bla*_LAP-2_ | blaLAP-2_1_EU159120 | | Beta-lactam | | Amoxicillin, Ampicillin, Cephalotin, Piperacillin, Ticarcillin | | | 18550213 | Enzymatic inactivation |
|  | *dfrA17* | dfrA17_6_AF180469 | | Folate pathway antagonist | | Trimethoprim | | | unpublished | Purine synthesis |
|  | *aadA5* | aadA5_1_AF137361 | | Aminoglycoside | | Spectinomycin, Streptomycin | | | 10673049 | Enzymatic modification |
|  | *mph(A)* | mph(A)_2_U36578 | | Macrolide | | Erythromycin, Azithromycin, Spiramycin, Telithromycin | | | Unpublished | Enzymatic inactivation |
|  | *sul2* | sul2_18_AJ830714 | | Folate pathway antagonist | | Sulfamethoxazole | | | 22888274 | Purine synthesis |
|  | *aph(3'')-Ib* | aph(3'')-Ib_5_AF321551 | | Aminoglycoside | | Streptomycin | | | 12029529 | Enzymatic modification |
|  | *aph(6)-Id* | aph(6)-Id_1_M28829 | | Aminoglycoside | | Streptomycin | | | 2653965 | Enzymatic modification |
|  | *tet(A)* | tet(A)_6_AF534183 | | Tetracycline | | Doxycycline, Tetracycline | | | unpublished | Increased efflux |
|  | *fosA3* | fosA3_1_AB522970 | | Fosfomycin | | Fosfomycin | | | 20404116 | Enzymatic inactivation |
|  | *rmtB* | rmtB_1_AB103506 | | Aminoglycoside | | Amikacin, Gentamicin, Tobramycin, Kanamycin, Arbekacin, Sisomicin, Isepamicin | | | 14742200 | Target protection |
|  |  | | | | | | | | | |
|  | **Table S3-2** The function of virulence genes detected in CR-hvKP221, CR-hvKP005, CR-hvKP006, CR-hvKP26, CR-hvKP128, CR-hvKP132, and CR-hvKP173 stains. | | | | | | | | | |
| Virulence Factor ID | | | Gene | | Function | | Subtype | type | | |
| VFG013407 | | | *rfaD* | | (rfaD) ADP-L-glycero-D-manno-heptose-6-epimerase [LOS (CVF494)] [Haemophilus influenzae 86-028NP] | | LOS_Endotoxin | Endotoxin | | |
| VFG049144 | | | *acrB* | | (acrB) acriflavine resistance protein B [AcrAB (VF0568)] [Klebsiella pneumoniae subsp. pneumoniae NTUH-K2044] | | AcrAB | Efflux Pump | | |
| VFG013517 | | | *mrsA/glmM* | | (mrsA/glmM) phosphoglucosamine mutase [Exopolysaccharide (CVF495)] [Haemophilus somnus 129PT] | | Exopolysaccharide | Immune Evasion | | |
| VFG048317 | | | *fimK* | | (fimK) transcriptional regulator [Type I fimbriae (VF0566)] [Klebsiella pneumoniae subsp. pneumoniae NTUH-K2044] | | Type_I_fimbriae | Adherence | | |
| VFG048307 | | | *fimH* | | (fimH) type 1 fimbrial adhesin precursor [Type I fimbriae (VF0566)] [Klebsiella pneumoniae subsp. pneumoniae NTUH-K2044] | | Fim | Fimbrial Adherence Determinants | | |
| VFG048298 | | | *fimG* | | (fimG) hypothetical protein [Type I fimbriae (CVF847)] [Klebsiella pneumoniae subsp. rhinoscleromatis SB3432] | | Type_I_fimbriae | Adherence | | |
| VFG048287 | | | *fimF* | | (fimF) type 1 fimbrial minor component [Type I fimbriae (VF0566)] [Klebsiella pneumoniae subsp. pneumoniae NTUH-K2044] | | Type_I_fimbriae | Adherence | | |
| VFG048277 | | | *fimD* | | (fimD) outer membrane usher protein [Type I fimbriae (VF0566)] [Klebsiella pneumoniae subsp. pneumoniae NTUH-K2044] | | Fimbriae | Adherence | | |
| VFG048268 | | | *fimC* | | fimC) hypothetical protein [Type I fimbriae (CVF847)] [Klebsiella pneumoniae subsp. rhinoscleromatis SB3432] | | Fimbriae | Adherence | | |
| VFG048256 | | | *fimI* | | fimI) fimbrial protein [Type I fimbriae (CVF847)] [Klebsiella pneumoniae subsp. pneumoniae MGH 78578] | | Fimbriae | Fimbrial Adherence Determinants | | |
| VFG000477 | | | *rpoS* | | (rpoS) sigma S (sigma 38) factor of RNA polymerase, major sigmafactor during stationary phase [RpoS (VF0112)] [Salmonella enterica subsp. enterica serovar Typhimurium str. LT2] | | Alternative_sigma_factor_RpoS | Regulation | | |
| VFG012582 | | | *sitC* | | (sitC) iron transport protein, inner membrane component [Iron/managanease transport (CVF459)] [Escherichia coli 536] | | Ferrous_iron_transport | Iron Uptake | | |
| VFG013045 | | | *sitB* | | (sitB) Iron transport protein, ATP-binding component [Ferrous iron transport (CVF479)] [Shigella dysenteriae Sd197] | | Ferrous_iron_transport | Iron Uptake | | |
| VFG034194 | | | *sitA* | | (sitA) iron ABC transporter, substrate-binding protein [Iron/managanease transport (CVF459)] [Escherichia coli O44:H18 042] | | Ferrous_iron_transport | Iron Uptake | | |
| VFG045727 | | | *csrA* | | (csrA) global regulator CsrA [Carbon storage regulator A (CVF362)] [Legionella longbeachae NSW150] | | Carbon_storage_regulator_A | Regulation | | |
| VFG018243 | | | *luxS* | | (luxS) S-ribosylhomocysteinase [Autoinducer-2 (CVF628)] [Vibrio parahaemolyticus RIMD 2210633] | | Autoinducer_2 | QuorumSensing | | |
| VFG049018 | | | *rcsB* | | (rcsB) transcriptional regulator RcsB [RcsAB (VF0571)] [Klebsiella pneumoniae subsp. pneumoniae NTUH-K2044] | | RcsAB | Regulation | | |
| VFG048971 | | | *wzc* | | (wzc) tyrosine autokinase [Capsule (CVF854)] [Klebsiella pneumoniae subsp. pneumoniae MGH 78578] | | Capsular_polysaccharide | Antiphagocytosis | | |
| VFG049083 | | | *rmlC* | | (rmlC) dTDP-4-dehydrorhamnose 3,5-epimerase [LPS rfb locus (CVF857)] [Klebsiella pneumoniae subsp. pneumoniae MGH 78578] | | Capsular_polysaccharide | Antiphagocytosis | | |
| VFG003114 | | | *psn/fyuA* | | (psn/fyuA) pesticin/yersiniabactin receptor protein [Yersiniabactin (CVF051)] [Yersinia pestis biovar Microtus str. 91001] | | Yersiniabactin | Iron Uptake | | |
| VFG012564 | | | *ybtE* | | (ybtE) yersiniabactin siderophore biosynthetic protein YbtE [Yersiniabactin siderophore (CVF458)] [Escherichia coli 536] | | Yersiniabactin | Iron Uptake | | |
| VFG034152 | | | *ybtT* | | (ybtT) putative thioesterase YbtT [Yersiniabactin siderophore (CVF458)] [Escherichia coli O17:K52:H18 str. UMN026] | | Yersiniabactin | Iron Uptake | | |
| VFG002743 | | | *ybtU* | | (ybtU) thiazolinyl-S-HMWP1 reductase [Yersiniabactin (CVF051)] [Yersinia pestis KIM 10] | | Yersiniabactin | Iron Uptake | | |
| VFG034118 | | | *irp1* | | (irp1) High-molecular-weight nonribosomal peptide/polyketide synthetase 1 [Yersiniabactin siderophore (CVF458)] [Escherichia coli O17:K52:H18 str. UMN026] | | Yersiniabactin | Iron Uptake | | |
| VFG034101 | | | *irp2* | | (irp2) High-molecular-weight nonribosomal peptide/polyketide synthetase 2 [Yersiniabactin siderophore (CVF458)] [Escherichia coli O17:K52:H18 str. UMN026] | | Yersiniabactin | Iron Uptake | | |
| VFG034084 | | | *ybtA* | | (ybtA) AraC family transcriptional regulator [Yersiniabactin siderophore (CVF458)] [Escherichia coli O17:K52:H18 str. UMN026] | | Yersiniabactin | Iron Uptake | | |
| VFG002881 | | | *ybtP* | | (ybtP) lipoprotein inner membrane ABC-transporter [Yersiniabactin (CVF051)] [Yersinia pestis Antiqua] | | Yersiniabactin | Iron Uptake | | |
| VFG034050 | | | *ybtQ* | | (ybtQ) yersiniabactin-iron ABC transporter permease and ATP-binding protein YbtQ [Yersiniabactin siderophore (CVF458)] [Escherichia coli O17:K52:H18 str. UMN026] | | Yersiniabactin | Iron Uptake | | |
| VFG002883 | | | *ybtX* | | (ybtX) putative signal transducer [Yersiniabactin (CVF051)] [Yersinia pestis Antiqua] | | Yersiniabactin | Iron Uptake | | |
| VFG012529 | | | *ybtS* | | (ybtS) putative salicylate synthetase [Yersiniabactin siderophore (CVF458)] [Escherichia coli UTI89] | | Yersiniabactin | Iron Uptake | | |
| VFG049005 | | | *rcsA* | | (rcsA) colanic acid capsular biosynthesis activation protein A [RcsAB (CVF856)] [Klebsiella pneumoniae KCTC 2242] | | RcsAB | Regulation | | |
| VFG043099 | | | *fliY* | | (fliY) cystine transporter subunit [peritrichous flagella (AI140)] [Escherichia coli O157:H7 str. EDL933] | | Flagella | Motility Invasion Adherence | | |
| VFG013466 | | | *kdsA* | | (kdsA) 2-dehydro-3-deoxyphosphooctonate aldolase [LOS (CVF494)] [Haemophilus influenzae 86-028NP] | | LPS | Immune Evasion | | |
| VFG013348 | | | *galU* | | (galU) carbon storage regulator [LOS (CVF494)] [Haemophilus influenzae PittEE] | | LOS_Endotoxin | Endotoxin | | |
| VFG004044 | | | *mgtC* | | (mgtC) conserved hyopthetical protein [Mg2+ transport (CVF005)] [Salmonella enterica subsp. enterica serovar Typhi str. CT18] | | Mg2+_transport | Magnesium Uptake | | |
| VFG044322 | | | *iroE* | | (iroE) siderophore esterase IroE [Sal (VF0563)] [Klebsiella pneumoniae subsp. pneumoniae NTUH-K2044] | | Salmochelin_synthesis_and_transport | Iron Uptake | | |
| VFG048784 | | | *sciN/tssJ* | | (sciN/tssJ) type VI secretion system lipoprotein TssJ [T6SS (VF0569)] [Klebsiella pneumoniae subsp. pneumoniae HS11286] | | T6SS_I | SecretionSystem | | |
| VFG048776 | | | *tssG* | | (tssG) type VI secretion system baseplate subunit TssG [T6SS (VF0569)] [Klebsiella pneumoniae subsp. pneumoniae HS11286] | | T6SS_I | SecretionSystem | | |
| VFG048767 | | | *tssF* | | (tssF) type VI secretion system baseplate subunit TssF [T6SS (VF0569)] [Klebsiella pneumoniae subsp. pneumoniae HS11286] | | T6SS_I | SecretionSystem | | |
| VFG048758 | | | *impA/tssA* | | (impA/tssA) type VI secretion system protein TssA [T6SS (VF0569)] [Klebsiella pneumoniae subsp. pneumoniae HS11286] | | T6SS_I | SecretionSystem | | |
| VFG048751 | | | *icmF/tssM* | | (icmF/tssM) type VI secretion protein TssM [T6SS (VF0569)] [Klebsiella pneumoniae subsp. pneumoniae HS11286] | | T6SS_I | SecretionSystem | | |
| VFG048736 | | | *tle1* | | (tle1) type VI secretion system effector [T6SS (VF0569)] [Klebsiella pneumoniae subsp. pneumoniae HS11286] | | T6SS_I | SecretionSystem | | |
| VFG048732 | | | *tli1* | | (tli1) type VI secretion system immunity protein [T6SS (VF0569)] [Klebsiella pneumoniae subsp. pneumoniae HS11286] | | T6SS_I | SecretionSystem | | |
| VFG048704 | | | *vgrG/tssI* | | (vgrG/tssI) type VI secretion system tip protein VgrG [T6SS (VF0569)] [Klebsiella pneumoniae subsp. pneumoniae HS11286] | | T6SS_I | SecretionSystem | | |
| VFG048693 | | | *clpV/tssH* | | (clpV/tssH) type VI secretion system ATPase TssH [T6SS (VF0569)] [Klebsiella pneumoniae subsp. pneumoniae HS11286] | | T6SS_I | SecretionSystem | | |
| VFG048683 | | | *hcp/tssD* | | (hcp/tssD) type VI secretion system protein, Hcp family [T6SS (VF0569)] [Klebsiella pneumoniae subsp. pneumoniae HS11286] | | T6SS_I | SecretionSystem | | |
| VFG048661 | | | *dotU/tssL* | | (dotU/tssL) type VI secretion system protein, DotU/TssL family [T6SS (VF0569)] [Klebsiella pneumoniae subsp. pneumoniae HS11286] | | T6SS_I | SecretionSystem | | |
| VFG048650 | | | *vasE/tssK* | | (vasE/tssK) type VI secretion system baseplate subunit TssK [T6SS (VF0569)] [Klebsiella pneumoniae subsp. pneumoniae HS11286] | | T6SS_I | SecretionSystem | | |
| VFG048639 | | | *vipB/tssC* | | (vipB/tssC) type VI secretion system contractile sheath large subunit VipB [T6SS (VF0569)] [Klebsiella pneumoniae subsp. pneumoniae HS11286] | | T6SS_I | SecretionSystem | | |
| VFG048628 | | | *vipA/tssB* | | (vipA/tssB) type VI secretion system contractile sheath small subunit VipA [T6SS (VF0569)] [Klebsiella pneumoniae subsp. pneumoniae HS11286] | | T6SS_I | SecretionSystem | | |
| VFG048546 | | | *iroN* | | (iroN) outer membrane receptor FepA [Salmochelin (CVF850)] [Klebsiella pneumoniae subsp. pneumoniae MGH 78578] | | Salmochelin_synthesis_and_transport | Iron Uptake | | |
| VFG018396 | | | *phoP* | | (phoP) hypothetical protein [PhoPQ (CVF010)] [Salmonella enterica subsp. enterica serovar Paratyphi B str. SPB7] | | PhoPQ | Regulation | | |
| VFG021077 | | | *phoQ* | | (phoQ) sensor protein PhoQ [PhoPQ (CVF010)] [Salmonella enterica subsp. enterica serovar Agona str. SL483] | | PhoPQ | Regulation | | |
| VFG048614 | | | *iutA* | | (iutA) ferric aerobactin receptor [Aerobactin (CVF852)] [Klebsiella pneumoniae 342] | | Aerobactin_transport | Iron Uptake | | |
| VFG001443 | | | *ompA* | | (ompA) outer membrane protein A [OmpA (VF0236)] [Escherichia coli O18:K1:H7 str. RS218] | | OmpA | Invasion | | |
| VFG038845 | | | *nueA* | | (nueA) 3-deoxy-D-manno-octulosonate cytidylyltransferase [Polar flagella (VF0473)] [Aeromonas hydrophila ML09-119] | | Polar_flagella | Adherence | | |
| VFG048409 | | | *entA* | | (entA) 2,3-dihydroxybenzoate-2,3-dehydrogenase [Ent (VF0562)] [Klebsiella pneumoniae subsp. pneumoniae NTUH-K2044] | | Enterobactin_synthesis | Iron Uptake | | |
| VFG048419 | | | *entB* | | (entB) 2,3-dihydro-2,3-dihydroxybenzoate synthetase, isochroismatase [Ent (VF0562)] [Klebsiella pneumoniae subsp. pneumoniae NTUH-K2044] | | Enterobactin_synthesis | Iron Uptake | | |
| VFG048427 | | | *entE* | | (entE) enterobactin synthase subunit E [Ent siderophore (CVF849)] [Klebsiella pneumoniae KCTC 2242] | | Acinetobactin | Iron Uptake | | |
| VFG048438 | | | *entC* | | (entC) isochorismate synthase [Ent siderophore (CVF849)] [Klebsiella pneumoniae subsp. pneumoniae MGH 78578] | | Enterobactin_synthesis | Iron Uptake | | |
| VFG048444 | | | *fepB* | | (fepB) iron-enterobactin transporter periplasmic binding protein [Ent siderophore (CVF849)] [Klebsiella pneumoniae 342] | | Enterobactin_transport | Iron Uptake | | |
| VFG048454 | | | *entS* | | (entS) enterobactin exporter EntS [Ent siderophore (CVF849)] [Klebsiella pneumoniae 342] | | Ent_siderophore | Iron Uptake | | |
| VFG048478 | | | *fepG* | | (fepG) iron-enterobactin transporter permease [Ent (VF0562)] [Klebsiella pneumoniae subsp. pneumoniae NTUH-K2044] | | Enterobactin_transport | Iron Uptake | | |
| VFG048488 | | | *fepC* | | (fepC) iron-enterobactin transporter ATP-binding protein [Ent (VF0562)] [Klebsiella pneumoniae subsp. pneumoniae NTUH-K2044] | | Enterobactin_transport | Iron Uptake | | |
| VFG048497 | | | *entF* | | (entF) enterobactin synthase subunit F [Ent siderophore (CVF849)] [Klebsiella pneumoniae subsp. pneumoniae MGH 78578] | | Enterobactin_synthesis | Iron Uptake | | |
| VFG048508 | | | *fes* | | (fes) enterobactin/ferric enterobactin esterase [Ent (VF0562)] [Klebsiella pneumoniae subsp. pneumoniae NTUH-K2044] | | Ent_siderophore | Iron Uptake | | |
| VFG048517 | | | *fepA* | | (fepA) outer membrane receptor FepA [Ent siderophore (CVF849)] [Klebsiella pneumoniae subsp. pneumoniae MGH 78578] | | Enterobactin_transport | Iron Uptake | | |
| VFG048528 | | | *entD* | | (entD) enterochelin synthetase component D [Ent (VF0562)] [Klebsiella pneumoniae subsp. pneumoniae NTUH-K2044] | | Brucebactin | IronUptake | | |
| VFG049133 | | | *acrA* | | (acrA) acriflavine resistance protein A [AcrAB (VF0568)] [Klebsiella pneumoniae subsp. pneumoniae NTUH-K2044] | | AcrAB | Efflux Pump | | |
| VFG034520 | | | *ecpE* | | (ecpE) chaperone [E. coli common pilus (ECP) (CVF625)] [Escherichia coli O26:H11 str. 11368] | | E._coli_common_pilus_ECP | Adherence | | |
| VFG018221 | | | *ecpD* | | (ecpD) Hypothetical protein yagW [E. coli common pilus (ECP) (CVF625)] [Escherichia coli CFT073] | | E._coli_common_pilus_ECP | Adherence | | |
| VFG034475 | | | *ecpC* | | (ecpC) hypothetical protein [E. coli common pilus (ECP) (CVF625)] [Escherichia coli E24377A] | | E._coli_common_pilus_ECP | Adherence | | |
| VFG034442 | | | *ecpB* | | (ecpB) hypothetical protein [E. coli common pilus (ECP) (CVF625)] [Escherichia coli O7:K1 str. IAI39] | | E._coli_common_pilus_ECP | Adherence | | |
| VFG034419 | | | *ecpA* | | (ecpA) fimbrillin MatB [E. coli common pilus (ECP) (CVF625)] [Escherichia coli E24377A] | | E._coli_common_pilus_ECP | Adherence | | |
| VFG034382 | | | *ecpR* | | (ecpR) transcriptional regulator [E. coli common pilus (ECP) (CVF625)] [Escherichia coli O104:H4 str. 2009EL-2050] | | E._coli_common_pilus_ECP | Adherence | | |
| VFG012874 | | | *ipaH* | | (ipaH) hypothetical protein [Mxi-Spa TTSS effectors controlled by MxiE (CVF465)] [Shigella flexneri 2a str. 2457T] | | Mxi_Spa_TTSS_effectors_controlled_by_MxiE | Secretion System | | |
| VFG013421 | | | *gmhA/lpcA* | | (gmhA/lpcA) phosphoheptose isomerase [LOS (CVF494)] [Haemophilus influenzae PittGG] | | LOS_Endotoxin | Endotoxin | | |
| VFG038287 | | | *exeG* | | (exeG) Pullulanase G protein [T2SS (CVF780)] [Aeromonas veronii B565] | | T2SS | Secretion System | | |
| VFG013414 | | | *lpxC* | | (lpxC) hypothetical protein [LOS (CVF494)] [Haemophilus influenzae PittEE] | | LPS | Immune Evasion | | |
| VFG034663 | | | *ibeB* | | (ibeB) copper/silver efflux system protein CusC [Invasion of brain endothelial cells (Ibes) (CVF429)] [Escherichia coli O103:H2 str. 12009] | | Invasion_of_brain_endothelial_cells_Ibes | Invasion | | |
| VFG013531 | | | *pgi* | | (pgi) glucose-6-phosphate isomerase [Exopolysaccharide (CVF495)] [Haemophilus influenzae 86-028NP] | | Exopolysaccharide | Immune Evasion | | |
| VFG013612 | | | *hemE* | | (hemE) uroporphyrinogen decarboxylase [Heme biosynthesis (CVF506)] [Haemophilus somnus 129PT] | | Heme_biosynthesis | Iron Uptake | | |
| VFG007659 | | | *rmlB* | | (rmlB) dTDP-glucose 4,6-dehydratase [Capsular polysaccharide (CVF282)] [Vibrio fischeri ES114] | | Capsular_polysaccharide | Antiphagocytosis | | |
| VFG007640 | | | *wbjD/wecB* | | (wbjD/wecB) UDP-N-acetylglucosamine 2-epimerase [Capsular polysaccharide (CVF282)] [Vibrio vulnificus YJ016] | | Capsular_polysaccharide | Antiphagocytosis | | |
| VFG048399 | | | *mrkA* | | (mrkA) hypothetical protein [Type 3 fimbriae (CVF848)] [Klebsiella pneumoniae subsp. rhinoscleromatis SB3432] | | Type_3_fimbriae | Adherence | | |
| VFG043626 | | | *mrkB* | | (mrkB) fimbrial chaperone protein mrkB precursor [Type 3 fimbriae (VF0567)] [Klebsiella pneumoniae subsp. pneumoniae NTUH-K2044] | | Type_3_fimbriae | Adherence | | |
| VFG043625 | | | *mrkC* | | (mrkC) fimbrial biogenesis outer membrane usher protein mrkC precursor [Type 3 fimbriae (VF0567)] [Klebsiella pneumoniae subsp. pneumoniae NTUH-K2044] | | Type_3_fimbriae | Adherence | | |
| VFG043624 | | | *mrkD* | | (mrkD) fimbrial adhesin protein precursor MrkD [Type 3 fimbriae (VF0567)] [Klebsiella pneumoniae subsp. pneumoniae NTUH-K2044] | | Type_3_fimbriae | Adherence | | |
| VFG043623 | | | *mrkF* | | (mrkF) type 3 fimbrial minor pilin subunit MrkF [Type 3 fimbriae (VF0567)] [Klebsiella pneumoniae subsp. pneumoniae NTUH-K2044] | | Type_3_fimbriae | Adherence | | |
| VFG048348 | | | *mrkJ* | | (mrkJ) phosphodiesterase [Type 3 fimbriae (VF0567)] [Klebsiella pneumoniae subsp. pneumoniae NTUH-K2044] | | Type_3_fimbriae | Adherence | | |
| VFG048338 | | | *mrkI* | | (mrkI) LuxR family regulatory protein [Type 3 fimbriae (VF0567)] [Klebsiella pneumoniae subsp. pneumoniae NTUH-K2044] | | Type_3_fimbriae | Adherence | | |
| VFG048327 | | | *mrkH* | | (mrkH) transcriptional activator [Type 3 fimbriae (VF0567)] [Klebsiella pneumoniae subsp. pneumoniae NTUH-K2044] | | Type_3_fimbriae | Adherence | | |
| VFG048247 | | | *fimA* | | (fimA) type 1 major fimbrial subunit precursor [Type I fimbriae (VF0566)] [Klebsiella pneumoniae subsp. pneumoniae NTUH-K2044] | | Type_3_fimbriae | Adherence | | |
| VFG048229 | | | *fimB* | | (fimB) tyrosine recombinase [Type I fimbriae (VF0566)] [Klebsiella pneumoniae subsp. pneumoniae NTUH-K2044] | | Type_3_fimbriae | Adherence | | |
| VFG048236 | | | *fimE* | | (fimE) tyrosine recombinase [Type I fimbriae (CVF847)] [Klebsiella pneumoniae subsp. pneumoniae MGH 78578] | | Type_I_fimbriae | Adherence | | |
| VFG048600 | | | *iucA* | | (iucA) aerobactin Synthetase IucA [Aerobactin (VF0565)] [Klebsiella pneumoniae subsp. pneumoniae NTUH-K2044] | | Aerobactin_synthesis | Iron Uptake | | |
| VFG048603 | | | *iucB* | | (iucB) N-acetyltransferase IucB [Aerobactin (VF0565)] [Klebsiella pneumoniae subsp. pneumoniae NTUH-K2044] | | Aerobactin_synthesis | Iron Uptake | | |
| VFG048606 | | | *iucC* | | (iucC) aerobactin siderophore biosynthesis protein IucC [Aerobactin (VF0565)] [Klebsiella pneumoniae subsp. pneumoniae NTUH-K2044] | | Aerobactin_synthesis | Iron Uptake | | |
| VFG048609 | | | *iucD* | | (iucD) lysine 6-monooxygenase IucD [Aerobactin (VF0565)] [Klebsiella pneumoniae subsp. pneumoniae NTUH-K2044] | | Aerobactin_synthesis | Iron Uptake | | |
| VFG033934 | | | *iroB* | | (iroB) IroB, putative glucosyltransferase [Salmochelin siderophore (CVF456)] [Escherichia coli O45:K1:H7 str. S88] | | Salmochelin_synthesis_and_transport | Iron Uptake | | |
| VFG012512 | | | *iroC* | | (iroC) IroC [Salmochelin (IA014)] [Escherichia coli APEC O1] | | Salmochelin_synthesis_and_transport | Iron Uptake | | |
| VFG012505 | | | *iroD* | | (iroD) esterase [Salmochelin (IA013)] [Escherichia coli CFT073] | | Salmochelin_synthesis_and_transport | Iron Uptake | | |
| VFG012590 | | | *sitD* | | (sitD) SitD [Iron/managanease transport (CVF459)] [Escherichia coli APEC O1] | | Ferrous_iron_transport | Iron Uptake | | |
| VFG049095 | | | *wzm* | | (wzm) lipopolysaccharide O-antigen ABC transport system transmembrane component [LPS (VF0561)] [Klebsiella pneumoniae subsp. pneumoniae NTUH-K2044] | | Capsule_I | Antiphagocytosis | | |
| VFG049084 | | | *wzt* | | (wzt) lipopolysaccharide O-antigen ABC transport system ATP-binding component [LPS (VF0561)] [Klebsiella pneumoniae subsp. pneumoniae NTUH-K2044] | | LPS | Immune Evasion | | |
| VFG000331 | | | *rfaE* | | (rfaE) ADP-heptose synthase [LOS (VF0044)] [Haemophilus influenzae Rd KW20] | | LOS_Endotoxin | Endotoxin | | |
