# Supplementary material for: Phenotypic and genomic characterization of ST11-K1 CR-hvKP with highly homologous blaKPC-2-bearing plasmids in China
Source: mSystems. 2024 Nov 18;9(12):e01101-24. doi: 10.1128/msystems.01101-24 (PMC11651102; doi:10.1128/msystems.01101-24)
Supplement: Table S4 — SNP loci detected in 402 genomes using the MUMmer alignment software. [file msystems.01101-24-s0005.docx]

**Table S4** All the SNP loci were detected in 402 genomes using the MUMmer alignment software

| Sample_name | Start_syn | Stop_syn | Start_nonsyn | Stop_nonsyn | Premature_stop | Synonymous | Nonsynonymous | Total_CDS_SNP | Intergenic | Total SNP | Coverage |
| --- | --- | --- | --- | --- | --- | --- | --- | --- | --- | --- | --- |
| SAMD00060934 | 1 | 9 | 9 | 7 | 6 | 10,899 | 2,249 | 13,172 | 1,706 | 14,878 | 82.30% |
| SAMD00129511 | 0 | 11 | 5 | 7 | 14 | 9,950 | 2,125 | 12,105 | 1,650 | 13,755 | 83.40% |
| SAMD00129512 | 0 | 11 | 5 | 7 | 14 | 9,950 | 2,125 | 12,105 | 1,650 | 13,755 | 83.17% |
| SAMD00129519 | 0 | 11 | 5 | 8 | 11 | 9,954 | 2,128 | 12,109 | 1,646 | 13,755 | 84.04% |
| SAMD00129523 | 0 | 9 | 7 | 8 | 5 | 10,359 | 2,173 | 12,556 | 1,620 | 14,176 | 82.44% |
| SAMD00129527 | 1 | 8 | 6 | 8 | 9 | 10,382 | 2,161 | 12,570 | 1,621 | 14,191 | 82.19% |
| SAMD00129533 | 0 | 10 | 5 | 8 | 8 | 10,445 | 2,165 | 12,637 | 1,665 | 14,302 | 82.78% |
| SAMD00129540 | 1 | 9 | 10 | 7 | 4 | 10,896 | 2,241 | 13,160 | 1,708 | 14,868 | 81.64% |
| SAMD00129546 | 1 | 9 | 10 | 7 | 5 | 10,906 | 2,239 | 13,169 | 1,703 | 14,872 | 82.14% |
| SAMD00129551 | 1 | 9 | 10 | 7 | 4 | 10,908 | 2,247 | 13,178 | 1,712 | 14,890 | 83.45% |
| SAMD00129566 | 1 | 9 | 9 | 7 | 7 | 10,911 | 2,264 | 13,200 | 1,705 | 14,905 | 83.32% |
| SAMD00129569 | 1 | 8 | 6 | 8 | 11 | 10,388 | 2,179 | 12,596 | 1,622 | 14,218 | 81.91% |
| SAMD00129572 | 1 | 10 | 4 | 8 | 4 | 10,380 | 2,158 | 12,555 | 1,754 | 14,309 | 82.89% |
| SAMD00129597 | 0 | 7 | 8 | 7 | 10 | 10,695 | 2,233 | 12,954 | 1,796 | 14,750 | 80.39% |
| SAMD00129599 | 1 | 9 | 9 | 7 | 8 | 10,911 | 2,260 | 13,197 | 1,704 | 14,901 | 83.25% |
| SAMD00129600 | 0 | 10 | 5 | 8 | 10 | 10,441 | 2,166 | 12,636 | 1,666 | 14,302 | 82.10% |
| SAMD00129608 | 1 | 8 | 6 | 8 | 11 | 10,387 | 2,175 | 12,591 | 1,621 | 14,212 | 80.82% |
| SAMD00129614 | 0 | 7 | 8 | 7 | 10 | 10,682 | 2,235 | 12,943 | 1,793 | 14,736 | 81.02% |
| SAMD00129619 | 0 | 10 | 5 | 8 | 8 | 10,438 | 2,168 | 12,633 | 1,663 | 14,296 | 82.38% |
| SAMD00129628 | 0 | 8 | 7 | 6 | 11 | 10,880 | 2,246 | 13,152 | 1,777 | 14,929 | 81.59% |
| SAMD00129631 | 0 | 12 | 5 | 7 | 8 | 10,101 | 2,100 | 12,227 | 1,669 | 13,896 | 83.56% |
| SAMD00129634 | 0 | 11 | 5 | 7 | 11 | 9,953 | 2,127 | 12,107 | 1,644 | 13,751 | 85.16% |
| SAMD00129636 | 0 | 12 | 5 | 7 | 8 | 10,105 | 2,109 | 12,240 | 1,666 | 13,906 | 83.55% |
| SAMD00129641 | 1 | 9 | 8 | 7 | 5 | 10,901 | 2,243 | 13,166 | 1,703 | 14,869 | 81.76% |
| SAMD00129647 | 0 | 10 | 5 | 8 | 10 | 10,436 | 2,165 | 12,630 | 1,667 | 14,297 | 83.88% |
| SAMD00212274 | 1 | 9 | 9 | 7 | 5 | 10,908 | 2,252 | 13,183 | 1,705 | 14,888 | 83.66% |
| SAMEA2272247 | 0 | 7 | 8 | 10 | 8 | 10,828 | 2,255 | 13,108 | 1,744 | 14,852 | 82.64% |
| SAMEA6099342 | 0 | 10 | 5 | 8 | 8 | 10,442 | 2,168 | 12,637 | 1,666 | 14,303 | 81.84% |
| SAMEA6099358 | 1 | 9 | 9 | 7 | 7 | 10,922 | 2,274 | 13,221 | 1,711 | 14,932 | 83.01% |
| SAMEA6099410 | 0 | 0 | 0 | 0 | 0 | 3 | 11 | 14 | 3 | 17 | 94.23% |
| SAMEA6099420 | 0 | 0 | 0 | 0 | 0 | 3 | 11 | 14 | 3 | 17 | 94.16% |
| SAMEA6099423 | 0 | 0 | 0 | 0 | 0 | 3 | 11 | 14 | 3 | 17 | 94.00% |
| SAMEA6099424 | 0 | 0 | 0 | 0 | 1 | 3 | 11 | 15 | 3 | 18 | 94.08% |
| SAMEA6099425 | 0 | 0 | 0 | 0 | 0 | 3 | 11 | 14 | 3 | 17 | 94.13% |
| SAMEA6099433 | 0 | 0 | 0 | 0 | 0 | 3 | 12 | 15 | 3 | 18 | 94.20% |
| SAMEA6099452 | 0 | 0 | 0 | 0 | 0 | 3 | 12 | 15 | 5 | 20 | 93.93% |
| SAMEA8552927 | 0 | 11 | 8 | 8 | 10 | 10,699 | 2,200 | 12,930 | 1,666 | 14,596 | 82.50% |
| SAMEA8602828 | 0 | 8 | 7 | 6 | 10 | 10,884 | 2,247 | 13,156 | 1,779 | 14,935 | 83.55% |
| SAMN02471933 | 0 | 10 | 5 | 8 | 10 | 10,445 | 2,189 | 12,663 | 1,670 | 14,333 | 81.11% |
| SAMN02581256 | 1 | 10 | 7 | 9 | 8 | 10,722 | 2,265 | 13,017 | 1,719 | 14,736 | 81.00% |
| SAMN02581345 | 0 | 5 | 6 | 10 | 6 | 10,373 | 2,209 | 12,602 | 1,761 | 14,363 | 83.92% |
| SAMN02581350 | 0 | 5 | 6 | 10 | 6 | 10,373 | 2,209 | 12,602 | 1,761 | 14,363 | 83.82% |
| SAMN02603582 | 0 | 7 | 8 | 10 | 12 | 10,848 | 2,281 | 13,158 | 1,752 | 14,910 | 82.43% |
| SAMN02869886 | 0 | 7 | 8 | 7 | 10 | 10,692 | 2,243 | 12,961 | 1,798 | 14,759 | 79.36% |
| SAMN02869887 | 0 | 7 | 8 | 7 | 10 | 10,701 | 2,254 | 12,981 | 1,801 | 14,782 | 78.96% |
| SAMN04377968 | 1 | 9 | 9 | 7 | 6 | 10,908 | 2,253 | 13,185 | 1,703 | 14,888 | 83.85% |
| SAMN04377969 | 1 | 8 | 4 | 13 | 10 | 10,685 | 2,335 | 13,044 | 1,702 | 14,746 | 81.47% |
| SAMN05231874 | 1 | 9 | 9 | 7 | 6 | 10,910 | 2,246 | 13,180 | 1,702 | 14,882 | 83.70% |
| SAMN05250855 | 1 | 9 | 8 | 7 | 10 | 10,911 | 2,251 | 13,189 | 1,712 | 14,901 | 81.67% |
| SAMN05412805 | 2 | 8 | 7 | 7 | 7 | 8,068 | 1,742 | 9,838 | 1,372 | 11,210 | 84.54% |
| SAMN05413133 | 1 | 9 | 9 | 7 | 5 | 10,909 | 2,263 | 13,195 | 1,708 | 14,903 | 83.26% |
| SAMN05425526 | 1 | 9 | 8 | 7 | 5 | 10,896 | 2,251 | 13,169 | 1,704 | 14,873 | 84.07% |
| SAMN05928590 | 1 | 7 | 6 | 8 | 8 | 10,110 | 2,082 | 12,215 | 1,540 | 13,755 | 82.85% |
| SAMN05928591 | 1 | 9 | 9 | 7 | 6 | 10,909 | 2,247 | 13,180 | 1,700 | 14,880 | 83.46% |
| SAMN05945851 | 1 | 9 | 9 | 8 | 7 | 10,908 | 2,254 | 13,188 | 1,708 | 14,896 | 81.40% |
| SAMN05945909 | 1 | 9 | 8 | 7 | 7 | 10,916 | 2,260 | 13,200 | 1,702 | 14,902 | 79.34% |
| SAMN05945968 | 0 | 10 | 4 | 8 | 5 | 10,388 | 2,161 | 12,566 | 1,761 | 14,327 | 79.75% |
| SAMN06112188 | 1 | 9 | 9 | 7 | 4 | 10,899 | 2,242 | 13,163 | 1,705 | 14,868 | 84.17% |
| SAMN06562536 | 1 | 9 | 9 | 7 | 6 | 10,905 | 2,245 | 13,174 | 1,705 | 14,879 | 82.00% |
| SAMN06671956 | 0 | 11 | 5 | 8 | 12 | 9,949 | 2,120 | 12,098 | 1,650 | 13,748 | 86.95% |
| SAMN08915824 | 1 | 9 | 9 | 7 | 7 | 10,910 | 2,255 | 13,190 | 1,701 | 14,891 | 86.35% |
| SAMN08932551 | 0 | 7 | 8 | 7 | 10 | 10,682 | 2,231 | 12,939 | 1,794 | 14,733 | 82.16% |
| SAMN09499691 | 0 | 9 | 7 | 8 | 5 | 10,359 | 2,180 | 12,563 | 1,618 | 14,181 | 81.82% |
| SAMN09499694 | 0 | 9 | 7 | 8 | 5 | 10,362 | 2,176 | 12,562 | 1,618 | 14,180 | 82.13% |
| SAMN09499696 | 0 | 10 | 5 | 9 | 8 | 10,446 | 2,183 | 12,657 | 1,671 | 14,328 | 82.51% |
| SAMN09736899 | 2 | 9 | 9 | 8 | 8 | 10,905 | 2,259 | 13,191 | 1,709 | 14,900 | 83.41% |
| SAMN09737030 | 0 | 12 | 5 | 7 | 9 | 10,113 | 2,116 | 12,256 | 1,680 | 13,936 | 83.48% |
| SAMN10086661 | 0 | 7 | 8 | 7 | 10 | 10,682 | 2,231 | 12,939 | 1,779 | 14,718 | 80.59% |
| SAMN10086829 | 0 | 10 | 4 | 8 | 3 | 10,373 | 2,164 | 12,552 | 1,753 | 14,305 | 82.03% |
| SAMN10371631 | 1 | 8 | 6 | 8 | 10 | 10,385 | 2,176 | 12,589 | 1,625 | 14,214 | 83.37% |
| SAMN10433884 | 0 | 0 | 0 | 0 | 0 | 6 | 11 | 17 | 4 | 21 | 95.92% |
| SAMN10477349 | 0 | 10 | 5 | 8 | 10 | 10,445 | 2,180 | 12,654 | 1,671 | 14,325 | 80.68% |
| SAMN10600509 | 0 | 12 | 5 | 7 | 11 | 10,107 | 2,109 | 12,245 | 1,674 | 13,919 | 84.83% |
| SAMN10600511 | 0 | 10 | 5 | 8 | 10 | 10,445 | 2,159 | 12,633 | 1,669 | 14,302 | 84.33% |
| SAMN10600535 | 0 | 12 | 5 | 7 | 11 | 10,108 | 2,107 | 12,244 | 1,673 | 13,917 | 83.69% |
| SAMN10600584 | 0 | 7 | 10 | 7 | 10 | 10,688 | 2,242 | 12,958 | 1,799 | 14,757 | 81.73% |
| SAMN10678732 | 0 | 10 | 5 | 9 | 9 | 10,441 | 2,176 | 12,646 | 1,672 | 14,318 | 84.12% |
| SAMN10908921 | 1 | 9 | 8 | 7 | 8 | 10,919 | 2,326 | 13,270 | 1,714 | 14,984 | 84.76% |
| SAMN10956502 | 1 | 9 | 8 | 7 | 8 | 10,922 | 2,311 | 13,258 | 1,717 | 14,975 | 84.86% |
| SAMN11039667 | 0 | 8 | 8 | 10 | 8 | 10,822 | 2,257 | 13,104 | 1,746 | 14,850 | 81.05% |
| SAMN11054834 | 1 | 9 | 9 | 7 | 4 | 10,901 | 2,255 | 13,178 | 1,704 | 14,882 | 84.67% |
| SAMN11125719 | 1 | 9 | 9 | 7 | 4 | 10,905 | 2,249 | 13,176 | 1,701 | 14,877 | 82.36% |
| SAMN11264881 | 0 | 10 | 4 | 8 | 3 | 10,379 | 2,165 | 12,559 | 1,753 | 14,312 | 82.06% |
| SAMN11533775 | 0 | 0 | 0 | 0 | 0 | 7 | 23 | 30 | 4 | 34 | 92.35% |
| SAMN11533776 | 0 | 0 | 0 | 0 | 0 | 7 | 23 | 30 | 4 | 34 | 92.26% |
| SAMN11533777 | 0 | 0 | 0 | 0 | 0 | 7 | 23 | 30 | 4 | 34 | 92.32% |
| SAMN11533778 | 0 | 0 | 0 | 0 | 0 | 7 | 23 | 30 | 4 | 34 | 91.99% |
| SAMN11533782 | 0 | 0 | 0 | 0 | 0 | 7 | 23 | 30 | 4 | 34 | 92.28% |
| SAMN11533784 | 0 | 0 | 0 | 0 | 0 | 7 | 23 | 30 | 5 | 35 | 92.47% |
| SAMN11533811 | 0 | 0 | 0 | 0 | 0 | 7 | 23 | 30 | 4 | 34 | 92.32% |
| SAMN11533812 | 0 | 0 | 0 | 0 | 0 | 7 | 25 | 32 | 4 | 36 | 92.21% |
| SAMN11579556 | 0 | 8 | 7 | 6 | 9 | 10,886 | 2,247 | 13,157 | 1,784 | 14,941 | 83.20% |
| SAMN11878529 | 1 | 9 | 9 | 7 | 7 | 10,909 | 2,269 | 13,203 | 1,706 | 14,909 | 83.01% |
| SAMN11878530 | 1 | 9 | 9 | 7 | 5 | 10,900 | 2,250 | 13,173 | 1,702 | 14,875 | 82.93% |
| SAMN11878531 | 1 | 9 | 9 | 7 | 5 | 10,899 | 2,257 | 13,179 | 1,709 | 14,888 | 82.74% |
| SAMN11878532 | 1 | 9 | 9 | 7 | 7 | 10,909 | 2,265 | 13,199 | 1,700 | 14,899 | 81.88% |
| SAMN11878533 | 1 | 9 | 9 | 7 | 7 | 10,909 | 2,268 | 13,202 | 1,700 | 14,902 | 82.07% |
| SAMN11878534 | 1 | 9 | 9 | 7 | 6 | 10,902 | 2,259 | 13,185 | 1,708 | 14,893 | 81.49% |
| SAMN11878535 | 1 | 9 | 9 | 8 | 7 | 10,912 | 2,267 | 13,205 | 1,708 | 14,913 | 81.18% |
| SAMN11878537 | 1 | 9 | 9 | 7 | 11 | 10,913 | 2,291 | 13,233 | 1,702 | 14,935 | 80.23% |
| SAMN11878538 | 1 | 9 | 10 | 7 | 4 | 10,906 | 2,251 | 13,180 | 1,702 | 14,882 | 82.50% |
| SAMN11998171 | 0 | 12 | 5 | 7 | 11 | 10,104 | 2,111 | 12,244 | 1,677 | 13,921 | 83.87% |
| SAMN12003074 | 1 | 9 | 8 | 7 | 8 | 10,908 | 2,269 | 13,202 | 1,709 | 14,911 | 83.66% |
| SAMN12101397 | 0 | 7 | 8 | 7 | 11 | 10,682 | 2,242 | 12,951 | 1,797 | 14,748 | 81.24% |
| SAMN12147558 | 1 | 12 | 5 | 7 | 11 | 11,097 | 2,387 | 13,513 | 1,662 | 15,175 | 81.97% |
| SAMN12250703 | 0 | 10 | 5 | 8 | 9 | 10,439 | 2,185 | 12,652 | 1,668 | 14,320 | 80.66% |
| SAMN12250733 | 1 | 9 | 9 | 7 | 4 | 10,906 | 2,256 | 13,184 | 1,707 | 14,891 | 82.67% |
| SAMN12250761 | 0 | 8 | 4 | 9 | 12 | 10,948 | 2,389 | 13,365 | 1,712 | 15,077 | 81.07% |
| SAMN12330902 | 0 | 9 | 7 | 8 | 5 | 10,363 | 2,178 | 12,565 | 1,620 | 14,185 | 81.49% |
| SAMN12330903 | 1 | 9 | 9 | 8 | 7 | 10,905 | 2,254 | 13,185 | 1,697 | 14,882 | 83.31% |
| SAMN12330904 | 0 | 10 | 5 | 8 | 10 | 10,445 | 2,183 | 12,657 | 1,671 | 14,328 | 81.70% |
| SAMN12330906 | 1 | 9 | 9 | 7 | 9 | 10,906 | 2,260 | 13,193 | 1,703 | 14,896 | 83.19% |
| SAMN12330907 | 0 | 7 | 5 | 8 | 13 | 10,979 | 2,323 | 13,323 | 1,762 | 15,085 | 80.32% |
| SAMN12330908 | 1 | 9 | 10 | 7 | 6 | 10,901 | 2,248 | 13,174 | 1,703 | 14,877 | 83.11% |
| SAMN12349649 | 1 | 9 | 9 | 7 | 7 | 10,910 | 2,272 | 13,207 | 1,707 | 14,914 | 84.74% |
| SAMN12349650 | 1 | 9 | 9 | 7 | 7 | 10,910 | 2,272 | 13,207 | 1,707 | 14,914 | 84.78% |
| SAMN12349652 | 1 | 9 | 9 | 7 | 5 | 10,903 | 2,256 | 13,182 | 1,704 | 14,886 | 83.37% |
| SAMN12349653 | 1 | 9 | 9 | 7 | 7 | 10,910 | 2,271 | 13,206 | 1,707 | 14,913 | 84.82% |
| SAMN12349654 | 1 | 9 | 9 | 7 | 7 | 10,910 | 2,271 | 13,206 | 1,707 | 14,913 | 84.77% |
| SAMN12349655 | 0 | 12 | 5 | 7 | 8 | 10,107 | 2,113 | 12,246 | 1,671 | 13,917 | 83.66% |
| SAMN12349710 | 1 | 9 | 10 | 7 | 6 | 10,907 | 2,246 | 13,178 | 1,712 | 14,890 | 82.98% |
| SAMN12419066 | 1 | 9 | 10 | 7 | 6 | 10,906 | 2,249 | 13,180 | 1,703 | 14,883 | 83.78% |
| SAMN12419067 | 1 | 9 | 10 | 7 | 6 | 10,906 | 2,249 | 13,180 | 1,703 | 14,883 | 84.25% |
| SAMN12419068 | 1 | 9 | 10 | 7 | 6 | 10,907 | 2,250 | 13,182 | 1,704 | 14,886 | 84.06% |
| SAMN12419069 | 1 | 9 | 10 | 7 | 4 | 10,904 | 2,258 | 13,185 | 1,701 | 14,886 | 83.81% |
| SAMN12419070 | 1 | 9 | 9 | 7 | 4 | 10,905 | 2,252 | 13,179 | 1,702 | 14,881 | 83.77% |
| SAMN12419071 | 0 | 12 | 8 | 6 | 6 | 10,141 | 2,093 | 12,258 | 1,706 | 13,964 | 86.11% |
| SAMN12419073 | 0 | 10 | 5 | 8 | 9 | 10,444 | 2,172 | 12,644 | 1,664 | 14,308 | 84.84% |
| SAMN12419075 | 0 | 7 | 8 | 7 | 10 | 10,680 | 2,232 | 12,938 | 1,797 | 14,735 | 81.37% |
| SAMN12419076 | 0 | 7 | 8 | 7 | 10 | 10,680 | 2,232 | 12,938 | 1,797 | 14,735 | 83.05% |
| SAMN12419077 | 0 | 7 | 8 | 7 | 10 | 10,681 | 2,232 | 12,939 | 1,797 | 14,736 | 81.43% |
| SAMN12419078 | 0 | 7 | 8 | 7 | 10 | 10,680 | 2,232 | 12,938 | 1,797 | 14,735 | 81.43% |
| SAMN12419079 | 0 | 7 | 8 | 7 | 10 | 10,680 | 2,233 | 12,939 | 1,797 | 14,736 | 81.39% |
| SAMN12419080 | 0 | 7 | 8 | 7 | 10 | 10,680 | 2,233 | 12,939 | 1,797 | 14,736 | 81.39% |
| SAMN12419081 | 0 | 7 | 8 | 7 | 10 | 10,680 | 2,232 | 12,938 | 1,797 | 14,735 | 81.43% |
| SAMN12419082 | 0 | 7 | 8 | 7 | 10 | 10,680 | 2,233 | 12,939 | 1,799 | 14,738 | 81.54% |
| SAMN12419083 | 0 | 7 | 8 | 7 | 10 | 10,680 | 2,232 | 12,938 | 1,798 | 14,736 | 82.36% |
| SAMN12868593 | 1 | 9 | 10 | 7 | 6 | 10,919 | 2,269 | 13,213 | 1,704 | 14,917 | 83.81% |
| SAMN12868596 | 0 | 12 | 5 | 7 | 11 | 10,104 | 2,103 | 12,236 | 1,669 | 13,905 | 84.58% |
| SAMN12868598 | 1 | 9 | 9 | 7 | 7 | 10,906 | 2,262 | 13,193 | 1,706 | 14,899 | 83.30% |
| SAMN12868599 | 0 | 10 | 5 | 8 | 10 | 10,448 | 2,163 | 12,640 | 1,667 | 14,307 | 82.94% |
| SAMN12868600 | 1 | 9 | 10 | 7 | 6 | 10,905 | 2,261 | 13,191 | 1,702 | 14,893 | 81.84% |
| SAMN12868601 | 0 | 12 | 5 | 7 | 10 | 10,113 | 2,119 | 12,260 | 1,671 | 13,931 | 82.38% |
| SAMN12868603 | 0 | 7 | 8 | 7 | 9 | 10,686 | 2,239 | 12,950 | 1,798 | 14,748 | 81.11% |
| SAMN12877716 | 0 | 6 | 5 | 9 | 6 | 10,310 | 2,158 | 12,487 | 1,615 | 14,102 | 83.54% |
| SAMN12877721 | 0 | 6 | 5 | 9 | 6 | 10,310 | 2,158 | 12,487 | 1,615 | 14,102 | 81.97% |
| SAMN13028530 | 0 | 10 | 5 | 9 | 4 | 10,381 | 2,165 | 12,564 | 1,761 | 14,325 | 82.02% |
| SAMN13301619 | 0 | 10 | 5 | 8 | 8 | 10,439 | 2,166 | 12,632 | 1,668 | 14,300 | 83.11% |
| SAMN13301620 | 0 | 7 | 8 | 7 | 11 | 10,685 | 2,230 | 12,942 | 1,794 | 14,736 | 82.24% |
| SAMN13301631 | 0 | 7 | 6 | 9 | 8 | 10,766 | 2,250 | 13,036 | 1,785 | 14,821 | 82.46% |
| SAMN13301637 | 0 | 7 | 6 | 9 | 8 | 10,766 | 2,251 | 13,037 | 1,785 | 14,822 | 82.42% |
| SAMN13301645 | 0 | 8 | 8 | 11 | 7 | 10,824 | 2,256 | 13,106 | 1,750 | 14,856 | 81.91% |
| SAMN13301649 | 0 | 7 | 8 | 7 | 9 | 10,686 | 2,226 | 12,937 | 1,793 | 14,730 | 80.89% |
| SAMN13301651 | 0 | 10 | 5 | 8 | 9 | 10,446 | 2,169 | 12,643 | 1,664 | 14,307 | 82.81% |
| SAMN13301654 | 1 | 9 | 9 | 7 | 9 | 10,911 | 2,257 | 13,195 | 1,709 | 14,904 | 83.21% |
| SAMN13301659 | 0 | 12 | 4 | 9 | 6 | 11,457 | 2,454 | 13,933 | 1,871 | 15,804 | 81.82% |
| SAMN13301662 | 1 | 9 | 10 | 8 | 6 | 10,897 | 2,247 | 13,169 | 1,703 | 14,872 | 83.32% |
| SAMN13301666 | 1 | 9 | 9 | 7 | 6 | 10,907 | 2,253 | 13,184 | 1,703 | 14,887 | 83.20% |
| SAMN13301674 | 0 | 10 | 6 | 7 | 7 | 10,286 | 2,178 | 12,487 | 1,680 | 14,167 | 83.39% |
| SAMN13301676 | 0 | 8 | 8 | 7 | 10 | 10,684 | 2,232 | 12,943 | 1,794 | 14,737 | 81.00% |
| SAMN13301681 | 0 | 10 | 6 | 7 | 7 | 10,286 | 2,174 | 12,483 | 1,680 | 14,163 | 83.55% |
| SAMN13301697 | 0 | 10 | 7 | 10 | 11 | 10,730 | 2,282 | 13,045 | 1,721 | 14,766 | 81.07% |
| SAMN13301699 | 1 | 9 | 9 | 7 | 8 | 10,909 | 2,257 | 13,192 | 1,713 | 14,905 | 82.77% |
| SAMN13441000 | 0 | 8 | 4 | 8 | 16 | 10,967 | 2,404 | 13,402 | 1,704 | 15,106 | 81.17% |
| SAMN13503954 | 1 | 9 | 9 | 8 | 5 | 10,909 | 2,254 | 13,187 | 1,698 | 14,885 | 83.75% |
| SAMN13503956 | 1 | 9 | 9 | 7 | 5 | 10,910 | 2,252 | 13,185 | 1,700 | 14,885 | 83.82% |
| SAMN13530329 | 0 | 7 | 8 | 7 | 11 | 10,687 | 2,228 | 12,942 | 1,795 | 14,737 | 80.65% |
| SAMN13563885 | 0 | 9 | 6 | 7 | 10 | 10,439 | 2,152 | 12,618 | 1,618 | 14,236 | 82.12% |
| SAMN13563886 | 0 | 8 | 8 | 10 | 9 | 10,832 | 2,268 | 13,127 | 1,743 | 14,870 | 82.00% |
| SAMN13563887 | 0 | 7 | 8 | 7 | 12 | 10,686 | 2,234 | 12,948 | 1,782 | 14,730 | 80.21% |
| SAMN13563888 | 1 | 9 | 9 | 7 | 6 | 10,972 | 2,291 | 13,287 | 1,723 | 15,010 | 83.27% |
| SAMN13563890 | 1 | 9 | 9 | 7 | 6 | 10,907 | 2,258 | 13,189 | 1,703 | 14,892 | 83.32% |
| SAMN13563892 | 1 | 9 | 9 | 7 | 6 | 10,907 | 2,263 | 13,194 | 1,698 | 14,892 | 83.22% |
| SAMN13563894 | 0 | 7 | 8 | 7 | 9 | 10,688 | 2,233 | 12,946 | 1,781 | 14,727 | 80.47% |
| SAMN13563896 | 1 | 9 | 10 | 7 | 5 | 10,896 | 2,239 | 13,159 | 1,702 | 14,861 | 84.18% |
| SAMN13563897 | 1 | 9 | 9 | 7 | 6 | 10,903 | 2,265 | 13,192 | 1,698 | 14,890 | 83.38% |
| SAMN13563902 | 1 | 9 | 9 | 7 | 6 | 10,913 | 2,259 | 13,195 | 1,705 | 14,900 | 83.25% |
| SAMN13563904 | 1 | 9 | 9 | 7 | 6 | 10,911 | 2,261 | 13,196 | 1,705 | 14,901 | 83.31% |
| SAMN13563905 | 1 | 9 | 10 | 7 | 7 | 10,910 | 2,262 | 13,198 | 1,708 | 14,906 | 83.21% |
| SAMN13563906 | 0 | 7 | 8 | 7 | 9 | 10,681 | 2,228 | 12,934 | 1,790 | 14,724 | 80.45% |
| SAMN13563909 | 1 | 9 | 10 | 7 | 5 | 10,905 | 2,243 | 13,172 | 1,702 | 14,874 | 81.91% |
| SAMN13563910 | 1 | 9 | 9 | 7 | 5 | 10,895 | 2,237 | 13,155 | 1,697 | 14,852 | 83.48% |
| SAMN13563911 | 1 | 9 | 9 | 7 | 8 | 10,906 | 2,246 | 13,178 | 1,700 | 14,878 | 81.95% |
| SAMN13563912 | 1 | 9 | 9 | 7 | 6 | 10,914 | 2,252 | 13,190 | 1,700 | 14,890 | 83.16% |
| SAMN13563913 | 1 | 9 | 9 | 7 | 7 | 10,907 | 2,251 | 13,183 | 1,706 | 14,889 | 83.25% |
| SAMN13563916 | 0 | 10 | 5 | 8 | 10 | 10,443 | 2,163 | 12,635 | 1,669 | 14,304 | 81.96% |
| SAMN13563917 | 0 | 8 | 8 | 10 | 9 | 10,829 | 2,258 | 13,114 | 1,750 | 14,864 | 80.11% |
| SAMN13563918 | 0 | 10 | 4 | 8 | 3 | 10,384 | 2,168 | 12,567 | 1,753 | 14,320 | 83.54% |
| SAMN13563920 | 0 | 7 | 7 | 8 | 9 | 9,759 | 2,089 | 11,870 | 1,521 | 13,391 | 81.34% |
| SAMN13563921 | 0 | 10 | 5 | 8 | 12 | 10,441 | 2,161 | 12,633 | 1,672 | 14,305 | 83.15% |
| SAMN13701845 | 1 | 9 | 9 | 7 | 7 | 10,896 | 2,235 | 13,156 | 1,695 | 14,851 | 83.16% |
| SAMN13702289 | 0 | 0 | 0 | 0 | 1 | 5 | 16 | 22 | 5 | 27 | 95.50% |
| SAMN13741660 | 1 | 9 | 10 | 7 | 6 | 10,898 | 2,247 | 13,170 | 1,702 | 14,872 | 83.82% |
| SAMN13915669 | 0 | 10 | 6 | 8 | 11 | 10,432 | 2,161 | 12,624 | 1,667 | 14,291 | 81.46% |
| SAMN13915723 | 0 | 11 | 5 | 9 | 9 | 10,439 | 2,159 | 12,628 | 1,676 | 14,304 | 81.90% |
| SAMN14168161 | 0 | 11 | 8 | 6 | 7 | 10,694 | 2,192 | 12,912 | 1,663 | 14,575 | 82.43% |
| SAMN14168461 | 0 | 7 | 6 | 9 | 6 | 10,772 | 2,257 | 13,047 | 1,790 | 14,837 | 81.10% |
| SAMN14168514 | 0 | 7 | 8 | 8 | 12 | 10,687 | 2,241 | 12,957 | 1,785 | 14,742 | 80.51% |
| SAMN14277886 | 1 | 9 | 10 | 7 | 4 | 10,900 | 2,246 | 13,168 | 1,707 | 14,875 | 83.12% |
| SAMN14540328 | 1 | 12 | 5 | 7 | 10 | 11,095 | 2,389 | 13,512 | 1,660 | 15,172 | 82.16% |
| SAMN15042437 | 0 | 7 | 7 | 8 | 10 | 9,756 | 2,092 | 11,871 | 1,521 | 13,392 | 82.06% |
| SAMN15331272 | 0 | 0 | 0 | 1 | 1 | 5 | 24 | 31 | 7 | 38 | 95.29% |
| SAMN15415360 | 1 | 9 | 10 | 7 | 5 | 10,905 | 2,259 | 13,188 | 1,709 | 14,897 | 83.72% |
| SAMN15415385 | 1 | 9 | 10 | 8 | 4 | 10,899 | 2,250 | 13,172 | 1,698 | 14,870 | 81.65% |
| SAMN15567838 | 0 | 7 | 8 | 7 | 9 | 10,682 | 2,228 | 12,935 | 1,792 | 14,727 | 81.35% |
| SAMN15816123 | 0 | 7 | 8 | 7 | 10 | 10,680 | 2,232 | 12,938 | 1,806 | 14,744 | 83.07% |
| SAMN15898396 | 0 | 7 | 8 | 7 | 13 | 10,692 | 2,244 | 12,965 | 1,777 | 14,742 | 80.98% |
| SAMN15898403 | 0 | 12 | 5 | 7 | 10 | 10,103 | 2,104 | 12,235 | 1,673 | 13,908 | 83.12% |
| SAMN15898404 | 0 | 8 | 7 | 6 | 9 | 10,881 | 2,253 | 13,158 | 1,783 | 14,941 | 82.66% |
| SAMN15898411 | 0 | 8 | 7 | 6 | 9 | 10,888 | 2,257 | 13,169 | 1,786 | 14,955 | 83.48% |
| SAMN15898420 | 0 | 7 | 8 | 7 | 11 | 10,683 | 2,228 | 12,938 | 1,778 | 14,716 | 80.99% |
| SAMN16094139 | 1 | 9 | 10 | 7 | 8 | 10,901 | 2,248 | 13,176 | 1,701 | 14,877 | 83.06% |
| SAMN16124401 | 1 | 9 | 8 | 7 | 4 | 10,894 | 2,252 | 13,167 | 1,706 | 14,873 | 83.32% |
| SAMN16124449 | 0 | 12 | 6 | 7 | 10 | 11,086 | 2,395 | 13,509 | 1,666 | 15,175 | 82.83% |
| SAMN16378163 | 1 | 8 | 6 | 8 | 11 | 10,383 | 2,180 | 12,592 | 1,622 | 14,214 | 83.16% |
| SAMN16378165 | 1 | 10 | 5 | 8 | 9 | 10,435 | 2,152 | 12,616 | 1,667 | 14,283 | 77.41% |
| SAMN16378166 | 1 | 8 | 6 | 8 | 11 | 10,386 | 2,188 | 12,603 | 1,620 | 14,223 | 80.70% |
| SAMN16378173 | 1 | 8 | 6 | 8 | 12 | 10,392 | 2,189 | 12,611 | 1,622 | 14,233 | 82.65% |
| SAMN16378175 | 1 | 9 | 9 | 7 | 5 | 10,900 | 2,250 | 13,173 | 1,702 | 14,875 | 82.93% |
| SAMN16378176 | 1 | 9 | 9 | 7 | 5 | 10,899 | 2,257 | 13,179 | 1,709 | 14,888 | 82.74% |
| SAMN16378177 | 1 | 8 | 6 | 8 | 11 | 10,390 | 2,172 | 12,591 | 1,623 | 14,214 | 81.99% |
| SAMN16378180 | 0 | 10 | 5 | 8 | 8 | 10,451 | 2,187 | 12,665 | 1,666 | 14,331 | 80.85% |
| SAMN16378182 | 1 | 9 | 9 | 7 | 6 | 10,902 | 2,259 | 13,185 | 1,708 | 14,893 | 81.49% |
| SAMN16378183 | 1 | 9 | 9 | 7 | 7 | 10,909 | 2,268 | 13,202 | 1,700 | 14,902 | 82.07% |
| SAMN16378184 | 1 | 9 | 9 | 7 | 7 | 10,909 | 2,265 | 13,199 | 1,700 | 14,899 | 81.88% |
| SAMN16378185 | 1 | 9 | 9 | 8 | 7 | 10,912 | 2,267 | 13,205 | 1,708 | 14,913 | 81.42% |
| SAMN16378187 | 1 | 9 | 9 | 7 | 7 | 10,909 | 2,269 | 13,203 | 1,706 | 14,909 | 83.01% |
| SAMN16378188 | 0 | 10 | 5 | 8 | 8 | 10,442 | 2,166 | 12,635 | 1,666 | 14,301 | 82.81% |
| SAMN16378189 | 1 | 8 | 6 | 8 | 11 | 10,393 | 2,206 | 12,628 | 1,624 | 14,252 | 82.13% |
| SAMN16378190 | 1 | 8 | 6 | 8 | 11 | 10,383 | 2,177 | 12,589 | 1,620 | 14,209 | 82.88% |
| SAMN16378191 | 1 | 8 | 6 | 8 | 12 | 10,383 | 2,177 | 12,590 | 1,621 | 14,211 | 82.95% |
| SAMN16378196 | 1 | 8 | 6 | 8 | 11 | 10,390 | 2,172 | 12,591 | 1,623 | 14,214 | 80.55% |
| SAMN16378197 | 1 | 10 | 5 | 8 | 9 | 10,436 | 2,152 | 12,617 | 1,667 | 14,284 | 81.91% |
| SAMN16378198 | 1 | 10 | 5 | 8 | 9 | 10,436 | 2,152 | 12,617 | 1,668 | 14,285 | 80.92% |
| SAMN16386297 | 1 | 9 | 9 | 8 | 7 | 10,910 | 2,264 | 13,200 | 1,700 | 14,900 | 84.59% |
| SAMN16427579 | 0 | 12 | 8 | 6 | 6 | 10,141 | 2,094 | 12,259 | 1,706 | 13,965 | 86.88% |
| SAMN16560577 | 0 | 9 | 7 | 7 | 11 | 10,590 | 2,310 | 12,927 | 1,654 | 14,581 | 82.97% |
| SAMN16560591 | 1 | 9 | 10 | 7 | 4 | 10,901 | 2,247 | 13,170 | 1,703 | 14,873 | 83.50% |
| SAMN16560593 | 0 | 10 | 5 | 8 | 9 | 10,445 | 2,158 | 12,631 | 1,668 | 14,299 | 83.65% |
| SAMN16560600 | 1 | 9 | 9 | 7 | 7 | 10,910 | 2,267 | 13,202 | 1,703 | 14,905 | 83.80% |
| SAMN16561285 | 1 | 9 | 9 | 8 | 8 | 10,916 | 2,260 | 13,203 | 1,707 | 14,910 | 84.77% |
| SAMN16561429 | 1 | 9 | 9 | 8 | 8 | 10,916 | 2,260 | 13,203 | 1,707 | 14,910 | 84.78% |
| SAMN16561436 | 1 | 9 | 10 | 7 | 5 | 10,903 | 2,248 | 13,175 | 1,704 | 14,879 | 82.72% |
| SAMN16561437 | 1 | 9 | 9 | 7 | 8 | 10,912 | 2,259 | 13,197 | 1,704 | 14,901 | 84.13% |
| SAMN16787939 | 0 | 10 | 5 | 8 | 10 | 10,445 | 2,171 | 12,645 | 1,665 | 14,310 | 82.89% |
| SAMN17075437 | 0 | 12 | 8 | 6 | 6 | 10,133 | 2,091 | 12,248 | 1,699 | 13,947 | 84.17% |
| SAMN17506720 | 2 | 8 | 4 | 7 | 6 | 11,065 | 2,311 | 13,398 | 1,712 | 15,110 | 81.13% |
| SAMN17766239 | 1 | 9 | 9 | 7 | 8 | 10,914 | 2,278 | 13,218 | 1,711 | 14,929 | 84.06% |
| SAMN17807608 | 1 | 9 | 9 | 7 | 4 | 10,906 | 2,249 | 13,177 | 1,702 | 14,879 | 83.07% |
| SAMN17807609 | 1 | 9 | 9 | 7 | 4 | 10,906 | 2,250 | 13,178 | 1,703 | 14,881 | 83.06% |
| SAMN17885214 | 1 | 8 | 6 | 8 | 10 | 10,381 | 2,173 | 12,582 | 1,619 | 14,201 | 81.30% |
| SAMN17976248 | 0 | 9 | 8 | 8 | 5 | 10,360 | 2,180 | 12,565 | 1,621 | 14,186 | 83.14% |
| SAMN18087500 | 0 | 0 | 0 | 0 | 0 | 7 | 14 | 21 | 4 | 25 | 94.38% |
| SAMN18087501 | 0 | 0 | 0 | 0 | 2 | 6 | 21 | 29 | 6 | 35 | 93.31% |
| SAMN18087505 | 0 | 0 | 0 | 0 | 0 | 5 | 23 | 28 | 4 | 32 | 93.78% |
| SAMN18106513 | 0 | 7 | 8 | 7 | 10 | 10,690 | 2,235 | 12,951 | 1,792 | 14,743 | 81.70% |
| SAMN18450110 | 0 | 8 | 7 | 6 | 9 | 10,882 | 2,245 | 13,151 | 1,779 | 14,930 | 81.71% |
| SAMN18450111 | 1 | 9 | 9 | 7 | 6 | 10,899 | 2,236 | 13,159 | 1,705 | 14,864 | 83.77% |
| SAMN18450112 | 1 | 12 | 5 | 7 | 10 | 11,089 | 2,382 | 13,499 | 1,660 | 15,159 | 81.82% |
| SAMN18511110 | 0 | 9 | 7 | 8 | 5 | 10,364 | 2,189 | 12,577 | 1,618 | 14,195 | 81.48% |
| SAMN18559365 | 0 | 10 | 7 | 11 | 9 | 10,714 | 2,275 | 13,020 | 1,722 | 14,742 | 80.71% |
| SAMN18874702 | 0 | 10 | 5 | 9 | 7 | 10,444 | 2,190 | 12,660 | 1,667 | 14,327 | 80.93% |
| SAMN18874754 | 0 | 12 | 5 | 7 | 8 | 10,101 | 2,119 | 12,246 | 1,669 | 13,915 | 83.66% |
| SAMN18874775 | 0 | 8 | 4 | 8 | 15 | 10,958 | 2,406 | 13,394 | 1,706 | 15,100 | 79.98% |
| SAMN18897823 | 0 | 7 | 8 | 7 | 9 | 10,687 | 2,235 | 12,947 | 1,788 | 14,735 | 81.89% |
| SAMN18897888 | 0 | 7 | 8 | 7 | 9 | 10,687 | 2,235 | 12,947 | 1,795 | 14,742 | 82.14% |
| SAMN19700739 | 1 | 8 | 6 | 9 | 11 | 10,386 | 2,184 | 12,600 | 1,622 | 14,222 | 82.04% |
| SAMN19700740 | 1 | 9 | 9 | 7 | 7 | 10,911 | 2,255 | 13,191 | 1,705 | 14,896 | 83.26% |
| SAMN19700741 | 1 | 9 | 9 | 7 | 6 | 10,914 | 2,258 | 13,196 | 1,704 | 14,900 | 83.14% |
| SAMN19700742 | 1 | 9 | 9 | 8 | 7 | 10,912 | 2,270 | 13,208 | 1,701 | 14,909 | 82.72% |
| SAMN19700743 | 1 | 9 | 10 | 7 | 6 | 10,918 | 2,277 | 13,220 | 1,702 | 14,922 | 83.29% |
| SAMN19700744 | 1 | 9 | 9 | 7 | 7 | 10,907 | 2,258 | 13,190 | 1,710 | 14,900 | 83.41% |
| SAMN19700745 | 1 | 9 | 9 | 7 | 6 | 10,905 | 2,248 | 13,177 | 1,703 | 14,880 | 81.81% |
| SAMN19700746 | 1 | 9 | 9 | 7 | 7 | 10,907 | 2,255 | 13,187 | 1,703 | 14,890 | 83.27% |
| SAMN19700747 | 1 | 9 | 9 | 7 | 7 | 10,904 | 2,256 | 13,185 | 1,703 | 14,888 | 81.96% |
| SAMN19700748 | 0 | 5 | 6 | 10 | 5 | 10,378 | 2,200 | 12,597 | 1,763 | 14,360 | 83.27% |
| SAMN19700749 | 0 | 12 | 5 | 8 | 3 | 10,387 | 2,168 | 12,571 | 1,755 | 14,326 | 82.44% |
| SAMN19700757 | 2 | 8 | 6 | 9 | 16 | 10,876 | 2,348 | 13,257 | 1,698 | 14,955 | 81.71% |
| SAMN19700758 | 0 | 10 | 5 | 8 | 10 | 10,453 | 2,164 | 12,646 | 1,668 | 14,314 | 82.10% |
| SAMN19700759 | 0 | 10 | 5 | 8 | 8 | 10,440 | 2,171 | 12,638 | 1,668 | 14,306 | 83.83% |
| SAMN19700760 | 0 | 10 | 5 | 8 | 10 | 10,449 | 2,162 | 12,640 | 1,666 | 14,306 | 82.05% |
| SAMN19700761 | 0 | 10 | 5 | 8 | 10 | 10,449 | 2,157 | 12,635 | 1,669 | 14,304 | 82.06% |
| SAMN19700762 | 0 | 10 | 6 | 8 | 10 | 10,439 | 2,171 | 12,640 | 1,668 | 14,308 | 83.06% |
| SAMN19843951 | 0 | 10 | 5 | 8 | 8 | 10,442 | 2,171 | 12,640 | 1,671 | 14,311 | 82.80% |
| SAMN20400403 | 0 | 7 | 8 | 7 | 9 | 10,697 | 2,243 | 12,965 | 1,779 | 14,744 | 80.13% |
| SAMN20834176 | 0 | 7 | 8 | 7 | 9 | 10,683 | 2,237 | 12,945 | 1,800 | 14,745 | 81.36% |
| SAMN20834183 | 0 | 9 | 7 | 8 | 6 | 10,363 | 2,191 | 12,579 | 1,622 | 14,201 | 81.38% |
| SAMN20966673 | 1 | 9 | 9 | 7 | 5 | 10,895 | 2,231 | 13,149 | 1,697 | 14,846 | 83.31% |
| SAMN20966683 | 0 | 12 | 5 | 7 | 11 | 10,100 | 2,109 | 12,238 | 1,671 | 13,909 | 84.28% |
| SAMN20982458 | 1 | 9 | 9 | 7 | 6 | 10,914 | 2,270 | 13,208 | 1,702 | 14,910 | 83.64% |
| SAMN20982460 | 0 | 8 | 4 | 8 | 14 | 10,965 | 2,402 | 13,396 | 1,712 | 15,108 | 81.44% |
| SAMN21237641 | 0 | 7 | 8 | 7 | 9 | 10,689 | 2,234 | 12,948 | 1,797 | 14,745 | 81.21% |
| SAMN21237643 | 2 | 8 | 6 | 9 | 15 | 10,884 | 2,350 | 13,266 | 1,706 | 14,972 | 81.74% |
| SAMN21479087 | 0 | 9 | 7 | 8 | 5 | 10,363 | 2,182 | 12,569 | 1,620 | 14,189 | 83.50% |
| SAMN22024789 | 0 | 10 | 5 | 8 | 9 | 10,440 | 2,153 | 12,621 | 1,666 | 14,287 | 83.80% |
| SAMN22024790 | 1 | 9 | 9 | 7 | 7 | 10,903 | 2,257 | 13,185 | 1,710 | 14,895 | 82.87% |
| SAMN22024799 | 0 | 7 | 8 | 10 | 10 | 10,836 | 2,255 | 13,118 | 1,749 | 14,867 | 83.70% |
| SAMN22108512 | 1 | 8 | 6 | 8 | 9 | 10,384 | 2,183 | 12,594 | 1,623 | 14,217 | 83.39% |
| SAMN22211739 | 0 | 12 | 8 | 6 | 5 | 10,129 | 2,092 | 12,244 | 1,703 | 13,947 | 85.75% |
| SAMN22567294 | 1 | 9 | 9 | 7 | 4 | 10,901 | 2,236 | 13,159 | 1,702 | 14,861 | 82.73% |
| SAMN23002890 | 1 | 9 | 9 | 7 | 7 | 10,909 | 2,262 | 13,196 | 1,706 | 14,902 | 83.93% |
| SAMN23898336 | 1 | 9 | 9 | 7 | 6 | 10,912 | 2,259 | 13,195 | 1,709 | 14,904 | 84.52% |
| SAMN24108702 | 0 | 9 | 7 | 8 | 5 | 10,362 | 2,176 | 12,562 | 1,619 | 14,181 | 81.61% |
| SAMN24108732 | 1 | 9 | 9 | 7 | 8 | 10,906 | 2,254 | 13,186 | 1,704 | 14,890 | 84.53% |
| SAMN24372389 | 1 | 9 | 9 | 7 | 7 | 10,909 | 2,251 | 13,185 | 1,702 | 14,887 | 83.82% |
| SAMN24562924 | 1 | 9 | 9 | 7 | 6 | 10,912 | 2,257 | 13,193 | 1,708 | 14,901 | 84.36% |
| SAMN24562925 | 1 | 9 | 9 | 7 | 6 | 10,913 | 2,261 | 13,198 | 1,709 | 14,907 | 84.34% |
| SAMN24664061 | 0 | 0 | 0 | 0 | 0 | 6 | 23 | 29 | 5 | 34 | 92.49% |
| SAMN24665688 | 0 | 7 | 8 | 7 | 9 | 10,685 | 2,234 | 12,944 | 1,786 | 14,730 | 80.89% |
| SAMN24667610 | 0 | 0 | 0 | 0 | 0 | 6 | 17 | 23 | 7 | 30 | 94.18% |
| SAMN24669135 | 0 | 0 | 0 | 0 | 0 | 5 | 22 | 27 | 6 | 33 | 93.07% |
| SAMN24814189 | 1 | 9 | 9 | 7 | 7 | 10,910 | 2,265 | 13,200 | 1,708 | 14,908 | 82.64% |
| SAMN24814194 | 0 | 9 | 7 | 8 | 5 | 10,363 | 2,184 | 12,571 | 1,618 | 14,189 | 81.76% |
| SAMN24814206 | 0 | 12 | 5 | 7 | 11 | 10,107 | 2,105 | 12,241 | 1,671 | 13,912 | 83.58% |
| SAMN24814214 | 1 | 9 | 3 | 8 | 17 | 10,968 | 2,397 | 13,398 | 1,704 | 15,102 | 81.42% |
| SAMN24814228 | 1 | 9 | 9 | 7 | 6 | 10,906 | 2,263 | 13,193 | 1,703 | 14,896 | 82.41% |
| SAMN24814231 | 0 | 7 | 8 | 7 | 12 | 10,685 | 2,234 | 12,947 | 1,781 | 14,728 | 81.38% |
| SAMN24814241 | 1 | 9 | 9 | 7 | 9 | 10,906 | 2,246 | 13,179 | 1,700 | 14,879 | 83.05% |
| SAMN25039377 | 1 | 9 | 9 | 7 | 6 | 10,900 | 2,246 | 13,170 | 1,700 | 14,870 | 83.40% |
| SAMN25039387 | 0 | 10 | 5 | 8 | 9 | 10,448 | 2,171 | 12,647 | 1,667 | 14,314 | 82.83% |
| SAMN25084274 | 0 | 7 | 7 | 8 | 10 | 9,746 | 2,095 | 11,864 | 1,522 | 13,386 | 81.25% |
| SAMN25378372 | 1 | 9 | 9 | 7 | 7 | 10,907 | 2,242 | 13,174 | 1,699 | 14,873 | 84.74% |
| SAMN25608758 | 1 | 9 | 9 | 7 | 6 | 10,904 | 2,257 | 13,185 | 1,705 | 14,890 | 83.43% |
| SAMN25761396 | 1 | 13 | 7 | 9 | 3 | 10,533 | 2,141 | 12,697 | 1,628 | 14,325 | 86.30% |
| SAMN25894731 | 0 | 12 | 8 | 6 | 5 | 10,137 | 2,095 | 12,255 | 1,706 | 13,961 | 85.22% |
| SAMN25953854 | 0 | 10 | 4 | 8 | 5 | 10,382 | 2,157 | 12,556 | 1,754 | 14,310 | 81.25% |
| SAMN26012048 | 1 | 9 | 9 | 7 | 4 | 10,895 | 2,228 | 13,145 | 1,696 | 14,841 | 81.44% |
| SAMN26012836 | 1 | 9 | 10 | 7 | 5 | 10,905 | 2,259 | 13,188 | 1,703 | 14,891 | 82.98% |
| SAMN26012843 | 1 | 9 | 9 | 7 | 8 | 10,910 | 2,254 | 13,190 | 1,706 | 14,896 | 82.62% |
| SAMN26012847 | 0 | 7 | 8 | 7 | 9 | 10,687 | 2,228 | 12,940 | 1,794 | 14,734 | 80.46% |
| SAMN26012945 | 1 | 9 | 10 | 7 | 8 | 10,908 | 2,261 | 13,196 | 1,706 | 14,902 | 84.35% |
| SAMN26012975 | 0 | 10 | 5 | 8 | 10 | 10,442 | 2,175 | 12,646 | 1,665 | 14,311 | 83.71% |
| SAMN26018321 | 1 | 9 | 10 | 7 | 5 | 10,897 | 2,247 | 13,168 | 1,701 | 14,869 | 81.35% |
| SAMN26019433 | 1 | 9 | 9 | 7 | 5 | 10,897 | 2,237 | 13,157 | 1,701 | 14,858 | 83.08% |
| SAMN26020063 | 1 | 9 | 9 | 7 | 7 | 10,910 | 2,261 | 13,196 | 1,704 | 14,900 | 84.32% |
| SAMN26020066 | 0 | 7 | 5 | 8 | 13 | 10,980 | 2,324 | 13,325 | 1,759 | 15,084 | 80.05% |
| SAMN26020162 | 0 | 12 | 5 | 7 | 9 | 10,110 | 2,108 | 12,245 | 1,680 | 13,925 | 83.30% |
| SAMN26020253 | 0 | 12 | 4 | 9 | 10 | 11,455 | 2,447 | 13,928 | 1,864 | 15,792 | 81.22% |
| SAMN26020259 | 0 | 7 | 5 | 8 | 14 | 10,979 | 2,323 | 13,324 | 1,757 | 15,081 | 80.00% |
| SAMN26020309 | 0 | 10 | 5 | 8 | 10 | 10,441 | 2,171 | 12,641 | 1,666 | 14,307 | 83.64% |
| SAMN26021135 | 0 | 10 | 5 | 8 | 8 | 10,444 | 2,172 | 12,643 | 1,666 | 14,309 | 83.59% |
| SAMN26021138 | 0 | 8 | 7 | 6 | 9 | 10,888 | 2,252 | 13,164 | 1,781 | 14,945 | 80.44% |
| SAMN26498263 | 2 | 8 | 7 | 7 | 8 | 8,071 | 1,752 | 9,852 | 1,373 | 11,225 | 83.60% |
| SAMN26498264 | 2 | 8 | 7 | 7 | 8 | 8,070 | 1,753 | 9,852 | 1,373 | 11,225 | 83.66% |
| SAMN26498269 | 1 | 9 | 9 | 7 | 8 | 10,907 | 2,247 | 13,180 | 1,702 | 14,882 | 84.47% |
| SAMN26498271 | 1 | 9 | 9 | 7 | 5 | 10,908 | 2,242 | 13,173 | 1,703 | 14,876 | 84.39% |
| SAMN26554413 | 0 | 11 | 5 | 8 | 8 | 10,450 | 2,185 | 12,662 | 1,686 | 14,348 | 84.03% |
| SAMN26554414 | 1 | 9 | 9 | 7 | 7 | 10,925 | 2,270 | 13,220 | 1,712 | 14,932 | 83.04% |
| SAMN26554415 | 1 | 9 | 9 | 9 | 7 | 10,924 | 2,268 | 13,219 | 1,721 | 14,940 | 83.02% |
| SAMN26750072 | 1 | 9 | 9 | 8 | 4 | 10,926 | 2,326 | 13,275 | 1,713 | 14,988 | 82.97% |
| SAMN26750073 | 0 | 13 | 8 | 9 | 22 | 10,626 | 2,402 | 13,074 | 1,714 | 14,788 | 80.33% |
| SAMN26750074 | 1 | 9 | 9 | 8 | 5 | 10,921 | 2,354 | 13,299 | 1,715 | 15,014 | 83.20% |
| SAMN26750075 | 0 | 14 | 8 | 9 | 20 | 10,615 | 2,370 | 13,030 | 1,711 | 14,741 | 79.68% |
| SAMN26750076 | 1 | 9 | 9 | 7 | 6 | 10,914 | 2,272 | 13,210 | 1,705 | 14,915 | 83.43% |
| SAMN26750077 | 0 | 9 | 4 | 8 | 16 | 10,971 | 2,452 | 13,455 | 1,718 | 15,173 | 81.00% |
| SAMN26750078 | 0 | 7 | 8 | 7 | 10 | 10,676 | 2,235 | 12,937 | 1,782 | 14,719 | 80.02% |
| SAMN26750079 | 0 | 13 | 8 | 11 | 22 | 10,623 | 2,382 | 13,053 | 1,714 | 14,767 | 80.09% |
| SAMN26750080 | 2 | 10 | 9 | 8 | 7 | 10,915 | 2,272 | 13,215 | 1,711 | 14,926 | 83.01% |
| SAMN26750081 | 0 | 8 | 4 | 8 | 16 | 10,962 | 2,420 | 13,413 | 1,709 | 15,122 | 80.79% |
| SAMN26750082 | 0 | 8 | 9 | 9 | 19 | 10,847 | 2,416 | 13,299 | 1,788 | 15,087 | 81.45% |
| SAMN26750084 | 0 | 5 | 6 | 10 | 6 | 10,366 | 2,208 | 12,594 | 1,761 | 14,355 | 82.28% |
| SAMN26750085 | 1 | 10 | 9 | 7 | 6 | 10,914 | 2,246 | 13,185 | 1,702 | 14,887 | 82.43% |
| SAMN26750086 | 0 | 8 | 8 | 10 | 9 | 10,839 | 2,272 | 13,138 | 1,755 | 14,893 | 81.13% |
| SAMN26750087 | 1 | 9 | 9 | 7 | 5 | 10,895 | 2,234 | 13,152 | 1,697 | 14,849 | 82.91% |
| SAMN26750088 | 0 | 8 | 4 | 8 | 16 | 10,958 | 2,419 | 13,408 | 1,712 | 15,120 | 80.35% |
| SAMN26750089 | 0 | 10 | 7 | 8 | 11 | 10,740 | 2,286 | 13,056 | 1,724 | 14,780 | 80.54% |
| SAMN26750090 | 0 | 7 | 8 | 7 | 9 | 10,685 | 2,248 | 12,958 | 1,779 | 14,737 | 80.27% |
| SAMN26750091 | 0 | 8 | 8 | 9 | 17 | 10,837 | 2,388 | 13,258 | 1,791 | 15,049 | 80.65% |
| SAMN26816214 | 1 | 9 | 8 | 7 | 7 | 10,917 | 2,260 | 13,201 | 1,699 | 14,900 | 81.62% |
| SAMN27162961 | 0 | 10 | 5 | 8 | 10 | 10,448 | 2,157 | 12,634 | 1,668 | 14,302 | 83.66% |
| SAMN27363256 | 0 | 0 | 0 | 1 | 1 | 6 | 23 | 31 | 10 | 41 | 95.10% |
| SAMN27593773 | 0 | 7 | 8 | 7 | 9 | 10,690 | 2,231 | 12,946 | 1,796 | 14,742 | 80.48% |
| SAMN28095484 | 0 | 0 | 0 | 1 | 1 | 7 | 24 | 33 | 7 | 40 | 95.10% |
| SAMN28187128 | 1 | 9 | 9 | 7 | 6 | 10,904 | 2,247 | 13,175 | 1,702 | 14,877 | 81.83% |
| SAMN28447051 | 1 | 9 | 9 | 7 | 5 | 10,898 | 2,250 | 13,171 | 1,705 | 14,876 | 84.44% |
| SAMN28447209 | 1 | 9 | 8 | 7 | 8 | 10,914 | 2,262 | 13,201 | 1,701 | 14,902 | 81.46% |
| SAMN28447278 | 0 | 10 | 5 | 8 | 8 | 10,443 | 2,166 | 12,636 | 1,667 | 14,303 | 82.91% |
| SAMN28447312 | 1 | 9 | 8 | 7 | 8 | 10,913 | 2,261 | 13,199 | 1,701 | 14,900 | 81.42% |
| SAMN28447345 | 0 | 10 | 5 | 8 | 8 | 10,443 | 2,166 | 12,636 | 1,667 | 14,303 | 82.93% |
| SAMN28729100 | 0 | 11 | 5 | 10 | 6 | 10,165 | 2,137 | 12,323 | 1,664 | 13,987 | 82.73% |
| SAMN28818454 | 0 | 0 | 0 | 0 | 1 | 5 | 27 | 33 | 9 | 42 | 93.05% |
| SAMN28818459 | 0 | 0 | 0 | 0 | 1 | 6 | 27 | 34 | 9 | 43 | 93.06% |
| SAMN29787424 | 0 | 10 | 5 | 8 | 8 | 10,440 | 2,167 | 12,634 | 1,665 | 14,299 | 81.66% |
| SAMN29889749 | 1 | 8 | 6 | 8 | 9 | 10,382 | 2,165 | 12,574 | 1,622 | 14,196 | 83.58% |
| SAMN30160477 | 0 | 0 | 0 | 0 | 0 | 7 | 25 | 32 | 4 | 36 | 88.64% |
| SAMN30160479 | 0 | 7 | 8 | 7 | 9 | 10,692 | 2,249 | 12,966 | 1,798 | 14,764 | 81.32% |
| SAMN30192241 | 0 | 0 | 0 | 1 | 0 | 5 | 21 | 27 | 4 | 31 | 93.12% |
| SAMN30192242 | 0 | 0 | 0 | 1 | 0 | 5 | 21 | 27 | 4 | 31 | 93.22% |
| SAMN30192243 | 0 | 0 | 0 | 1 | 0 | 5 | 21 | 27 | 4 | 31 | 93.15% |
| SAMN30192245 | 0 | 0 | 0 | 1 | 0 | 5 | 21 | 27 | 4 | 31 | 93.21% |
| SAMN30192257 | 0 | 0 | 0 | 1 | 0 | 5 | 21 | 27 | 4 | 31 | 92.00% |
| SAMN30192258 | 0 | 0 | 0 | 1 | 0 | 6 | 21 | 28 | 4 | 32 | 92.70% |
| SAMN30192259 | 0 | 0 | 0 | 0 | 0 | 7 | 23 | 30 | 6 | 36 | 93.73% |
| SAMN30203660 | 0 | 8 | 7 | 6 | 9 | 10,892 | 2,248 | 13,164 | 1,779 | 14,943 | 78.47% |
| SAMN30252885 | 1 | 9 | 9 | 7 | 7 | 10,912 | 2,265 | 13,202 | 1,704 | 14,906 | 84.87% |
| SAMN31888397 | 0 | 0 | 0 | 1 | 0 | 7 | 22 | 30 | 7 | 37 | 92.57% |
| SAMN31888398 | 0 | 1 | 0 | 0 | 0 | 330 | 83 | 414 | 83 | 497 | 91.04% |
| SAMN31888399 | 0 | 1 | 0 | 0 | 0 | 327 | 76 | 404 | 82 | 486 | 90.65% |
| SAMN31888400 | 0 | 0 | 0 | 0 | 0 | 9 | 23 | 32 | 6 | 38 | 92.91% |
| SAMN31888401 | 0 | 0 | 0 | 0 | 0 | 9 | 23 | 32 | 6 | 38 | 94.00% |
| SAMN31888402 | 0 | 0 | 0 | 0 | 0 | 6 | 14 | 20 | 5 | 25 | 95.87% |
| SAMN31888403 |  |  |  |  |  |  |  |  |  |  | 100.00% |

Annotion: The alignment results of the genome coverage between CR-hvKP221 and 402 strains are shown in the last column.
